# Synthesis of quaternary centres by single electron reduction and alkylation of alkylsulfones†‡

**DOI:** 10.1039/d1sc00133g

**Published:** 2021-02-04

**Authors:** Masakazu Nambo, Yasuyo Tahara, Jacky C.-H. Yim, Daisuke Yokogawa, Cathleen M. Crudden

**Affiliations:** Institute of Transformative Bio-Molecules (WPI-ITbM), Nagoya University Chikusa Nagoya Aichi 464-8601 Japan mnambo@itbm.nagoya-u.ac.jp; Graduate School of Arts and Science, The University of Tokyo Komaba, Meguro-ku Tokyo 153-8902 Japan; Department of Chemistry, Queen's University Chernoff Hall Kingston Ontario K7L 3N6 Canada cruddenc@chem.queensu.ca

## Abstract

A new method for the generation of tertiary radicals through single electron reduction of alkylsulfones promoted by Zn and 1,10-phenanthroline has been developed. These radicals could be employed in the Giese reaction, affording structurally diverse quaternary products in good yields. With the high modularity and functional group compatibility of sulfones, the utility of this method was demonstrated by intramolecular and iterative reactions to give complex structures. The radical generation process was investigated by control experiments and theoretical calculations.

## Abstract 2

A new method for the generation of tertiary radicals through single electron reduction of alkylsulfones promoted by Zn and 1,10-phenanthroline has been developed.

## Graphical abstract

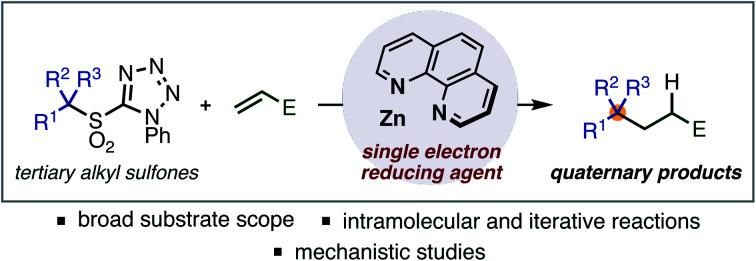

## Introduction

**Scheme 1:**
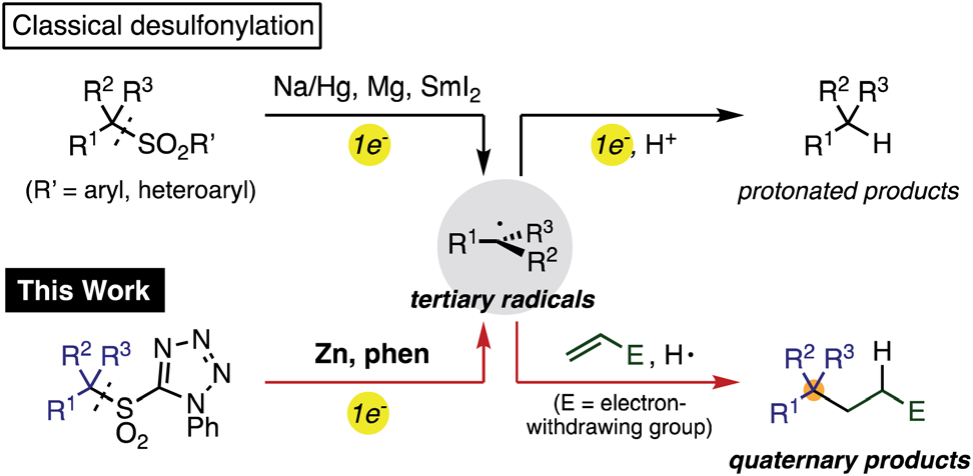
Generation of radicals from alkylsulfones by single electron reducing agent.

Organosulfones are versatile intermediates in organic synthesis because of the ease with which they permit facile structural modification through α-functionalization or conjugate addition.^1^ Due to the inherent stability of sulfonyl groups, strong reducing agents such as Na amalgam and Mg are generally required for their removal (Scheme 1).^2^ However, a stepwise alkylative desulfonylation process would be much more valuable. In this process, single electron transfer to the sulfonyl group by a reductant would fragment into a sulfinic anion and a sp^3^-carbon radical, which could be trapped with organic electrophiles rather than merely reduced and protonated. Thus, a controlled single electron reduction would have the potential to establish a new method for the generation of carbon radicals in organic synthesis and expand the utility of sulfones.^3^

Recently, organosulfones have attracted considerable attention in cross-coupling reactions as a new class of electrophiles.^4^ Our group and others have developed several transition-metal catalyzed cross-coupling reactions of functionalized aromatic and benzylic sulfones *via* carbon–sulfonyl (C–SO_2_) bond activation.^5^ Substituents on the sulfonyl group were found to provide a powerful new avenue for controlling reactivity. For example, strongly electron-withdrawing substituents such as 3,5-bis(trifluoromethyl)phenyl^5*b*,*d*,*e*^ and trifluoromethyl groups^5*f*,*g*^ not only facilitate α-functionalization, but also promote C–SO_2_ bond activation, enabling the rapid synthesis of multiply-arylated structures. In Baran's Ni-catalyzed radical cross-coupling of primary and secondary alkylsulfones with arylzinc reagents,^4*d*^ tetrazolyl group^7^ as the sulfone substituent was critical to enable the desired reaction. Hu,^6*a*^ and others^6*b*–*d*^ reported a radical fluoroalkylations using fluoroalkylsulfones, and Gouverneur and Davis recently established a photocatalytic radical modification of proteins.^6*e*^

Building on these reports, we envisioned a new method for generating reactive alkyl radicals by careful selection of a suitable sulfonyl substituent^8^ and reducing agent. Herein we report a simple method for the conversion of tertiary alkylsulfones to tertiary alkyl radicals, and their alkylation to generate quaternary carbon-centers. This reaction proceeds efficiently using readily available Zn powder with 1,10-phenanthroline (phen) as a new single electron reducing agent. The key reducing species generated by Zn and phen was investigated by control experiments and theoretical calculations.

## Results and discussion

We began our investigation by studying the Giese reaction^9^ of 3-phenyl-1,1-dimethylpropyl-1-phenyltetrazolyl sulfone **1a** with benzyl acrylate **2a** as a model reaction at room temperature (Table 1). Optimized conditions include the use of Hantzsch ester as a proton source in DMF, and the combination of Zn powder (particle size <10 μm, 2.5 equiv.) with phen (3 equiv.), giving the quaternary product **3aa** in 82% isolated yield (entry 1).

**Table 1:** Optimization of Zn/phen-mediated Giese reaction of tertiary sulfone **1a** with benzyl acrylate **2a**^a^

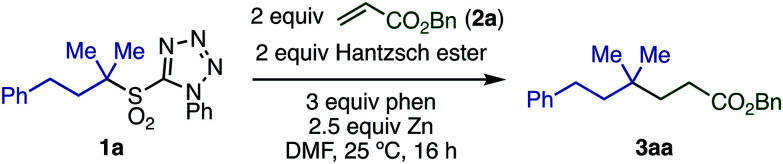		
Entry	Deviations from above	Yield^b^ [%]
1	None	84(82)
2	No Zn	0
3	No phen	0
4	Zn pre-activated by TMSCl, no phen	0
5	Ricke Zn, no phen	0
6	Zn (particle size 75–150 μm)	0
7	Mg or Mn instead of Zn	0
8	1.5 equiv. Hantzsch ester	67
9	DMAP or bpy instead of phen	0
10	Terpy instead of phen	25
11	2 equiv. phen	61
12	50 °C	77
13	2 equiv. LiCl	58
14	10 mol% NiCl_2_, 2 equiv. MgCl_2_	59
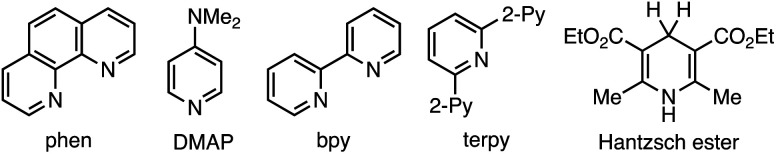		

a Conditions: **1a** (0.1 mmol), **2a** (2 equiv.), Hantzsch ester (2 equiv.), phen (3 equiv.), Zn (particle size < 10 μm, 2.5 equiv.), DMF (0.2 M), 25 °C, 16 h.

b Yields were determined by ^1^H NMR using anisole as an internal standard. Isolated yield is shown in parentheses (0.2 mmol scale).

Both Zn and phen were essential for this reaction (entries 2 and 3). Even the use of Zn activated by TMSCl^10^ or Rieke zinc^11^ showed no reactivity in the absence of phen, indicating that the ligand does more than simply activate the Zn surface. Using Zn with a larger particle size (75–150 μm) or other metals, such as Mg and Mn, also in the presence of phen, shut down the reaction (entries 4–6). The use of 1.5 equiv. Hantzsch ester decreased the yield of product (entry 8). Other *N*-heteroarene additives including 4-dimethylaminopyridine (DMAP), 2,2′-bipyridyl (bpy), and 2,2′:6′,2′′-terpyridine (terpy) instead of phen also inhibited product formation (entries 9 and 10). Reducing the amount of phen to 2 equiv. resulted in a decreased yield (entry 11). Product yield was not improved by increasing the reaction temperature (entry 12). Other additives that have been employed in similar transformations were examined^12^ but did not show any beneficial effect in this reaction (entries 13 and 14).

**Scheme 2:**
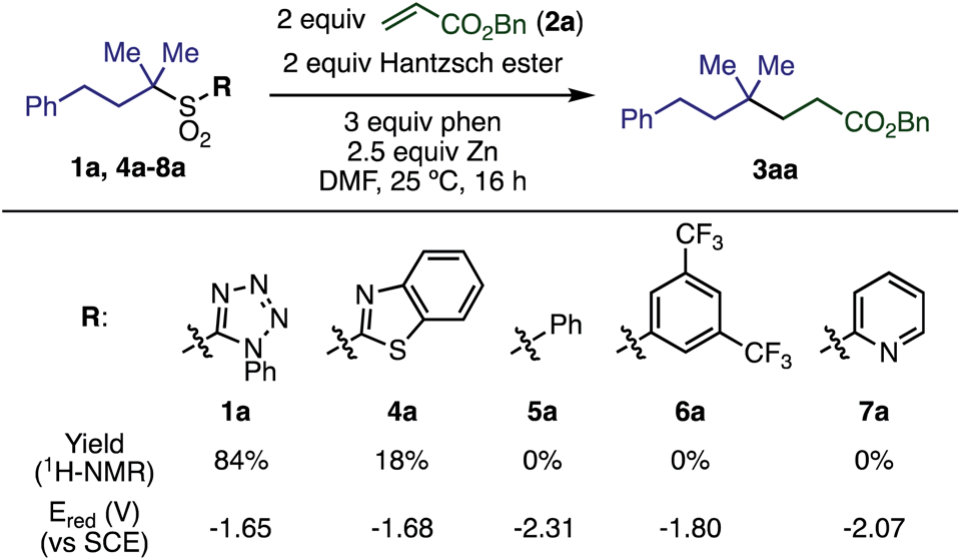
Effect of sulfonyl substituents.

The substituent on the sulfonyl group was found to be a key factor for radical generation (Scheme 2). 2-Benzothiazolyl sulfone (**4a**) was converted into **3aa**, albeit in poor yield. Other sulfones with substituents such as phenyl (**5a**), 3,5-bis(trifluoromethyl)phenyl (**6a**), and 2-pyridyl (**7a**) were completely unreactive. The first reduction potentials (*E*_red_) of sulfones were measured to evaluate the ease of single electron reduction, and were shown to correlate with reactivity. Specifically, sulfones **1a** and **4a** were the easiest to reduce, and the only two to show any product under the conditions examined.

**Scheme 3:**
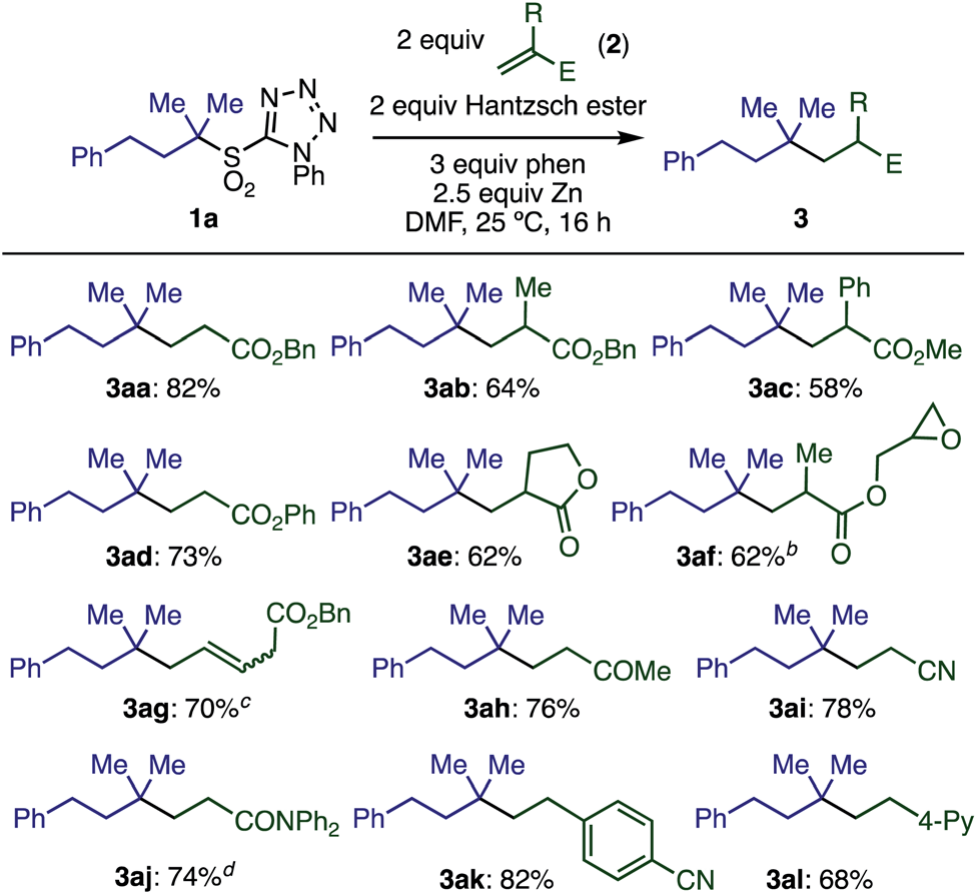
Substrate scope with respect to olefins.^*a*^Conditions: **1a** (0.2 mmol), **2a** (2 equiv.), Hantzsch ester (2 equiv.), phen (3 equiv.), Zn (particle size < 10 μm, 2.5 equiv.), DMF (0.2 M), 25 °C, 16 h. ^*b*^1 : 1 ratio of diastereomers. ^*c*^*trans* : *cis* = 17 : 1. ^*d*^50 °C.

With optimized conditions in hand, we investigated the substrate scope of electron-deficient olefins **2** (Scheme 3). A variety of acrylates having substituents in the 2-position (**2a–2e**) gave the desired products in good yields. Interestingly, the sensitive oxirane moiety in acrylate **2f** was compatible with the reaction conditions, and conjugated olefin **2g** gave the product from selective reaction at the terminal position. Other electron-deficient olefins, including vinyl ketone **2h** and acrylonitrile **2i**, reacted smoothly. Although acrylamide **2j** was less reactive, the yield of **3aj** could be improved to 74% by running the reaction at 50 °C.

Electron-poor styrene derivatives (**2k**, **2l**) were also viable substrates, giving products **3ak** and **3al** in good yields. Simple styrenes without electron withdrawing groups or acrylate derivatives with steric hindrance at the beta-position were not reactive. For full details see ESI Table S6.‡

**Scheme 4:**
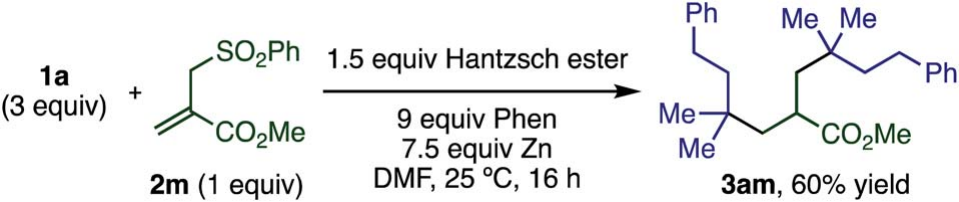
Double radical addition to allylic sulfone derivative.

When the more complex allyl sulfone **2m** bearing an ester moiety was reacted with excess **1a**, the doubly alkylated product **3ma** was formed through radical addition and then subsequent elimination of the sulfonyl group followed by the Giese reaction (Scheme 4).

**Scheme 5:**
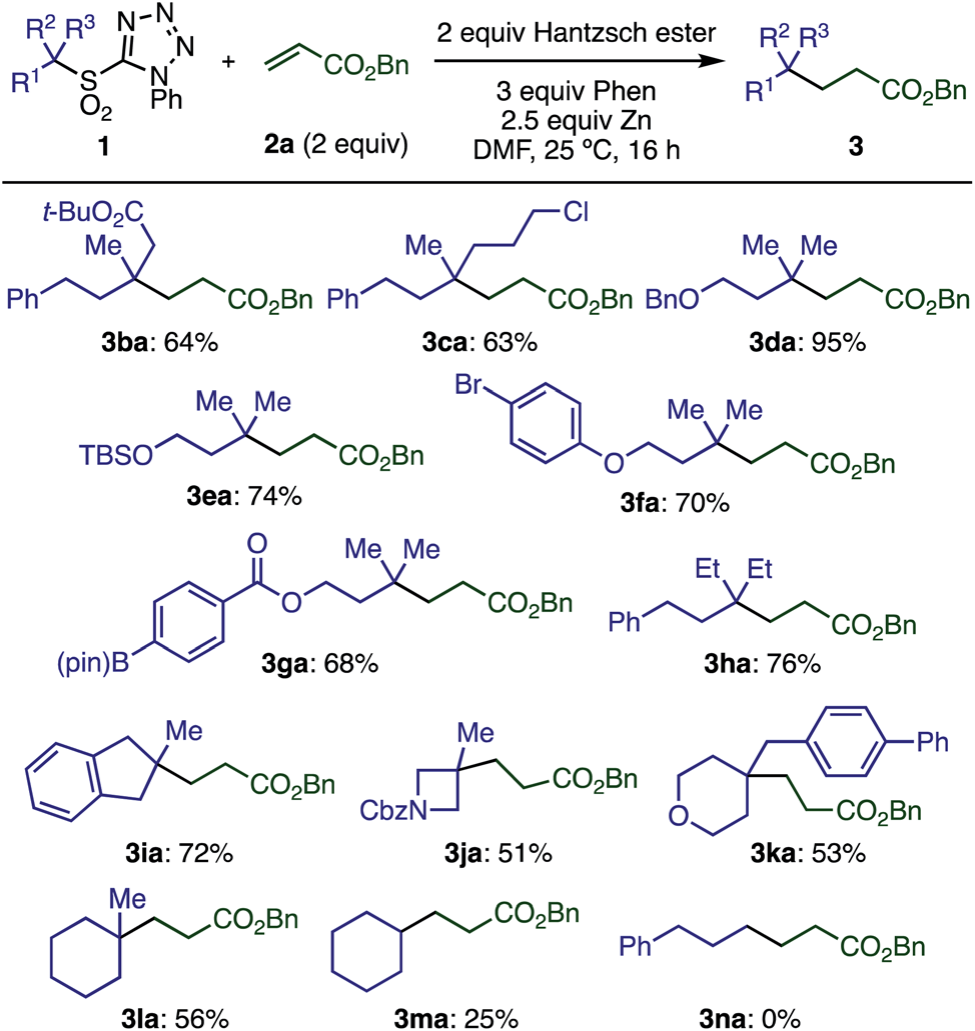
Substrate scope with respect to sulfones.^*a*^Conditions: **1a** (0.1 mmol), **2a** (2 equiv.), Hantzsch ester (2 equiv.), phen (3 equiv.), Zn (particle size < 10 μm, 2.5 equiv.), DMF (0.2 M), 25 °C, 16 h.

Next, the scope of tertiary sulfones **1** was evaluated (Scheme 5). These substrates were readily prepared by α-alkylation of primary or secondary sulfones with high functional group compatibility. Acyclic sulfones bearing various functional groups including ester, chloride, benzyloxy, silyloxy, bromoaryl, and boryl groups (**1b–1g**) were well tolerated, which is important for further transformations. Sterically hindered radicals generated from sulfone **1h** gave the desired product (**3ha**) without loss of yield. Cyclic tertiary sulfones with different ring sizes (**1i–1l**) were also suitable substrates, however, secondary sulfones gave decreased yields of the desired product (**3ma**) due to the formation of a by-product derived from the addition of sulfinic radical to the olefin. Primary sulfone **1n** showed no conversion under optimized conditions, likely because the generation of a primary radical is unfavorable.

**Fig. 1:**
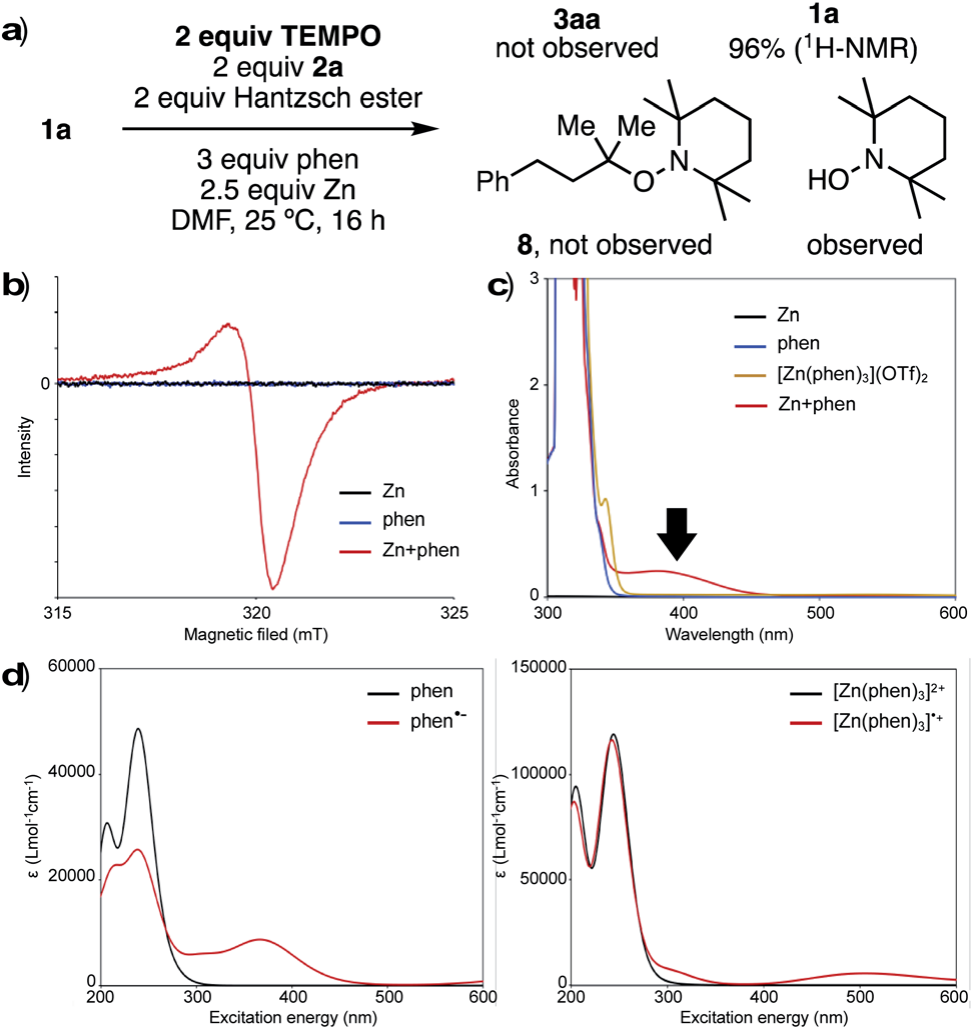
Investigation of reducing species: (a) reaction mixture in the presence of TEMPO. (b) The electron paramagnetic resonance (EPR) spectra. (c) UV-Vis absorption spectra. (d) UV-Vis absorption spectra calculated by TD-DFT. Geometry optimizations were performed at CAM-B3LYP/6-31+G* level coupled with SMD (solvent = DMF). By using optimized geometries, spectra were computed using TD-DFT (cc-pVDZ basis set for H, C, Zn, and aug-cc-pVDZ for N).

To investigate the nature of the key reducing agent for radical generation, we performed several control experiments. When the reaction of **1a** with **2a** was conducted under standard conditions in the presence of 2,2,6,6-tetramethylpiperidine-1-oxy (TEMPO), neither product **3a** nor TEMPO-adduct **8** was observed, and **1a** was recovered quantitatively (Fig. 1a). In addition, 1-hydroxy-2,2,6,6-tetramethylpiperidine (TEMPO-OH) was detected by GCMS and LCMS analysis. This suggests that generated reductant has radical character, and the single electron transfer process can be inhibited before desulfonylation. When Zn and phen were stirred in DMF at room temperature, a red/purple-colored mixture was obtained. This mixture was analyzed by an electron paramagnetic resonance (EPR), and an EPR signal was observed (*g* = 2.0017), clearly indicating that a radical species is generated from Zn and phen (Fig. 1b). One reasonable hypothesis is that Zn reduces phen to form reactive phen radical anion (phen˙^−^)^13^ and [Zn(phen)_3_]˙^+^ species.

UV-Vis spectroscopic analysis of the mixture of Zn and phen showed a broad absorption band from 350 nm to 460 nm, which was not observed in the spectra for either phen or [Zn(phen)_3_](OTf)_2_ alone (Fig. 1c). To understand the origin of this absorption, simulation of UV-Vis absorption spectra was carried out by TD-DFT calculations (Fig. 1d). The predicted spectra of both phen and [Zn(phen)_3_]^2+^ care characterized by signals below 350 nm (black lines, Fig. 1d right and left). On the other hand, the proposed radical species, phen˙^−^ and [Zn(phen)_3_]˙^+^, are predicted to absorb at a longer wavelength (red lines, Fig. 1d right and left). In particular, the absorption band of phen˙^−^ (Fig. 1d, left) is similar to the experimentally obtained spectra from a mixture from Zn and phen, supporting the proposed generation of radical species as key reducing agents.

**Fig. 2:**
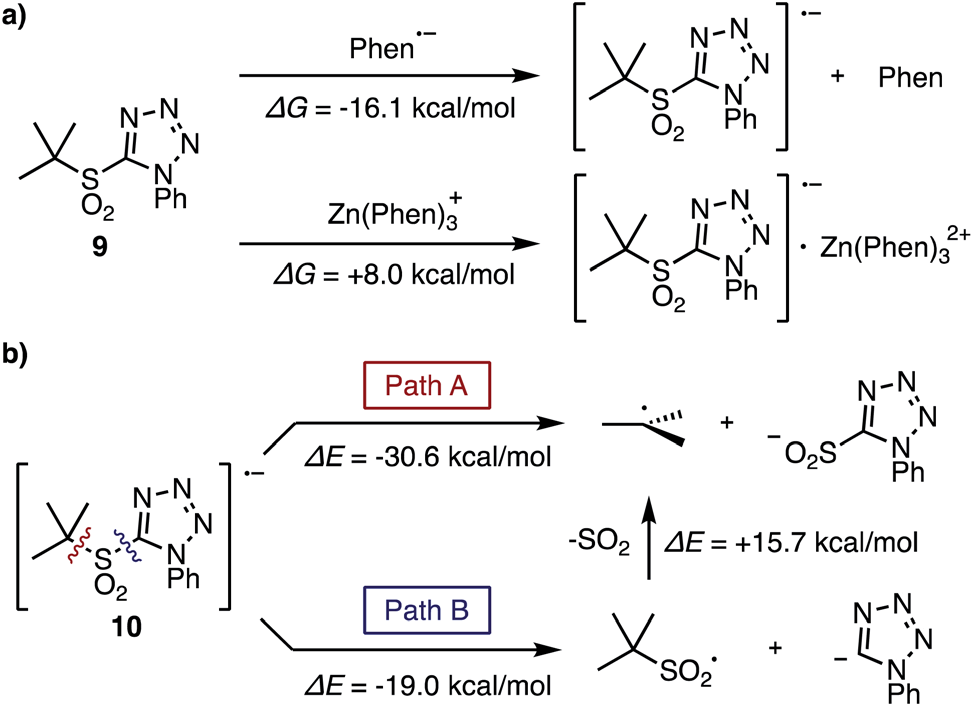
Investigation of fragmentation process of tertiary sulfone: (a) Gibbs free energies in single electron reduction of tertiary sulfone **9** by phen˙^−^ or [Zn(phen)_3_]˙^+^. (b) The bond dissociation energies for the fragmentation of sulfone radical **10**. Geometry optimizations and thermal correction were performed at CAM-B3LYP/6-31+G* level coupled with SMD (solvent = DMF). By using optimized geometries, Gibbs free energies and bond dissociation energies were computed using SCSMP2 (cc-pVDZ basis set for H, C, S, Zn, and aug-cc-pVDZ for N, O).

The fragmentation process of the tertiary sulfone was also evaluated by theoretical studies. For the first single electron reduction of *t*-butyl sulfone **9**, both phen˙^−^ and [Zn(phen)_3_]˙^+^ are possible reducing agents based on the Gibbs free energies for their generation at room temperature (Δ*G*, −16.1, +8.0 kcal mol^−1^, respectively, Fig. 2a). In addition, the Lei group reported the first reduction potential of phen in DMF to be −2.06 V.^13^ With an experimentally determined sulfone reduction potential of −1.65 V, (Scheme 2) a thermodynamically downhill reduction by phen˙^−^ is completely reasonable.

Considering the fragmentation process of sulfone radical **10**, two pathways are possible (Fig. 2b): direct formation of the requisite *t*-butyl radical along with the formation of tetrazolylsulfinic anion (Path A), or fragmentation to generate the tetrazolyl anion along with the *t*-butylsulfonyl radical, which subsequently fragments into the *t*-butyl radical and SO_2_ (Path B). The bond dissociation energies (Δ*E*) of these two pathways were calculated to be −30.6 and −19.0 kcal mol^−1^ respectively, therefore Path A should be favorable. Moreover, the addition of *in situ* generated tertiary alkylsulfonyl radical to electron-deficient olefins was reported by Wu *et al.*^14^ and Ley *et al.*,^7*c*^ but this product was never observed under our reaction conditions. Thus, Path B cannot be ruled out in this stage, but Path A is more plausible for the generation of radical species.

**Scheme 6:**
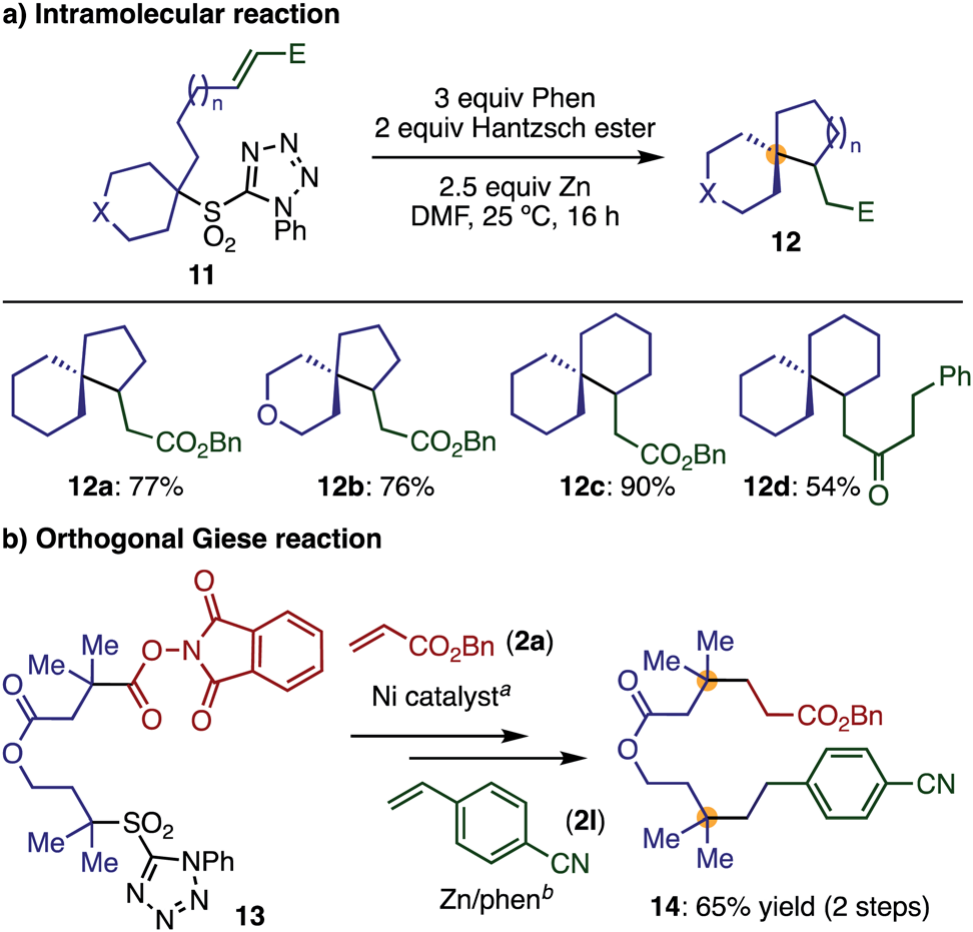
Synthetic applications. Reaction conditions: (a) **2a**, Ni(CIO_4_)_2_·6H_2_O, Zn, LiCl, MeCN, 25 °C. (b) **2l**, Hantzsch ester, phen, Zn, DMF, 25 °C.

Finally, substrates in which the olefin moieties were introduced by α-alkylation of secondary sulfones can be employed in an intramolecular Giese reaction, giving interesting spiro compounds (Scheme 6a) and demonstrating the high modularity and functional group compatibility of the transformation. A variety of spiro compounds (**12**) with different ring sizes were prepared in good to excellent yields by varying the alkyl tether lengths.

Moreover, we were interested in whether the sulfone employed in our transformation would be sufficiently differentiated from Baran's widely employed redox-active ester,^12^ enabling an orthogonal Giese reaction. As a first step, we showed that tertiary sulfone **1a** was completely inactive under Ni-catalyzed decarbonylative reaction condition as developed by Baran *et al.* for cross coupling^8*c*^ (see ESI‡). Then, we prepared compound **13**, bearing a sulfone and a redox-active ester, and reacted this species sequentially with two different electron-deficient olefins. This simple sequence of reactions afforded compound **14**, bearing two quaternary centers in reasonable yield (Scheme 6b). This transformation highlights how our method can be used complementarily with other methods, resulting in facile routes to highly functionalized products with multiple quaternary centers.

## Conclusions

We have developed a new and simple protocol for the generation of tertiary radicals through reductive desulfonylation of alkylsulfones by Zn and phen, enabling the Giese reaction to form various quaternary products. Control experiments and theoretical calculations illustrate the importance of the substituents on sulfonyl group and the nature of reducing agents to control the radical generation process. Most importantly, we have demonstrated that the sulfone, which has been regarded as an electrophile, can also be used to generate nucleophilic radicals simply by the modification of sulfonyl group. This umpolung approach will allow the expansion of the utility of organosulfones in chemical transformations using radical species. Further studies to develop new bond forming reactions through reductive activation of sulfones are currently ongoing in our laboratory.

## Conflicts of interest

The authors declare no conflicts of interest.

## Supplementary Material

SC-012-D1SC00133G-s001
