# Supplementary material for: Synthesis of quaternary centres by single electron reduction and alkylation of alkylsulfones
Source: Chem Sci. 2021 Feb 4;12(13):4866–71. doi: 10.1039/d1sc00133g (PMC8179647; doi:10.1039/d1sc00133g)
Supplement: SC-012-D1SC00133G-s001 [file SC-012-D1SC00133G-s001.pdf]

Electronic Supplementary Material (ESI) for Chemical Science.
This journal is © The Royal Society of Chemistry 2021

- Supporting Information -

Synthesis of Quaternary Centres by Single Electron Reduction and Alkylation of Alkylsulfones

Masakazu Nambo*, Yasuyo Tahara, Jacky C.-H. Yim, Daisuke Yokogawa, and Cathleen M. Crudden*

Institute of Transformative Bio-Molecules (WPI-ITbM), Nagoya University, Nagoya, Japan 464-8601
Department of Chemistry, Queen's University, 90 Bader Lane, Kingston, Ontario, Canada K7L 3N6

E-mail: mnambo@itbm.nagoya-u.ac.jp, cruddenc@chem.queensu.ca

Table of Contents

1. General S2
2. Preparation of primary and secondary sulfones S3-S4
3. Preparation of tertiary sulfones S4–S11
4. Typical Procedure for Zn/phen-mediated Giese reaction of tertiary sulfone **1** with electron-deficient olefins **2** S12-S19
5. Intermolecular Giese reaction of tertiary sulfone **11** S20-S21
6. Orthogonal Giese reaction of tertiary sulfone **13** S21-S23
7. Preparation of samples for EPR and UV-Vis measurement S23
8. Cyclic voltammetry measurement S23-S26
9. Computational methods S26
10. References S27
11. ^{1}H, ^{13}C and ^{19}F NMR spectra of products S28-S85
12. Coordinates of calculated structures S86-S94

1. General

Unless otherwise noted, all materials including dry solvents were obtained from commercial suppliers and used without further purification. Zn powder (particle size: 6~9 μm) was purchased from FUJIFILM Wako Chemicals, and was activated by 1N HCl*aq* before use.

N,N-Diphenylacrylamide (**2j**)[1], methyl 2-((phenylsulfonyl)methyl)acrylate (**2m**)[2], 1,1-dimethyl-1-(phenylsulfonyl)-3-phenylpropane (**5a**)[3], 2-((3-phenylpropylsulfonyl)benzo[d]thiazole[4], 2-((3-phenylpropylsulfonyl)pyridine[5], 1-phenyl-5-((3-phenylpropyl)sulfonyl)-1*H*-tetrazole[6], 5-(Cyclohexylsulfonyl)-1-phenyl-1*H*-tetrazole[6], 1-Phenyl-5-((tetrahydro-*2H*-pyran-4-yl)sulfonyl)-*1H*-tetrazole[6], 5-((2,3-Dihydro-*1H*-inden-2-yl)sulfonyl)-1-phenyl-*1H*-tetrazole[6], 5-((3-benzyloxypropyl)sulfonyl)-1-phenyl-*1H*-tetrazole[7], 5-((3-(*tert*-butyldimethylsilyloxy)propyl)sulfonyl)-1-phenyl-*1H*-tetrazole[8], 1-benzyloxycarbonyl-(4-(1-phenyl-1*H*-tetrazol-5-yl)sulfonyl)azetidine[9], and $Zn(phen)_3(OTf)_2$[10] were prepared according to procedures reported in the literature.

Unless otherwise noted, all reactions were performed with dry solvents under an atmosphere of argon in flame-dried glassware with standard vacuum-line techniques. All work-up and purification procedures were carried out with reagent-grade solvents in air.

Analytical thin-layer chromatography (TLC) was performed using E. Merck silica gel 60 F_{254} precoated plates (0.25 mm) visualizing with UV light (254 nm) and ethanolic phosphomolybdic acid. Preparative thin-layer chromatography (PTLC) was performed using Wakogel B5-F silica coated plates (0.75 mm) prepared in our laboratory. Preparative recycling HPLC was performed with a JAI LC-9204 instrument equipped with JAIGEL-1H/JAIGEL-2H columns using chloroform as an eluent.

High-resolution mass spectra (HRMS) were obtained from a Thermo Fisher Scientific Exactive (ESI) and a JMS-T100TD instrument (DART). Nuclear magnetic resonance (NMR) spectra were recorded on a JEOL ECA600II (^{1}H 600 MHz, ^{13}C 150 MHz), a JEOL ECA-500 (^{1}H 500 MHz, ^{13}C 125 MHz), a JEOL ECS-400 (^{1}H 400 MHz, ^{13}C 100 MHz) spectrometers. Chemical shifts for ^{1}H NMR are expressed in parts per million (ppm) relative to tetramethylsilane (δ 0.00 ppm). Chemical shifts for ^{13}C NMR spectra are expressed in ppm relative to $CDCl_3$ (δ 77.0 ppm). Data are reported as follows: chemical shift, multiplicity (s = singlet, d = doublet, t = triplet, q = quartet, quin = quintet, m = multiplet), coupling constant (Hz), and integration. Inductively coupled plasma-atomic emission spectroscopy (ICP-AES) of Zn powder was performed with a Shimadzu ICPE-9800 insturment. An analytical sample was prepared by treating Zn powder with HCl*aq*. The Electron Paramagnetic Resonance (EPR) spectrum were collected in DMF at 85 K with a JEOL JES-FA200, the central field was set to 320mT and the range of scan was 20mT. UV-vis spectra were recorded with Shimadzu UV-1800 spectrometer using 1 cm quartz cuvettes.

2. Preparation of primary and secondary sulfones

A) Preparation of 3,5-bis(trifluoromethyl)phenyl 1-(3-phenylpropyl) sulfone

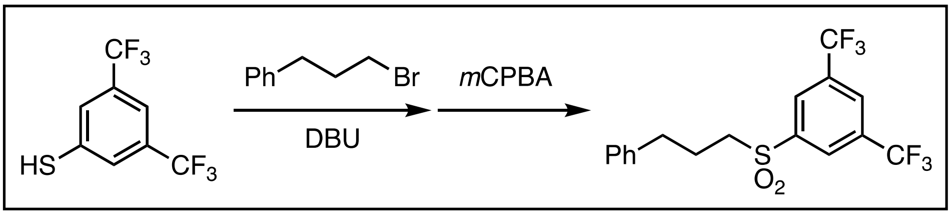

A 100-mL flask containing a magnetic stirring bar was flame-dried under vacuum and filled with argon after cooling to room temperature. To the flask were added 3,5-bis(trifluoromethyl)benzenethiol (760 μL, 5 mmol), and 3-phenylpropyl bromide (840 μL, 5.25 mmol), and dry THF (30 mL) under argon. DBU (825 μL, 5.5 mmol) was added, and the mixture was stirred at room temperature for 16 h. The mixture was quenched with sat. NH_4Cl*aq* and extracted with EtOAc (3 times). The combined extracts were dried over $NaSO_4$, filtered and evaporated under reduced pressure. The residue was dissolved in CH_2Cl_2 (5 mL). *m*CPBA (>77%, 2.8 g, 12.5 mmol) in CH_2Cl_2 (15 mL) was slowly added to this solution at 0 ºC and the mixture was stirred at room temperature for 12 h. The mixture was quenched with sat. Na_2SO_3 solution (~3 mL) and was washed with sat. $NaHCO_3$*aq* (3 times). The organic layer was dried over Na_2SO_4, filtered and evaporated under reduced pressure. The residue was purified by column chromatography (EtOAc/hexane = 1:20) to afford 3,5-bis(trifluoromethyl)phenyl 1-(3-phenylpropyl) sulfone as a white solid (1.77 g, 89% yield, 2 steps). ^{1}H NMR (600 MHz, $CDCl_3$) δ 2.09-2.14 (m, 2H), 2.75 (t, *J* = 7.2 Hz, 2H), 3.12-3.14 (m, 2H), 7.10 (d, *J* = 7.2 Hz, 2H), 7.22 (t, *J* = 7.2 Hz, 1H), 7.28 (t, *J* = 7.2 Hz, 2H), 8.15 (s, 1H), 8.33 (s, 2H). ^{13}C NMR (150 MHz, $CDCl_3$) δ 23.8, 33.8, 55.2, 122.3 (q, *J* = 272 Hz), 126.7, 127.4 (t, *J* = 4.2 Hz), 128.3, 128.5, 128.7, 133.3 (q, *J* = 35 Hz), 139.2, 142.0. ^{19}F NMR (376 MHz, $CDCl_3$) δ -62.8. IR (ATR): 3066, 2945, 1559, 1457, 1284, 753 cm^{-1}. HRMS (ESI) *m/z* calcd for $C_{17}H_{13}O_2F_6S$ $[M-H]^-$: 395.0535, found 395.0544.

B) Preparation of 5-((4-phenylbutan-2-yl)sulfonyl)-1-phenyl-1*H*-tetrazole

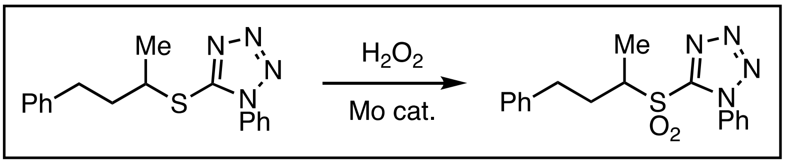

To a 100-mL two-neck flask containing a magnetic stirring bar were added 1-phenyl-5-(4-phenylbutan-2-ylthio)-1*H*-tetrazole (1.93 g, 6.4 mmol), EtOH (15 mL), and ammonium molybdate tetrahydrate (207 mg, 0.17 mmol). 30% H_2O_2 in H_2O (6.2 ml, 30 mmol) was slowly added to this solution at 0 ºC and the mixture was stirred at room temperature for 16 h. The mixture was quenched with sat. Na_2SO_3 solution at 0 ºC, and was extracted with CH_2Cl_2. (3 times). The organic layer was dried over $Na_{24}SO_4$, filtered and evaporated under reduced pressure. The residue was purified by column chromatography (EtOAc/hexane = 1:8 to 1:6) to afford 5-((4-phenylbutan-2-yl)sulfonyl)-1-phenyl-1*H*-tetrazole as a pale yellow oil (1.83 g, 71% yield). ^{1}H NMR (600 MHz, $CDCl_3$) δ 1.53 (d, *J* = 6.6 Hz, 3H), 1.96-2.02 (m, 1H), 2.41-2.47 (m, 1H), 2.69-2.74 (m, 1H), 2.88-2.92 (m, 1H), 3.77-3.81 (m, 1H), 7.17 (d, *J* = 7.2 Hz, 2H), 7.23 (t, *J* = 7.2 Hz, 1H), 7.30 (t, *J* = 7.2 Hz, 2H), 7.55-7.62

(m, 5H). ^{13}C NMR (150 MHz, $CDCl_3$) δ 12.7, 30.1, 32.2, 60.5, 125.4, 126.6, 128.3, 128.7, 129.5, 131.4, 133.0, 139.5, 152.7. IR (ATR): 3026, 2944, 1559, 1453, 1332, 1148, 713 cm^{-1}. HRMS (ESI) *m/z* calcd for $C_{17}H_{19}N_4O_2S$ [M+Na]$^+$: 343.1223, found 343.1225.

3. Preparation of tertiary sulfones

A) Typical procedure for preparation of tertiary sulfones from primary sulfones

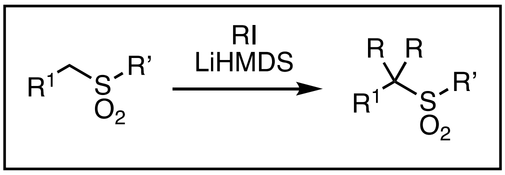

A two-neck Schlenk flask containing a magnetic stirring bar was flame-dried under vacuum and filled with argon after cooling to room temperature. To the flask were added primary sulfone (1 equiv) and dry THF (5 mL per mmol of sulfone). A solution of LiHMDS (1.3 M in THF, 3 equiv) was added drop wise to the reaction mixture at -78 °C under argon. After stirring for 30 min, alkyl iodide (4 equiv) was added, the mixture was stirred at rt for 16 h. Sat. NH_4Cl*aq* was added to the reaction mixture, and the layers were separated. The aqueous layer was extracted with EtOAc (3 times), and the combined organic layer was washed with sat. $NaHCO_3$*aq* and brine. The organic layer was dried over Na_2SO_4, filtered and the solvent was evaporated under reduced pressure. The crude product was purified by column chromatography to afford the corresponding tertiary sulfone.

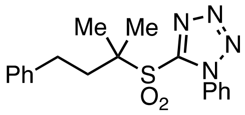

5-((1',1'-Dimethyl-3'-phenylpropyl)sulfonyl)-1-phenyl-*1H*-tetrazole 1a

Purification by column chromatography (DCM/hexane = 1:2).

2.10 g, 84% isolated yield (7 mmol scale); white solid.

^{1}H NMR (600 MHz, $CDCl_3$) δ 1.59 (s, 6H), 2.15-2.18 (m, 2H), 2.69-2.72 (m, 2H), 7.14 (d, *J* = 7.2 Hz, 2H), 7.21 (t, *J* = 7.2 Hz, 1H), 7.29 (t, *J* = 7.2 Hz, 2H), 7.53-7.56 (m, 4H), 7.60-7.62 (m, 1H). ^{13}C NMR (150 MHz, $CDCl_3$) δ 20.4, 30.0, 36.8, 67.7, 126.2, 126.4, 128.3, 128.6, 129.3, 131.4, 133.3, 140.3, 152.0. IR (ATR): 3067, 2872, 1495, 1461, 1335, 1169, 1113, 767 cm^{-1}. HRMS (ESI) *m/z* calcd for $C_{18}H_{20}N_4O_2SNa$ [M+Na]$^+$: 379.1199, found 379.1199.

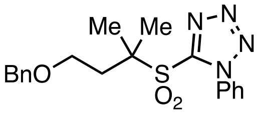

5-((3'-Benzyloxy-1',1'-dimethylpropyl)sulfonyl)-1-phenyl-*1H*-tetrazole 1d

Purification by column chromatography (EtOAc/hexane = 1:10).

614 mg, 82% isolated yield (1.9 mmol scale); white solid

^{1}H NMR (600 MHz, $CDCl_3$) δ 1.56 (s, 6H), 2.26 (t, *J* = 6.0 Hz, 2H), 3.64 (t, *J* = 6.0 Hz, 2H), 4.45 (s, 2H), 7.27-7.30 (m, 3H), 7.34 (t, *J* = 7.2 Hz, 2H), 7.54-7.63 (m, 5H). ^{13}C NMR (150 MHz, $CDCl_3$) δ 20.7, 34.2, 65.6, 67.3, 73.1, 126.1, 127.6, 127.7, 128.4, 129.3, 131.4, 133.4, 137.7, 151.9. IR (ATR): 3028, 2870, 1495, 1454, 1335, 1115, 760 cm^{-1}. HRMS (ESI) *m/z* calcd for $C_{19}H_{22}N_4O_3SNa$ [M+Na]$^+$: 409.1305, found 409.1305.

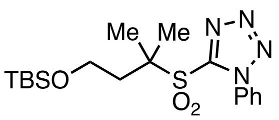

5-((3’-*tert*-Butyldimethylsilyl)oxy-1’,1’-dimethylpropyl)sulfonyl)-1-phenyl-*1H*-tetrazole 1e

Purification by column chromatography (EtOAc/hexane = 1:10).

3.08 g, 85% isolated yield (8.8 mmol scale); white solid.

^{1}H NMR (600 MHz, $CDCl_3$) δ 0.04 (s, 6H), 0.87 (s, 9H), 1.57 (s, 6H), 2.14 (t, *J* = 6.0 Hz, 2H), 3.80 (t, *J* = 6.0 Hz, 2H), 7.57-7.63 (m, 5H). ^{13}C NMR (150 MHz, $CDCl_3$) δ -5.5, 18.1, 20.6, 25.8, 36.6, 58.7, 67.5, 126.2, 129.3, 131.4, 133.4, 151.9. IR (ATR): 3070, 2958, 1498, 1462, 1339, 1255, 1094, 839 cm^{-1}. HRMS (ESI) *m/z* calcd for $C_{18}H_{30}N_4O_3SiSNa$ $[M+Na]^+$: 433.1700, found 433.1698.

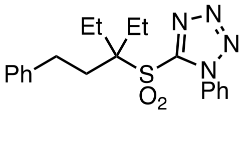

5-((1’,1’-Diethyl-3’-phenylpropyl)sulfonyl)-1-phenyl-*1H*-tetrazole 1h

Purification by column chromatography (DCM/hexane = 1:1).

297 mg, 77% isolated yield (1 mmol scale); white solid

^{1}H NMR (600 MHz, $CDCl_3$) δ 1.59 (s, 6H), 2.03 (q, *J* = 7.2 Hz, 4H), 2.15-2.18 (m, 2H), 2.69-2.72 (m, 2H), 7.14 (d, *J* = 7.2 Hz, 2H), 7.21 (t, *J* = 7.2 Hz, 1H), 7.29 (t, *J* = 7.2 Hz, 2H), 7.53-7.56 (m, 4H), 7.60-7.62 (m, 1H). ^{13}C NMR (150 MHz, $CDCl_3$) δ 8.2, 25.0, 29.9, 34.0, 74.0, 126.3, 126.4, 128.2, 128.6, 129.2, 131.4, 133.4, 140.9, 152.7. IR (ATR): 3067, 2872, 1495, 1461, 1335, 1169, 1113, 767 cm^{-1}. HRMS (ESI) *m/z* calcd for $C_{20}H_{24}N_4O_2SNa$ $[M+Na]^+$: 407.1512, found 407.1508.

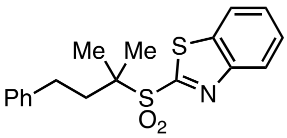

2-((1,1-Dimethyl-3-phenylpropyl)sulfonyl)benzothiazole 4a

Purification by column chromatography (EtOAc/hexane = 1:10).

407 mg, 59% isolated yield (2 mmol scale); white solid.

^{1}H NMR (600 MHz, $CDCl_3$) δ 1.60 (s, 6H), 2.18-2.21 (m, 2H), 2.76-2.79 (m, 2H), 7.18-7.20 (m, 3H), 7.28 (t, *J* = 7.8 Hz, 2H), 7.59 (t, *J* = 8.4 Hz, 1H), 7.63 (t, *J* = 8.4 Hz, 1H), 8.01 (d, *J* = 8.4 Hz, 1H), 8.26 (d, *J* = 8.4 Hz, 1H). ^{13}C NMR (150 MHz, $CDCl_3$) δ 21.1, 30.2, 37.3, 65.2, 122.1, 125.7, 126.2, 127.5, 127.9, 128.3, 128.5, 137.3, 140.9, 153.1, 164.0. IR (ATR): 3062, 2939, 1499, 1469, 1314, 1116, 763 cm^{-1}. HRMS (ESI) *m/z* calcd for $C_{18}H_{19}NO_2S_2Na$ $[M+Na]^+$: 368.0749, found 368.0749.

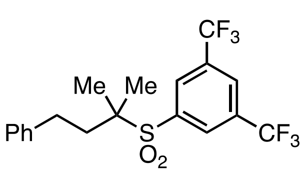

3,5-Bis(trifluoromethyl)phenyl 1-(1,1-dimethyl-3-phenylpropyl) sulfone 6a

Purification by column chromatography (EtOAc/hexane = 1:20).

632 mg, 75% isolated yield (2 mmol scale); white solid.

^{1}H NMR (600 MHz, $CDCl_3$) δ 1.41 (s, 6H), 2.01-2.04 (m, 2H), 2.73-2.76 (m, 2H), 7.15 (d, *J* = 7.8 Hz, 2H), 7.21 (t, *J* = 7.8 Hz, 1H), 7.29 (t, *J* = 7.8 Hz, 2H), 8.16 (s, 1H), 8.34 (s, 2H). ^{13}C NMR (150 MHz, $CDCl_3$) δ 21.1, 30.2, 37.0, 63.9, 122.3 (q, *J* = 272 Hz), 126.4, 127.3 (t, *J* = 17 Hz), 128.1, 128.6, 130.7, 132.8 (q, *J* = 35 Hz), 138.7, 140.5. ^{19}F NMR (376 MHz, $CDCl_3$) δ -62.8. IR (ATR): 3062, 2978, 1455, 1278, 1127, 1095, 909 cm^{-1}. HRMS (DART) *m/z* calcd for $C_{11}H_{15}$ $[M-C_8H_3SO_2F_6]^+$: 147.1174, found 147.1171.

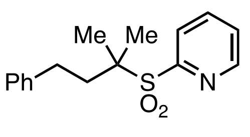

2-((1,1-Dimethyl-3-phenylpropyl)sulfonyl)pyridine 7a

Purification by column chromatography (EtOAc/hexane = 1:5).

568 mg, 98% isolated yield (2 mmol scale); colorless oil.

^{1}H NMR (600 MHz, $CDCl_3$) δ 1.48 (s, 6H), 2.07-2.10 (m, 2H), 2.71-2.74 (m, 2H), 7.16 (d, J = 7.2 Hz, 2H), 7.19 (d, J = 7.2 Hz, 1H), 7.27 (d, J = 7.2 Hz, 2H), 7.55 (dd, J = 8.4, 4.8 Hz, 1H), 7.95 (t, J = 8.4 Hz, 1H), 8.10 (d, J = 8.4 Hz, 1H), 8.80 (d, J = 4.8 Hz, 1H). ^{13}C NMR (150 MHz, $CDCl_3$) δ 21.1, 30.2, 37.5, 63.6, 125.4, 126.1, 127.2, 128.3, 128.4, 137.7, 141.2, 150.1, 155.4. IR (ATR): 3057, 2942, 1577, 1543, 1425, 1301, 1099, 745 cm^{-1}. HRMS (ESI) *m/z* calcd for $C_{16}H_{19}NO_2SNa$ [M+Na]$^+$: 312.1029, found 312.1028.

B) Typical procedure for preparation of tertiary sulfones from secondary sulfones

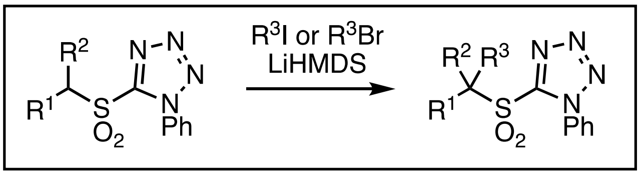

A two-neck flask containing a magnetic stirring bar was flame-dried under vacuum and filled with argon after cooling to room temperature. To the flask were added primary sulfone (1 equiv) and dry THF (5 mL per mmol of sulfone). A solution of LiHMDS (1.3 M in THF, 1.5 equiv) was added drop wise to the reaction mixture at -78 °C under argon. After stirring for 30 min, alkyl bromide or iodide (2 equiv) was added, the mixture was stirred at rt for 16 h. Sat. NH_4Cl*aq* was added to the reaction mixture, and the layers were separated. The aqueous layer was extracted with EtOAc (3 times), and the combined organic layer was washed with sat. $NaHCO_3$*aq* and brine. The organic layer was dried over Na_2SO_4, filtered and the solvent was evaporated under reduced pressure. The crude product was purified by column chromatography or GPC to afford the corresponding tertiary sulfone.

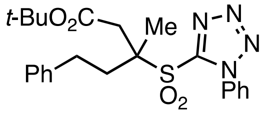

5-((1'-(*tert*-butoxycarbonylmethyl)-1'-methyl-3'-phenylpropyl)sulfonyl)-1-phenyl-*1H*-tetrazole 1b

Purification by GPC.

268 mg, 58% isolated yield (1 mmol scale); yellow oil.

^{1}H NMR (600 MHz, $CDCl_3$) δ 1.44 (s, 9H), 1.63 (s, 3H), 2.24 (ddd, J = 14.4, 12.6, 6.4 Hz, 1H), 2.39 (ddd, J = 14.4, 12.6, 6.4 Hz, 1H), 2.73-2.83 (m, 2H), 3.05 (d, J = 15.0 Hz, 1H), 3.11 (d, J = 15.0 Hz, 1H), 7.13 (d, J = 7.8 Hz, 2H), 7.19 (t, J = 7.8 Hz, 1H), 7.27 (t, J = 7.8 Hz, 1H), 7.53-7.56 (m, 4H), 7.60-7.62 (m, 1H). ^{13}C NMR (150 MHz, $CDCl_3$) δ 19.7, 28.0, 30.4, 35.4, 38.2, 69.3, 82.2, 126.3, 128.3, 128.5, 129.2, 131.4, 133.3, 140.6, 152.2, 167.8. IR (ATR): 3001, 2979, 1726, 1498, 1458, 1340, 1145, 761 cm^{-1}. HRMS (ESI) *m/z* calcd for $C_{20}H_{23}N_4O_2ClSNa$ [M+Na]$^+$: 411.1122, found 411.1122.

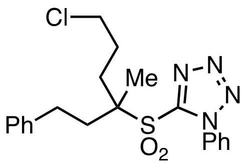

5-((1'-(4-Chloropropyl)-1'-methyl-3'-phenylpropyl)sulfonyl)-1-phenyl-1*H*-tetrazole 1c

Purification by GPC and column chromatography (EtOAc/hexane = 1:12).

301 mg, 72% isolated yield (1 mmol scale); white solid

^{1}H NMR (500 MHz, $CDCl_3$) δ 1.54 (s, 3H), 1.90-2.03 (m, 2H), 2.07-2.24 (m, 4H), 2.68-2.78 (m, 2H), 3.50-3.57 (m, 2H), 7.14 (d, *J* = 7.5 Hz, 2H), 7.22 (t, *J* = 7.5 Hz, 1H), 7.29 (t, *J* = 7.5 Hz, 2H), 7.52-7.57 (m, 4H), 7.61-7.64 (m, 1H). ^{13}C NMR (125 MHz, $CDCl_3$) δ 19.8, 26.9, 29.9, 30.8, 35.1, 44.6, 70.1, 126.3, 126.5, 128.2, 128.7, 129.3, 131.5, 133.3, 140.3, 152.1. IR (ATR): 3028, 2952, 1496, 1455, 1321, 1141, 761, 732 cm^{-1}. HRMS (ESI) *m/z* calcd for $C_{20}H_{23}N_4O_2ClSNa$ $[M+Na]^+$: 441.1122, found 411.1122.

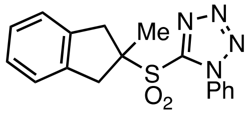

5-((2',3'-Dihydro-2'-methyl-1*H*-inden-2'-yl)sulfonyl)-1-phenyl-1*H*-tetrazole 1i

Purification by column chromatography (EtOAc/hexane = 1:20).

139 mg, 41% isolated yield (1 mmol scale); white solid

^{1}H NMR (600 MHz, $CDCl_3$) δ 1.77 (s, 3H), 3.17 (d, *J* = 16.2 Hz, 2H), 3.94 (d, *J* = 16.2 Hz, 2H), 7.21 (s, 4H), 7.59-7.65 (m, 5H). ^{13}C NMR (150 MHz, $CDCl_3$) δ 22.5, 41.2, 72.1, 124.8, 125.8, 127.6, 129.5, 131.5, 133.2, 138.1, 152.2. IR (ATR): 3075, 2917, 1496, 1443, 1332, 1155, 1104, 764cm^{-1}. HRMS (ESI) *m/z* calcd for $C_{17}H_{16}N_4O_2SNa$ $[M+Na]^+$: 363.0886, found 363.0885.

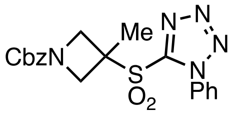

1-Benzyloxycarbonyl-3-methyl-3-((1-phenyl-1*H*-tetrazol-5-yl)sulfonyl)azetidine 1j

Purification by column chromatography (EtOAc/hexane = 1:10).

345 mg, 84% isolated yield (1 mmol scale); colorless oil

^{1}H NMR (600 MHz, $CDCl_3$) δ 1.95 (s, 3H), 4.09 (d, *J* = 10.2 Hz, 2H), 4.71 (d, *J* = 10.2 Hz, 2H), 5.12 (s, 2H), 7.31-7.37 (m, 5H), 7.60-7.69 (m, 5H). ^{13}C NMR (150 MHz, $CDCl_3$) δ 20.5, 55.4, 56.2, 60.0, 67.3, 125.1, 128.1, 128.3, 128.5, 129.7, 131.6, 132.8, 135.9, 151.1, 155.9. IR (ATR): 3071, 2952, 1712, 1498, 1415, 1338, 1144, 1097, 763 cm^{-1}. HRMS (ESI) *m/z* calcd for $C_{19}H_{19}N_5O_4SNa$ $[M+Na]^+$: 436.1050, found 436.1045.

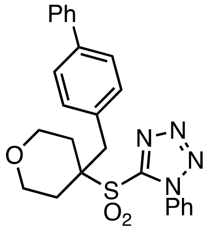

4-(4'-Biphenylmethyl)-4-((1'-phenyl-1*H*-tetrazol-5'-yl)sulfonyl)tetrahydropyran 1k

Purification by column chromatography (EtOAc/hexane = 1:5).

394 mg, 86% isolated yield (1 mmol scale); white solid.

^{1}H NMR (400 MHz, $CDCl_3$) δ 2.08-2.14 (m, 2H), 2.48-2.54 (m, 2H), 3.32 (s, 2H), 3.69-3.74 (m, 2H), 4.01-4.06 (m, 2H), 7.18 (d, *J* = 8.0 Hz, 2H), 7.34-7.38 (m, 1H), 7.45 (t, *J* = 8.0 Hz, 2H), 7.48 (d, *J* = 8.0 Hz, 2H), 7.55-7.67 (m, 7H). ^{13}C NMR (150 MHz, $CDCl_3$) δ 28.0, 38.5, 63.0, 68.9, 126.1, 127.0, 127.1, 127.5, 128.8, 129.4, 131.50, 131.51, 132.3, 133.3, 140.3, 140.7, 152.3. IR (ATR): 3075, 2944, 2354, 1457, 1149, 758 cm^{-1}. HRMS (ESI) *m/z* calcd for $C_{25}H_{24}N_4O_3SNa$ $[M+Na]^+$: 483.1461, found 483.1459.

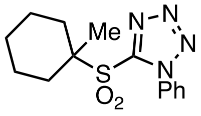

5-(1'-Methylcyclohexylsulfonyl)-1-phenyl-1*H*-tetrazole 1l

Purification by column chromatography (DCM/hexane = 1:1).

283 mg, 92% isolated yield (1 mmol scale); white solid.

^{1}H NMR (600 MHz, $CDCl_3$) δ 1.22 (qt, *J* = 13.2, 3.6 Hz, 1H), 1.44 (qt, *J* = 13.2, 3.6 Hz, 2H), 1.56 (s, 3H), 1.69-1.71 (m, 1H), 1.78 (dt, *J* = 14.4, 3.6 Hz, 2H), 1.88-1.90 (m, 2H), 1.97 (td, *J* = 13.2, 3.6 Hz, 2H), 7.56-7.64 (m, 5H). ^{13}C NMR (150 MHz, $CDCl_3$) δ 16.9,

21.2, 24.8, 29.2, 68.3, 126.2, 129.3, 131.4, 133.5, 151.9. IR (ATR): 3088, 2936, 1498, 1465, 1335, 1150, 1094, 757 cm^{-1}. HRMS (ESI) *m/z* calcd for $C_{14}H_{18}N_4O_2SNa$ $[M+Na]^+$: 329.1043, found 329.1042.

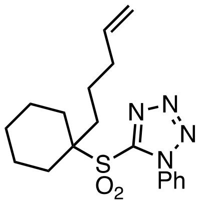

5-(1’-(4-Pentenyl)cyclohexylsulfonyl)-1-phenyl-1*H*-tetrazole

Purification by column chromatography (EtOAc/hexane = 1:20).

104 mg, 29% isolated yield (1 mmol scale); pale yellow solid.

^{1}H NMR (500 MHz, $CDCl_3$) δ 1.20-1.26 (m, 1H), 1.36-141 (m, 2H), 1.50-1.56 (m, 3H), 1.66 (dt, *J* = 13.5, 3.5 Hz, 1H), 1.75 (dt, *J* = 13.5, 3.5 Hz, 2H), 1.90-1.94 (m, 6H), 1.99 (q, *J* = 14.0, 2.5 Hz, 2H), 4.96-5.01 (m, 2H), 5.67-5.75 (m, 1H), 7.53-7.64 (m, 5H). ^{13}C NMR (150 MHz, $CDCl_3$) δ 21.2, 22.4, 24.4, 28.1, 29.1, 33.9, 70.7, 115.4, 126.4, 129.2, 131.4, 133.5, 137.6, 152.1. IR (ATR): 3080, 2943, 1647, 1495, 1461, 1332, 1143, 1015, 771 cm^{-1}. HRMS (ESI) *m/z* calcd for $C_{18}H_{24}N_4O_2SNa$ $[M+Na]^+$: 383.1512, found 383.1512.

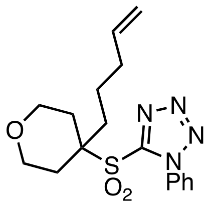

4-(1’-(4-Pentenyl)-4-(1’-phenyl-1*H*-tetrazol-5’-yl)sulfonyl)tetrahydropyran

Purification by column chromatography (DCM/hexane = 1:1).

347 mg, 46% isolated yield (2.1 mmol scale); white solid.

^{1}H NMR (600 MHz, $CDCl_3$) δ 1.48-1.54 (m, 2H), 1.85 (d, *J* = 13.2 Hz, 2H), 1.99-2.03 (m, 4H), 2.29-2.34 (m, 2H), 3.52-3.56 (m, 2H), 3.94 (dt, *J* = 11.4, 4.2 Hz, 2H), 4.97-5.00 (m, 2H), 5.66-5.72 (m, 1H), 7.56-7.65 (m, 5H). ^{13}C NMR (150 MHz, $CDCl_3$) δ 22.3, 28.1, 29.4, 33.6, 62.8, 67.9, 115.7, 126.2, 129.2, 131.4, 133.2, 137.2, 151.6. IR (ATR): 2970, 2860, 1495, 1344, 1135, 1099, 762 cm^{-1}. HRMS (ESI) *m/z* calcd for $C_{17}H_{22}N_4O_3SNa$ $[M+Na]^+$: 385.1305, found 385.1305.

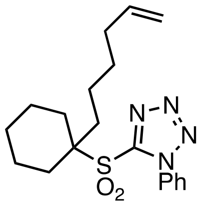

5-(1’-(5-Hexenyl)cyclohexylsulfonyl)-1-phenyl-1*H*-tetrazole

Purification by column chromatography (EtOAc/hexane = 1:30).

85 mg, 23% isolated yield (1 mmol scale); colorless oil

^{1}H NMR (500 MHz, $CDCl_3$) δ 1.17-1.26 (m, 1H), 1.28-1.34 (m, 1H), 1.35-1.46 (m, 3H), 1.65 (dt, *J* = 13.0, 3.5 Hz, 1H), 1.75 (dt, *J* = 14.0, 3.5 Hz, 2H), 1.89-1.94 (m, 6H), 1.99-2.03 (m, 2H), 4.92-5.00 (m, 2H), 5.71-5.80 (m, 1H), 7.54-7.65 (m, 5H). ^{13}C NMR (150 MHz, $CDCl_3$) δ 21.3, 22.7, 24.4, 28.1, 29.3, 29.7, 33.3, 70.8, 114.7, 126.4, 129.2, 131.4, 133.5, 138.4, 152.1. IR (ATR): 3083, 3062, 2947, 1653, 1559, 1458, 1015, 707 cm^{-1}. HRMS (ESI) *m/z* calcd for $C_{19}H_{26}N_4O_2SNa$ $[M+Na]^+$: 397.1669, found 397.1668.

C) Preparation of 5-((3’-(4’-bromophenyl)oxy-1’,1’-dimethylpropyl)sulfonyl)-1-phenyl-*1H*-tetrazole 1f

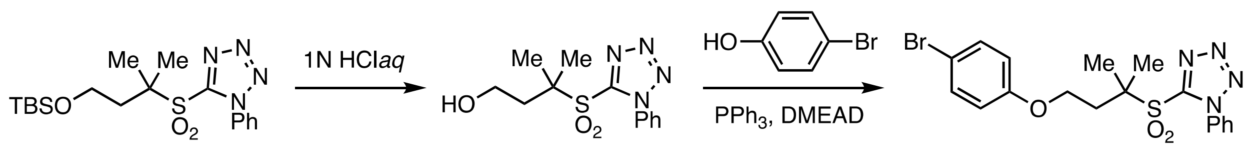

To a 100 mL flask were added 5-((3'-*tert*-butyldimethylsilyl)oxy-1',1'-dimethylpropyl)sulfonyl)-1-phenyl-*1H*-tetrazole **1f** (1.80 g, 4.4 mmol) and THF (21 mL) and H_2O (10.5 mL). 1N HCl*aq* (5.1 mL) was added at room temperature. After stirring at room temperature for 4 h, brine was added and this mixture was extracted with EtOAc. (3 times). The organic layer was dried over Na_2SO_4, filtered and the solvent was evaporated under reduced pressure. The crude material was purified by column chromatography (EtOAc/hexane = 1:2) to give 5-((1',1'-dimethyl-3'-hydroxypropyl)sulfonyl)-1-phenyl-*1H*-tetrazole (1.23 g, 95%) as a white solid.

^{1}H NMR (600 MHz, $CDCl_3$) δ 1.59 (s, 6H), 2.22 (t, *J* = 6.6 Hz, 2H), 3.85 (q, *J* = 6.6 Hz, 2H), 7.58-7.64 (m, 5H). ^{13}C NMR (150 MHz, $CDCl_3$) δ 21.1, 37.4, 58.3, 67.2, 126.1, 129.3, 131.5, 133.4, 151.9. IR (ATR): 3613, 3023, 2941, 1498, 1117, 1046, 763 cm^{-1}. HRMS (ESI) *m/z* calcd for $C_{12}H_{16}N_4O_3SNa$ [M+Na]$^+$: 319.0835, found 319.0835.

To a 20-mL flask were added 5-((1',1'-dimethyl-3'-hydroxypropyl)sulfonyl)-1-phenyl-*1H*-tetrazole (118 mg, 0.4 mmol), PPh_3 (126 mg, 0.48 mmol), 4-bromophenol (83 mg, 0.48 mmol) and dry THF (2 mL). under a stream of argon. Bis(2-methoxyethyl) azodicarboxylate (112 mg, 0.48 mmol) was slowly added to this mixture at 0 ºC. After stirring at room temperature for 24 h, organic solvent was evaporated under reduced pressure. The crude material was purified by column chromatography (EtOAc/hexane = 1:10) to give 5-((3'-(4'-bromophenyl)oxy-1',1'-dimethylpropyl)sulfonyl)-1-phenyl-*1H*-tetrazole **1f** (145 mg, 80%) as a white solid.

^{1}H NMR (600 MHz, $CDCl_3$) δ 1.62 (s, 6H), 2.47 (t, *J* = 6.0 Hz, 2H), 4.12 (t, *J* = 6.0 Hz, 2H), 6.73 (d, *J* = 7.2 Hz, 2H), 7.37 (d, *J* = 7.2 Hz, 2H), 7.58-7.64 (m, 5H). ^{13}C NMR (150 MHz, $CDCl_3$) δ 21.0, 34.2, 63.6, 67.0, 113.4, 116.2, 126.1, 129.4, 131.5, 132.4, 133.3, 151.8, 157.2. IR (ATR): 3064, 2901, 1488, 1338, 1243, 1172, 1117, 764 cm^{-1}. HRMS (ESI) *m/z* calcd for $C_{18}H_{19}N_4O_3SBrNa$ [M+Na]$^+$: 473.0253, found 473.0253.

D) Preparation of 5-(1',1'-Dimethyl-3'-(4-(4',4',5',5'-tetramethyl-1',3',2'-dioxaborolan-2'-yl)benzoyl)oxypropyl)sulfonyl)-1-phenyl-*1H*-tetrazole 1g

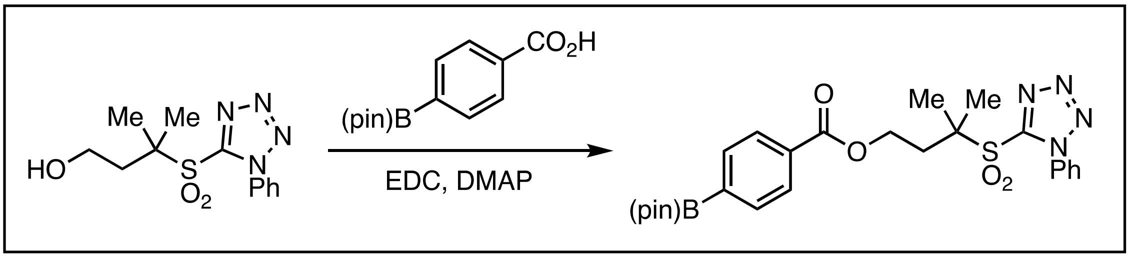

To a flask were added 5-((1',1'-dimethyl-3'-hydroxypropyl)sulfonyl)-1-phenyl-*1H*-tetrazole (119 mg, 0.4 mmol), 4-(4,4,5,5-tetramethyl-1,3,2-dioxaborolan-2-yl)benzoic acid (119 mg, 0.48 mmol), 4-dimethylaminopyridine (68.4 mg, 0.56 mmol) and dry CH_2Cl_2 (5 mL) under a stream of argon. 1-(3-Dimethylaminopropyl)-3-ethylcarbodiimide (99 μL, 0.56 mmol) was added to this mixture at room temperature. After stirring at room temperature for 16 h, sat. NH_4Cl*aq* was added to the reaction mixture, and the layers were separated. The aqueous layer was extracted with EtOAc (3 times), and the combined organic layer was dried over Na_2SO_4, filtered and the solvent was evaporated under reduced pressure. The crude

material was purified by GPC to give 5-(1',1'-Dimethyl-3'-(4-(4',4',5',5'-tetramethyl-1',3',2'-dioxaborolan-2'-yl)benzoyl)oxypropyl)sulfonyl)-1-phenyl-*1H*-tetrazole **1g** (148 mg, 70%) as a beige solid.

^{1}H NMR (600 MHz, $CDCl_3$) δ 1.36 (s, 12H), 1.62 (s, 6H), 2.47 (t, *J* = 7.2 Hz, 2H), 4.50 (t, *J* = 7.2 Hz, 2H), 7.56-7.63 (m, 5H), 7.88 (d, *J* = 8.4 Hz, 2H), 7.97 (d, *J* = 8.4 Hz, 2H). ^{13}C NMR (150 MHz, $CDCl_3$) δ 20.7, 24.8, 33.5, 60.2, 66.6, 84.1, 126.0, 128.5, 129.3, 131.4, 131.7, 133.2, 134.7, 151.7, 166.1. IR (ATR): 3085, 2980, 1718, 1507, 1399, 1119, 761cm^{-1}. HRMS (DART) *m/z* calcd for $C_{25}H_{32}BN_4O_6S$ [M+H]$^+$: 527.2146, found 527.2148.

E) Preparation of 12 for intermolecular Giese reaction

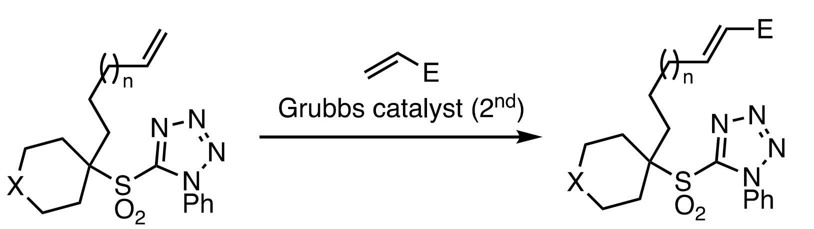

11

A 20-mL two-neck flask containing a magnetic stirring bar was flame-dried under vacuum and filled with argon after cooling to room temperature. To this flask were added sulfone (1.0 equiv), DCM (7.5 mL per mmol), olefin (5 equiv), and Grubbs catalyst 2nd generation (5 mol%) under a stream of argon. This mixture was heated at 40 ºC for 12 h. After cooling to room temperature, the mixture was passed through a pad of silica gel with copious washings with EtOAc (~10 mL). The filtrate was concentrated under reduced pressure. The crude product was purified by PTLC to afford the corresponding sulfone **11**.

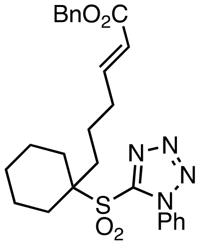

Benzyl 6-(1-(1'-phenyl-1*H*-tetrazol-5'-yl)sulfonylcyclohexyl)-2-hexanoate 11a

Purification by column chromatography (EtOAc/hexane = 1:5).

122 mg, 91% isolated yield (0.27 mmol scale); white solid.

^{1}H NMR (500 MHz, $CDCl_3$) δ 1.16-1.41 (m, 3H), 1.62-1.68 (m, 3H), 1.75 (dt, *J* = 13.5, 3.5 Hz, 2H), 1.90-1.97 (m, 6H), 2.13-2.17 (m, 2H), 5.18 (s, 2H), 5.85 (d, *J* = 15.5 Hz, 1H), 6.92 (dt, *J* = 15.5, 6.5 Hz, 1H), 7.32-7.38 (m, 5H), 7.53-7.64 (m, 5H). ^{13}C NMR (125 MHz, $CDCl_3$) δ 21.2, 21.7, 24.3, 28.0, 29.2, 32.3, 66.1, 70.6, 121.8, 126.3, 128.2, 128.5, 129.3, 131.4, 133.4, 136.0, 148.3, 151.9, 166.1. (1 aryl carbon signal is obscured). IR (ATR): 3100, 3060, 2937, 1712, 1495, 1461, 1334, 1145, 760 cm^{-1}. HRMS (ESI) *m/z* calcd for $C_{26}H_{30}N_4O_4SNa$ [M+Na]$^+$: 517.1880, found 517.1880.

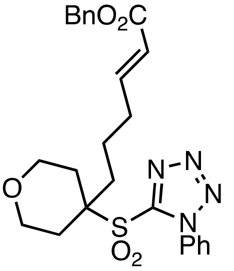

Benzyl 6-(4-(1'-phenyl-1*H*-tetrazol-5'-yl)sulfonyltetrahydro-2*H*-pyran)-2-hexanoate 11b

Purification by column chromatography (EtOAc/hexane = 1:5).

161 mg, 81% isolated yield (0.4 mmol scale); beige solid.

^{1}H NMR (600 MHz, $CDCl_3$) δ 1.63 (quin, J = 7.8 Hz, 2H), 1.83 (d, J = 13.8 Hz, 2H), 2.05-2.08 (m, 2H), 2.17 (q, J = 7.2 Hz, 2H), 2.28-2.33 (m, 2H), 3.49-3.53 (m, 2H), 3.94 (dt, J = 12.0, 4.2 Hz, 2H), 5.17 (s, 2H), 5.85 (d, J = 15.6 Hz, 1H), 6.89 (dt, J = 15.6, 7.2 Hz, 1H), 7.31-7.37 (m, 5H), 7.55-7.63 (m, 5H). ^{13}C NMR (150 MHz, $CDCl_3$) δ 21.8, 28.1, 29.5, 32.1, 62.7, 66.1, 67.8, 122.0, 126.1, 128.1, 128.5, 129.3, 131.5, 133.2, 135.9, 147.7, 151.5, 165.9 (1 aryl carbon signal is obscured). IR (ATR): 3091, 3035, 2869, 2364, 1713, 1642, 1521, 1436, 957, 751 cm^{-1}. HRMS (ESI) *m/z* calcd for $C_{25}H_{28}N_4O_5SNa$ $[M+Na]^+$: 519.1673, found 519.1672.

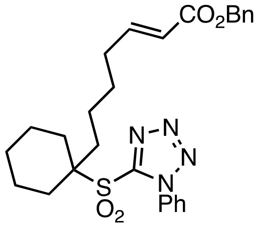

Benzyl 7-(1-(1'-phenyl-1*H*-tetrazol-5'-yl)sulfonylcyclohexyl)-2-heptanoate 11c

Purification by column chromatography (EtOAc/hexane = 1:10).

73.9 mg, 51% isolated yield (0.29 mmol scale); beige solid.

^{1}H NMR (500 MHz, $CDCl_3$) δ 1.17-1.22 (m, 1H), 1.35-1.40 (m, 4H), 1.44-1.50 (m, 1H), 1.62-1.65 (m, 1H), 1.72-1.74 (m, 2H), 1.88-1.93 (m, 6H), 2.17 (q, J = 14.0 Hz, 2H), 5.17 (s, 2H), 5.85 (d, J = 15.5 Hz, 1H), 6.96 (dt, J = 15.5, 7.0 Hz, 1H), 7.31-7.37 (m, 5H), 7.53-7.62 (m, 5H). ^{13}C NMR (125 MHz, $CDCl_3$) δ 21.1, 22.7, 24.2, 27.9, 28.2, 29.3, 31.7, 65.9, 70.4, 121.1, 126.2, 128.0, 128.4, 129.1, 131.3, 133.3, 135.9, 149.1, 149.2, 151.8, 166.2. IR (ATR): 3053, 3041, 2940, 2370, 1717, 1643, 1456, 1441, 797, 703 cm^{-1}. HRMS (ESI) *m/z* calcd for $C_{27}H_{32}N_4O_4SNa$ $[M+Na]^+$: 531.2036, found 531.2036.

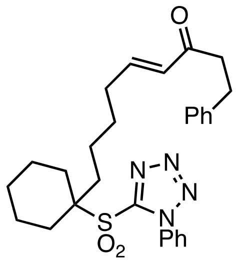

(*4E*)-1-phenyl-9-(1-(1'-phenyl-1*H*-tetrazol-5'-yl)sulfonylcyclohexyl)-4-nonen-3-one 11d

Purification by column chromatography (EtOAc/hexane = 1:20).

57.2 mg, 34% isolated yield (0.34 mmol scale); white solid.

^{1}H NMR (600 MHz, $CDCl_3$) δ 1.18-1.22 (m, 1H), 1.36-1.41 (m, 4H), 1.46-1.51 (m, 2H), 1.63-1.66 (m, 1H), 1.72-1.75 (m, 2H), 1.88-1.95 (m, 6H), 2,16-2.20 (m, 2H), 2.85 (t, J = 7.8 Hz, 2H), 2.93 (t, J = 7.8 Hz, 2H), 6.08 (d, J = 16.2 Hz, 1H), 6.77 (dt, J = 16.2, 6.6 Hz, 1H), 7.17-7.20 (m, 3H), 7.26-7.28 (m, 3H), 7.52-7.63 (m, 5H). ^{13}C NMR (150 MHz, $CDCl_3$) δ 21.2, 22.7, 24.2, 28.0, 28.4, 29.4, 30.0, 31.9, 41.7, 70.5, 126.0, 126.3, 128.3, 128.4, 129.1, 130.4, 131.3, 133.4, 141.2, 146.6, 151.9, 199.3. IR (ATR): 3053, 2968, 1696, 1457, 1437, 1049, 760, 727 cm^{-1}. HRMS (ESI) *m/z* calcd for $C_{28}H_{34}N_4O_3SNa$ $[M+Na]^+$: 529.2244, found 529.2244.

4. Typical Procedure for Zn/phen-mediated Giese reaction of tertiary sulfone 1 with electron-deficient olefins 2

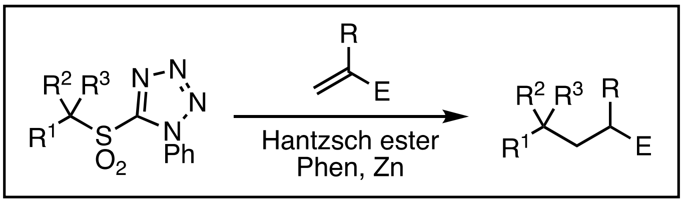

To A 10-mL sealable glass vessel containing a magnetic stirring bar was added Zn powder (32.7 mg, 0.5 mmol), flame-dried under vacuum, and filled with argon after cooling to room temperature. The tube was charged with 5-((1',1'-dimethyl-3'-phenylpropyl)sulfonyl)-1-phenyl-*1H*-tetrazole **1a** (71.2 mg, 0.2 mmol), 1,10-phenanthroline (108 mg, 0.6 mmol), and Hantzsch ester (101 mg, 0.4 mmol). The mixture was evacuated under vacuum and refilled with Ar. This cycle was repeated two additional times. Under an argon atmosphere, DMF (1.0 mL) and benzyl acrylate **2a** (61 µL, 0.4 mmol) were added and the reaction was sealed, and stirred at 25 ºC for 16 h. The reaction was quenched with 3-4 drops of H_2O and EtOAc (~1.0 mL). The mixture was passed through a pad of silica gel with copious washings with EtOAc. The filtrate was concentrated under reduced pressure. The crude product was purified by PTLC (Hexane/EtOAc = 40:1) to afford benzyl 4,4-dimethyl-6-phenylhexanoate **3aa** (50.7 mg, 82% yield) as a colorless oil.

Table S1. Optimization of reaction conditions

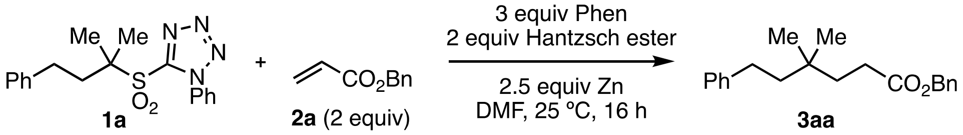

Variation	Yield (^{1}H-NMR)	Variation	Yield (^{1}H-NMR)
None	84%	2,2-bipydiryl instead of phen	0%
No Zn	0%	terpyridine instead of phen	25%
No phen	0%	2 equiv phen	61%
Zn pre-activated by TMSCl, no phen	0%	4 equiv phen	74%
Ricke Zn, no phen	0%	1.5 equiv Hantzsch ester	67%
Zn (particle size 75-150 um)	0%	No Hantzsch ester	26%
Mg instead of Zn	0%	2 equiv MeOH instead of Hantzsch ester	28%
Mn instead of Zn	0%	50 °C	77%
2 equiv Zn	71%	2 equiv LiCl	58%
4 equiv Zn	79%	10 mol% $NiCl_2$, 2 equiv $MgCl_2$	59%
DMAP instead of phen	0%	1.5 equiv **2a**	55%

Yields were determined by ^{1}H NMR using anisole as an internal standard.

Table S2. Reaction in DMF-d_7

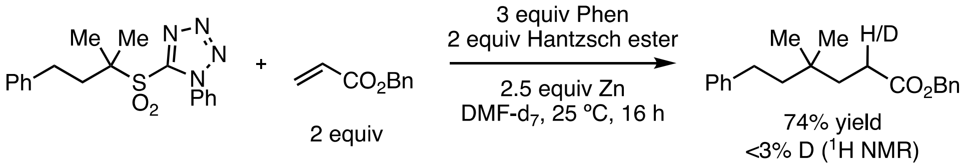

Table S3. Reaction using deuterated Hantzsch esters

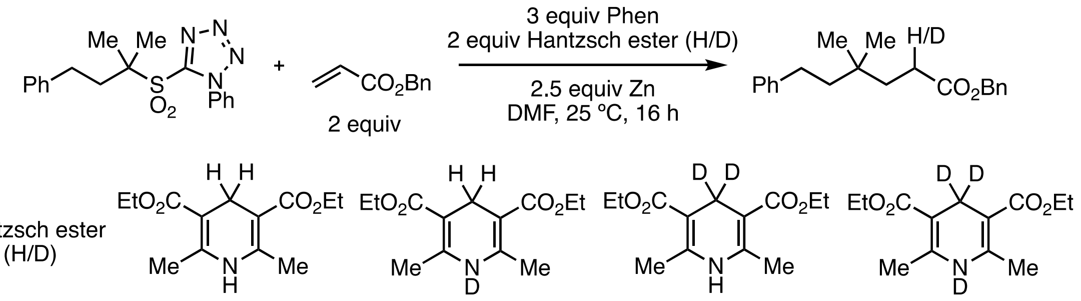

^{1}H-NMR yield	84%	78%	53%	43%
H/D ratio	100:0	94:6	50:50	14:86

Table S4. ICP-AES analysis of Zn powder

Fe	Co	Ni	Cu	Pd
0.5 ppm	<0.1 ppm	<0.5 ppm	2.5 ppm	<0.4 ppm

Table S5. Competitive reaction of sulfone and redox-active ester in Ni-catalyzed Giese reaction.[11]

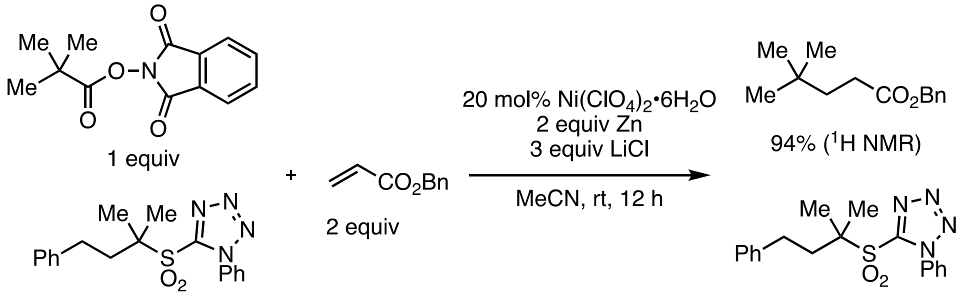

Compound Data for Quaternary product 3

Unless otherwise noted, sulfone derivatives described in this section were prepared following the typical procedure. All products were purified by column chromatography, PTLC or GPC (preparative recycling HPLC equipped with JAIGEL-1H/JAIGEL-2H column (eluent: $CHCl_3$)).

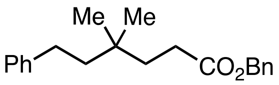

Benzyl 4,4-dimethyl-6-phenylhexanoate 3aa[12]

Purification by PTLC (Hexane/EtOAc = 40:1).

50.7 mg, 82% isolated yield (0.2 mmol scale); colorless oil.

^{1}H NMR (600 MHz, $CDCl_3$) δ 0.93 (s, 6H), 1.48-1.51 (m, 2H), 1.64-1.67 (m, 2H), 2.33-2.36 (m, 2H), 2.53-2.56 (m, 2H), 5.11 (s, 2H), 7.15-7.16 (m, 3H), 7.26 (t, J = 7.2 Hz, 2H), 7.30-7.36 (m, 5H). ^{13}C NMR (150 MHz, $CDCl_3$) δ 26.7, 29.6, 30.6, 32.6, 36.3, 44.0, 66.2, 125.6, 128.17, 128.20, 128.24, 128.3, 128.5, 136.0, 143.1, 174.1. IR (ATR): 3029, 2953, 1737,1506, 1462, 1148, 740 cm^{-1}. HRMS (DART) *m/z* calcd for $C_{21}H_{27}O_2$ $[M+H]^+$: 312.2011, found 312.2006.

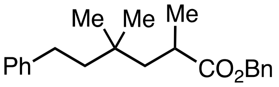

Benzyl 2,4,4-trimethyl-6-phenylhexanoate 3ab

Purification by PTLC (Hexane/EtOAc = 40:1).

40.0 mg, 62% isolated yield (0.2 mmol scale); colorless oil.

^{1}H NMR (600 MHz, $CDCl_3$) δ 0.90 (s, 3H), 0.91 (s, 3H), 1.19 (d, J = 7.8 Hz, 3H), 1.26 (dd, J = 8.4, 2.4 Hz, 1H), 1.49 (d, J = 8.4 Hz, 2H), 1.96-2.00 (m, 1H), 2.52-2.57 (m, 2H), 2.58-2.60 (m, 1H), 5.06 (d, J = 12.0 Hz, 1H), 5.09 (d, J = 12.0 Hz, 1H), 7.14-7.15 (m, 3H), 7.25 (t, J = 7.2 Hz, 2H), 7.29-7.34 (m, 5H). ^{13}C NMR (150 MHz, $CDCl_3$) δ 20.5, 26.88, 26.90, 30.6, 33.4, 35.8, 44.4, 45.6, 66.2, 125.5, 128.1,128.2, 128.26, 128.28, 128.5, 136.0, 143.2, 177.6. IR (ATR): 3205, 2953, 1737, 1555, 1454, 1170, 733 cm^{-1}. HRMS (DART) *m/z* calcd for $C_{22}H_{29}O_2$ $[M+H]^+$: 325.2168, found 325.2165.

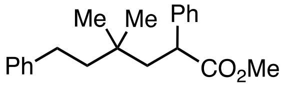

Methyl 4,4-dimethyl-2,6-diphenylhexanoate 3ac

Purification by PTLC (Hexane/EtOAc = 20:1).

36.2 mg, 58% isolated yield (0.2 mmol scale); colorless oil.

^{1}H NMR (600 MHz, $CDCl_3$) δ 0.94 (s, 6H), 1.50-1.53 (m, 2H), 1.66-1.69 (m, 1H), 2.35-2.40 (m, 1H), 2.46-2.57 (m, 2H), 3.61 (s, 3H), 3.68-3.70 (m, 1H), 7.11-7.16 (m, 3H), 7.22-7.26 (m, 3H), 7.29-7.34 (m, 4H). ^{13}C NMR (150 MHz, $CDCl_3$) δ 27.0, 30.6, 33.6, 44.4, 45.2, 47.6, 52.0, 125.6, 127.1, 127.8, 128.2, 128.3, 128.7, 140.9, 143.1, 175.2. IR (ATR): 3026, 2947, 1737, 1454, 1433, 1204, 730 cm^{-1}. HRMS (DART) *m/z* calcd for $C_{21}H_{27}O_2$ $[M+H]^+$: 311.2011, found 311.2009.

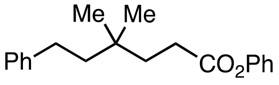

Phenyl 4,4-dimethyl-6-phenylhexanoate 3ad[13]

Purification by PTLC (Hexane/EtOAc = 40:1).

43.4 mg, 73% isolated yield (0.2 mmol scale); pale yellow oil.

^{1}H NMR (600 MHz, $CDCl_3$) δ 1.00 (s, 6H), 1.54-1.57 (m, 2H), 1.75-1.78 (m, 2H), 2.53-2.55 (m, 2H), 2.57-2.60 (m, 2H), 7.06 (d, J = 7.8 Hz, 2H), 7.16-7.22 (m, 4H), 7.27 (t, J = 7.8 Hz, 2H), 7.37 (t, J = 7.8 Hz, 2H). ^{13}C NMR (150 MHz, $CDCl_3$) δ 26.7, 29.7, 30.6, 32.7, 36.4, 44.0, 121.5, 125.6, 125.7, 128.25, 128.34, 129.3, 143.0, 150.7, 172.7. IR (ATR): 3205, 2955, 1750, 1493, 1456, 1118, 701 cm^{-1}. HRMS (DART) *m/z* calcd for $C_{20}H_{25}O_2$ $[M+H]^+$: 297.1855, found 297.1859.

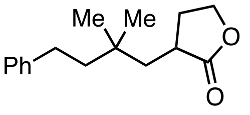

3-(2’,2’-Dimethyl-4’-phenylbutyl)dihydrofuran-2’(3H)-one 3ae[13]

Purification by PTLC (Hexane/EtOAc = 20:1).

30.6 mg, 62% isolated yield (0.2 mmol scale); yellow oil.

^{1}H NMR (600 MHz, $CDCl_3$) δ 1.00 (s, 3H), 1.03 (s, 3H), 1.34 (dd, J = 14.4, 8.4 Hz, 1H), 1.51-1.59 (m, 2H), 1.93-2.01 (m, 1H), 2.14 (d, J = 14.4 Hz, 1H), 2.48-2.52 (m, 2H), 2.54-2.64 (m, 2H), 4.13 (td, J = 9.6, 6.0 Hz, 1H), 4.33 (t, J = 8.4 Hz, 1H), 7.16-7.18 (m, 3H), 7.27 (t, J = 7.8 Hz, 2H). ^{13}C NMR (150 MHz, $CDCl_3$) δ 27.1, 27.3, 30.5, 31.7, 33.1, 36.0, 43.0, 44.5, 66.1, 125.6, 128.2, 128.3, 142.9, 180.2. IR (ATR): 3029, 2955, 1765, 1453, 1437, 1146, 1025, 727 cm^{-1}. HRMS (DART) *m/z* calcd for $C_{16}H_{23}O_2$ $[M+H]^+$: 247.1698, found 247.1695.

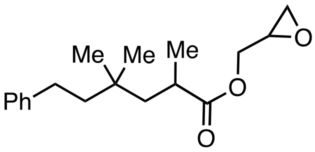

Oxiran-2-ylmethyl 2,4,4-trimethyl-6-phenylhexanoate 3af

Purification by PTLC (Hexane/EtOAc = 40:1) and GPC.

18.1 mg, 62% isolated yield (0.1 mmol scale, diastereomeric ratio = 1:1); colorless oil.

^{1}H NMR (600 MHz, $CDCl_3$, mixture of diastereomers) δ 0.93 (s, 6H), 1.199 (d, *J* = 7.2 Hz, 1.5H), 1.203 (d, *J* = 7.2 Hz, 1.5H), 1.272 (d, *J* = 14.4 Hz, 0.5H), 1.276 (d, *J* = 14.4 Hz, 0.5H), 1.50 (d, *J* = 9.0 Hz, 2H), 1.964 (dd, *J* = 14.4, 8.4 Hz, 0.5H), 1.967 (dd, *J* = 14.4, 8.4 Hz, 0.5H), 2.53-2.63 (m, 4H), 2.79 (t, *J* = 4.2 Hz, 0.5H), 2.80 (t, *J* = 4.2 Hz, 0.5H), 3.13-3.18 (m, 1H), 3.86-3.90 (m, 1H), 4.35-4.36 (m, 0.5H), 4.37-4.39 (m, 0.5H), 7.15-7.18 (m, 3H), 7.26 (d, *J* = 7.2 Hz, 2H). ^{13}C NMR (150 MHz, $CDCl_3$, mixture of diastereomers) δ 20.5, 26.85/26.88, 30.6, 33.4, 35.62/35.67, 44.4, 44.58/44.61, 45.66/45.72, 49.3, 64.84/64.97, 125.6, 128.26/128.29, 143.2, 177.53/177.56. IR (ATR): 3025, 2936, 1745, 1462, 1455, 1445, 908, 742cm^{-1}. HRMS (DART) *m/z* calcd for $C_{18}H_{27}O_3$ $[M+H]^+$: 291.1960, found 291.1955.

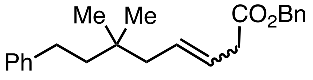

Benzyl 6,6-dimethyl-8-phenyl-3-octenoate 3ag

Purification by PTLC (Hexane/EtOAc = 40:1).

46.8 mg, 70% isolated yield (0.2 mmol scale, *trans:cis* = 17:1); white solid.

^{1}H NMR (600 MHz, $CDCl_3$, mixture of isomers) δ 0.91 (s, 5.6H), 0.93 (s, 0.4H), 1.42-1.52 (m, 2H), 2.00 (d, *J* = 6.0 Hz, 2H), 2.53-2.56 (m, 2H), 3.10 (d, *J* = 6.0 Hz, 1.9H), 3.15 (d, *J* = 6.0 Hz, 0.1H), 5.10 (s, 0.06H), 5.11 (s, 0.94H), 5.54-5.63 (m, 1.9H), 5.66-5.72 (m, 0.1H), 7.14-7.16 (m, 3H), 7.25 (t, *J* = 7.2 Hz, 2H), 7.30-7.33 (m, 5H). ^{13}C NMR (150 MHz, $CDCl_3$, mixture of isomers) (*trans*) δ 26.9, 30.6, 33.5, 38.2, 44.1, 44.8, 66.3, 123.8, 125.5, 128.2, 128.3, 128.5, 131.5, 135.9, 143.3, 171.8. (2 aryl carbon signal is obscured). IR (ATR): 3060, 3027, 2957, 1729, 1647, 1454, 1437, 969, 732 cm^{-1}. HRMS (DART) *m/z* calcd for $C_{23}H_{29}O_2$ $[M+H]^+$: 337.2168, found 337.2166.

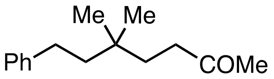

5,5-Dimethyl-7-phenyl-2-hepanone 3ah

Purification by PTLC (Hexane/EtOAc = 40:1).

33.3 mg, 76% isolated yield (0.2 mmol scale); pale yellow oil.

^{1}H NMR (600 MHz, $CDCl_3$) δ 0.93 (s, 6H), 1.48-1.51 (m, 2H), 1.54-1.57 (m, 2H), 2.15 (s, 3H), 2.39-2.41 (m, 2H), 2.53-2.56 (m, 2H), 7.15-7.17 (m, 3H), 7.27 (t, *J* = 7.2 Hz, 2H). ^{13}C NMR (150 MHz, $CDCl_3$) δ 26.8, 29.9, 30.6, 32.5, 35.1, 38.9, 44.0, 125.6, 128.2, 128.3, 143.1, 209.3. IR (ATR): 3027, 2959, 1713, 1468, 1452, 742 cm^{-1}. HRMS (ESI) *m/z* calcd for $C_{15}H_{22}ONa$ $[M+Na]^+$: 241.1563, found 241.1563.

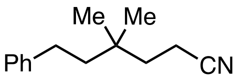

4,4-Dimethyl-6-phenylhexanenitrile 3ai[13]

Purification by PTLC (Hexane/EtOAc = 20:1).

31.5 mg, 78% isolated yield (0.2 mmol scale); pale yellow oil.

^{1}H NMR (600 MHz, $CDCl_3$) δ 0.97 (s, 6H), 1.49-1.52 (m, 2H), 1.69 (t, *J* = 7.8 Hz, 2H), 2.28 (t, *J* = 7.8 Hz, 2H), 2.53-2.56 (m, 2H), 7.16-7.19 (m, 3H), 7.28 (t, *J* = 7.8 Hz, 2H). ^{13}C NMR (150 MHz, $CDCl_3$) δ 12.3, 26.3, 30.5, 32.9, 37.2, 43.6, 120.4, 125.8, 128.2, 128.4, 142.5. IR (ATR): 3030, 2936, 2334, 1458, 1419, 740 cm^{-1}. HRMS (DART) *m/z* calcd for $C_{14}H_{20}N$ $[M+H]^+$: 202.1596, found 202.1600.

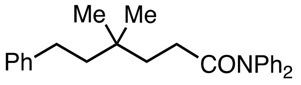

4,4-Dimethyl-*N*,*N*,6-triphenylhexanamide 3aj

Reaction temperature was 50 ºC

Purification by PTLC (Hexane/EtOAc = 20:1) and GPC.

55.2 mg, 74% isolated yield (0.2 mmol scale); pale yellow oil.

^{1}H NMR (600 MHz, $CDCl_3$) δ 0.81 (s, 6H), 1.33-1.36 (m, 2H), 1.67-1.70 (m, 2H), 2.25-2.27 (m, 2H), 2.42-2.45 (m, 2H), 7.06-7.07 (m, 2H), 7.13-7.34 (m, 13H). ^{13}C NMR (150 MHz, $CDCl_3$) δ 26.7, 30.46, 30.50, 32.7, 37.0, 43.9, 125.48, 125.52, 128.2, 128.9, 143.0, 143.1, 173.7. IR (ATR): 3059, 2956, 1592, 1488, 1452, 748 cm^{-1}. HRMS (ESI) *m/z* calcd for $C_{26}H_{29}NONa$ [M+Na]$^+$: 394.2141, found 394.2141.

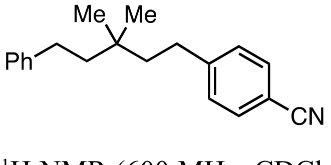

4-(3',3'-dimethyl-5'-phenylpentyl)benzonitrile 3ak

Purification by PTLC (Hexane/EtOAc = 40:1).

45.3 mg, 82% isolated yield (0.2 mmol scale); colorless oil.

^{1}H NMR (600 MHz, $CDCl_3$) δ 1.02 (s, 6H), 1.54-1.59 (m, 4H), 2.56-2.59 (m, 2H), 2.62-2.64 (m, 2H), 7.17-7.19 (m, 3H), 7.26-7.29 (m, 4H), 7.55 (d, *J* = 7.8 Hz, 2H). ^{13}C NMR (150 MHz, $CDCl_3$) δ 27.0, 30.7, 31.1, 33.2, 43.7, 44.0, 109.5, 119.1, 125.7, 128.2, 128.4, 129.1, 132.2, 143.1, 149.1. IR (ATR): 3030, 2936, 2228, 1606, 1414, 771 cm^{-1}. HRMS (DART) *m/z* calcd for $C_{20}H_{24}N$ [M+H]$^+$: 278.1909, found 278.1905.

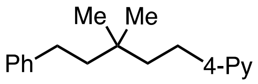

4-(3',3'-dimethyl-5'-phenylpentyl)pyridine 3al

Purification by PTLC (Hexane/EtOAc = 40:1).

34.4 mg, 68% isolated yield (0.2 mmol scale); colorless oil.

^{1}H NMR (600 MHz, $CDCl_3$) δ 1.02 (s, 6H), 1.55-1.59 (m, 4H), 2.55-2.60 (m, 4H), 7.10 (d, *J* = 4.8 Hz, 2H), 7.16-7.19 (m, 3H), 7.28 (t, *J* = 7.8 Hz, 2H), 8.47 (d, *J* = 4.8 Hz, 2H). ^{13}C NMR (150 MHz, $CDCl_3$) δ 27.0, 30.2, 30.7, 33.1, 42.9, 44.0, 128.3, 125.6, 128.2, 128.3, 143.1, 149.6, 152.3. IR (ATR): 3400, 3273, 3025, 2954, 1602, 1416, 804 cm^{-1}. HRMS (ESI) *m/z* calcd for $C_{18}H_{24}N$ [M+H]$^+$: 254.1903, found 254.1904.

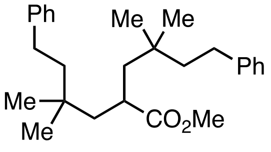

Methyl 2,2-bis(2',2'-dimethyl-4'-phenylbutyl)acetate 3am

3 equiv of **1a** and 1 equiv olefin **2m** were used.

Purification by PTLC (Hexane/EtOAc = 40:1) and GPC.

23.8 mg, 60% isolated yield (0.1 mmol scale); white solid.

^{1}H NMR (600 MHz, $CDCl_3$) δ 0.93 (s, 6H), 0.94 (s, 6H), 1.33 (d, *J* = 14.4 Hz, 2H), 1.50 (t, *J* = 8.4 Hz, 4H), 1.87 (dd, *J* = 14.4, 10.2 Hz, 2H), 2.48-2.57 (m, 4H), 2.59-2.63 (m, 1H), 3.59 (s, 3H), 7.15-7.17 (m, 6H), 7.25 (t, *J* = 7.2 Hz, 4H). ^{13}C NMR (150 MHz, $CDCl_3$) δ 27.0, 30.6, 33.7, 37.3, 44.4, 47.3, 51.4, 125.6, 128.28, 128.31, 143.2, 178.8. IR (ATR): 3088, 2949, 1736, 1520, 1432, 1157, 810 cm^{-1}. HRMS (ESI) *m/z* calcd for $C_{27}H_{38}O_2Na$ [M+Na]$^+$: 417.2764, found 417.2763.

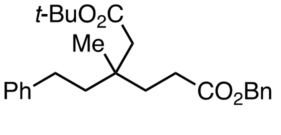

1-Benzyl 6-*tert*-butyl 4-methyl-4-(2'-phenylethyl)hexanedioate 3ba

Purification by PTLC (Hexane/EtOAc = 20:1).

26.1 mg, 64% isolated yield (0.1 mmol scale); pale yellow oil.

^{1}H NMR (600 MHz, $CDCl_3$) δ 1.04 (s, 3H), 1.43 (s, 9H), 1.59-1.63 (m, 2H), 1.76-1.80 (m, 2H), 2.19 (s, 2H), 2.40-2.42 (m, 2H), 2.58-2.60 (m, 2H), 5.11 (s, 2H), 7.15-7.18 (m, 3H), 7.26 (t, J = 7.8 Hz, 2H), 7.31-7.37 (m, 5H). ^{13}C NMR (150 MHz, $CDCl_3$) δ 24.8, 28.1, 29.2, 30.1, 34.2, 35.7, 41.7, 44.5, 66.3, 80.4, 125.7, 128.19, 128.24, 128.27, 128.35, 128.5, 136.0, 142.6, 171.0, 173.6. IR (ATR): 3028, 2940, 1737, 1454, 1153, 750 cm^{-1}. HRMS (DART) *m/z* calcd for $C_{26}H_{35}O_4$ $[M+H]^+$: 411.2535, found 411.2532.

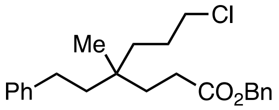

Benzyl 7-chloro-4-methyl-4-(2'-phenylethyl)heptanoate 3ca

Purification by PTLC (Hexane/EtOAc = 20:1).

23.3 mg, 63% isolated yield (0.1 mmol scale); pale yellow oil.

^{1}H NMR (600 MHz, $CDCl_3$) δ 0.92 (s, 3H), 1.38-1.41 (m, 2H), 1.48-1.51 (m, 2H), 1.66-1.69 (m, 2H), 1.72-1.77 (m, 2H), 2.32-2.35 (m, 2H), 2.51-2.54 (m, 2H), 3.51 (t, J = 6.6 Hz, 2H), 5.12 (s, 2H), 7.15-7.17 (m, 3H), 7.27 (t, J = 7.2 Hz, 2H), 7.31-7.38 (m, 5H). ^{13}C NMR (150 MHz, $CDCl_3$) δ 24.5, 27.0, 29.1, 30.1, 33.9, 34.8, 36.2, 41.3, 45.7, 66.3, 125.7, 128.2, 128.4, 128.56, 128.57, 136.0, 142.8, 173.9 (1 aryl carbon signal is obscured). IR (ATR): 3032, 2940, 1737, 1454, 1448, 1180, 1157, 746 cm^{-1}. HRMS (DART) *m/z* calcd for $C_{23}H_{30}O_2Cl$ $[M+H]^+$:373.1934, found 373.1931.

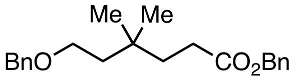

Benzyl 6-benzyloxy-4,4-dimethylhexanoate 3da

Purification by PTLC (Hexane/EtOAc = 30:1).

66.2 mg, 97% isolated yield (0.2 mmol scale); pale yellow oil.

^{1}H NMR (600 MHz, $CDCl_3$) δ 0.89 (s, 6H), 1.55-1.61 (m, 4H), 2.33 (t, J = 7.8 Hz, 2H), 3.51 (t, J = 7.8 Hz, 2H), 4.46 (s, 2H), 5.09 (s, 2H), 7.22-7.36 (m, 10H). ^{13}C NMR (150 MHz, $CDCl_3$) δ 27.0, 29.5, 31.8, 36.8, 40.6, 66.1, 67.1, 72.9, 127.4, 127.5, 128.10, 128.13, 128.3, 128.5, 136.0, 138.5, 174.0. IR (ATR): 3025, 2957, 1734, 1540, 1458, 1118, 1095, 1010, 731 cm^{-1}. HRMS (DART) *m/z* calcd for $C_{22}H_{29}O_3$ $[M+H]^+$: 341.2117, found 341.2117.

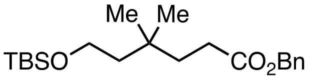

Benzyl 6-((*tert*-butyldimethylsilyl)oxy)-4,4-dimethylhexanoate 3ea[12]

Purification by PTLC (Hexane/EtOAc = 40:1).

27.1 mg, 74% isolated yield (0.1 mmol scale); colorless oil.

^{1}H NMR (600 MHz, $CDCl_3$) δ 0.04 (s, 6H), 0.88 (s, 9H), 0.89 (s, 6H), 1.46 (t, J = 7.2 Hz, 2H), 1.59 (t, J = 8.4 Hz, 2H), 2.34 (t, J = 8.4 Hz, 2H), 3.65 (t, J = 7.2 Hz, 2H), 5.10 (s, 2H), 7.32-7.38 (m, 5H). ^{13}C NMR (150 MHz, $CDCl_3$) δ -5.3, 18.3, 25.9, 27.1, 29.6, 31.9, 36.9, 43.8, 59.8, 66.2, 128.17, 128.21, 128.5, 136.1, 174.1. IR (ATR): 3043, 2953, 1735, 1472, 1448, 1256, 1094, 829, 740 cm^{-1}. HRMS (DART) *m/z* calcd for $C_{21}H_{37}O_3Si$ $[M+H]^+$: 365.2512, found 365.2510.

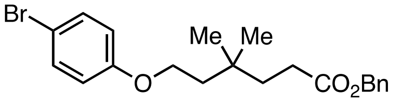

Benzyl 6-(4'-bromophenyloxy)-4,4-dimethylhexanoate 3fa

Purification by GPC.

28.2 mg, 70% isolated yield (0.1 mmol scale); yellow oil.

^{1}H NMR (600 MHz, $CDCl_3$) δ 0.95 (s, 6H), 1.65-1.67 (m, 2H), 1.70 (t, J = 7.2 Hz, 2H), 2.36-2.39 (m, 2H), 3.96 (t, J = 7.2 Hz, 2H), 5.11 (s, 2H), 6.73 (d, J = 9.0 Hz, 2H), 7.33-7.38 (m, 7H). ^{13}C NMR (150 MHz, $CDCl_3$) δ 27.1, 29.6, 32.0, 36.8, 39.9, 64.9, 66.3, 112.6, 116.2, 128.2, 128.6, 132.2, 136.0, 157.9, 173.9. (1 aryl carbon signal is obscured). IR (ATR): 3033, 2958, 1729, 1487, 1455, 1242, 1170, 740 cm^{-1}. HRMS (DART) *m/z* calcd for $C_{21}H_{26}O_3Br$ [M+H]$^+$: 405.1065, found 405.1063.

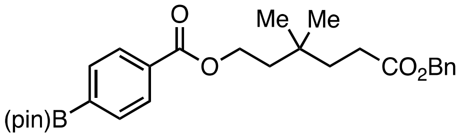

Benzyl 4,4-Dimethyl-6-(4-(4',4',5',5'-tetramethyl-1',3',2'-dioxaborolan-2'-yl)benzoyl)oxyhexanoate 3ga

Purification by GPC.

32.6 mg, 68% isolated yield (0.1 mmol scale); pale yellow oil.

^{1}H NMR (400 MHz, $CDCl_3$) δ 0.97 (s, 6H), 1.35 (s, 12H), 1.66-1.73 (m, 4H), 2.36-2.40 (m, 2H), 4.37 (t, J = 7.2 Hz, 2H), 5.10 (s, 2H), 7.32-7.36 (m, 5H), 7.86 (d, J = 8.0 Hz, 2H), 8.00 (d, J = 8.0 Hz, 2H). ^{13}C NMR (150 MHz, $CDCl_3$) δ 24.9, 26.9, 29.5, 32.0, 36.8, 39.5, 62.2, 66.3, 84.1, 128.2, 128.3, 128.5, 128.6, 132.5, 134.6, 136.0, 166.7, 173.8. IR (ATR): 3021, 2974, 1720, 1400, 1361, 1267, 1141, 709 cm^{-1}. HRMS (DART) *m/z* calcd for $C_{28}H_{38}BO_6$ [M+H]$^+$: 481.2761, found 481.2771.

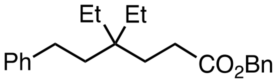

Benzyl 4,4-diethyl-6-phenylhexanoate 3ha

Purification by PTLC (Hexane/EtOAc = 40:1).

20.8 mg, 62% isolated yield (0.1 mmol scale); pale yellow oil.

^{1}H NMR (600 MHz, $CDCl_3$) δ 0.81 (t, J = 7.8 Hz, 6H), 1.28 (q, J = 7.8 Hz, 4H), 1.43-1.46 (m, 2H), 1.61-1.64 (m, 2H), 2.27-2.30 (m, 2H), 2,45-2.48 (m, 2H), 5.12 (s, 2H), 7.15-7.18 (m, 3H), 7.26 (t, J = 7.8 Hz, 2H), 7.30-7.38 (m, 5H). ^{13}C NMR (150 MHz, $CDCl_3$) δ 7.4, 27.7, 28.7, 29.7, 30.2, 37.2, 37.8, 66.2, 125.6, 128.16, 128.17, 128.21, 128.3, 128.5, 136.0, 143.2, 174.2. IR (ATR): 3027, 2936, 1522, 1453, 1152, 1147, 702 cm^{-1}. HRMS (DART) *m/z* calcd for $C_{23}H_{31}O_2$ [M+H]$^+$: 339.2324, found 339.2330.

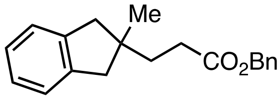

2-(2'-Benzyloxycarbonyl)ethyl-2,3-dihydroxy-2-methyl-*1H*-indene 3ia

Purification by PTLC (Hexane/EtOAc = 40:1).

21.2 mg, 72% isolated yield (0.1 mmol scale); white solid.

^{1}H NMR (600 MHz, $CDCl_3$) δ 1.06 (s, 3H), 1.87 (t, J = 8.4 Hz, 2H), 2.39 (t, J = 8.4 Hz, 2H), 2.65 (d, J = 15.6 Hz, 2H), 2.80 (d, J = 15.6 Hz, 2H), 5.11 (s, 2H), 7.10-7.13 (m, 5H), 7.31-7.37 (m, 5H). ^{13}C NMR (150 MHz, $CDCl_3$) δ 25.9, 30.8, 36.6, 42.7, 45.9, 66.3, 124.7, 126.2, 128.2, 128.3, 128.5, 136.0, 142.6, 173.8. IR (ATR): 3056, 2923, 1730, 1646, 1437, 1153, 738 cm^{-1}. HRMS (DART) *m/z* calcd for $C_{20}H_{21}O_2$ [M-H]$^+$: 293.1542, found 293.1539.

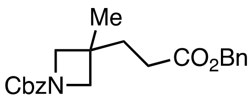

Benzyl 3-(1'-benzyloxycarboyl-3-methylazetidin-3-yl)propanoate 3ja

Purification by GPC.

18.9 mg, 51% isolated yield (0.1 mmol scale); pale yellow oil.

^{1}H NMR (600 MHz, $CDCl_3$) δ 1.22 (s, 3H), 1.93 (t, J = 7.8 Hz, 2H), 2.33 (t, J = 7.8 Hz, 2H), 3.62 (d, J = 7.8 Hz, 2H), 3.71 (d, J = 7.8 Hz, 2H), 5.08 (s, 2H), 5.12 (s, 2H), 7.30-7.38 (m, 10H). ^{13}C NMR (150 MHz, $CDCl_3$) δ 23.9, 29.8, 33.9, 34.3, 59.4, 60.2, 66.5, 66.6, 127.91, 127.98, 128.30, 128.32, 128.4, 128.6, 135.7, 136.7, 156.5, 172.9. IR (ATR): 3056, 2955, 1731, 1453, 1422, 1356, 1100, 702 cm^{-1}. HRMS (ESI) *m/z* calcd for $C_{22}H_{25}O_4NNa$ $[M+Na]^+$: 390.1676, found 390.1676.

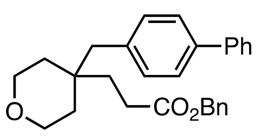

Benzyl 4-biphenylmehyl-tetrahydro-*2H*-pyran-4-propanoate 3ka

Purification by PTLC (Hexane/EtOAc = 20:1).

21.9 mg, 53% isolated yield (0.1 mmol scale); pale yellow oil.

^{1}H NMR (600 MHz, $CDCl_3$) δ 1.38-1.42 (m, 2H), 1.52-1.56 (m, 2H), 1.75-1.77 (m, 2H), 2.42-2.45 (m, 2H), 2.68 (s, 2H), 3.66-3.70 (m, 2H), 3.76-3.79 (m, 2H), 5.13 (s, 2H), 7.16 (d, J = 7.8 Hz, 2H), 7.32-7.37 (m, 5H), 7.43 (t, J = 7.2 Hz, 2H), 7.47 (d, J = 7.2 Hz, 2H), 7.56 (d, J = 7.8 Hz, 2H). ^{13}C NMR (150 MHz, $CDCl_3$) δ 28.7, 30.2, 34.3, 34.9, 42.9, 63.5, 66.4, 126.7, 126.9, 127.1, 128.3, 128.6, 128.7, 130.9, 135.9, 136.4, 139.2, 140.8, 173.6 (1 aryl carbon signal is obscured). IR (ATR): 3014, 2955, 1735, 1448, 1166, 1157, 1103, 739 cm^{-1}. HRMS (ESI) *m/z* calcd for $C_{28}H_{30}O_3Na$ $[M+Na]^+$: 437.2087, found 437.2087.

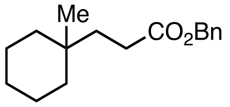

Benzyl 3-(1-methylcyclohexyl)propanoate 3la[12]

Purification by PTLC (Hexane/EtOAc = 40:1).

14.5 mg, 56% isolated yield (0.1 mmol scale); colorless oil.

^{1}H NMR (600 MHz, $CDCl_3$) δ 0.84 (s, 3H), 1.22-1.25 (m, 5H), 1.40-1.46 (m, 5H), 1.60 (t, J = 8.4 Hz, 2H), 2.31 (t, J = 8.4 Hz, 2H), 5.11 (s, 2H), 7.31-7.38 (m, 5H). ^{13}C NMR (150 MHz, $CDCl_3$) δ 21.9, 24.4, 26.4, 29.0, 32.3, 36.7, 37.5, 66.1, 128.1, 128.2, 128.5, 136.1, 174.4. IR (ATR): 3065, 2931, 1743, 1560, 1419, 1163, 749 cm^{-1}. HRMS (DART) *m/z* calcd for $C_{17}H_{23}O_2$ $[M-H]^+$: 259.1698, found 259.1697.

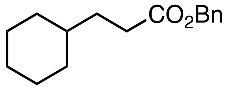

Benzyl 3-cyclohexylpropanoate 3ma[14]

Purification by PTLC (Hexane/EtOAc = 40:1).

12.5 mg, 25% isolated yield (0.2 mmol scale); colorless oil.

^{1}H NMR (600 MHz, $CDCl_3$) δ 0.85-0.91 (m, 2H), 1.11-1.26 (m, 4H), 1.53-1.56 (m, 2H), 1.62-1.65 (m, 1H), 1.68-1.70 (m, 4H), 2.36 (t, J = 7.8 Hz, 2H), 5.11 (s, 2H), 7.31-7.37 (m, 5H). ^{13}C NMR (150 MHz, $CDCl_3$) δ 26.2, 26.5, 31.9, 32.3, 32.9, 37.2, 66.1, 128.1, 128.2, 128.5, 136.2, 174.0.IR (ATR): 3094, 2923, 1738, 1490, 1448, 1157, 728 cm^{-1}. HRMS (DART) *m/z* calcd for $C_{16}H_{21}O_2$ $[M-H]^+$: 245.1542, found 245.1549.

Table S6. Unsuccessful olefin substrates

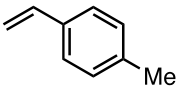
<20%

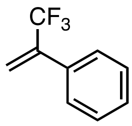
<10%

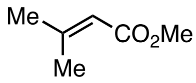
n.d.

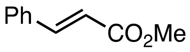
n.d.

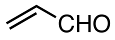
n.d.

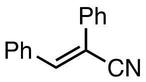
n.d.

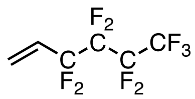
n.d.

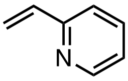
n.d.

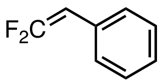
n.d.

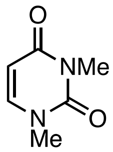
n.d.

5. Intermolecular Giese reaction of tertiary sulfone 11

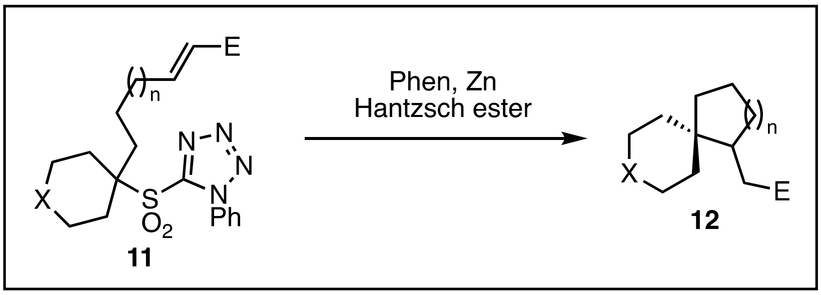

To A 10-mL sealable glass vessel containing a magnetic stirring bar was added Zn powder (16.4 mg, 0.25 mmol), flame-dried under vacuum, and filled with argon after cooling to room temperature. The tube was charged with benzyl 6-(1-(1'-phenyl-1*H*-tetrazol-5'-yl)sulfonylcyclohexyl)-2-hexanoate **11a** (49.5 mg, 0.1 mmol), 1,10-phenanthroline (54.1 mg, 0.3 mmol), and Hantzsch ester (50.7 mg, 0.2 mmol). The mixture was evacuated under vacuum and refilled with Ar. This cycle was repeated two additional times. Under an argon atmosphere, DMF (0.5 mL) were added and the reaction was sealed, and stirred at 25 ºC for 16 h. The reaction was quenched with 3-4 drops of H_2O and EtOAc (~1.0 mL). The mixture was passed through a pad of silica gel with copious washings with EtOAc. The filtrate was concentrated under reduced pressure. The crude product was purified by PTLC (Hexane/EtOAc = 50:1) to afford benzyl spiro[4,5]decane-1-acetate **12a** (22.0 mg, 77% yield) as a colorless oil.

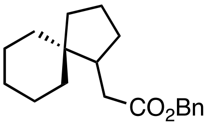

Benzyl spiro[4,5]decane-1-acetate 12a

Purification by PTLC (Hexane/EtOAc = 50:1).

22.0 mg, 77% isolated yield (0.1 mmol scale); colorless oil.

^{1}H NMR (600 MHz, $CDCl_3$) δ 1.01 (td, *J* = 12.6, 4.2 Hz, 1H), 1.06-1.14 (m, 1H), 1.20-1.26 (m, 2H), 1.28-1.38 (m, 5H), 1.51-1.61 (m, 5H), 1.68-1.75 (m, 1H), 1.83-1.90 (m, 2H), 2.12 (dd, *J* = 14.4, 10.8 Hz, 1H), 2.45 (dd, *J* = 14.4, 4.2 Hz, 1H), 5.10 (d, *J* = 18.6 Hz, 1H), 5.13 (d, *J* = 18.6 Hz, 1H), 7.30-7.38 (m, 5H). ^{13}C NMR (150 MHz, $CDCl_3$) δ 21.4, 22.6, 23.9, 26.5, 30.1, 30.3, 34.8, 35.2, 37.3, 44.2, 46.5, 66.1, 128.1, 128.2, 128.5, 136.1, 174.1. IR (ATR): 3036, 2919, 1731, 1450, 1419, 1147, 735 cm^{-1}. HRMS (ESI) *m/z* calcd for $C_{19}H_{26}O_2Na$ $[M+Na]^+$: 309.1825, found 309.1825.

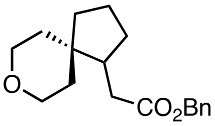

Benzyl 8-oxaspiro[4,5]decane-1-acetate 12b

Purification by PTLC (Hexane/EtOAc = 10:1).

21.9 mg, 76% isolated yield (0.1 mmol scale); pale yellow oil.

^{1}H NMR (600 MHz, $CDCl_3$) δ 1.12-1.16 (m, 2H), 1.30-1.36 (m, 1H), 1.43-1.49 (m, 2H), 1.59-1.75 (m, 3H), 1.81-1.85 (m, 1H), 1.89-1.94 (m, 2H), 2.14 (dd, *J* = 14.4, 10.8 Hz, 1H), 2.48 (dd, *J* = 14.4, 3.6 Hz, 1H), 3.48-3.53 (m, 2H), 3.79-3.83 (m, 2H), 5.12 (s, 2H), 7.31-7.38 (m, 5H). ^{13}C NMR (150 MHz, $CDCl_3$) δ 21.3, 29.8, 30.9, 34.1, 34.9, 36.9, 42.0, 46.3, 64.6, 65.6, 66.2, 128.18, 128.22, 128.5, 136.0, 176.5. IR (ATR): 3025, 2952, 1729, 1559, 1456, 1148, 1106, 1016, 752 cm^{-1}. HRMS (ESI) *m/z* calcd for $C_{18}H_{24}O_3Na$ $[M+Na]^+$: 311.1618, found 311.1617.

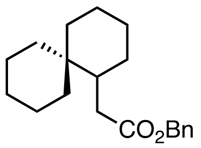

Benzyl spiro[5,5]undecane-1-acetate 12c

Purification by PTLC (Hexane/EtOAc = 50:1).

27.0 mg, 90% isolated yield (0.1 mmol scale); colorless oil.

^{1}H NMR (600 MHz, $CDCl_3$) δ 0.99 (td, *J* = 12.0, 3.6 Hz, 2H), 1.14-1.38 (m, 6H), 1.42-1.59 (m, 9H), 1.77-1.86 (m, 2H), 2.10 (dd, *J* = 15.0, 10.2 Hz, 1H), 2.58 (dd, *J* = 15.0, 3.6 Hz, 1H), 5.10 (d, *J* = 12.6 Hz, 1H), 5.12 (d, *J* = 12.6 Hz, 2H), 7.30-7.38 (m, 5H). ^{13}C NMR (150 MHz, $CDCl_3$) δ 21.0, 21.1, 21.2, 24.7, 26.7, 27.1, 29.2, 32.2, 34.9, 35.0, 36.2, 42.1, 66.0, 128.08, 128.12, 128.5, 136.2, 174.3. IR (ATR): 3072, 2920, 1728, 1454, 1447, 1143, 732 cm^{-1}. HRMS (ESI) *m/z* calcd for $C_{20}H_{28}O_2Na$ $[M+Na]^+$: 323.1982, found 323.1979.

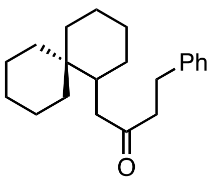

1-Phenyl-4-spiro[5,5]undec-1-yl-3-butanone 12d

Purification by PTLC (Hexane/EtOAc = 15:1).

16.1 mg, 54% isolated yield (0.1 mmol scale); colorless oil.

^{1}H NMR (600 MHz, $CDCl_3$) δ 0.91-0.97 (m, 2H), 1.09-1.17 (m, 3H), 1.24-1.53 (m, 12H), 1.74-1.78 (m, 1H), 1.87 (d, *J* = 13.2 Hz, 1H), 2.13 (dd, *J* = 15.6, 10.2 Hz, 1H), 2.53 (dd, *J* = 15.6, 3.6 Hz, 1H), 2.66-2.71 (m, 1H), 2.73-2.78 (m, 1H), 2.89 (t, *J* = 7.2 Hz, 2H), 7.17-7.19 (m, 3H), 7.26-7.29 (m, 2H). ^{13}C NMR (150 MHz, $CDCl_3$) δ 21.0, 21.15, 21.25, 25.0, 26.7, 27.4, 28.8, 29.9, 32.4, 35.0, 36.4, 41.0, 43.6, 44.8, 126.0, 128.3, 128.4, 141.2, 210.8. IR (ATR): 3083, 2916, 1711, 1540, 1448, 741 cm^{-1}. HRMS (ESI) *m/z* calcd for $C_{21}H_{30}ONa$ $[M+Na]^+$: 321.2189, found 321.2188.

6. Orthogonal Giese reaction of tertiary sulfone 13

A) Preparation of tertiary sulfone 13

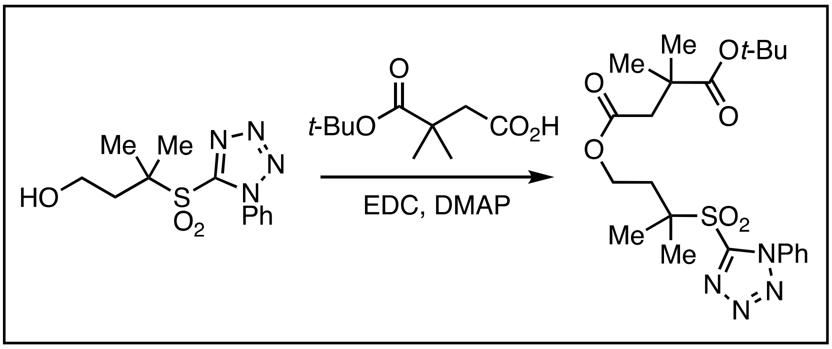

To a 30-mL flask were added 5-((1',1'-dimethyl-3'-hydroxypropyl)sulfonyl)-1-phenyl-*1H*-tetrazole (215 mg, 0.72 mmol), 1-(1,1-dimethylethyl) 2,2-dimethylbutanedioate[15] (160 mg, 0.80 mmol), 4-dimethylaminopyridine (122 mg, 1 mmol) and dry CH_2Cl_2 (5 mL) under a stream of argon. 1-(3-Dimethylaminopropyl)-3-ethylcarbodiimide (177 µL, 1 mmol) was added to this mixture at room temperature. After stirring at room temperature for 12 h, sat. NH_4Cl*aq* was added to the reaction mixture, and the layers were separated. The aqueous layer was extracted with EtOAc (3 times), and the combined organic layer was dried over Na_2SO_4, filtered and the solvent was evaporated under reduced pressure. The crude material was purified by column chromatography (Hexane/EtOAc = 5:1) to give the corresponding *t*-butyl ester (337 mg, 88%) as a colorless oil.

^{1}H NMR (600 MHz, $CDCl_3$) δ 1.22 (s, 6H), 1.43 (s, 9H), 1.56 (s, 6H), 2.31 (t, J = 7.2 Hz, 2H), 2.51 (s, 2H), 4.23 (t, J = 7.2 Hz, 2H), 7.32-7.37 (m, 5H). ^{13}C NMR (150 MHz, $CDCl_3$) δ 20.6, 25.4, 27.8, 33.5, 41.0, 43.9, 59.4, 66.6, 80.3, 126.1, 129.3, 131.5, 133.3, 151.7, 171.0, 175.6. IR (ATR): 3073, 2978, 1729, 1499, 1339, 1120, 760 cm^{-1}. HRMS (ESI) *m/z* calcd for $C_{22}H_{32}N_4O_6SNa$ $[M+Na]^+$: 503.1935, found 503.1935.

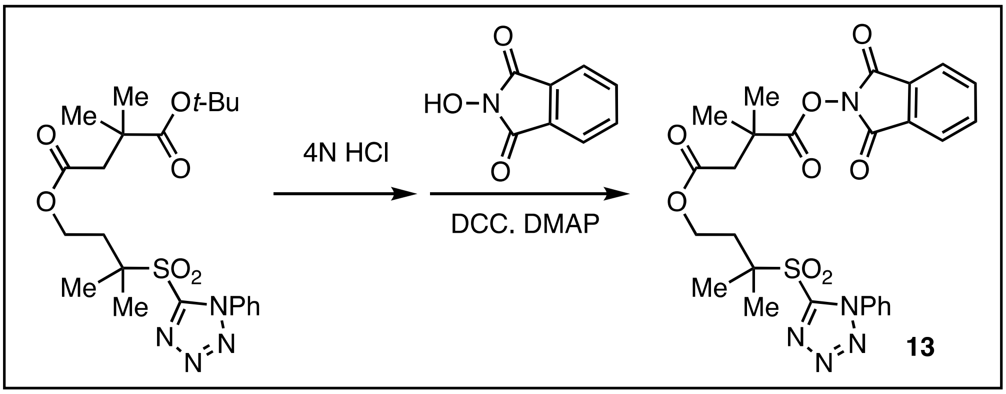

To a 20-mL flask were added *t*-butyl ester (144 mg, 0.3 mmol) and CH_2Cl_2 (1.1 mL) under a stream of argon. 4N HCl in dioxane (1.1 mL) was added to this mixture at room temperature. After stirring at room temperature for 12 h, brine was added to the reaction mixture, and the layers were separated. The aqueous layer was extracted with EtOAc (3 times), and the combined organic layer was dried over Na_2SO_4, filtered and the solvent was evaporated under reduced pressure.

To this flask were added *N*-hydroxyphthalimide (53.8 mg, 0.33 mmol), DCC (74.3 mg, 0.36 mmol), CH_2Cl_2 (2 mL), and 4-dimethylaminopyridine (5.5 mg, 0.045 mmol) under a stream of argon. After stirring at room temperature for 12 h, solids were filtered and the filtrate was evaporated under reduced pressure. The crude material was purified by column chromatography (Hexane/EtOAc = 5:1) to give the corresponding sulfone **13** (164 mg, 96%) as a white solid.

^{1}H NMR (600 MHz, $CDCl_3$) δ 1.51 (s, 6H), 1.58 (s, 6H), 2.30 (t, J = 7.8 Hz, 2H), 2.77 (s, 2H), 4.31 (t, J = 7.8 Hz, 2H), 7.57-7.62 (m, 5H), 7.78-7.80 (m, 2H), 7.88-7.89 (m, 2H). ^{13}C NMR (150 MHz, $CDCl_3$) δ 20.5, 25.5, 33.3, 40.2, 43.9, 60.1, 66.4, 124.0, 126.2, 128.9, 129.3, 131.4, 133.3, 134.7, 151.6, 161.8, 169.7, 172.7. IR (ATR): 3082, 2979, 1737, 1647, 1336, 1121, 1049, 764 cm^{-1}. HRMS (ESI) *m/z* calcd for $C_{26}H_{27}N_5O_8SNa$ $[M+Na]^+$: 592.1473, found 592.1472.

B) Intermolecular Giese reaction

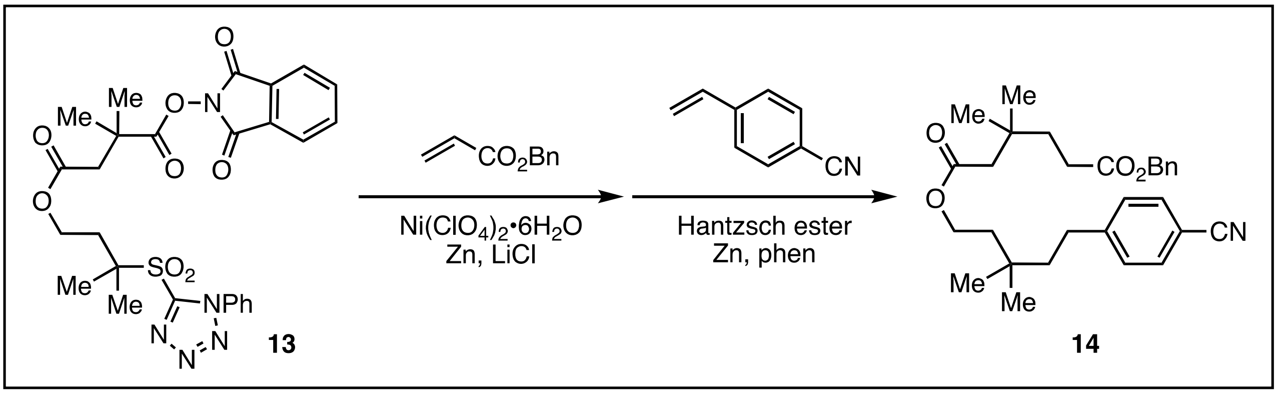

To A 10-mL sealable glass vessel containing a magnetic stirring bar was added Zn powder (26 mg, 0.4 mmol), flame-dried under vacuum, and filled with argon after cooling to room temperature. The tube was charged with sulfone **13** (114 mg, 0.2 mmol), $Ni(ClO_4)_2\cdot6H_2O$ (14.6 mg, 0.04 mmol), LiCl (25.4 mg, 0.6 mmol). The mixture was evacuated under vacuum and refilled with Ar. This cycle was repeated two additional times. Under an argon atmosphere, dry MeCN (1.0 mL) and benzyl acrylate **2a** (61.0 μL, 0.4 mmol) were added and the reaction was sealed, and stirred at 25 ºC for 12 h. The reaction was quenched with 3-4 drops of H_2O and EtOAc (~1.0 mL). The mixture was passed through a pad of silica gel with copious washings with EtOAc. The filtrate was concentrated under reduced pressure. Toluene was added and insoluble solids were filtered and the filtrate was evaporated under reduced pressure to remove **2a**. To this flask was added Zn powder (flame-dried under vacuum, 32.6 mg, 0.5 mmol), 1,10-phenanthroline (108 mg, 0.6 mmol), and Hantzsch ester (101 mg, 0.4 mmol). The mixture was evacuated under vacuum and refilled with Ar. This cycle was repeated two additional times. Under an argon atmosphere, DMF (1.0 mL) and *p*-cyanostyrene **2l** (51.7 mg, 0.4 mmol) were added and the reaction was sealed, and stirred at 25 ºC for 16 h. The reaction was quenched with 3-4 drops of H_2O and EtOAc (~1.0 mL). The mixture was passed through a pad of silica gel with copious washings with EtOAc. The filtrate was concentrated under reduced pressure. The crude product was purified by column (Hexane/EtOAc = 30:1) and GPC to afford **14** (60.1 mg, 65% yield) as a colorless oil. ^{1}H NMR (600 MHz, $CDCl_3$) δ 0.99 (s, 6H), 1.00 (s, 6H), 1.50-1.52 (m, 2H), 1.62 (t, J = 7.8 Hz, 2H), 1.70 (t, J = 8.4 Hz, 2H), 2.18 (s, 2H), 2.37 (t, J = 8.4 Hz, 2H), 2.61-2.64 (m, 2H), 4.12 (t, J = 7.8 Hz, 2H), 5.11 (s, 2H), 7.27 (d, J = 7.8 Hz, 2H), 7.32-7.37 (m, 5H), 7.55 (d, J = 7.8 Hz, 2H). ^{13}C NMR (150 MHz, $CDCl_3$) δ 27.0, 27.1, 29.5, 30.9, 32.4, 32.9, 36.5, 39.5, 43.9, 45.8, 61.2, 66.2, 109.5, 119.1, 128.2, 128.5, 129.1, 132.2, 135.9, 148.7, 171.9, 173.6. IR (ATR): 3052, 2958, 2228, 1728, 1471, 1155, 820 cm^{-1}. HRMS (ESI) *m/z* calcd for $C_{29}H_{37}NO_4Na$ $[M+Na]^+$: 486.2615, found 486.2615.

7. Preparation of samples for EPR and UV-Vis measurement

To A 10-mL sealable glass vessel containing a magnetic stirring bar was added Zn powder (32.7 mg, 0.5 mmol), flame-dried under vacuum, and filled with argon after cooling to room temperature. The tube was charged with 1,10-phenanthroline (108 mg, 0.6 mmol). The mixture was evacuated under vacuum and refilled with Ar. This cycle was repeated two additional times. Under an argon atmosphere, dry DMF (1.0 mL) was added and the reaction was sealed, and stirred at 25 ºC for 16 h.

For EPR measurement, supernatant was transferred into tube, and then DMF was removed under vacuum before measurement. For UV-Vis measurement, the mixture was diluted with dry DMF, and supernatant was used for measurement (0.2 mM). The solution of 1,9-phenanthlorine (c = 1.0 mM) and $Zn(phen)_3(OTf)_2$ (c = 1.4 mM) were prepared in DMF.

8. Cyclic Voltammetry Measurement

Cyclic voltammograms were taken on a CH Instruments 600E potentiostat using a 3 mm glassy carbon working electrode, $Ag/AgNO_3$ reference electrode, and a Pt wire counter electrode. The voltammograms were taken at room temperature in a 100 mM MeCN

solution of tetrabutylammonium hexafluorophosphate containing 1 mM of the designated substance. The scan rate was 0.2 V/s. At the end of each sample, a small amount of ferrocene was added as a reference. Conversion to SCE was achieved by adding 380 mV to the corrected potentials against Fc/Fc+.

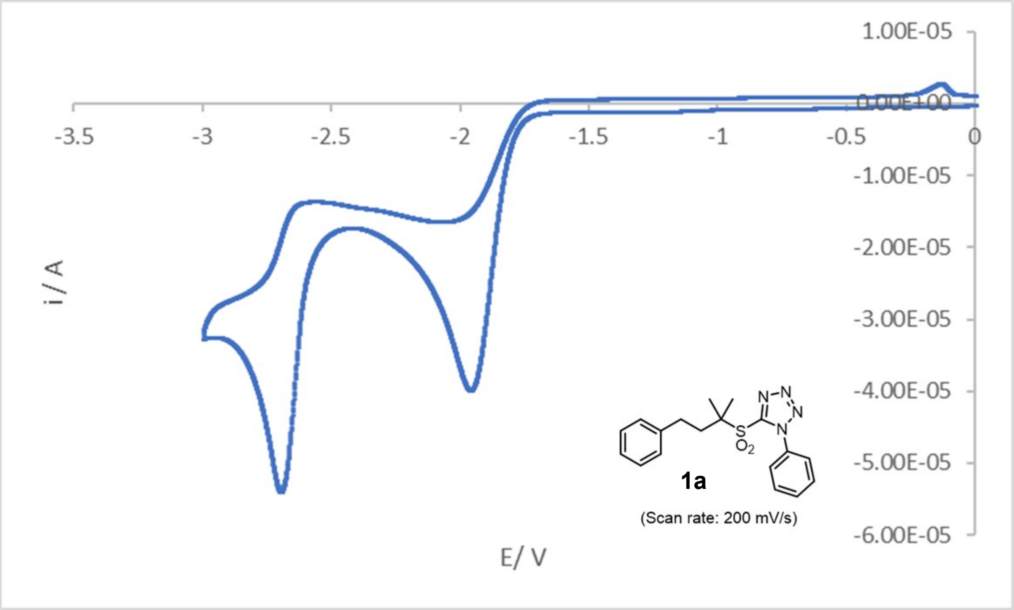

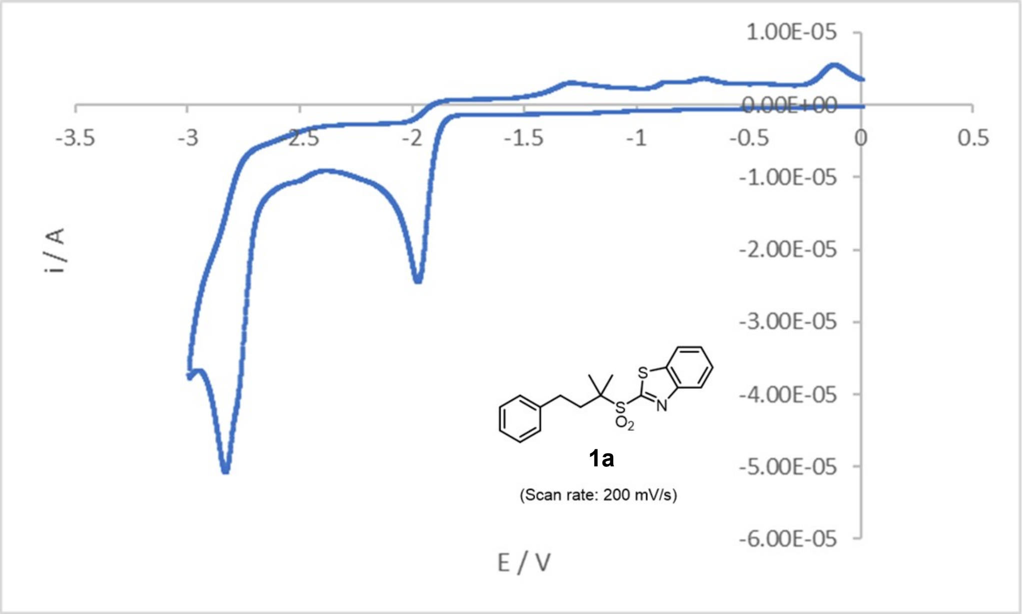

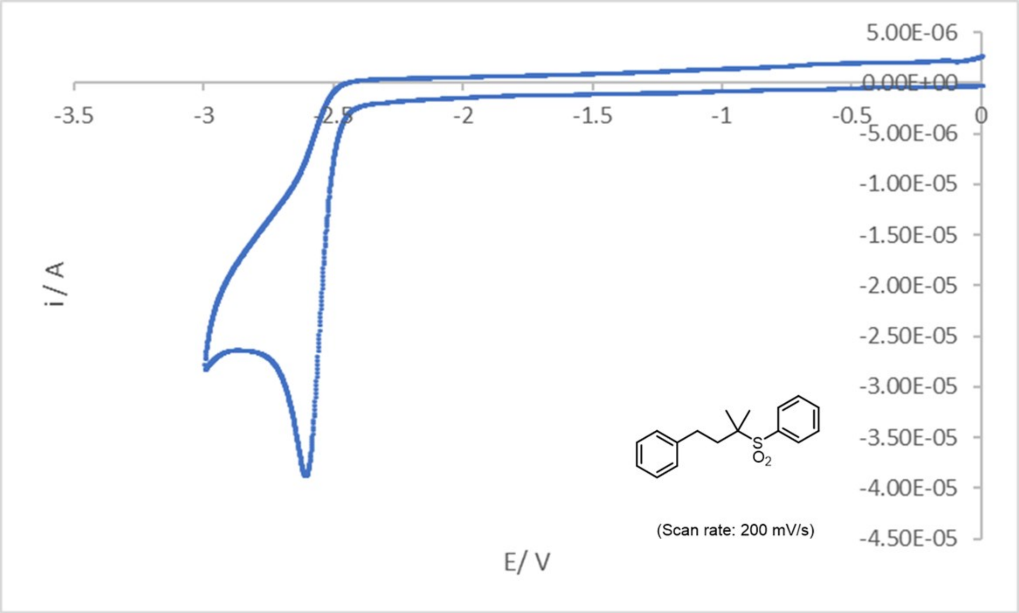
5.00E-06
0.00E+00
-5.00E-06
-1.00E-05
-1.50E-05
-2.00E-05
-2.50E-05
-3.00E-05
-3.50E-05
-4.00E-05
-4.50E-05
-3.5
-3
-2.5
-2
-1.5
-1
-0.5
0
i / A
E/ V
S
O2
(Scan rate: 200 mV/s)

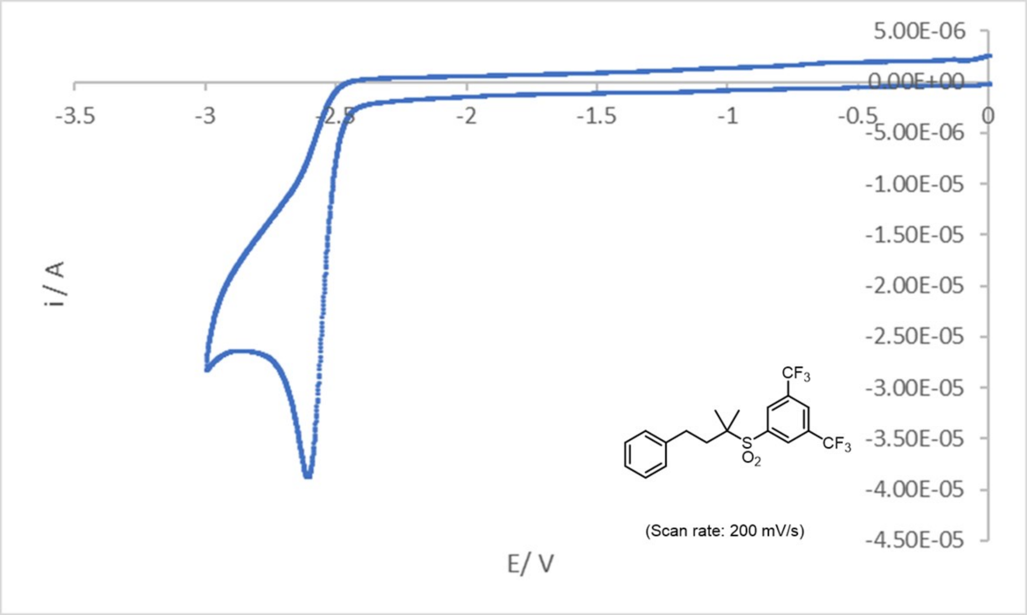
5.00E-06
0.00E+00
-5.00E-06
-1.00E-05
-1.50E-05
-2.00E-05
-2.50E-05
-3.00E-05
-3.50E-05
-4.00E-05
-4.50E-05
-3.5
-3
-2.5
-2
-1.5
-1
-0.5
0
i / A
E/ V
CF3
CF3
S
O2
(Scan rate: 200 mV/s)

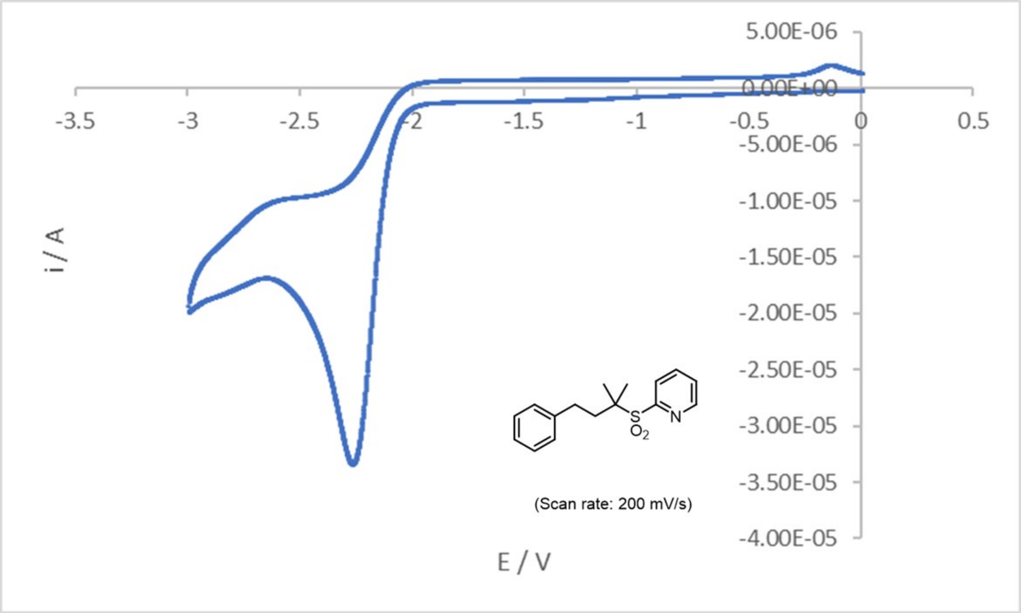

9. Computational methods

All calculations were performed using Gaussian 09. Geometry optimizations and thermal correction were performed at CAM-B3LYP/6-31+G* level. Stationary points were verified by vibrational frequency analysis. Solvation effects of DMF were evaluated with the IEFPCM calculation with radii and non-electrostatic terms for Truhlar and coworkers' SMD solvation model.

By using the optimized geometries, UV-Vis absorption spectra and bond dissociation energies were computed using TD-DFT and SCSMP2, respectively. The basis set for the TD-DFT and SCSMP2 calculations was cc-pVDZ basis set for H, C, S, Zn, and aug-cc-pVDZ for N, O.

10. References

1) Bateman, L. A.; Nguyen, T. B.; Roberts, A. M.; Miyamoto, D. K.; Ku, W. M.; Huffman, T. R.; Petri, Y.; Heslin, M. J.; Contreras, C. M.; Skibola, C. F.; Olzmann, J. A.; Nomura, D. K., *Chem. Commun.* **2017,** *53*, 7234-7237.

2) Uno, M.; Sumino, S.; Fukuyama, T.; Matsuura, M.; Kuroki, Y.; Kishikawa, Y.; Ryu, I., *J. Org. Chem.* **2019,** *84*, 9330-9338.

3) Denmark, S. E.; Cresswell A. J. *J. Org. Chem.* **2013**, *78*, 12593–12628.

4) Rodrigo, E.; Alonso, I.; Cid, M. B. *Org. Lett.* **2018**, *20*, 5789-5793.

5) Legarda, P. D.; Garcia-Rubia, A.; Arrayás, R. G.; Carretero, J. C. *Adv. Synth. Catal.* **2016**, *358*, 1065-1072.

6) Merchant, R. R.; Edwards, J. T.; Qin, T.; Kruszyk, M. M.; Bi, C.; Che, G. D.; Bao, D. H.; Qiao, W. H.; Sun, L. J.; Collins, M. R.; Fadeyi, O. O.; Gallego, G. M.; Mousseau, J. J.; Nuhant, P.; Baran, P. S. *Science* **2018,** *360*, 75-80.

7) Dussart, N.; Trinh, H. V.; Gueyrard, D. *Org. Lett.* **2016,** *18*, 4790-4793.

8) Sedrani, R.; Kallen, J.; Martin Cabrejas, L. M.; Papageorgiou, C. D.; Senia, F.; Rohrbach, S.; Wagner, D.; Thai, B.; Jutzi Eme, A. M.; France, J.; Oberer, L.; Rihs, G.; Zenke, G.; Wagner, J. *J. Am. Chem. Soc.* **2003,** *125*, 3849-59.

9) Hughes, J. M. E.; Fier, P. S. *Org. Lett.* **2019,** *21*, 5650-5654.

10) Sanhueza, L.; Cortes-Arriagada, D.; Ledoux-Rak, I.; Crivelli, I.; Loeb, B. *Synthetic Met.* **2017,** *234*, 9-17.

11) Qin, T.; Malins, L. R.; Edwards, J. T.; Merchant, R. R.; Novak, A. J.; Zhong, J. Z.; Mills, R. B.; Yan, M.; Yuan, C.; Eastgate, M. D.; Baran, P. S. *Angew. Chem. Int. Ed.* **2017,** *56*, 260-265.

12) Nawrat, C. C.; Jamison, C. R.; Slutskyy, Y.; MacMillan, D. W. C.; Overman, L. E. *J. Am. Chem. Soc.* **2015,** *137*, 11270-11273.

13) Wu, X.; Hao, W.; Ye, K. Y.; Jiang, B.; Pombar, G.; Song, Z.; Lin, S. *J. Am. Chem. Soc.* **2018,** *140*, 14836-14843.

14) Chinzei, T.; Miyazawa, K.; Yasu, Y.; Koike, T.; Akita, M. *RSC Adv.*, **2015**, *5*, 21297-21300.

15) Han, Han, N.; Johns, B. A.; Tang, J., Patent WO 2013020245, Feb. 14, 2013.

11. ^{1}H, ^{13}C and ^{19}F NMR Spectra of Products

^{1}H-NMR (400 MHz, $CDCl_3$) of 3,5-bis(trifluoromethyl)phenyl 1-(3-phenylpropyl) sulfone

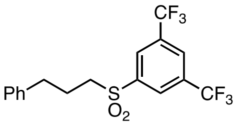

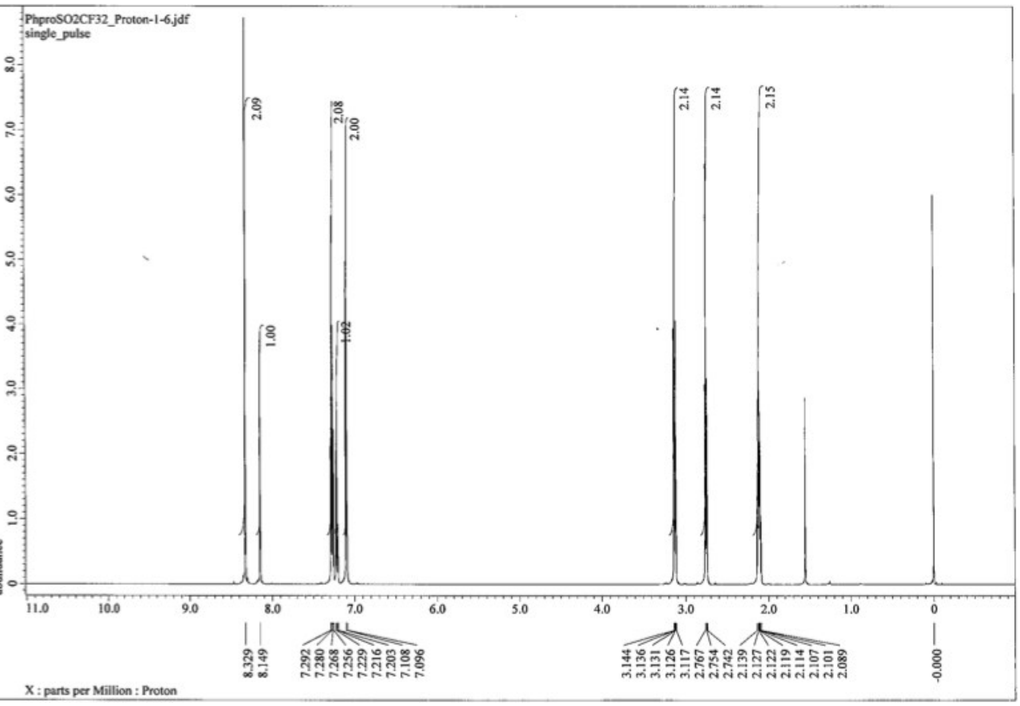

^{13}C-NMR (150 MHz, $CDCl_3$) of 3,5-bis(trifluoromethyl)phenyl 1-(3-phenylpropyl) sulfone

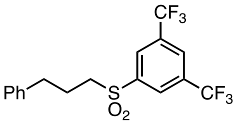

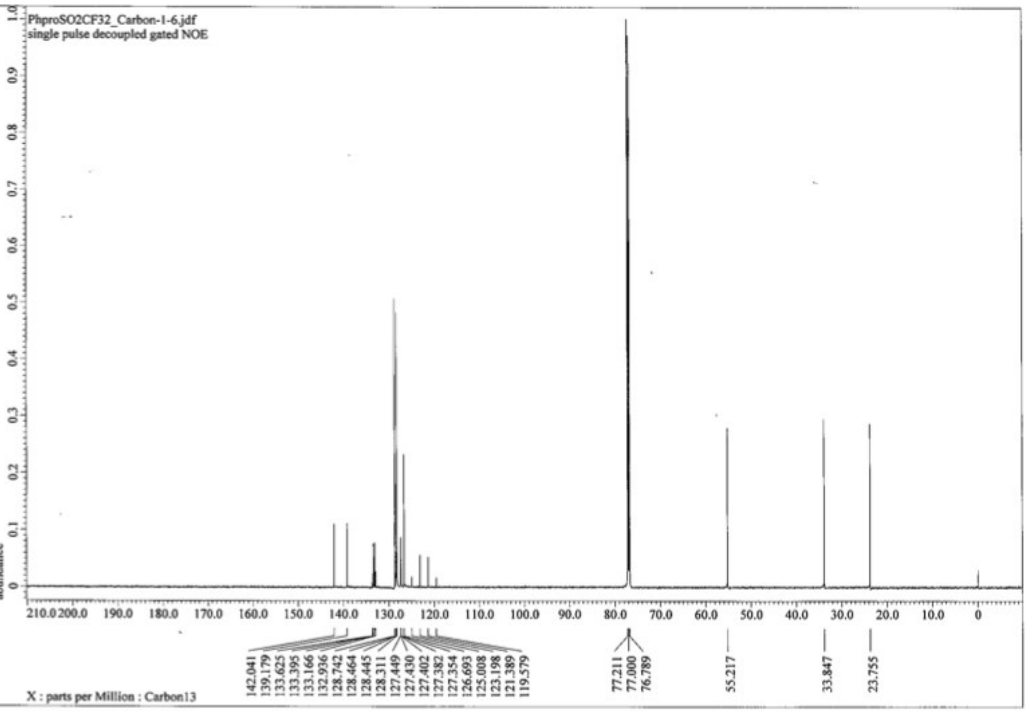

^{19}F-NMR (376 MHz, $CDCl_3$) of 3,5-bis(trifluoromethyl)phenyl 1-(3-phenylpropyl) sulfone

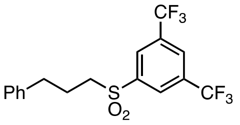

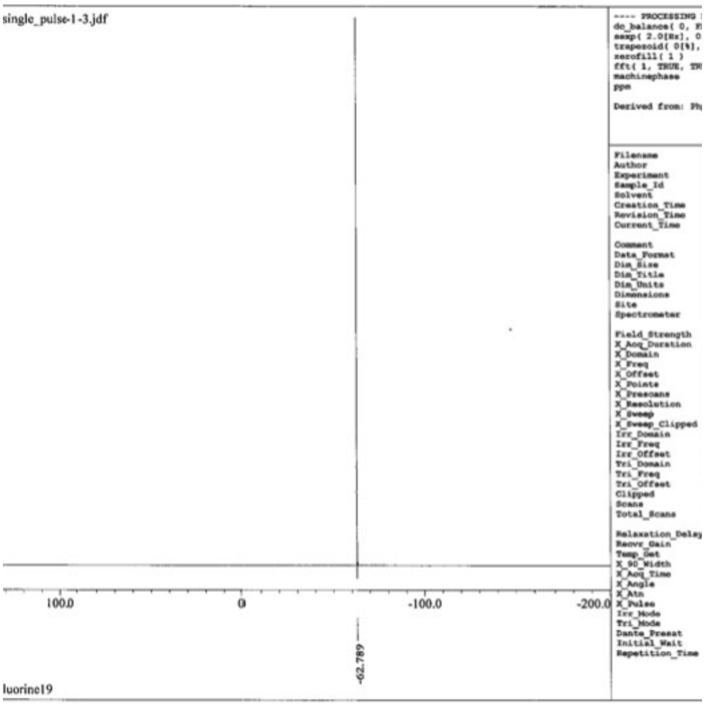

^{1}H-NMR (600 MHz, $CDCl_3$) of 5-((4-phenylbutan-2-yl)sulfonyl)-1-phenyl-1*H*-tetrazole

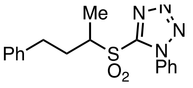

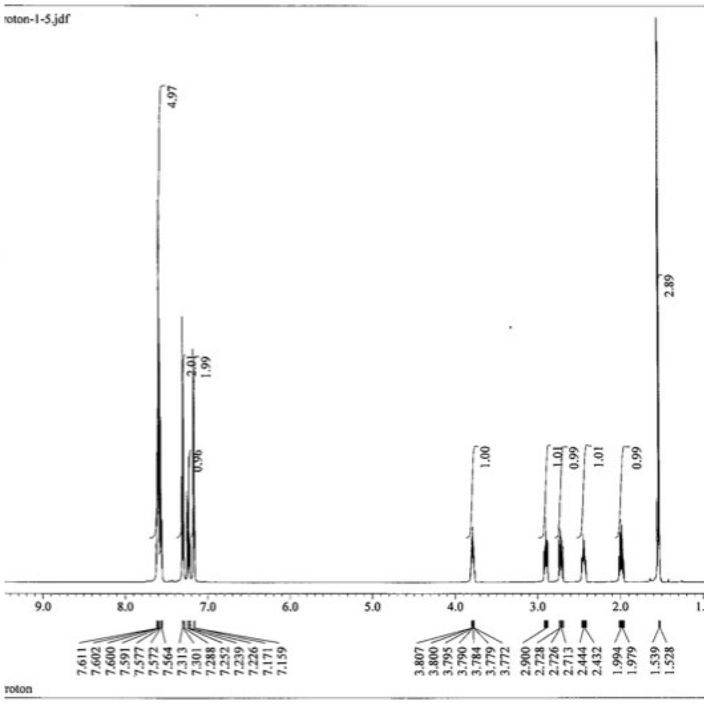

^{13}C-NMR (150 MHz, $CDCl_3$) of 5-((4-phenylbutan-2-yl)sulfonyl)-1-phenyl-1*H*-tetrazole

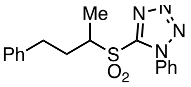

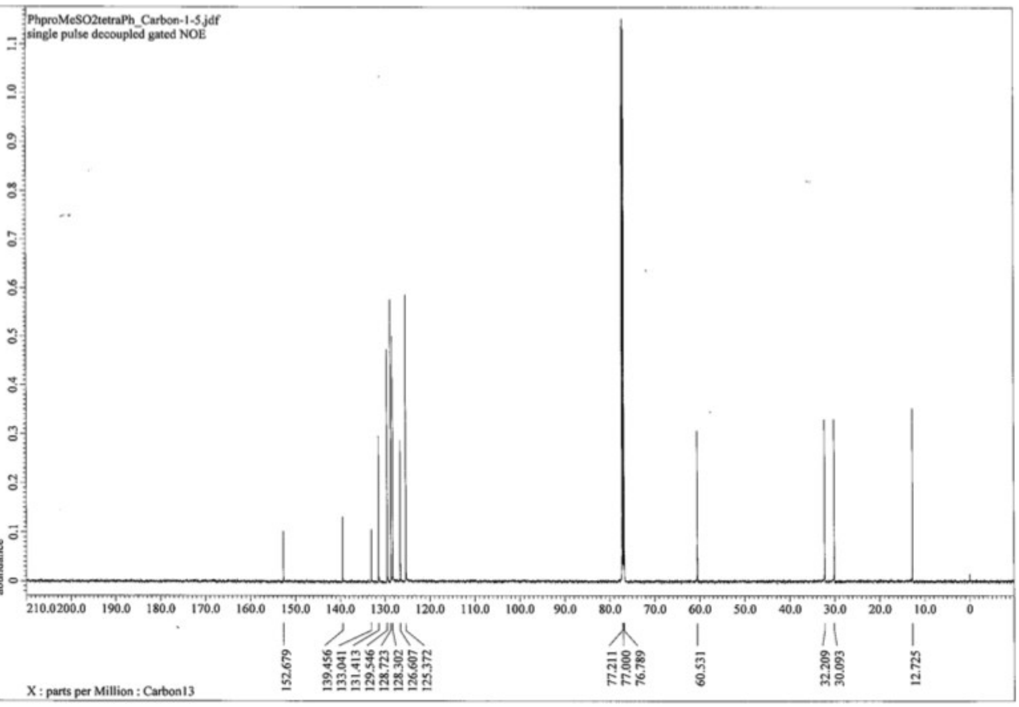

^{1}H-NMR (600 MHz, $CDCl_3$) of 1a

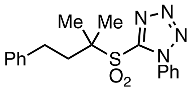

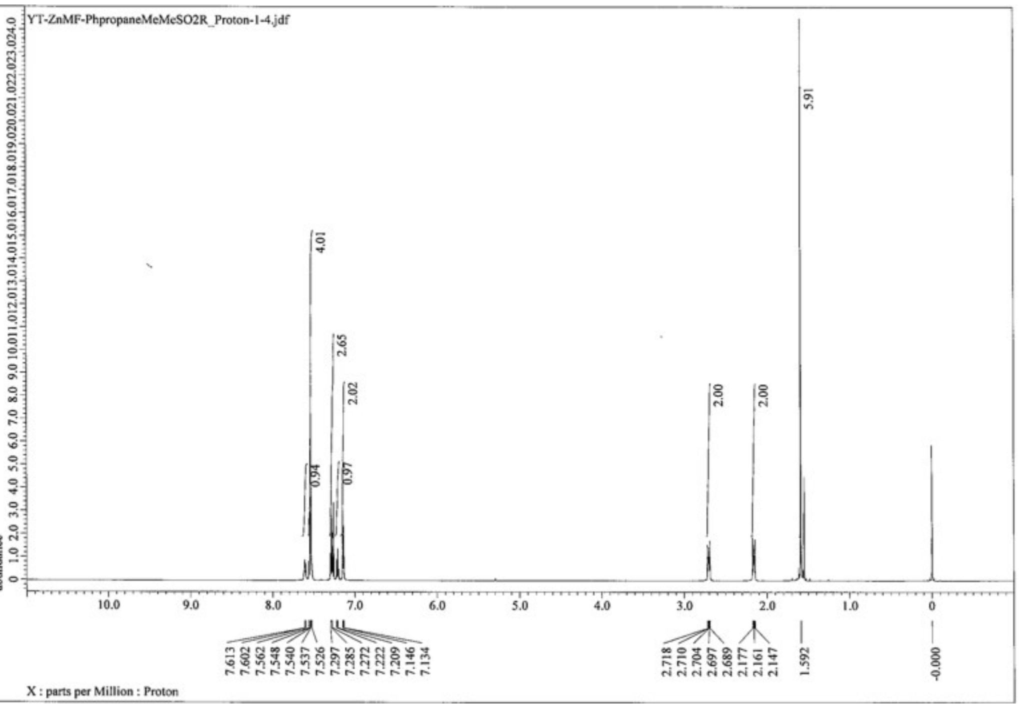
YT-ZnMF-PhpropaneMeMeSO2R_Proton-1-4.jdf
4.01
2.65
2.02
0.94
0.97
2.00
2.00
5.91
10.0 9.0 8.0 7.0 6.0 5.0 4.0 3.0 2.0 1.0 0
7.613 7.602 7.562 7.548 7.540 7.537 7.526 7.297 7.285 7.272 7.222 7.209 7.146 7.134
2.718 2.710 2.704 2.697 2.689 2.177 2.161 2.147
1.592
-0.000
X : parts per Million : Proton

^{13}C-NMR (150 MHz, CDCl$_3$) of 1a

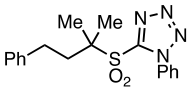

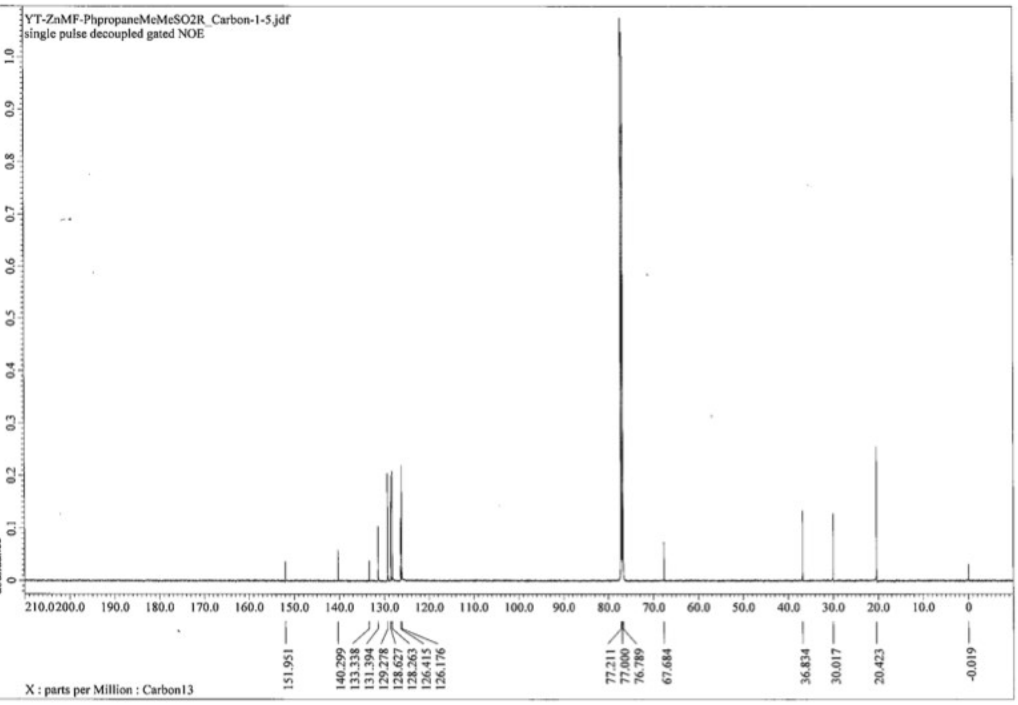

^{1}H-NMR (600 MHz, CDCl$_3$) of 1b

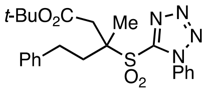

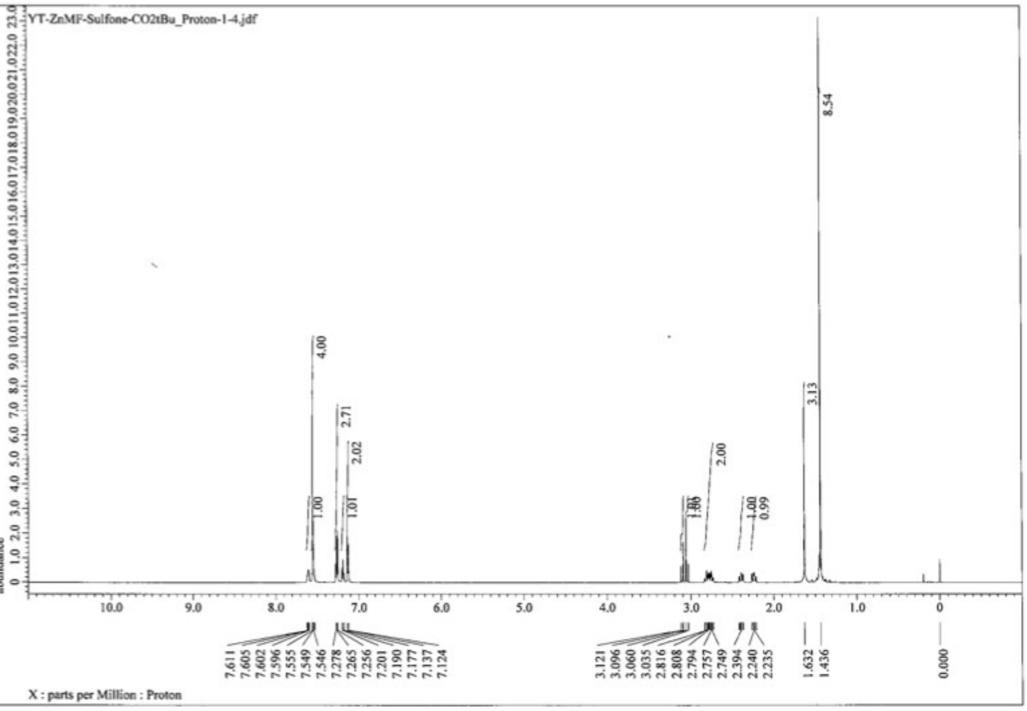

^{13}C-NMR (150 MHz, $CDCl_3$) of 1b

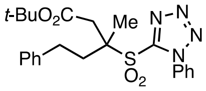

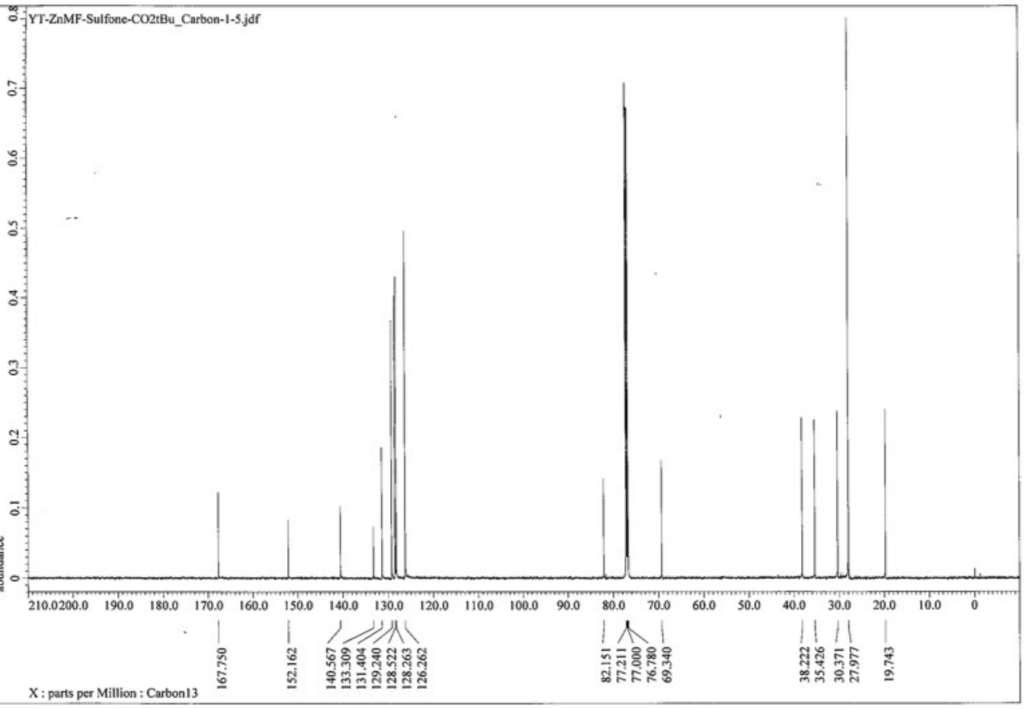

^{1}H-NMR (500 MHz, $CDCl_3$) of 1c

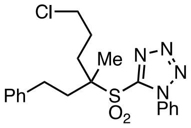

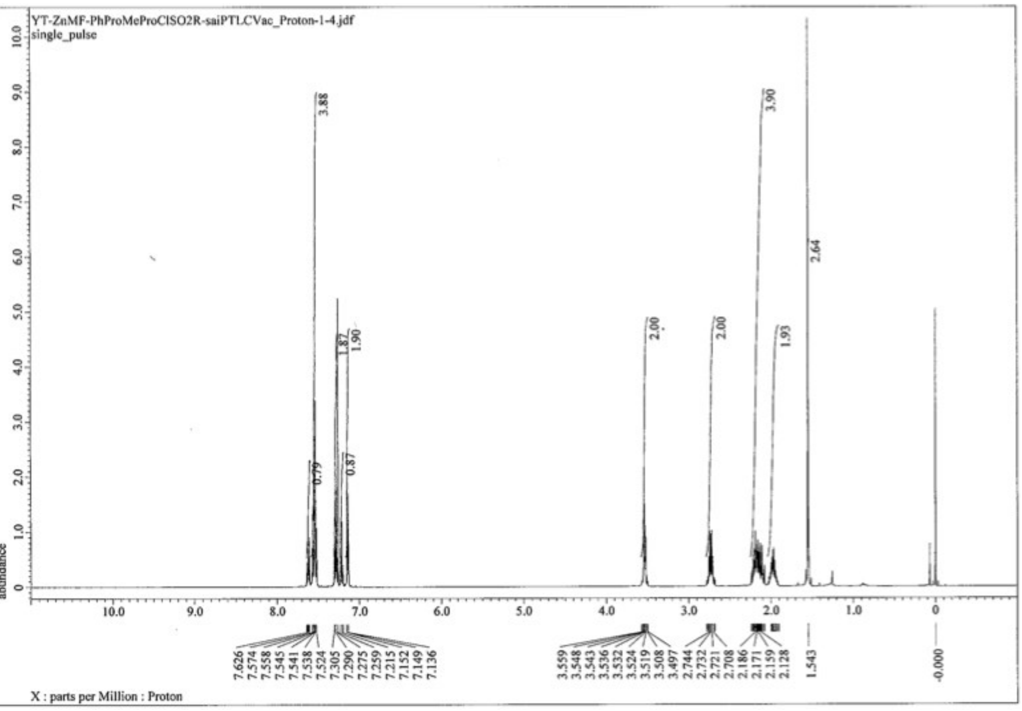

^{13}C-NMR (125 MHz, $CDCl_3$) of 1c

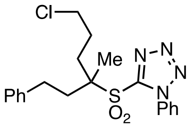

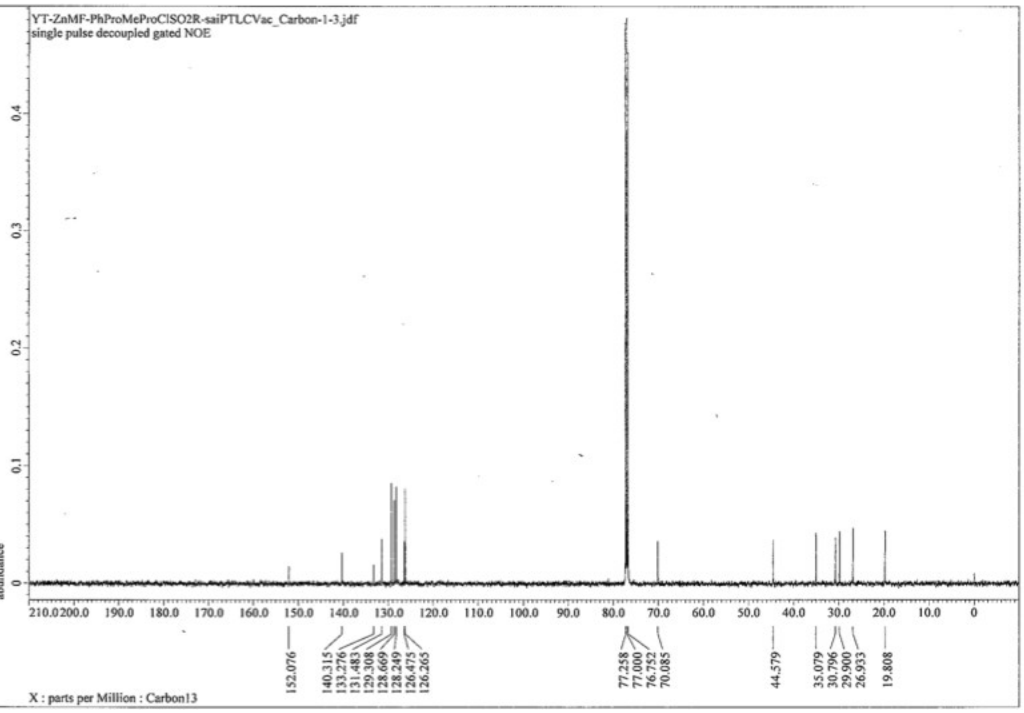

^{1}H-NMR (600 MHz, $CDCl_3$) of 1d

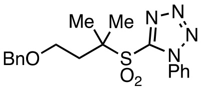

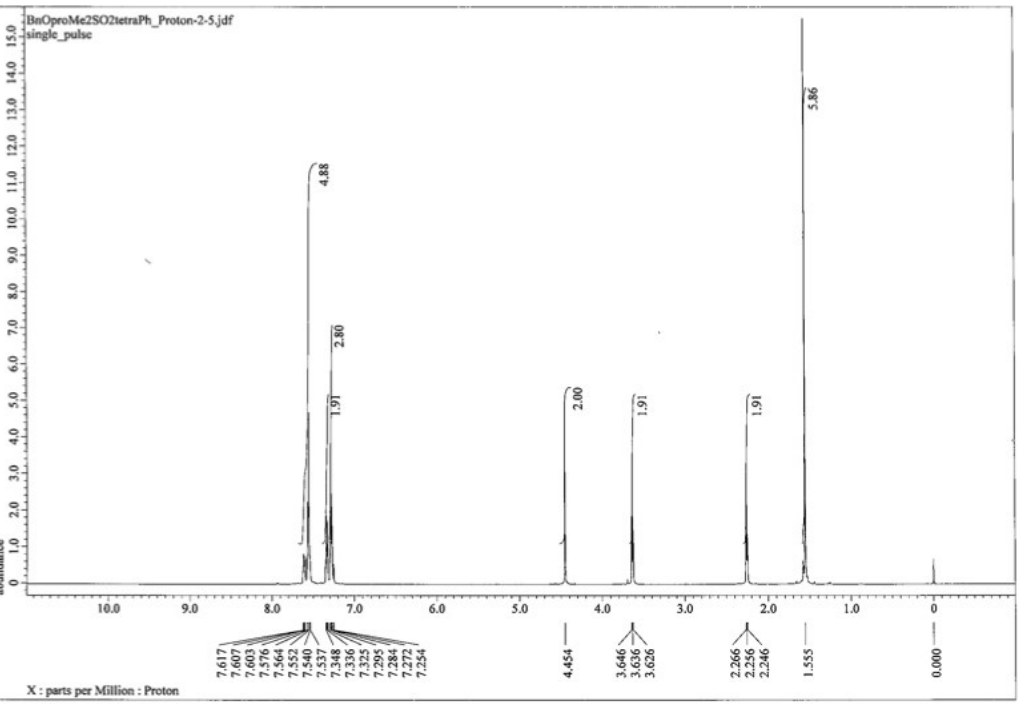
BnOproMe2SO2tetraPh_Proton-2-5.jdf
single_pulse
X : parts per Million : Proton
4.88
2.80
1.91
2.00
1.91
1.91
5.86
7.617
7.607
7.603
7.576
7.564
7.552
7.540
7.537
7.348
7.336
7.325
7.295
7.284
7.272
7.254
4.454
3.646
3.636
3.626
2.266
2.256
2.246
1.555
0.000

^{13}C-NMR (150 MHz, $CDCl_3$) of 1d

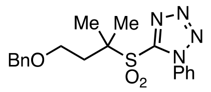

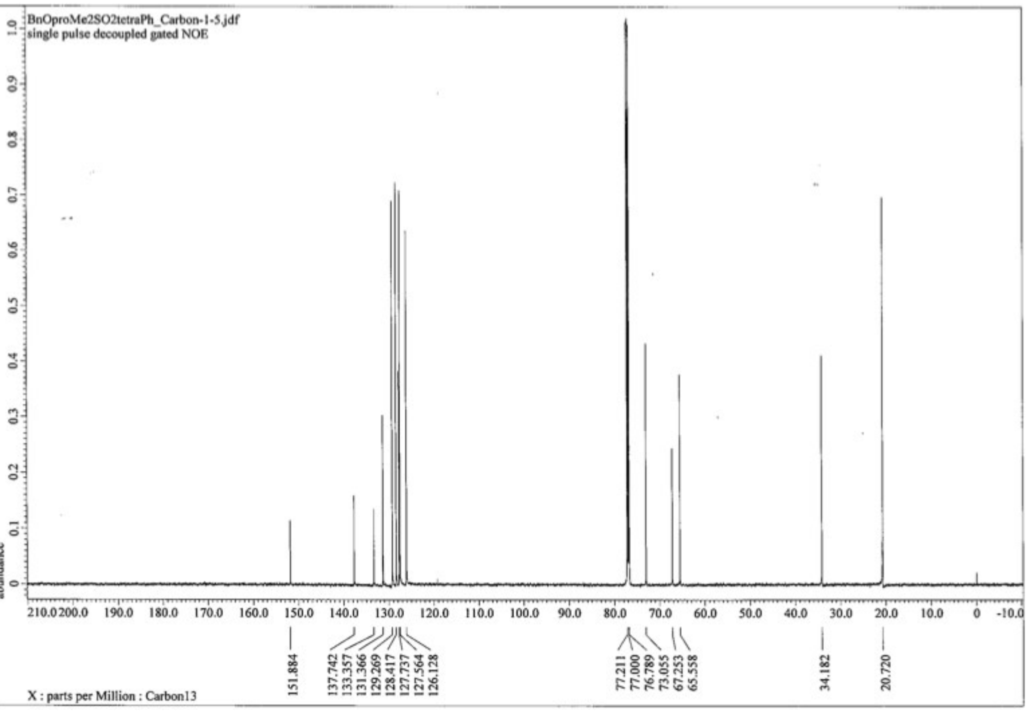

^{1}H-NMR (600 MHz, $CDCl_3$) of 1e

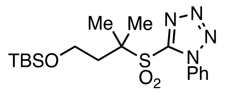

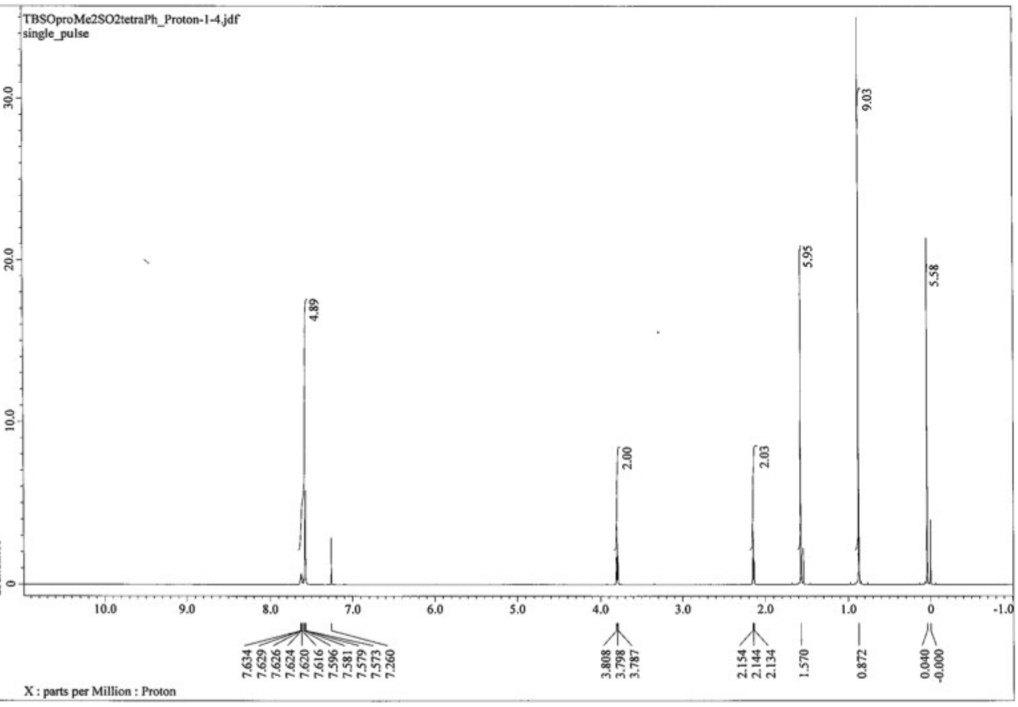
TBSOproMe2SO2tetraPh_Proton-1-4.jdf
single_pulse
4.89
2.00
2.03
5.95
9.03
5.58
7.634
7.629
7.626
7.624
7.620
7.616
7.596
7.581
7.579
7.573
7.260
3.808
3.798
3.787
2.154
2.144
2.134
1.570
0.872
0.040
-0.000
X : parts per Million : Proton

^{13}C-NMR (150 MHz, CDCl$_3$) of 1e

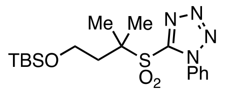

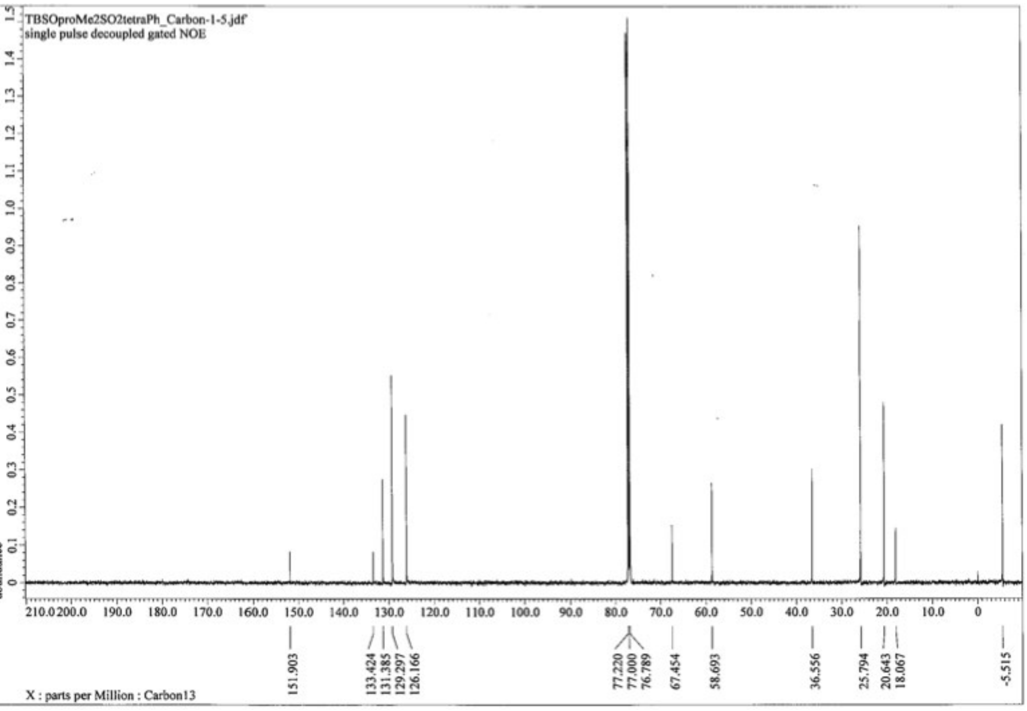

^{1}H-NMR (600 MHz, CDCl$_3$) of 5-((1',1'-dimethyl-3'-hydroxypropyl)sulfonyl)-1-phenyl-*1H*-tetrazole

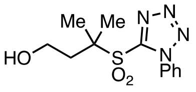

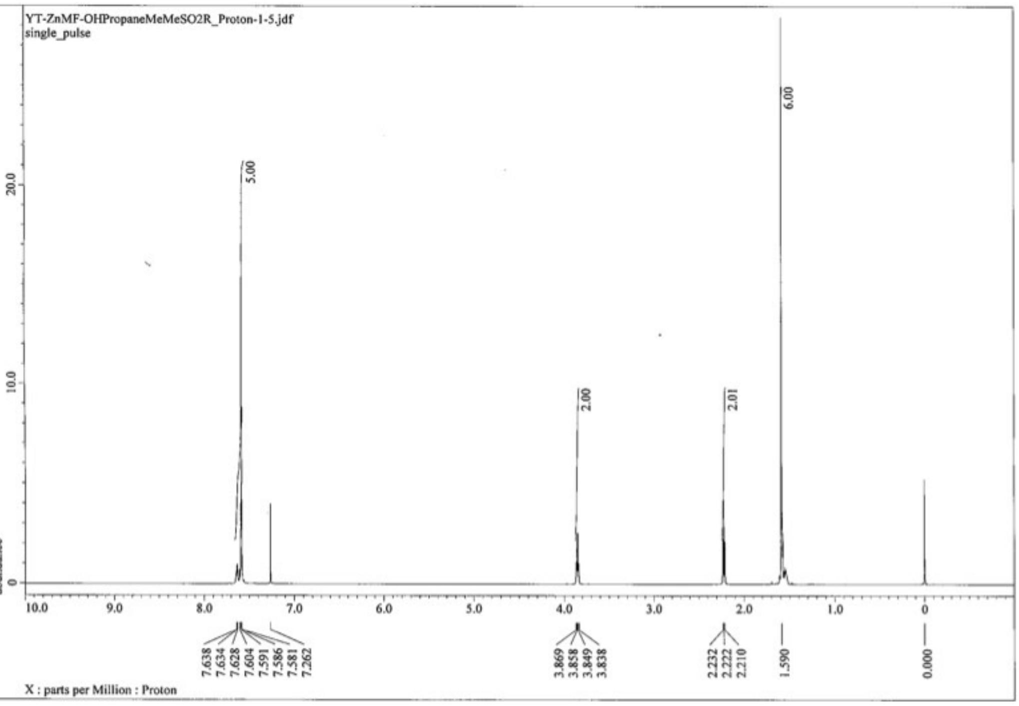

^{13}C-NMR (150 MHz, $CDCl_3$) of 5-((1’,1’-dimethyl-3’-hydroxypropyl)sulfonyl)-1-phenyl-*1H*-tetrazole

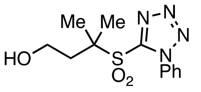

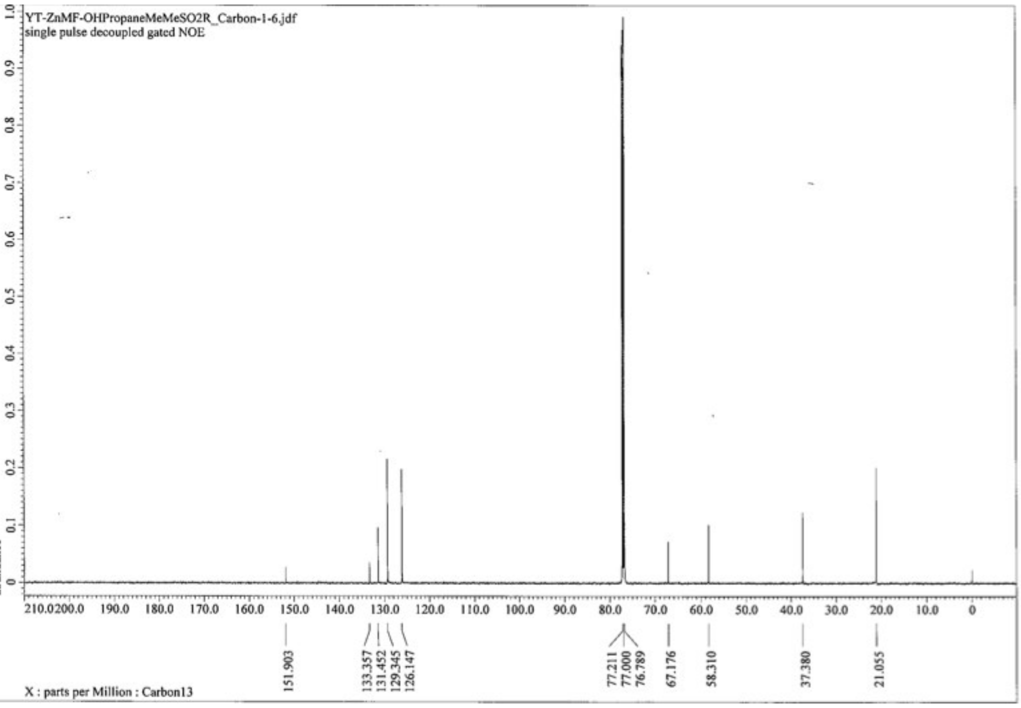

^{1}H-NMR (600 MHz, $CDCl_3$) of 1f

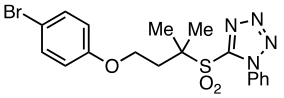

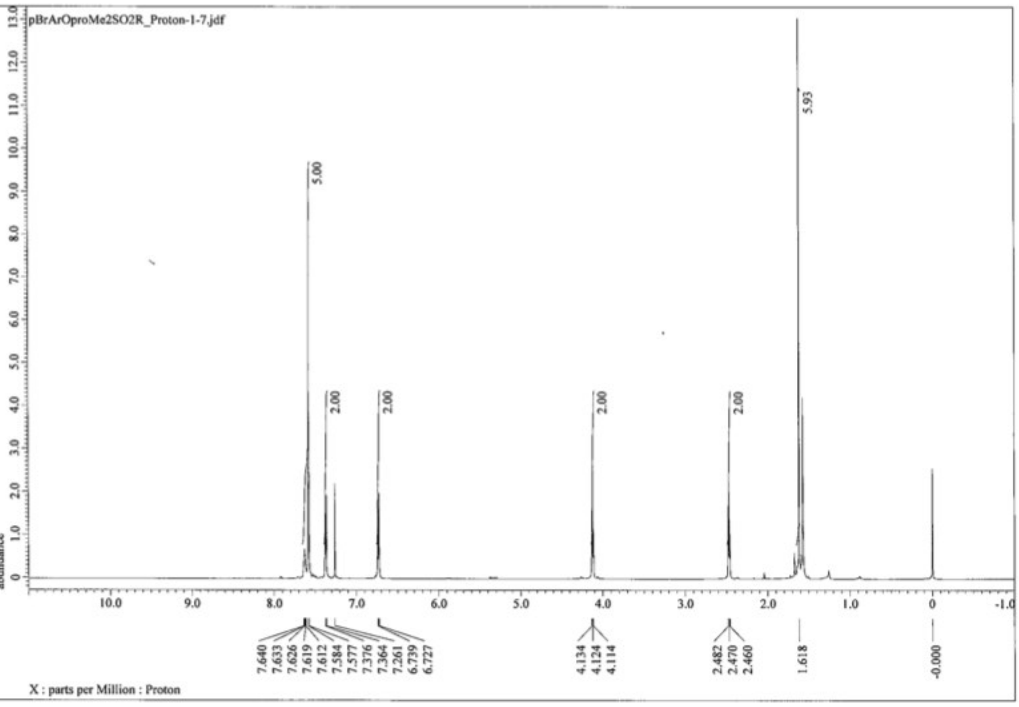

^{13}C-NMR (150 MHz, $CDCl_3$) of 1f

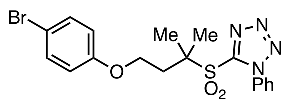

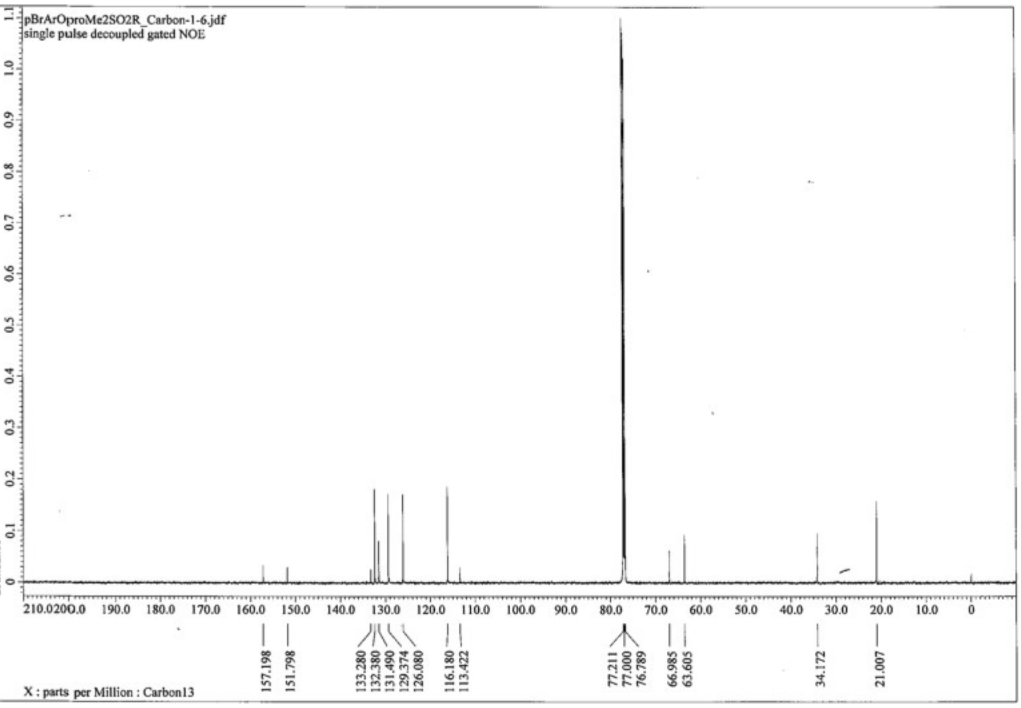

^{1}H-NMR (600 MHz, $CDCl_3$) of 1g

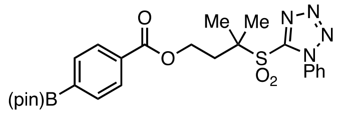

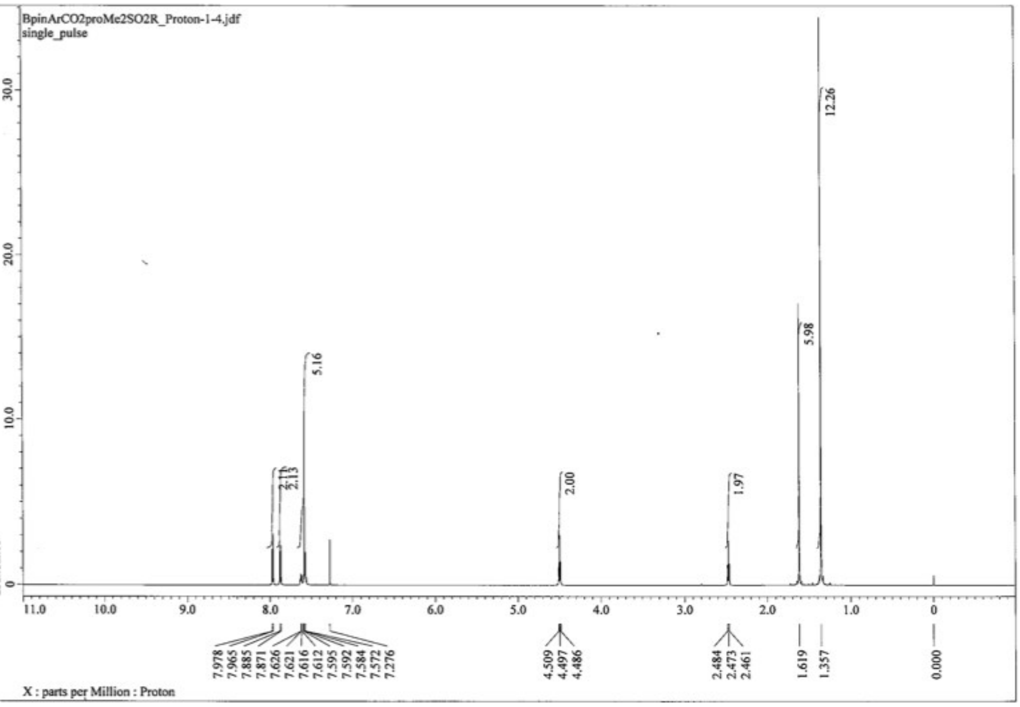

BpinArCO2proMe2SO2R_Proton-1-4.jdf
single_pulse
X : parts per Million : Proton

^{13}C-NMR (150 MHz, $CDCl_3$) of 1g

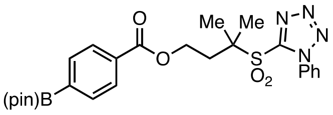

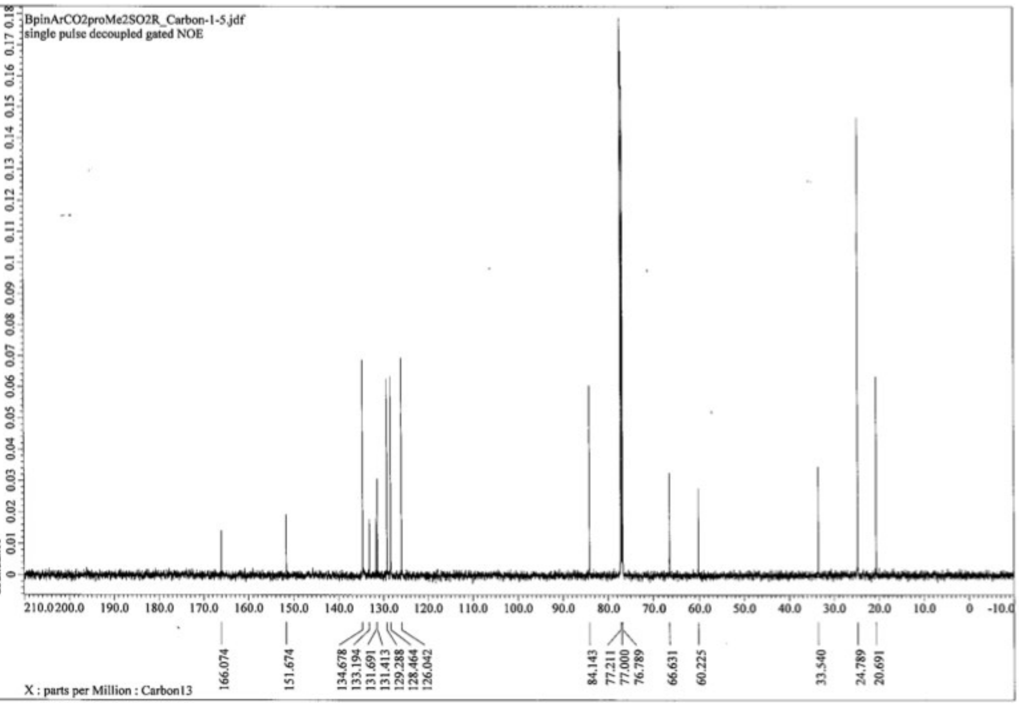

^{1}H-NMR (600 MHz, $CDCl_3$) of 1h

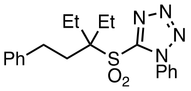

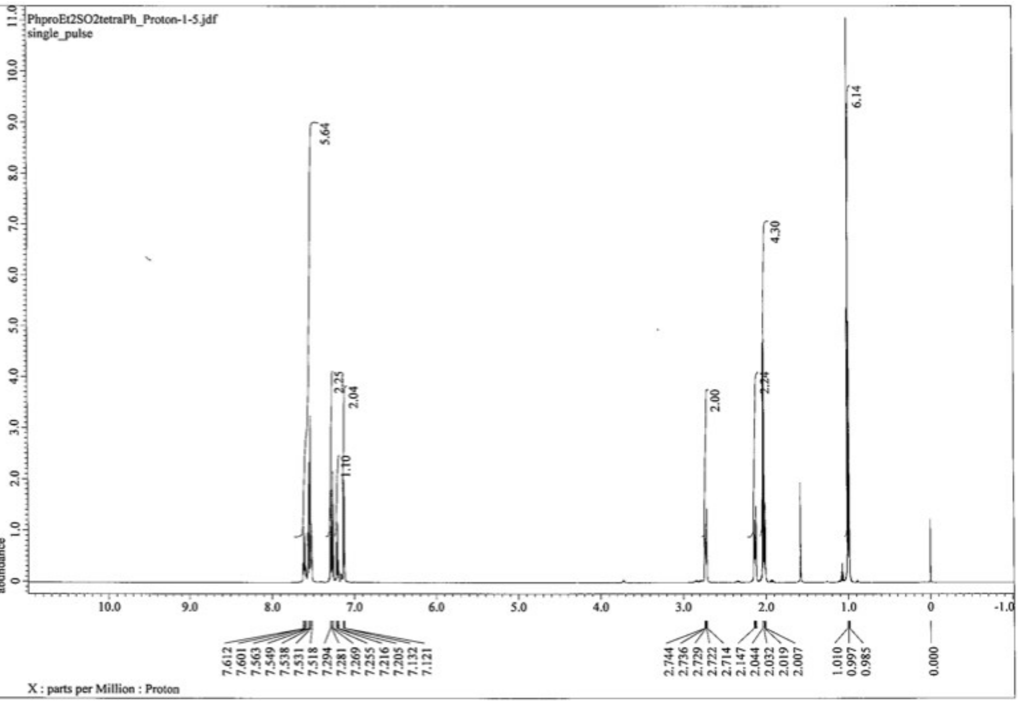

^{13}C-NMR (150 MHz, CDCl$_3$) of 1h

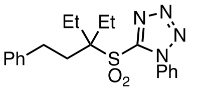

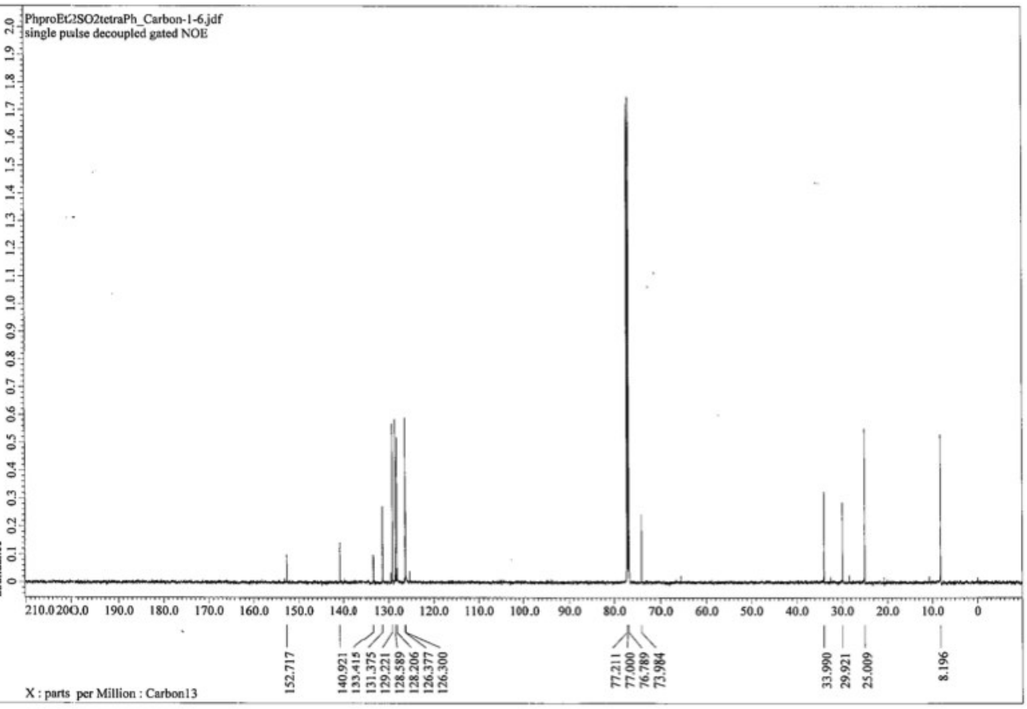

^{1}H-NMR (600 MHz, $CDCl_3$) of 1i

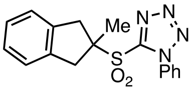

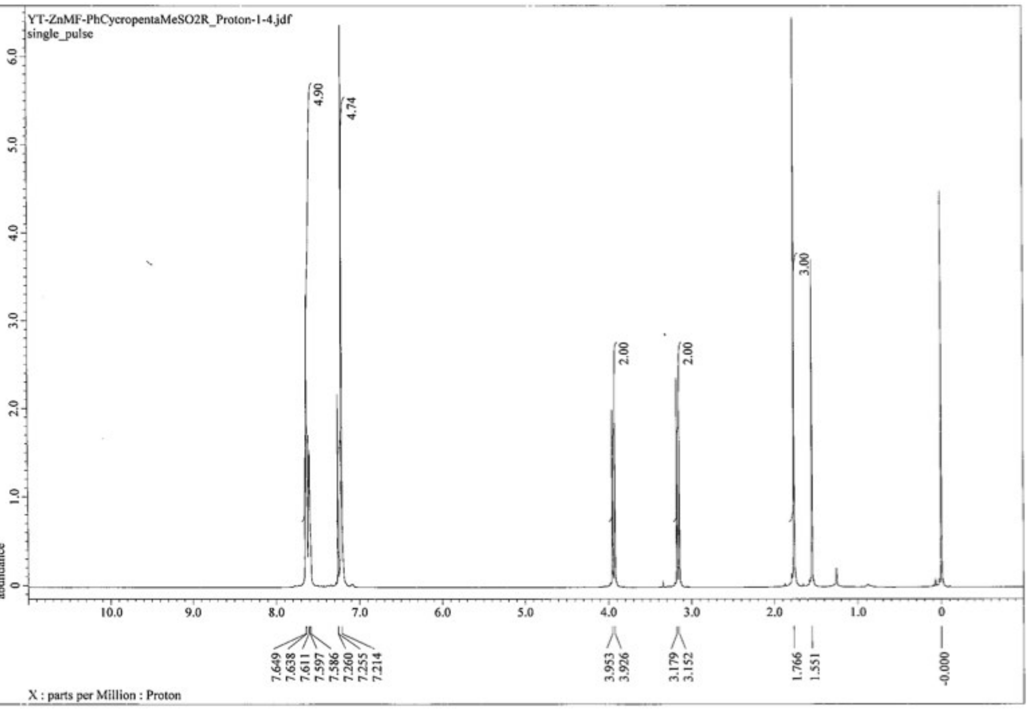

^{13}C-NMR (150 MHz, CDCl$_3$) of 1i

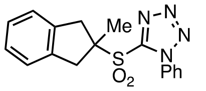

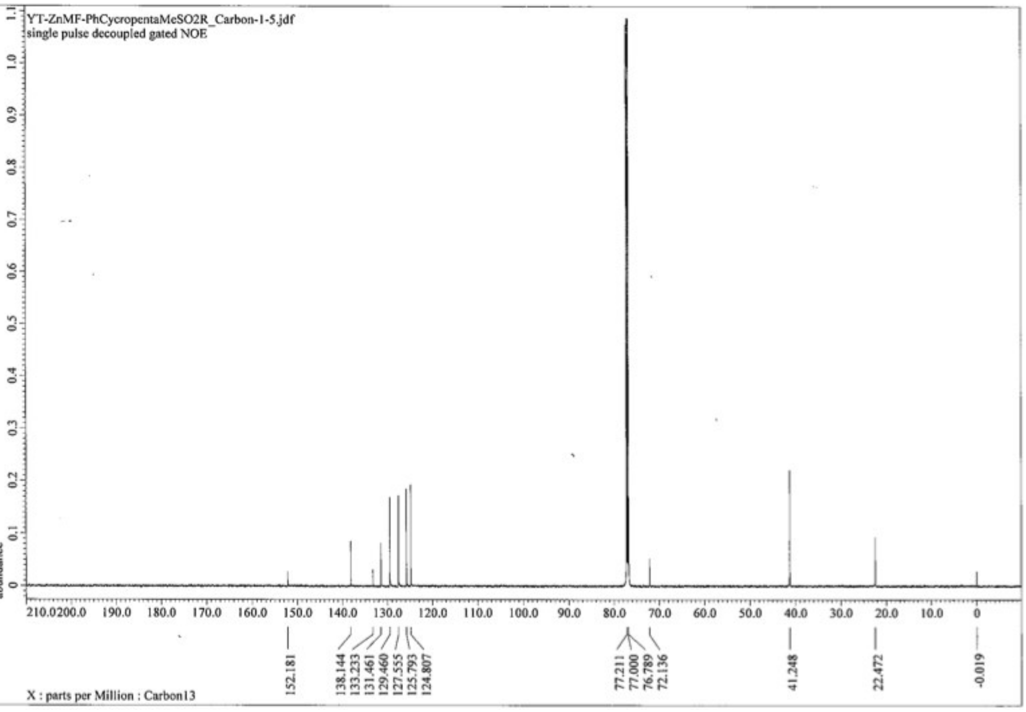

^{1}H-NMR (600 MHz, $CDCl_3$) of 1j

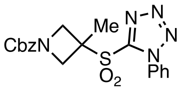

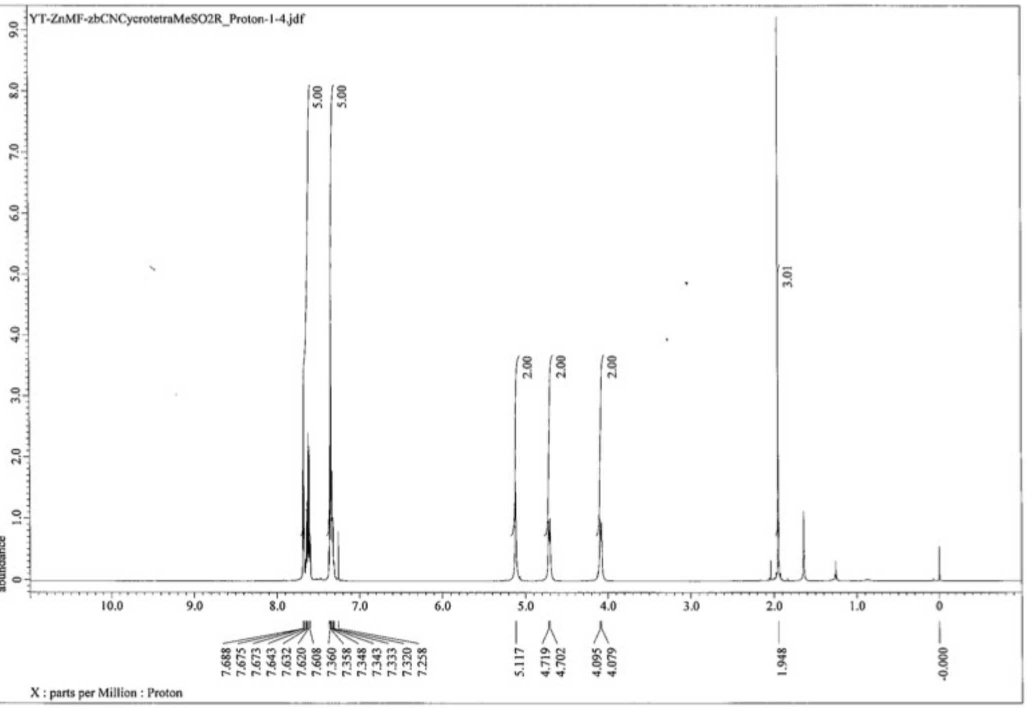

YT-ZnMF-zbCNCyrotetraMeSO2R_Proton-1-4.jdf
5.00
5.00
2.00
2.00
2.00
3.01
7.688
7.675
7.673
7.643
7.632
7.620
7.608
7.360
7.358
7.348
7.343
7.333
7.320
7.258
5.117
4.719
4.702
4.095
4.079
1.948
-0.000
X : parts per Million : Proton
abundance

^{13}C-NMR (150 MHz, $CDCl_3$) of 1j

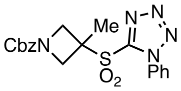

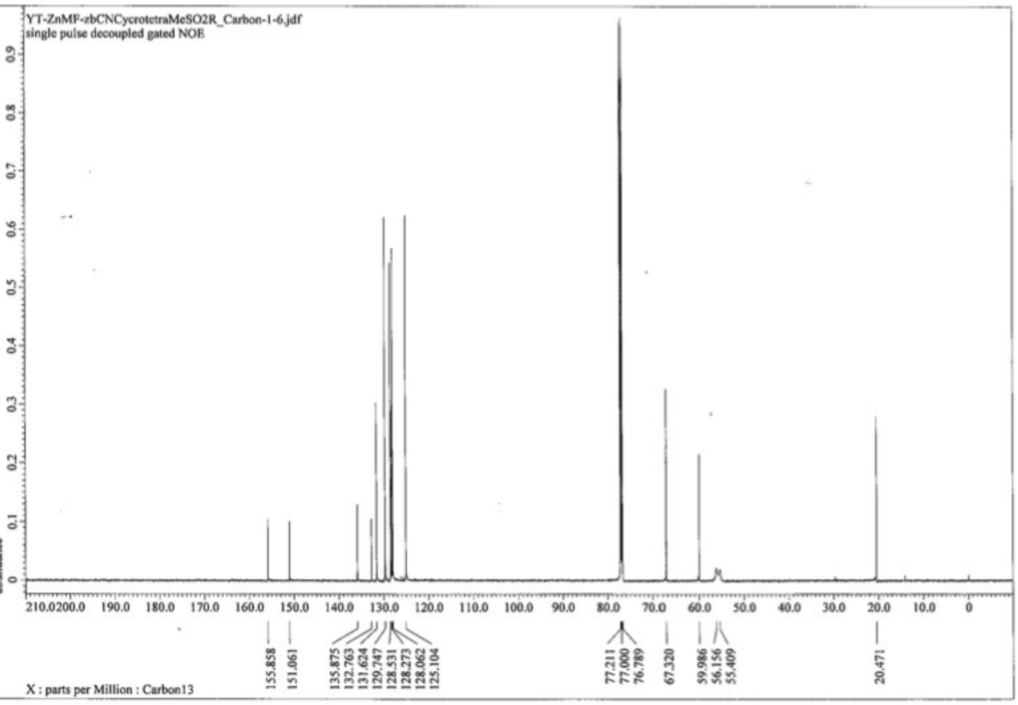

^{1}H-NMR (400 MHz, $CDCl_3$) of 1k

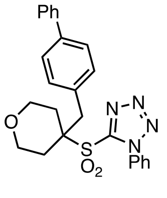

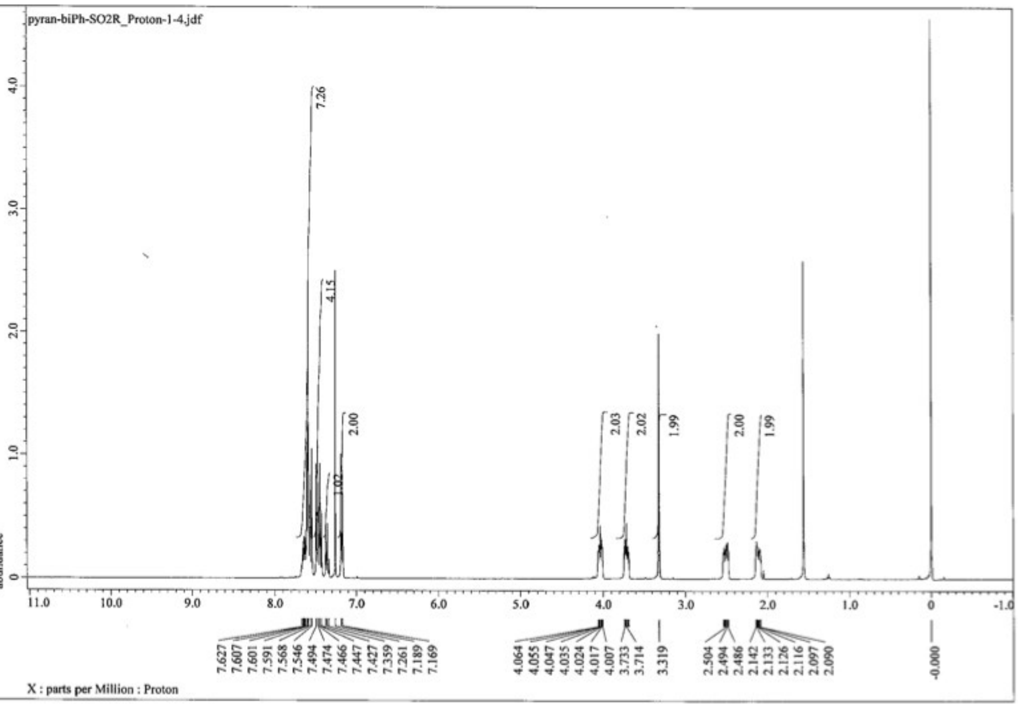

^{13}C-NMR (150 MHz, $CDCl_3$) of 1k

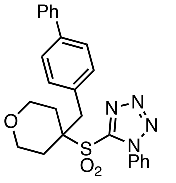

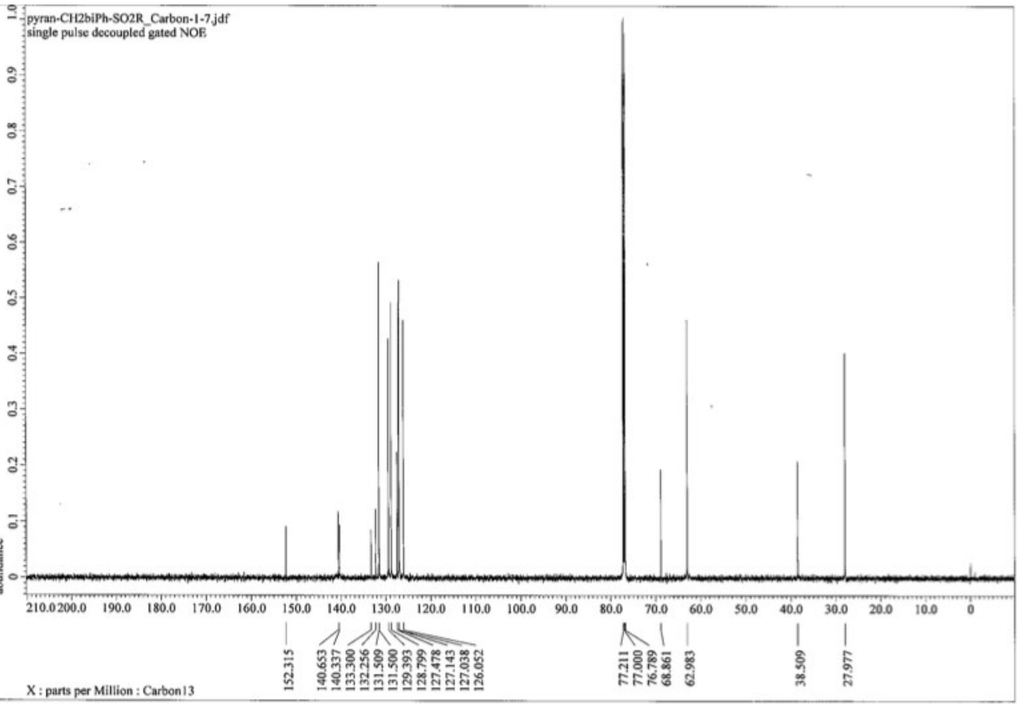

^{1}H-NMR (600 MHz, $CDCl_3$) of 1l

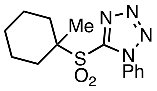

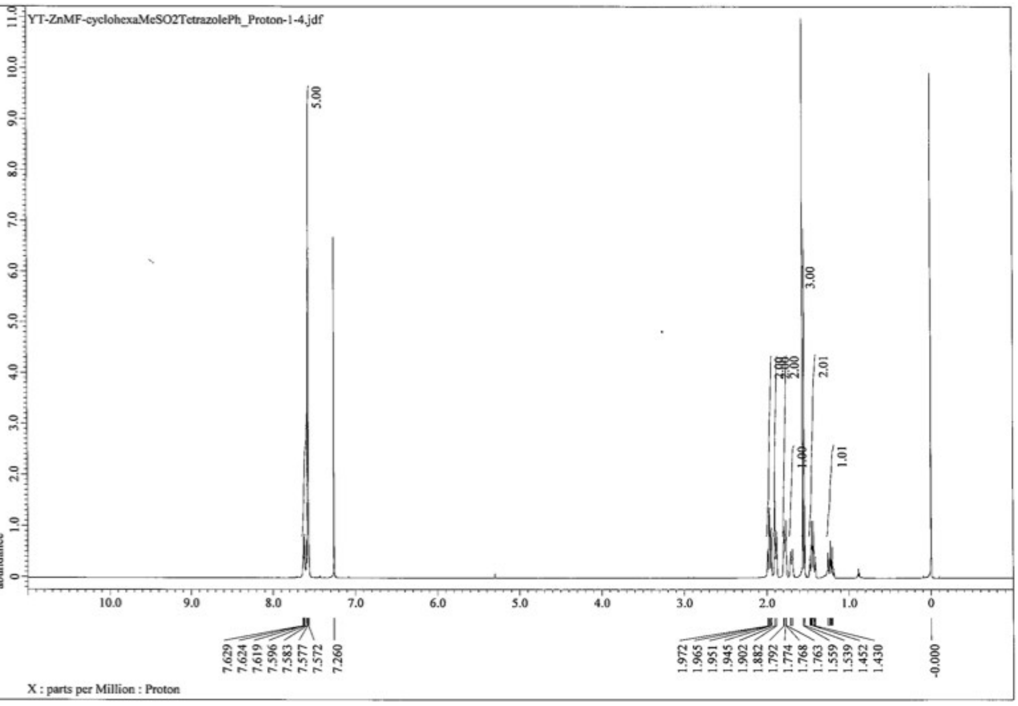

^{13}C-NMR (150 MHz, $CDCl_3$) of 1l

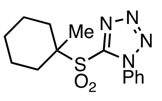

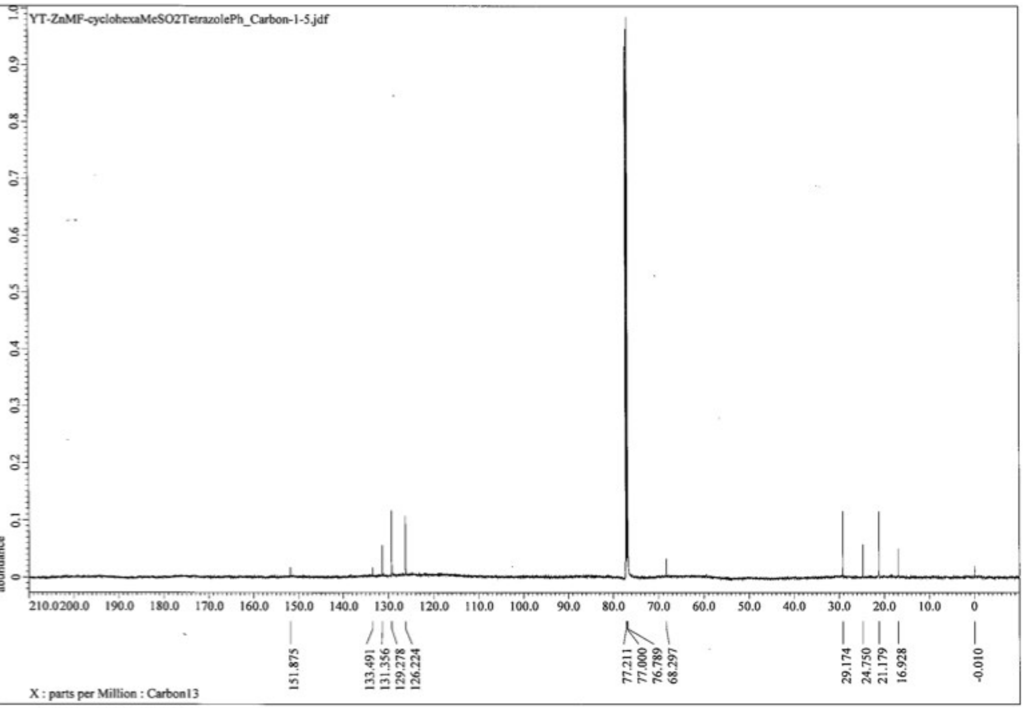

^{1}H-NMR (600 MHz, $CDCl_3$) of 4a

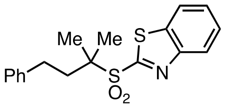

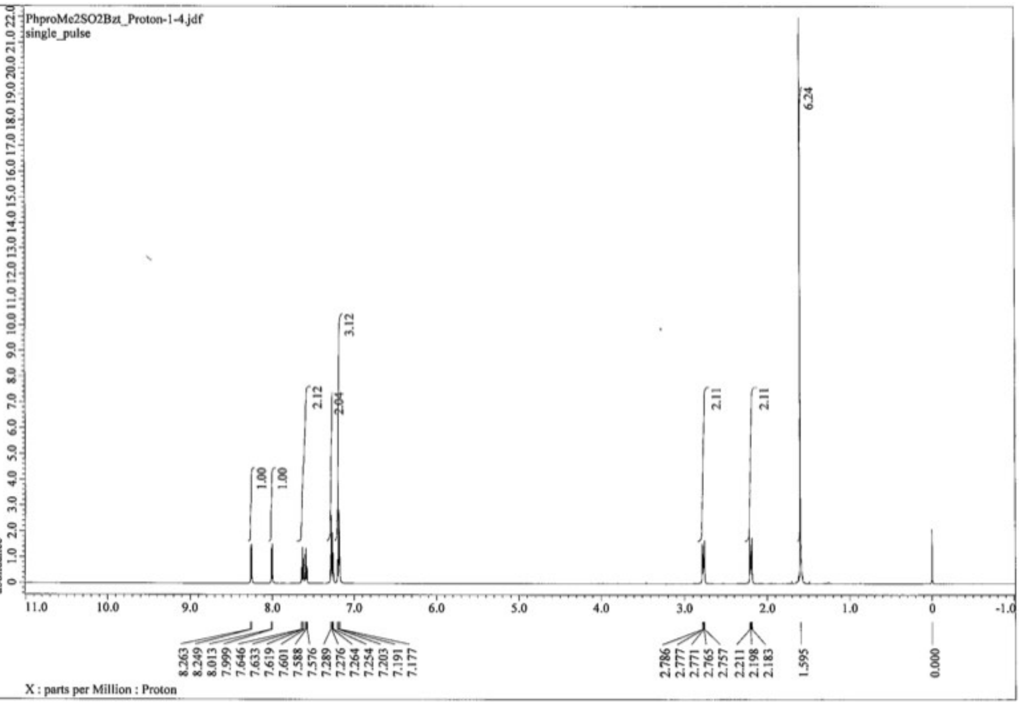

^{13}C-NMR (150 MHz, CDCl$_3$) of 4a

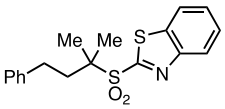

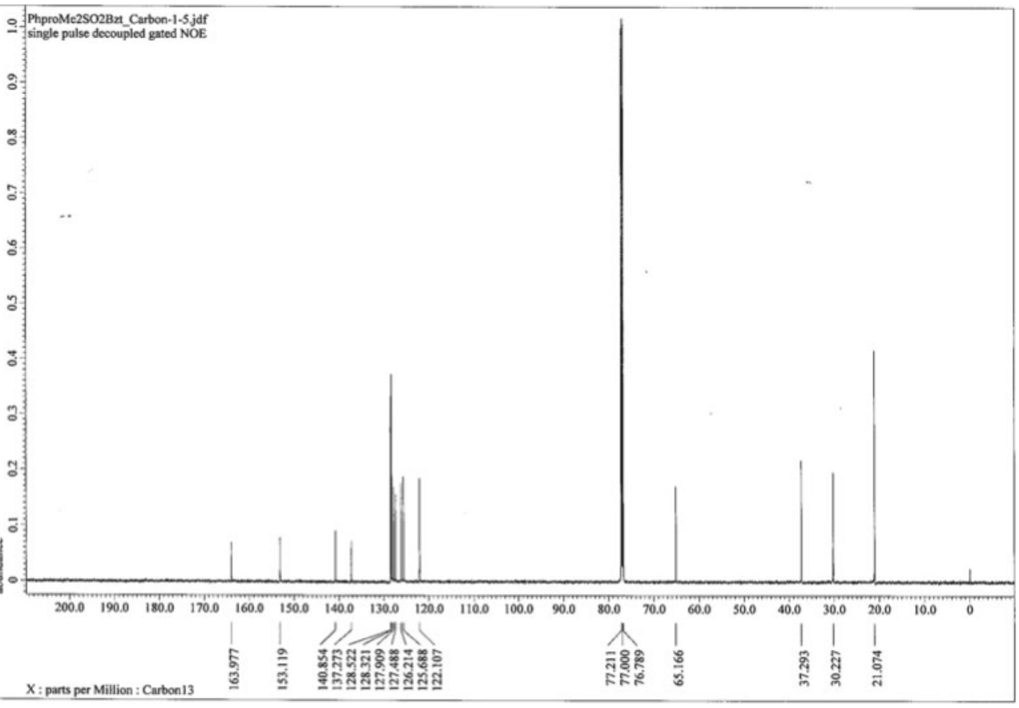

^{1}H-NMR (600 MHz, CDCl$_3$) of 6a

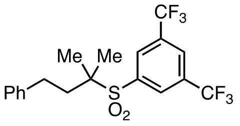

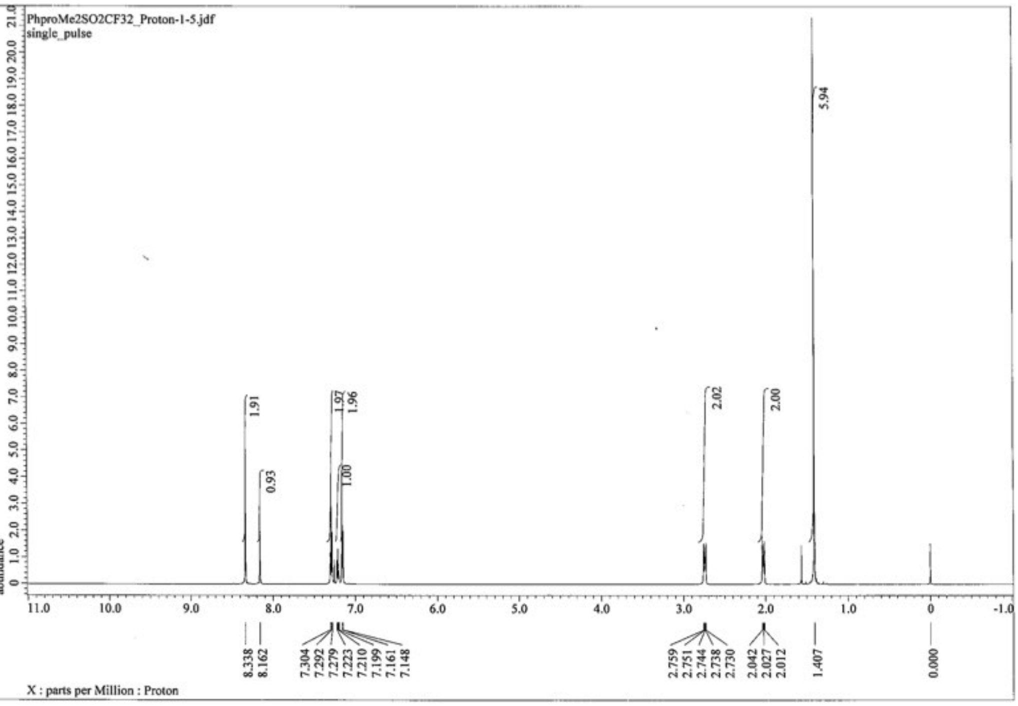
PhproMe2SO2CF32_Proton-1-5.jdf
single_pulse
X : parts per Million : Proton
8.338
8.162
7.304
7.292
7.279
7.223
7.210
7.199
7.161
7.148
2.759
2.751
2.744
2.738
2.730
2.042
2.027
2.012
1.407
0.000
1.91
0.93
1.97
1.96
1.00
2.02
2.00
5.94

^{13}C-NMR (150 MHz, $CDCl_3$) of 6a

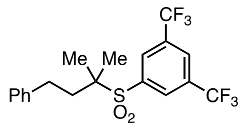

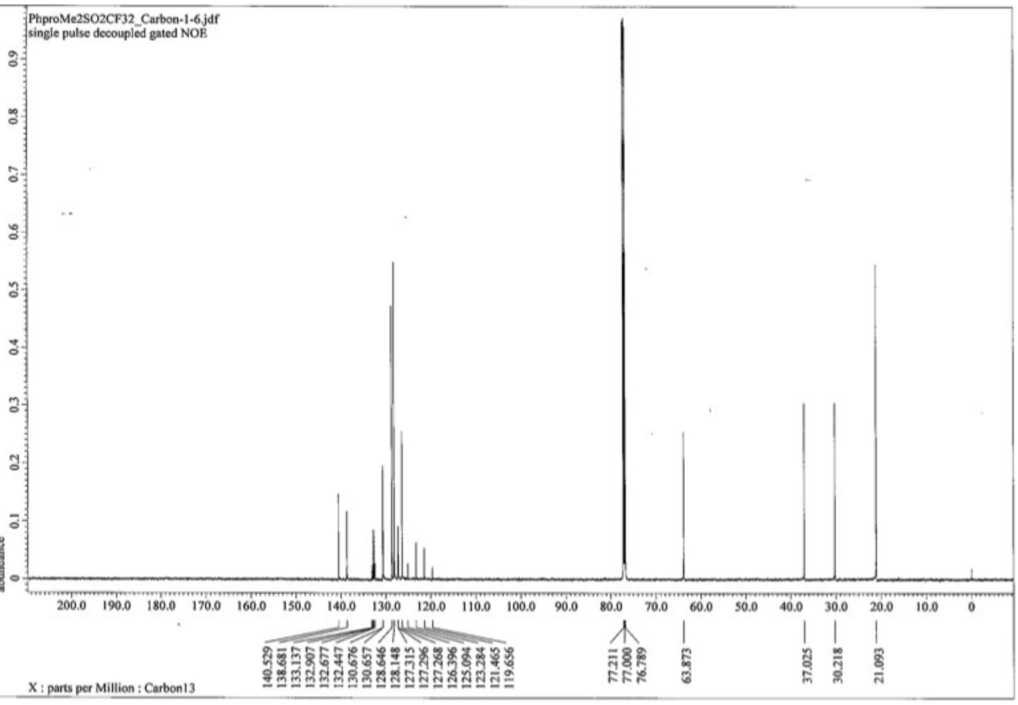

^{19}F-NMR (376 MHz, $CDCl_3$) of 6a

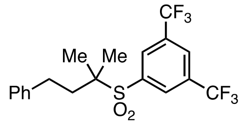

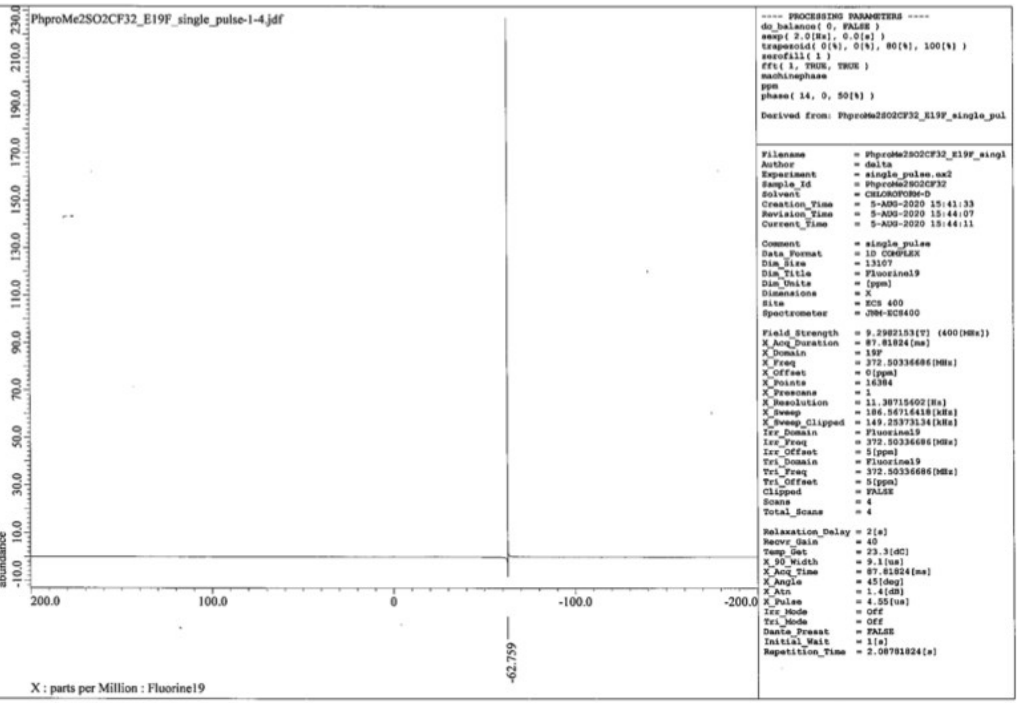

^{1}H-NMR (600 MHz, $CDCl_3$) of 7a

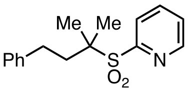

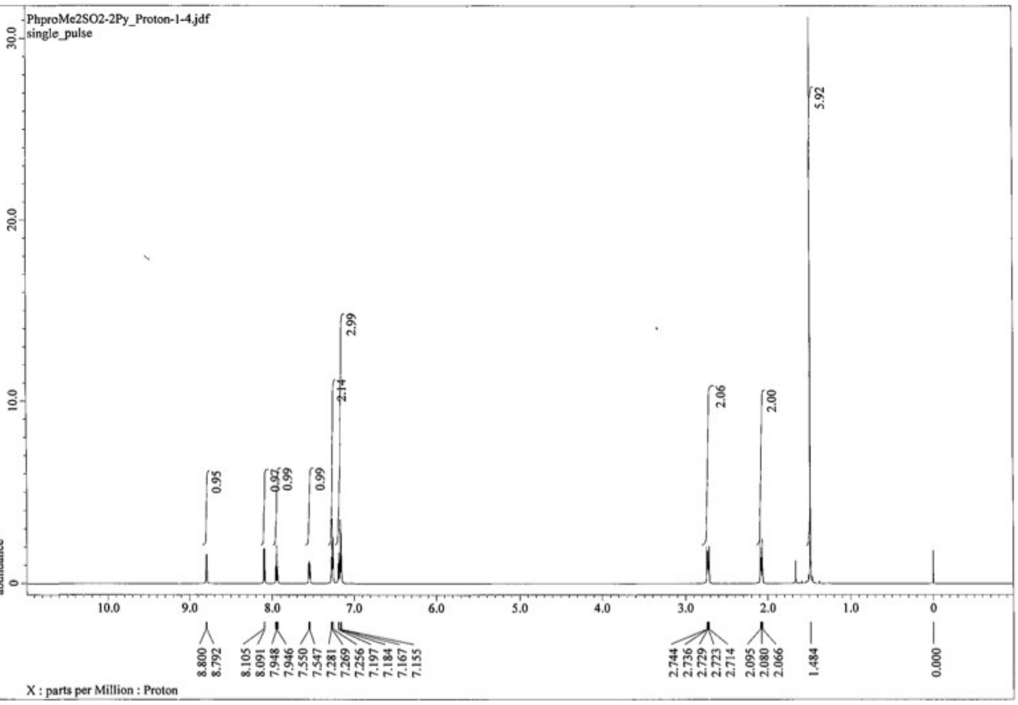

^{13}C-NMR (150 MHz, $CDCl_3$) of 7a

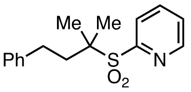

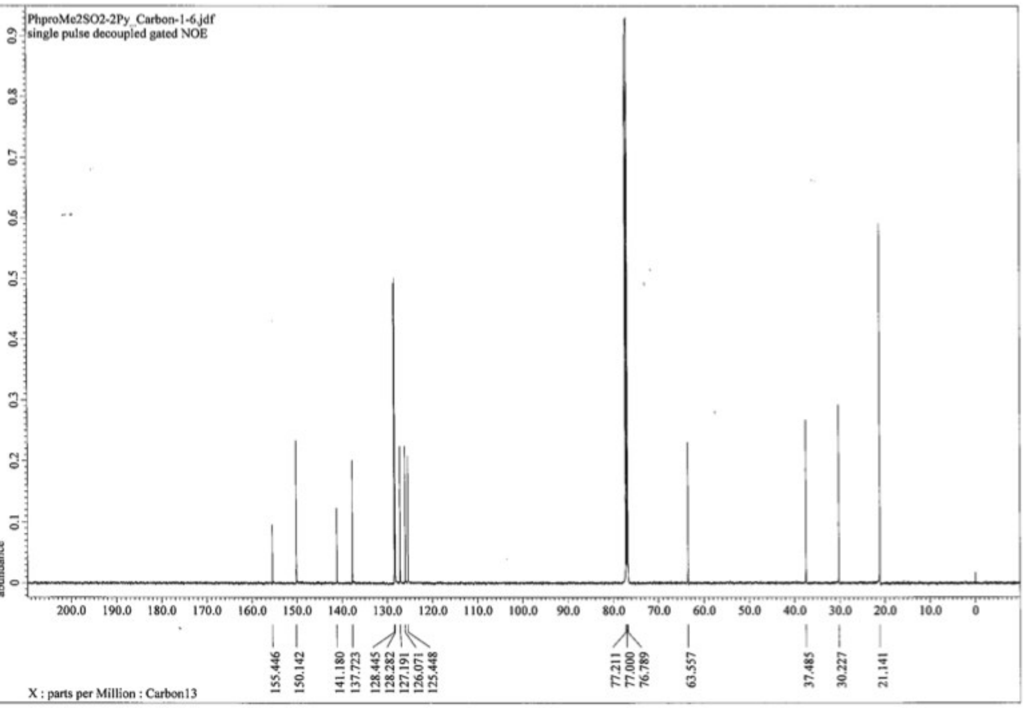

^{1}H-NMR (500 MHz, CDCl$_3$) of 5-(1'-(4-Pentenyl)cyclohexylsulfonyl)-1-phenyl-1*H*-tetrazole

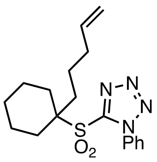

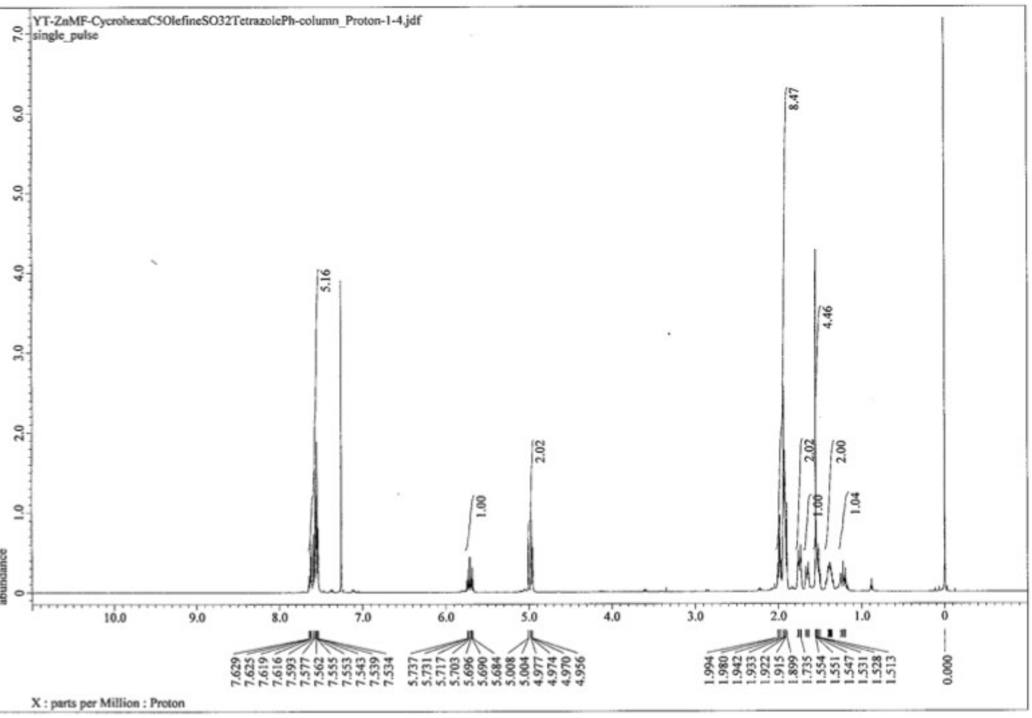

^{13}C-NMR (150 MHz, $CDCl_3$) of 5-(1'-(4-Pentenyl)cyclohexylsulfonyl)-1-phenyl-1*H*-tetrazole

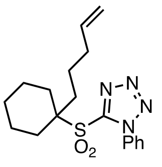

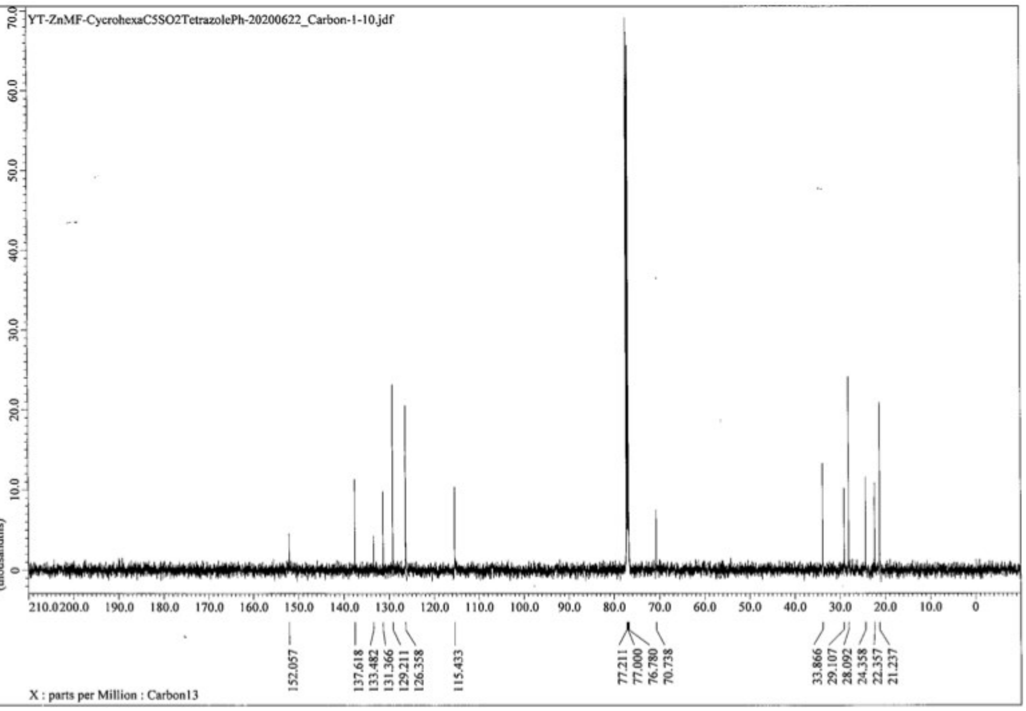

^{1}H-NMR (600 MHz, $CDCl_3$) of 4-(1'-(4-Pentenyl)-4-(1'-phenyl-1*H*-tetrazol-5'-yl)sulfonyl)tetra-hydropyran

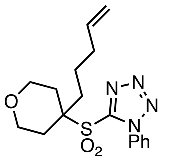

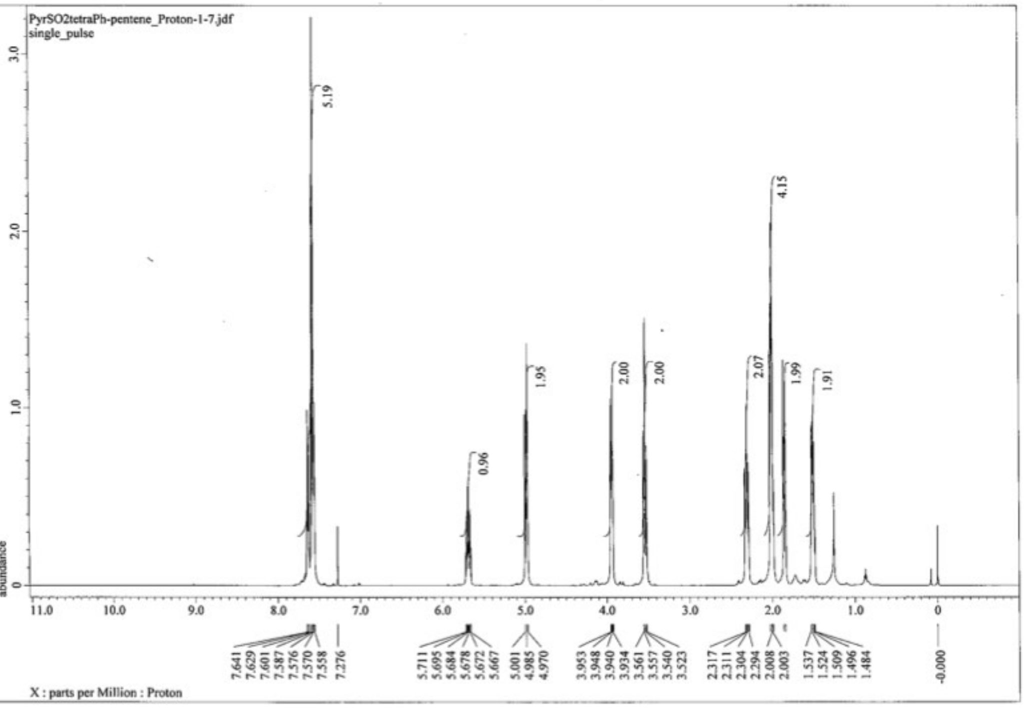

^{13}C-NMR (150 MHz, $CDCl_3$) of 4-(1'-(4-Pentenyl)-4-(1'-phenyl-1*H*-tetrazol-5'-yl)sulfonyl)tetra-hydropyran

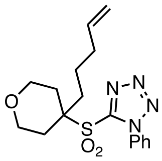

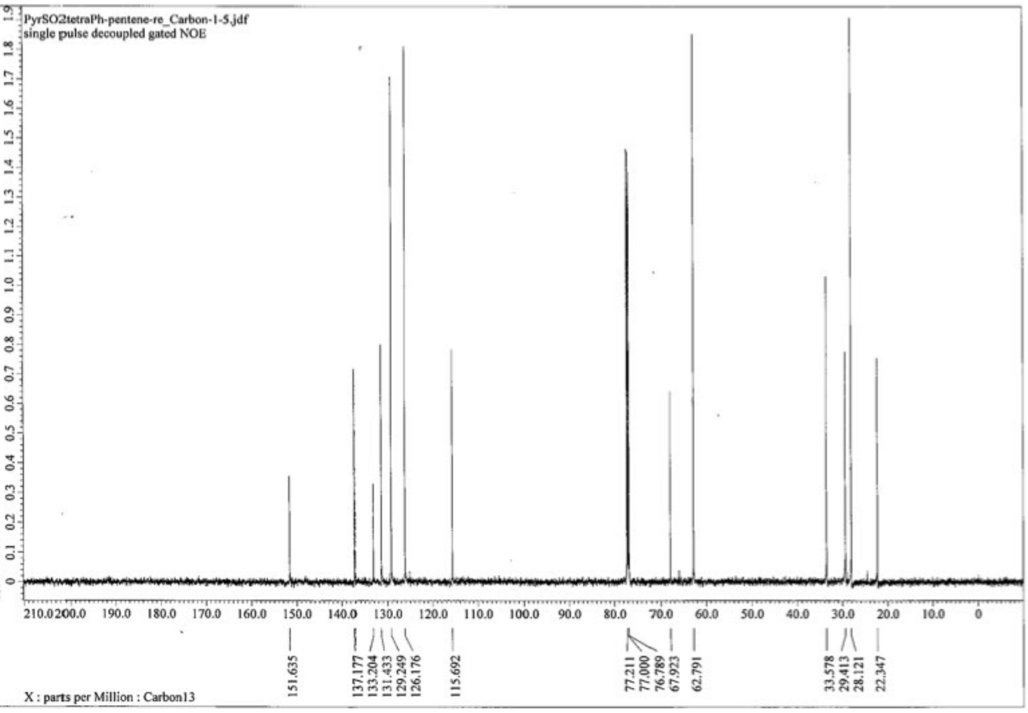

^{1}H-NMR (500 MHz, CDCl$_3$) of 5-(1'-(5-Hexenyl)cyclohexylsulfonyl)-1-phenyl-1*H*-tetrazole

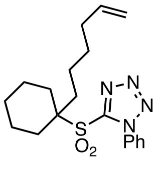

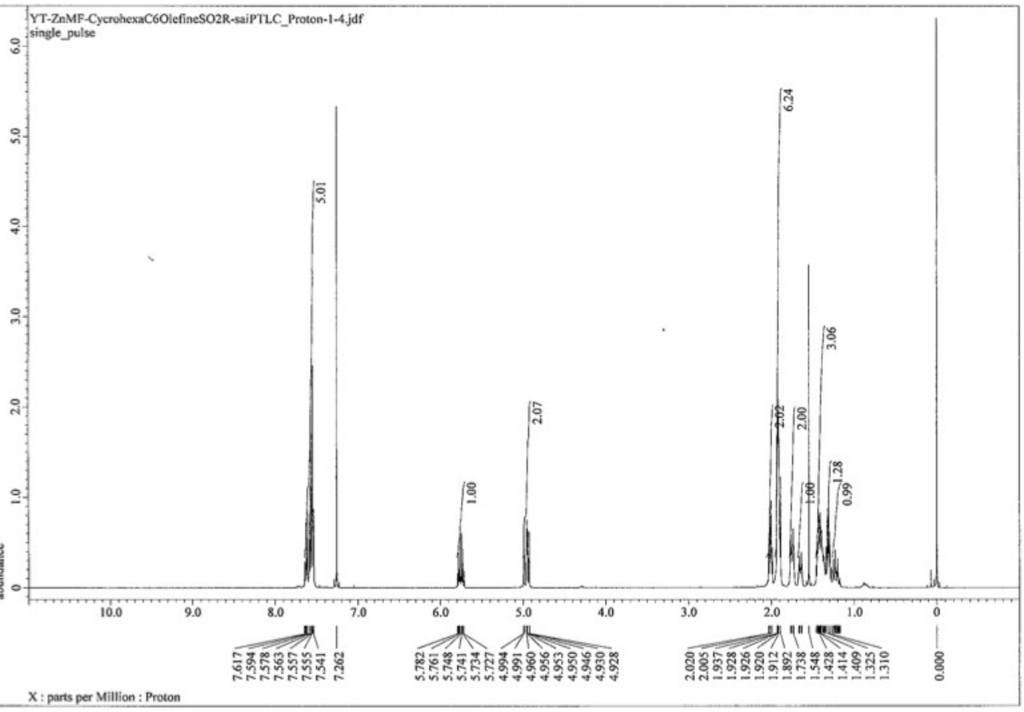

^{13}C-NMR (150 MHz, $CDCl_3$) of 5-(1'-(5-Hexenyl)cyclohexylsulfonyl)-1-phenyl-1*H*-tetrazole

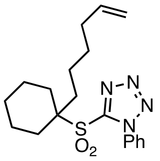

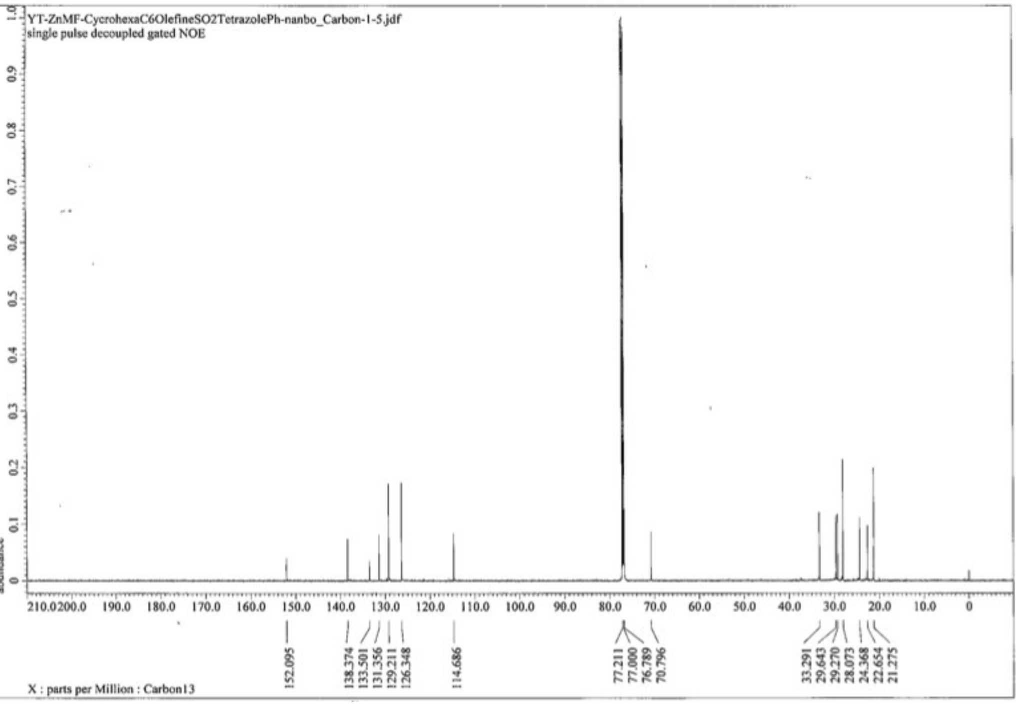

^{1}H-NMR (600 MHz, $CDCl_3$) of 3aa

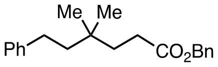

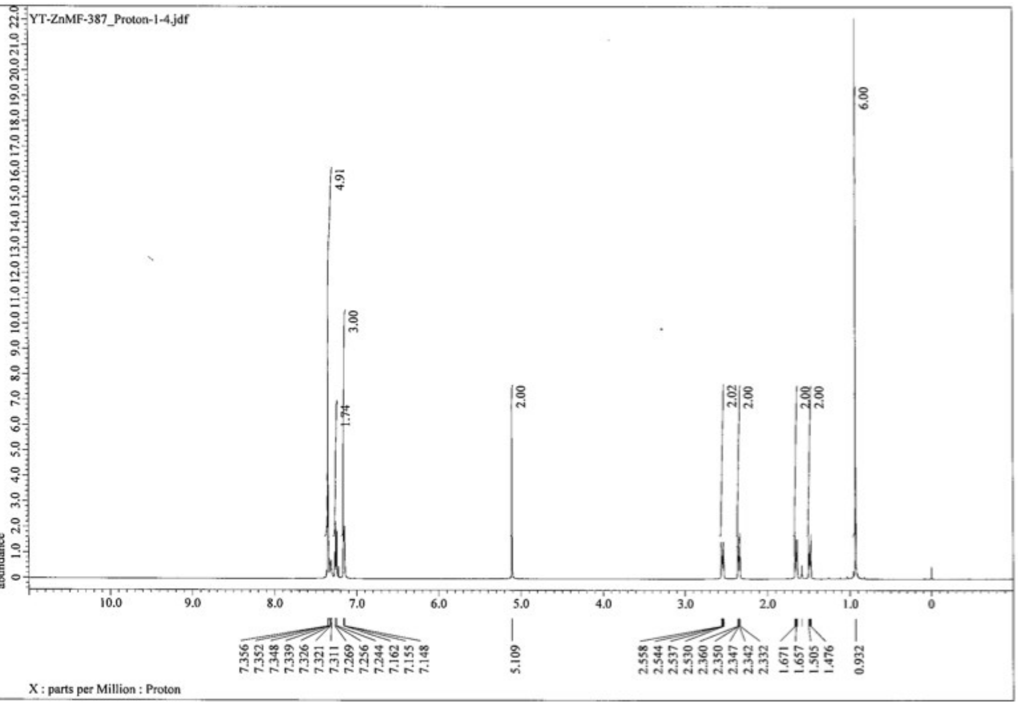

^{13}C-NMR (150 MHz, $CDCl_3$) of 3aa

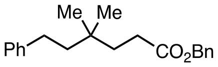

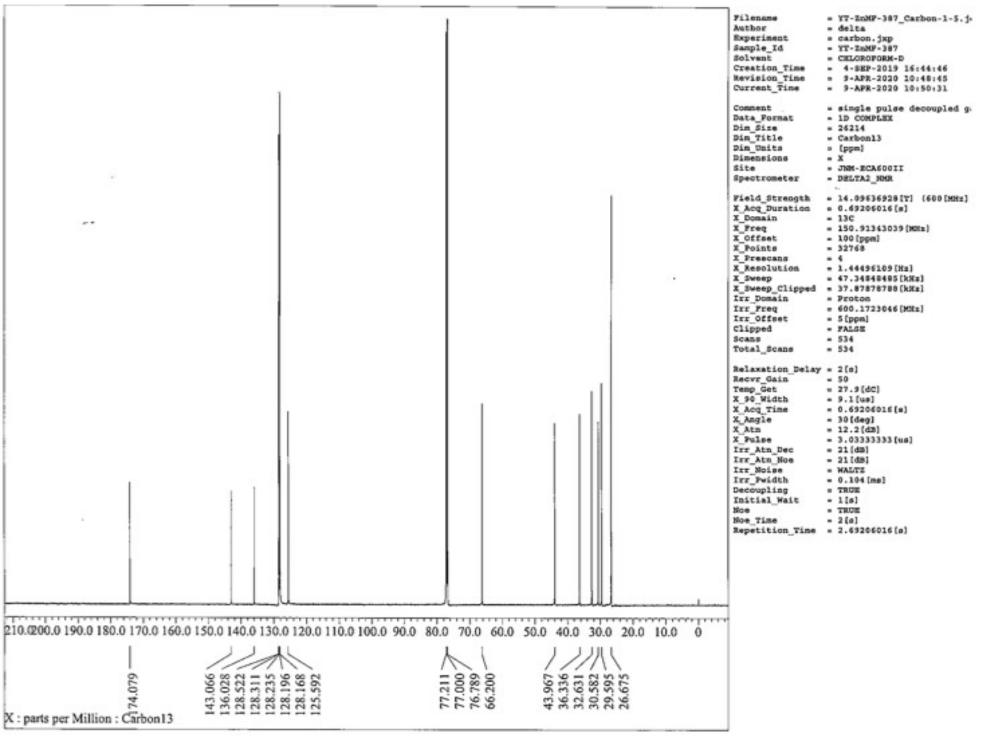
210.0 200.0 190.0 180.0 170.0 160.0 150.0 140.0 130.0 120.0 110.0 100.0 90.0 80.0 70.0 60.0 50.0 40.0 30.0 20.0 10.0 0
174.079
143.066
136.028
128.522
128.311
128.235
128.196
128.168
125.592
77.211
77.000
76.789
66.200
43.967
36.336
32.631
30.582
29.595
26.675
X : parts per Million : Carbon13
Filename = YT-ZnMF-387_Carbon-1-5.j
Author = delta
Experiment = carbon.jxp
Sample_Id = YT-ZnMF-387
Solvent = CHLOROFORM-D
Creation_Time = 4-SEP-2019 16:44:46
Revision_Time = 9-APR-2020 10:48:45
Current_Time = 9-APR-2020 10:50:31
Comment = single pulse decoupled g
Data_Format = 1D COMPLEX
Dim_Size = 26214
Dim_Title = Carbon13
Dim_Units = [ppm]
Dimensions = X
Site = JNM-ECA600II
Spectrometer = DELTA2_NMR
Field_Strength = 14.09636928[T] (600[MHz]
X_Acq_Duration = 0.69206016[s]
X_Domain = 13C
X_Freq = 150.91343039[MHz]
X_Offset = 100[ppm]
X_Points = 32768
X_Prescans = 4
X_Resolution = 1.44496109[Hz]
X_Sweep = 47.34848485[kHz]
X_Sweep_Clipped = 37.87878788[kHz]
Irr_Domain = Proton
Irr_Freq = 600.1723046[MHz]
Irr_Offset = 5[ppm]
Clipped = FALSE
Scans = 534
Total_Scans = 534
Relaxation_Delay = 2[s]
Recvr_Gain = 50
Temp_Get = 27.9[dC]
X_90_Width = 9.1[us]
X_Acq_Time = 0.69206016[s]
X_Angle = 30[deg]
X_Atn = 12.2[dB]
X_Pulse = 3.03333333[us]
Irr_Atn_Dec = 21[dB]
Irr_Atn_Noe = 21[dB]
Irr_Noise = WALTZ
Irr_Pwidth = 0.104[ms]
Decoupling = TRUE
Initial_Wait = 1[s]
Noe = TRUE
Noe_Time = 2[s]
Repetition_Time = 2.69206016[s]

^{1}H-NMR (600 MHz, $CDCl_3$) of 3ab

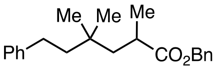

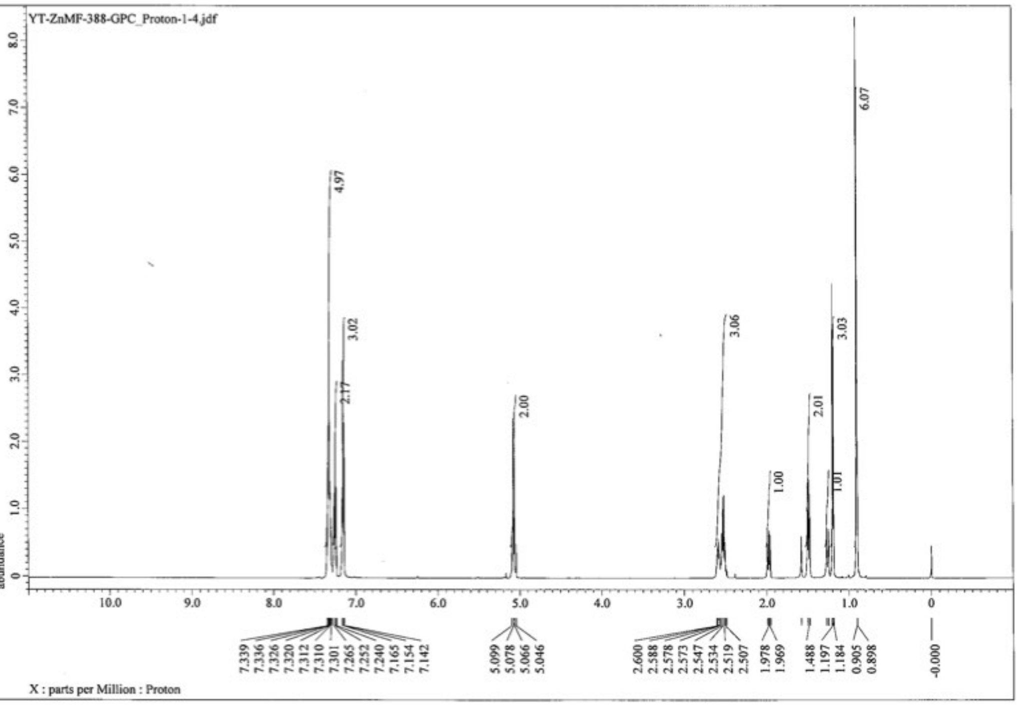

^{13}C-NMR (150 MHz, $CDCl_3$) of 3ab

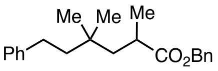

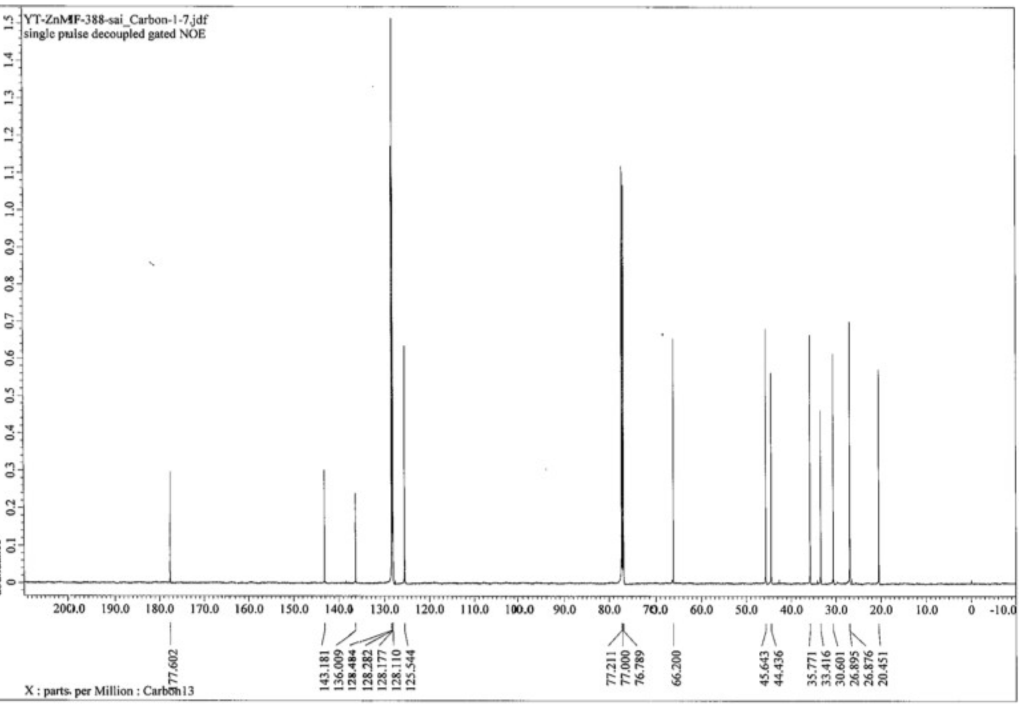

YT-ZnMF-388-sai_Carbon-1-7.jdf
single pulse decoupled gated NOE
X : parts per Million : Carbon13
177.602
143.181
136.009
128.484
128.282
128.177
128.110
125.544
77.211
77.000
76.789
66.200
45.643
44.436
35.771
33.416
30.601
26.895
26.876
20.451

^{1}H-NMR (600 MHz, $CDCl_3$) of 3ac

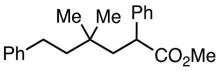

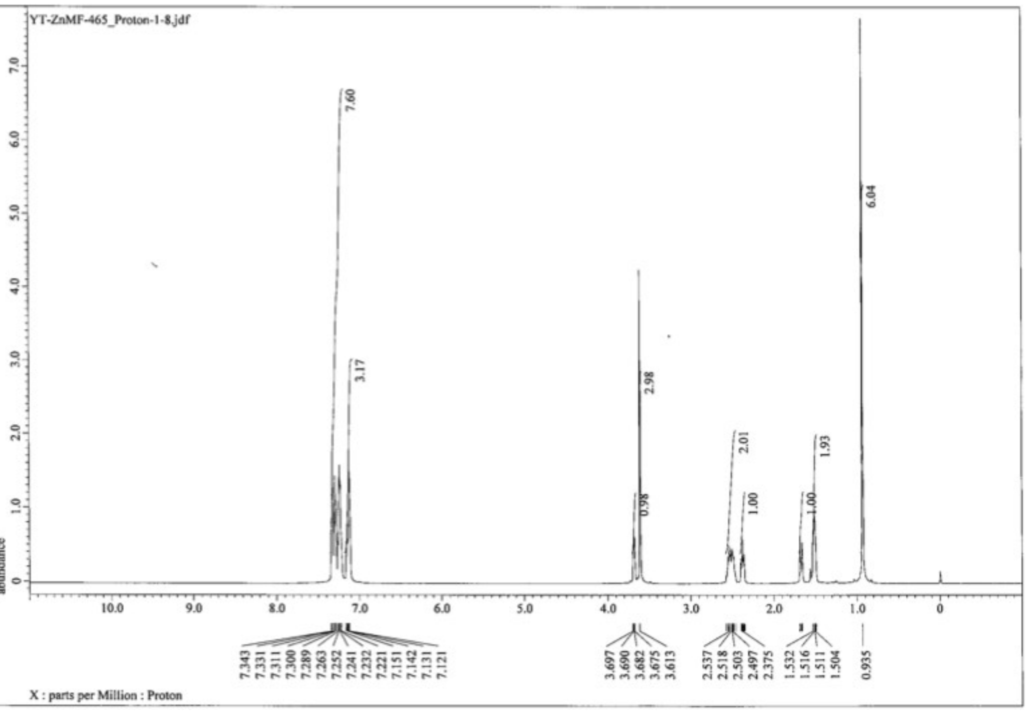

^{13}C-NMR (150 MHz, $CDCl_3$) of 3ac

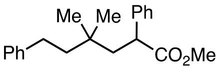

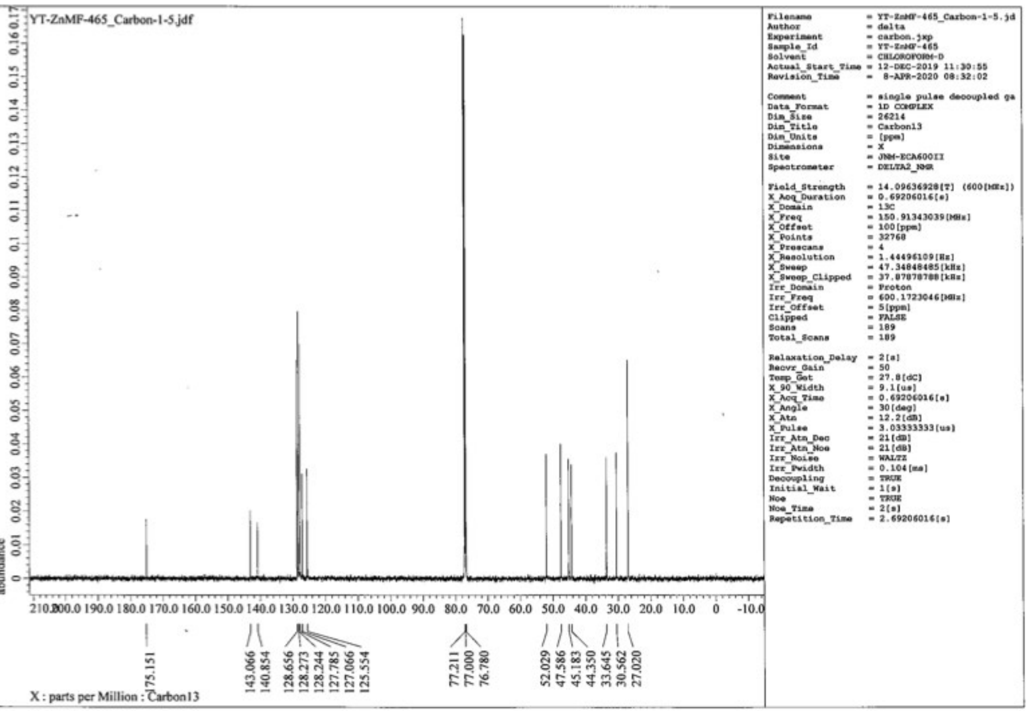
YT-ZnMF-465_Carbon-1-5.jdf
175.151
143.066
140.854
128.656
128.273
128.244
127.785
127.066
125.554
77.211
77.000
76.780
52.029
47.586
45.183
44.350
33.645
30.562
27.020
X : parts per Million : Carbon13
Filename = YT-ZnMF-465_Carbon-1-5.jd
Author = delta
Experiment = carbon.jxp
Sample_Id = YT-ZnMF-465
Solvent = CHLOROFORM-D
Actual_Start_Time = 12-DEC-2019 11:30:55
Revision_Time = 8-APR-2020 08:32:02
Comment = single pulse decoupled ga
Data_Format = 1D COMPLEX
Dim_Size = 26214
Dim_Title = Carbon13
Dim_Units = [ppm]
Dimensions = X
Site = JNM-ECA600II
Spectrometer = DELTA2_NMR
Field_Strength = 14.09636928[T] (600[MHz])
X_Acq_Duration = 0.69206016[s]
X_Domain = 13C
X_Freq = 150.91343039[MHz]
X_Offset = 100[ppm]
X_Points = 32768
X_Prescans = 4
X_Resolution = 1.44496109[Hz]
X_Sweep = 47.34848485[kHz]
X_Sweep_Clipped = 37.87878788[kHz]
Irr_Domain = Proton
Irr_Freq = 600.1723046[MHz]
Irr_Offset = 5[ppm]
Clipped = FALSE
Scans = 189
Total_Scans = 189
Relaxation_Delay = 2[s]
Recvr_Gain = 50
Temp_Get = 27.8[dC]
X_90_Width = 9.1[us]
X_Acq_Time = 0.69206016[s]
X_Angle = 30[deg]
X_Atn = 12.2[dB]
X_Pulse = 3.03333333[us]
Irr_Atn_Dec = 21[dB]
Irr_Atn_Noe = 21[dB]
Irr_Noise = WALTZ
Irr_Pwidth = 0.104[ms]
Decoupling = TRUE
Initial_Wait = 1[s]
Noe = TRUE
Noe_Time = 2[s]
Repetition_Time = 2.69206016[s]

^{1}H-NMR (600 MHz, $CDCl_3$) of 3ad

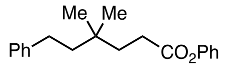

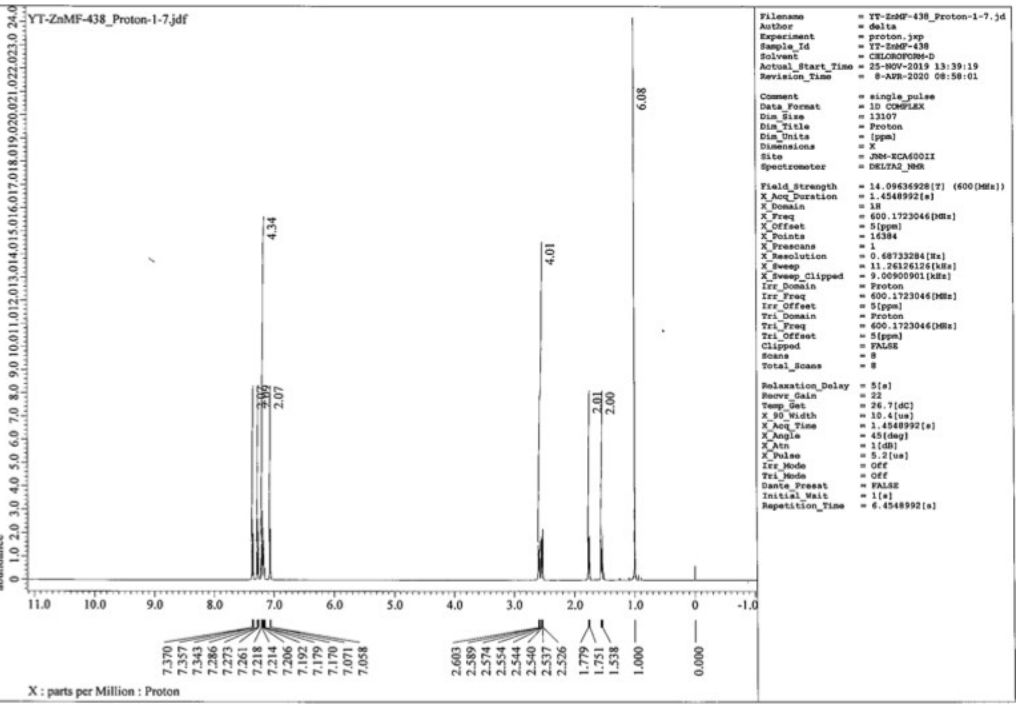

^{13}C-NMR (150 MHz, $CDCl_3$) of 3ad

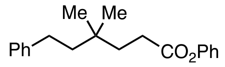

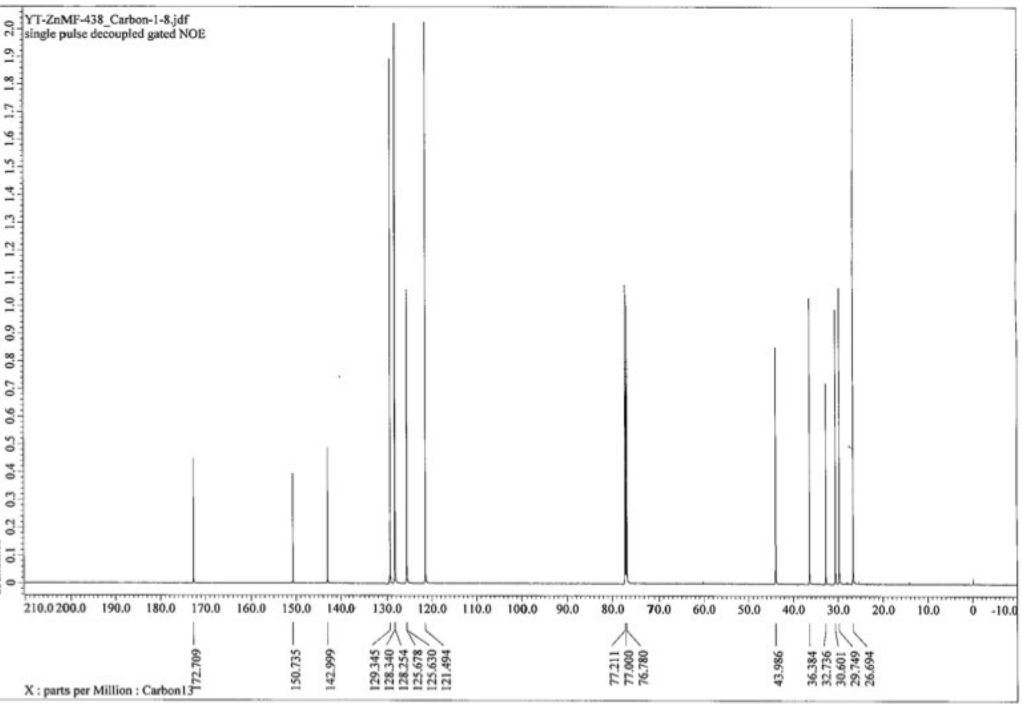

YT-ZnMF-438_Carbon-1-8.jdf
single pulse decoupled gated NOE
172.709
150.735
142.999
129.345
128.340
128.254
125.678
125.630
121.494
77.211
77.000
76.780
43.986
36.384
32.736
30.601
29.749
26.694
X : parts per Million : Carbon13

^{1}H-NMR (600 MHz, $CDCl_3$) of 3ae

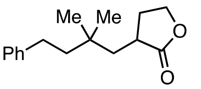

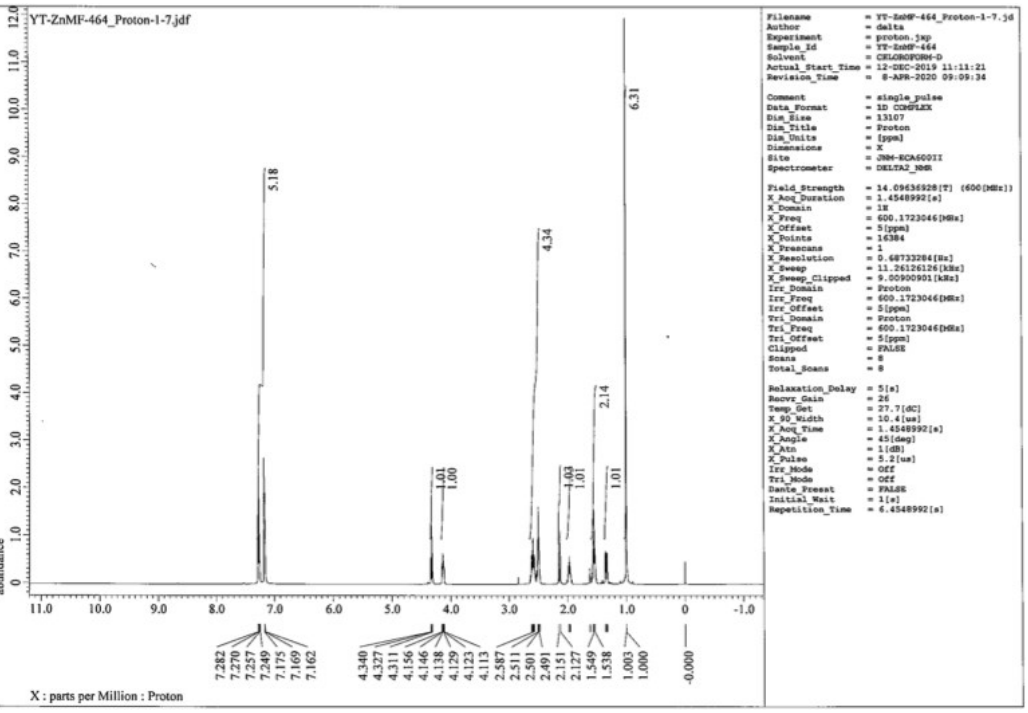

^{13}C-NMR (150 MHz, $CDCl_3$) of 3ae

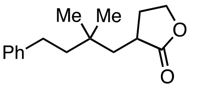

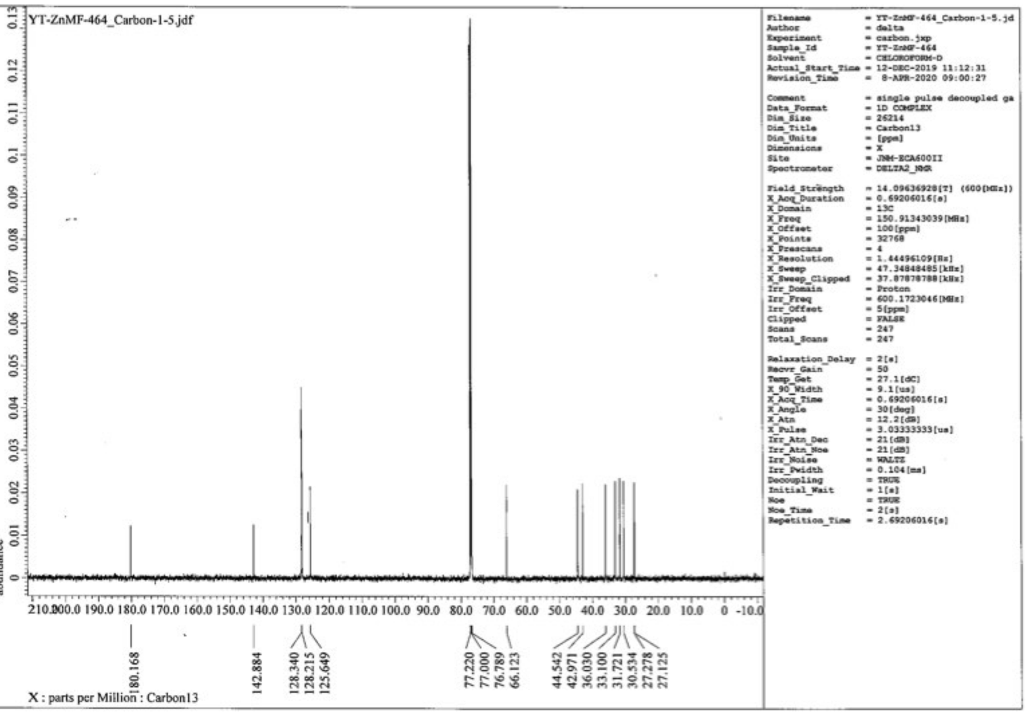

¹H-NMR (600 MHz, $CDCl_3$) of 3af

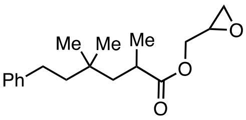

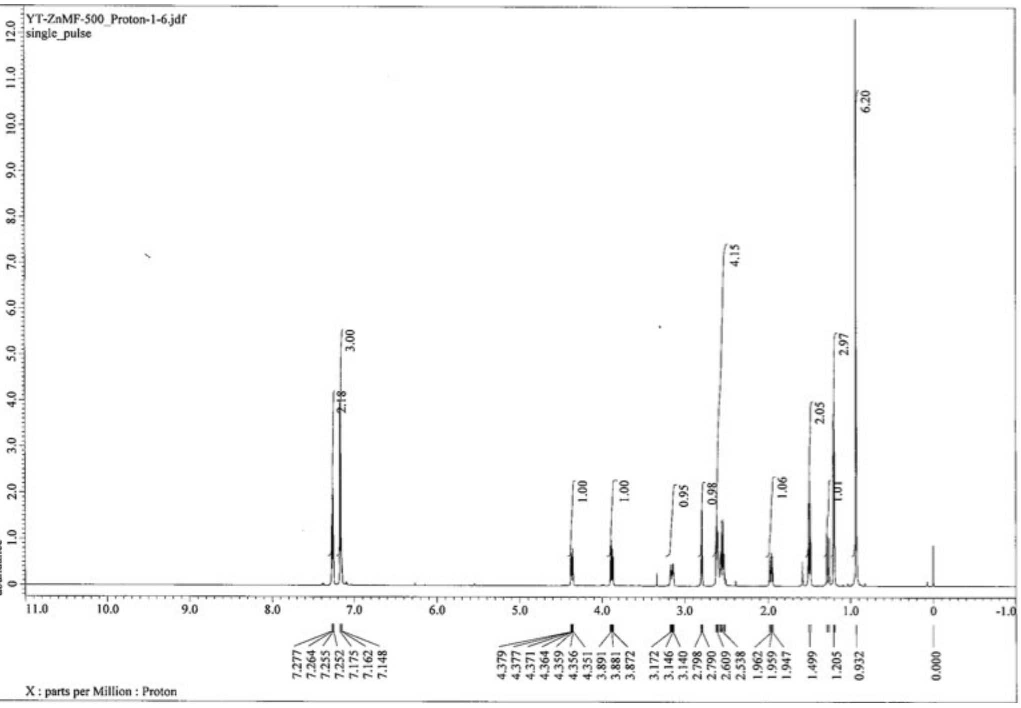

^{13}C-NMR (150 MHz, $CDCl_3$) of 3af

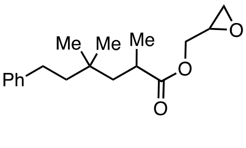

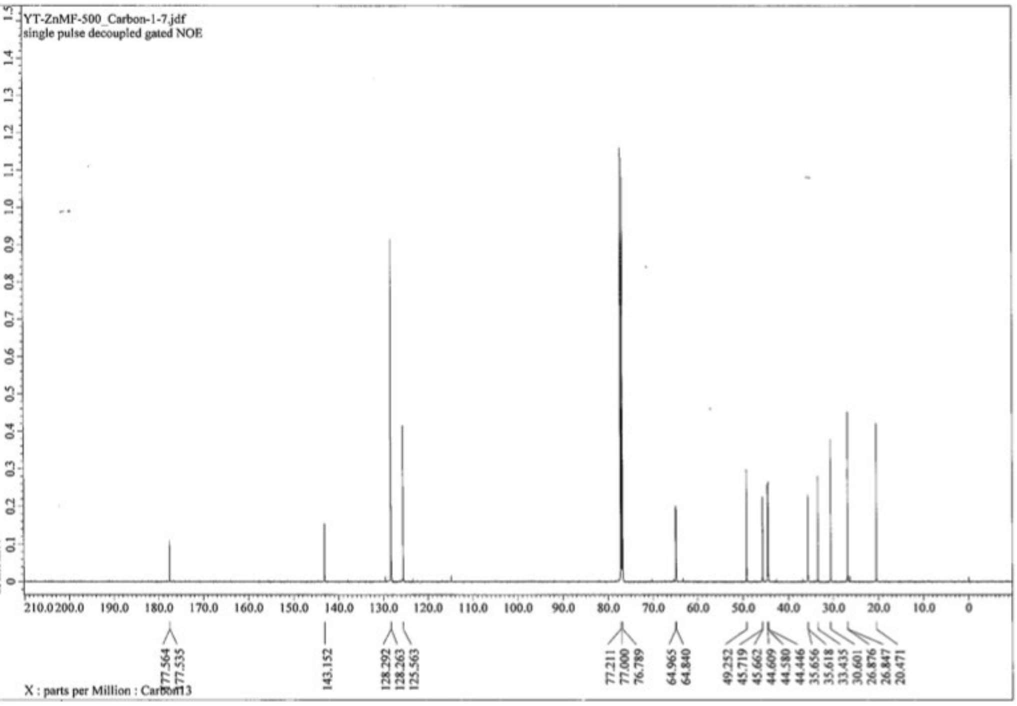

^{1}H-NMR (600 MHz, $CDCl_3$) of 3ag

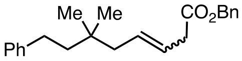

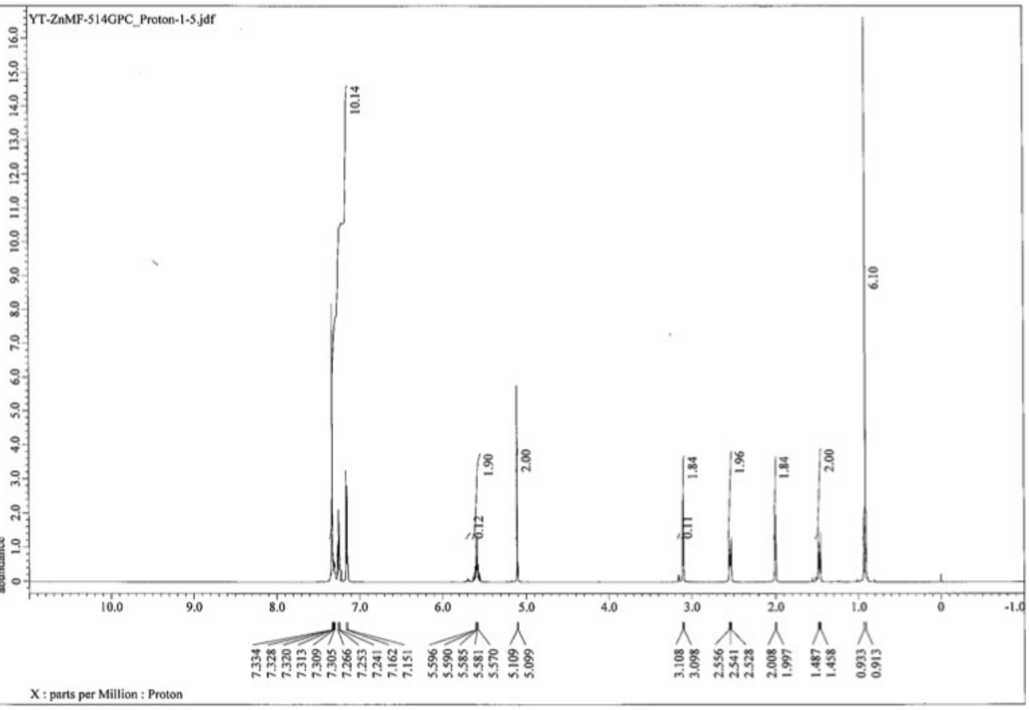

^{13}C-NMR (150 MHz, $CDCl_3$) of 3ag

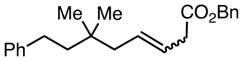

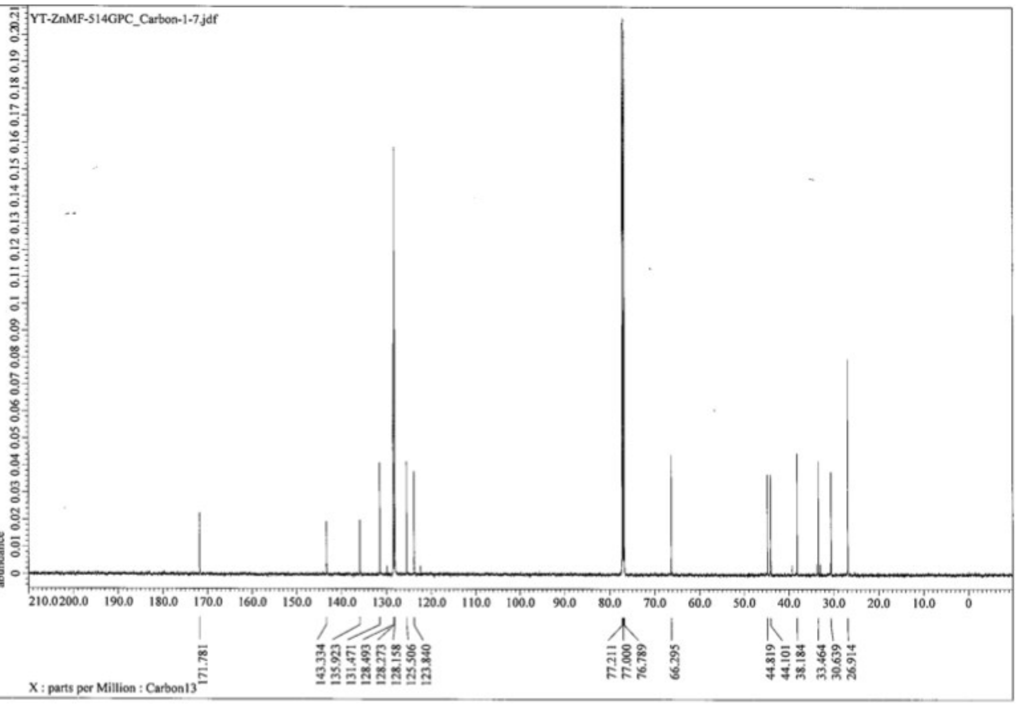
YT-ZnMF-514GPC_Carbon-1-7.jdf
abundance
X : parts per Million : Carbon13
171.781
143.334
135.923
131.471
128.493
128.273
128.158
125.506
123.840
77.211
77.000
76.789
66.295
44.819
44.101
38.184
33.464
30.639
26.914

^{1}H-NMR (600 MHz, $CDCl_3$) of 3ah

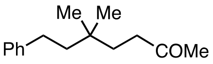

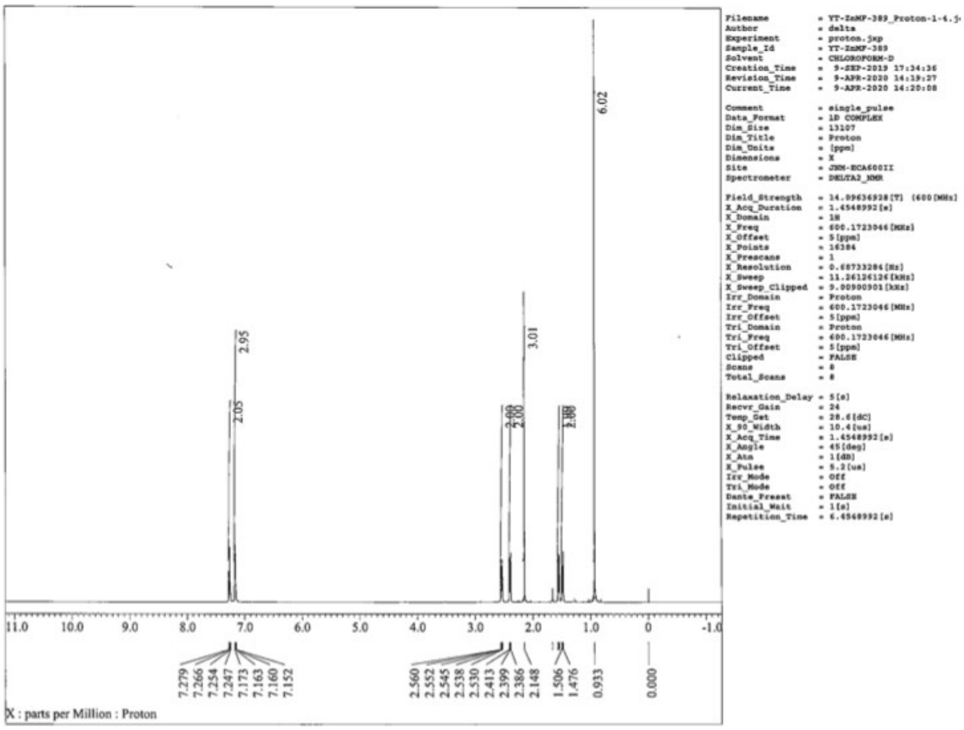

^{13}C-NMR (150 MHz, $CDCl_3$) of 3ah

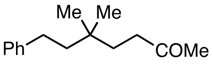

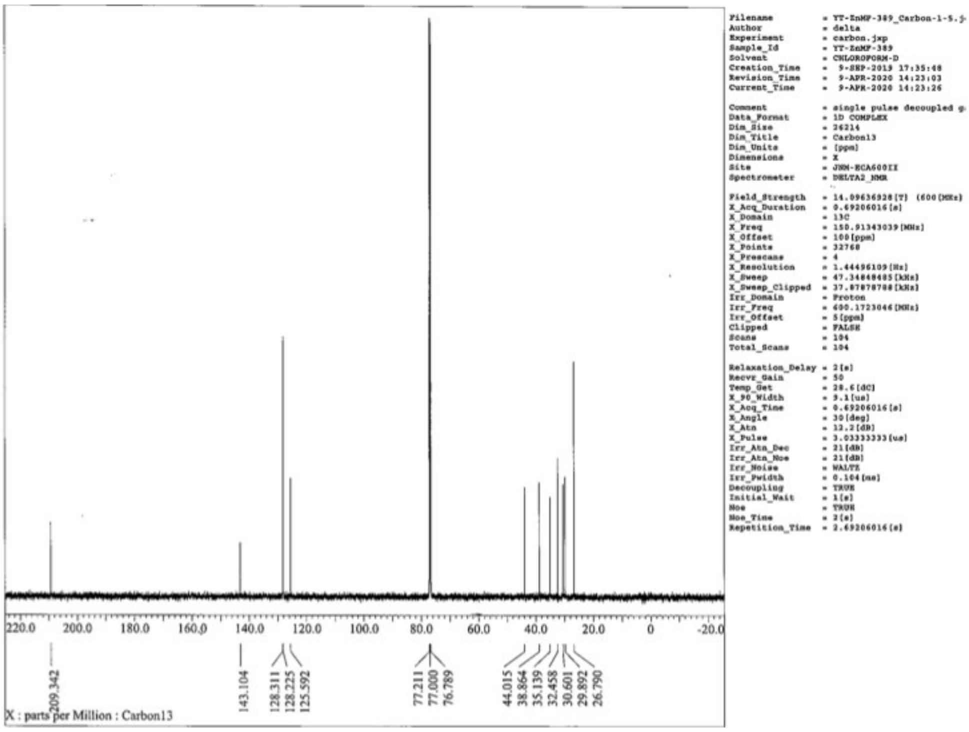

^{1}H-NMR (600 MHz, $CDCl_3$) of 3ai

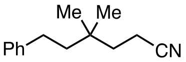

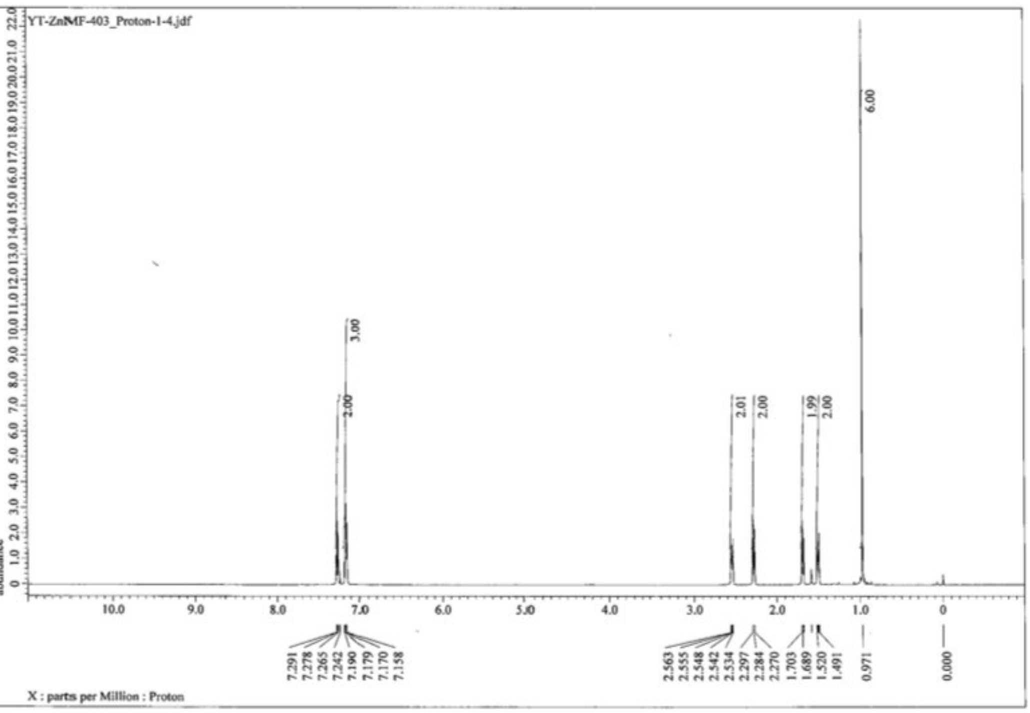

^{13}C-NMR (150 MHz, CDCl$_3$) of 3ai

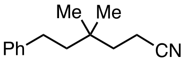

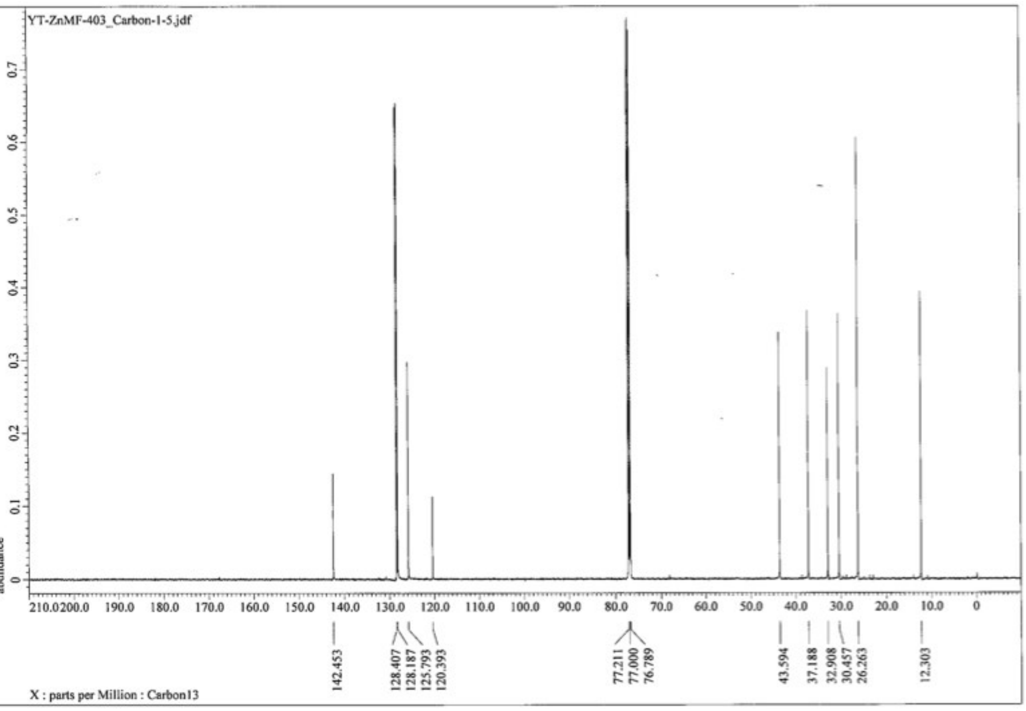

^{1}H-NMR (600 MHz, $CDCl_3$) of 3aj

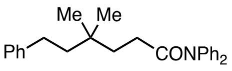

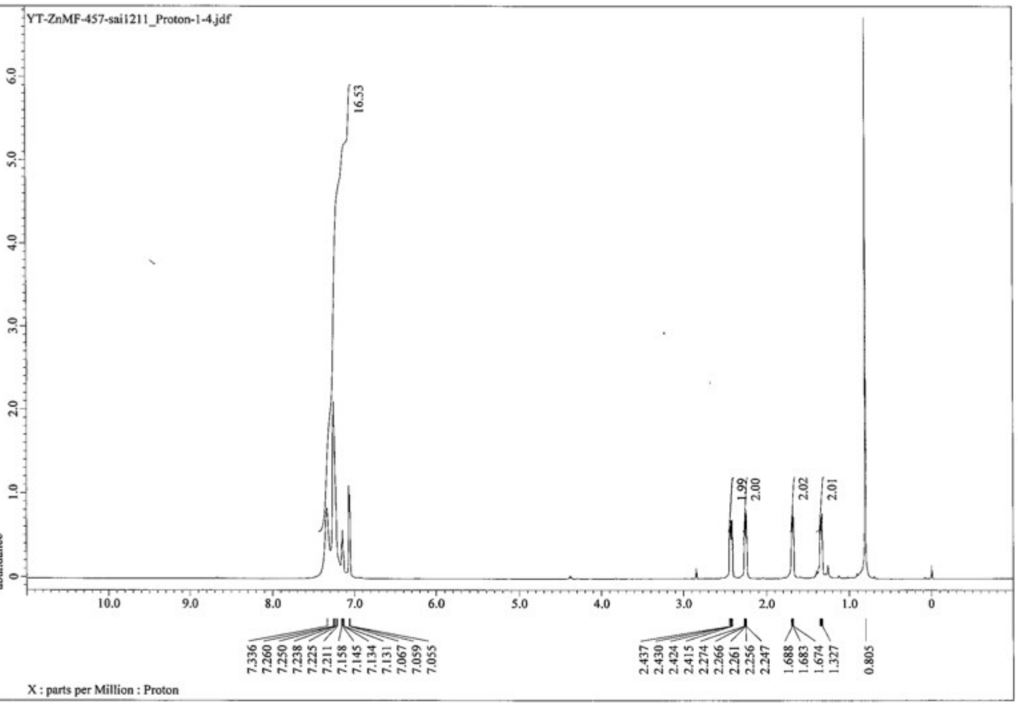

^{13}C-NMR (150 MHz, $CDCl_3$) of 3aj

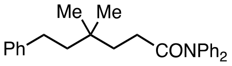

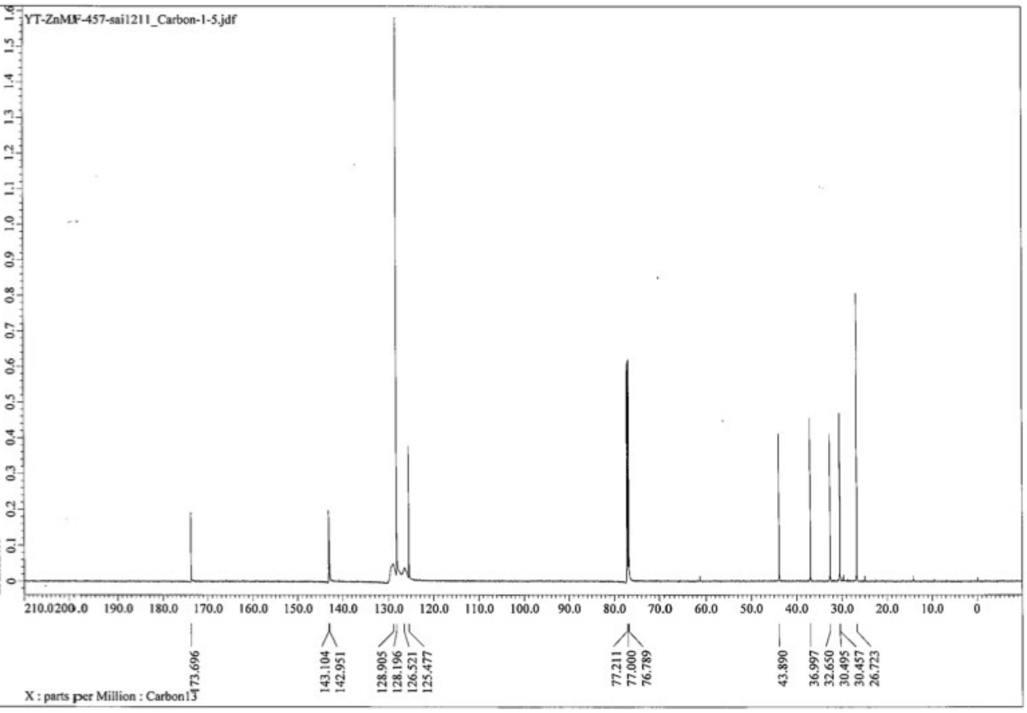

^{1}H-NMR (600 MHz, $CDCl_3$) of 3ak

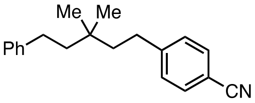

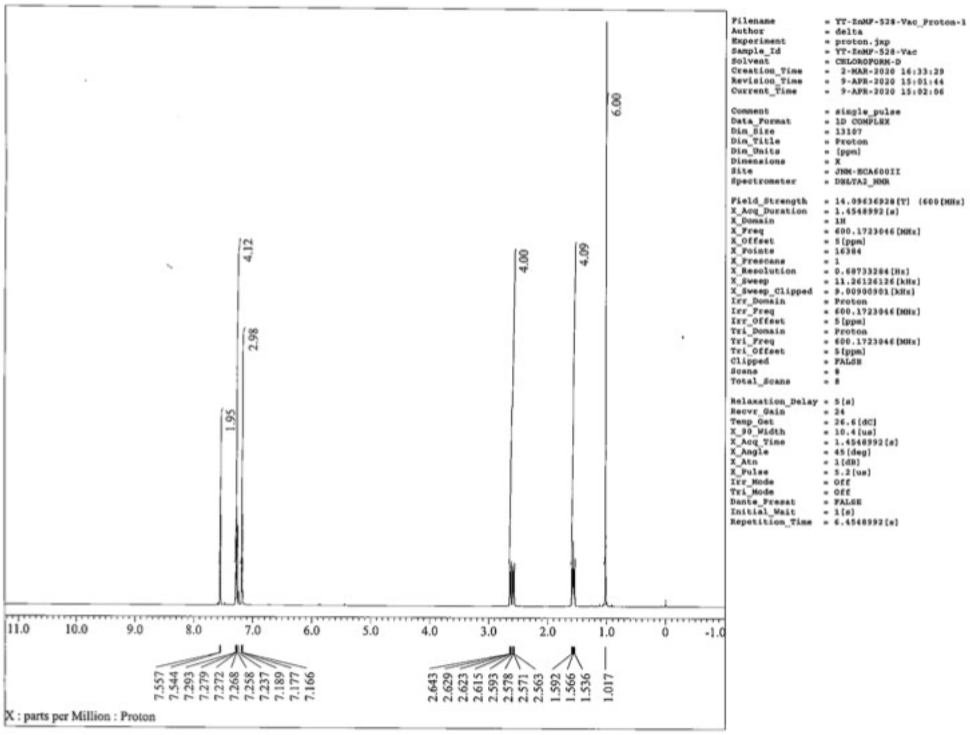

^{13}C-NMR (150 MHz, $CDCl_3$) of 3ak

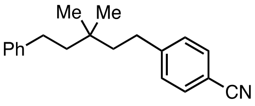

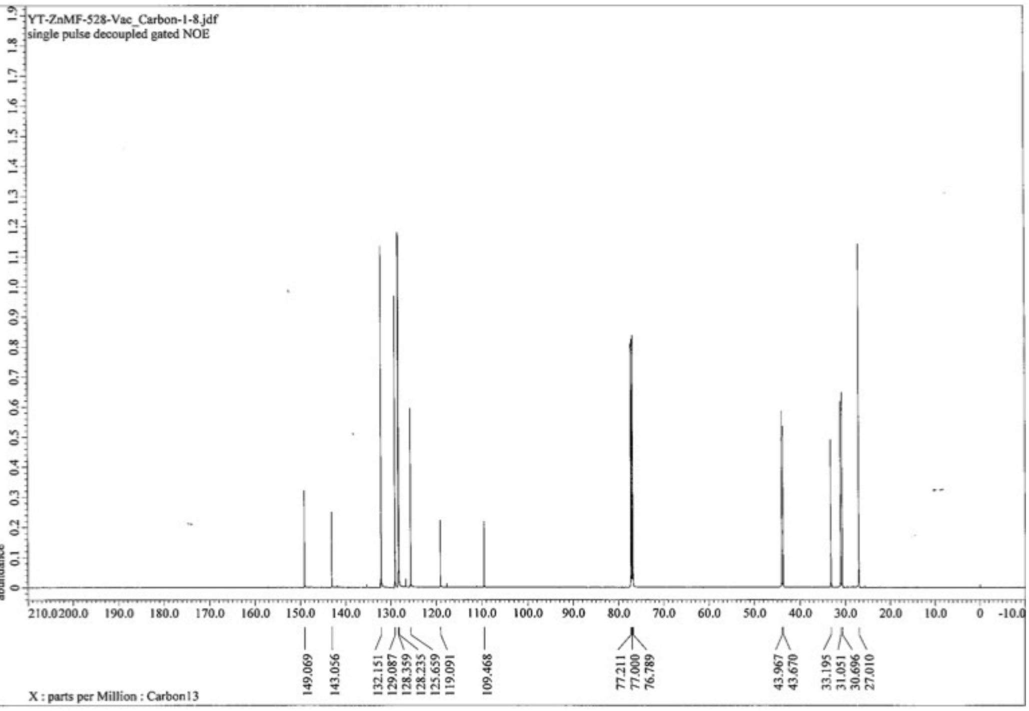

^{1}H-NMR (600 MHz, $CDCl_3$) of 3al

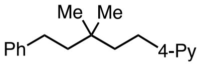

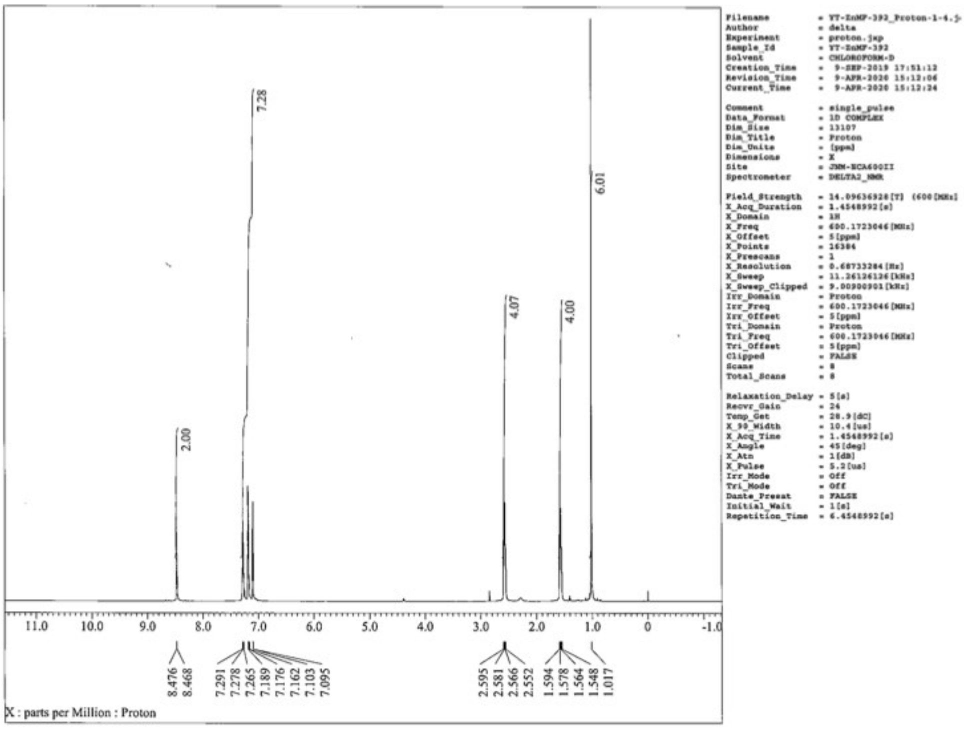

^{13}C-NMR (150 MHz, $CDCl_3$) of 3al

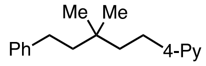

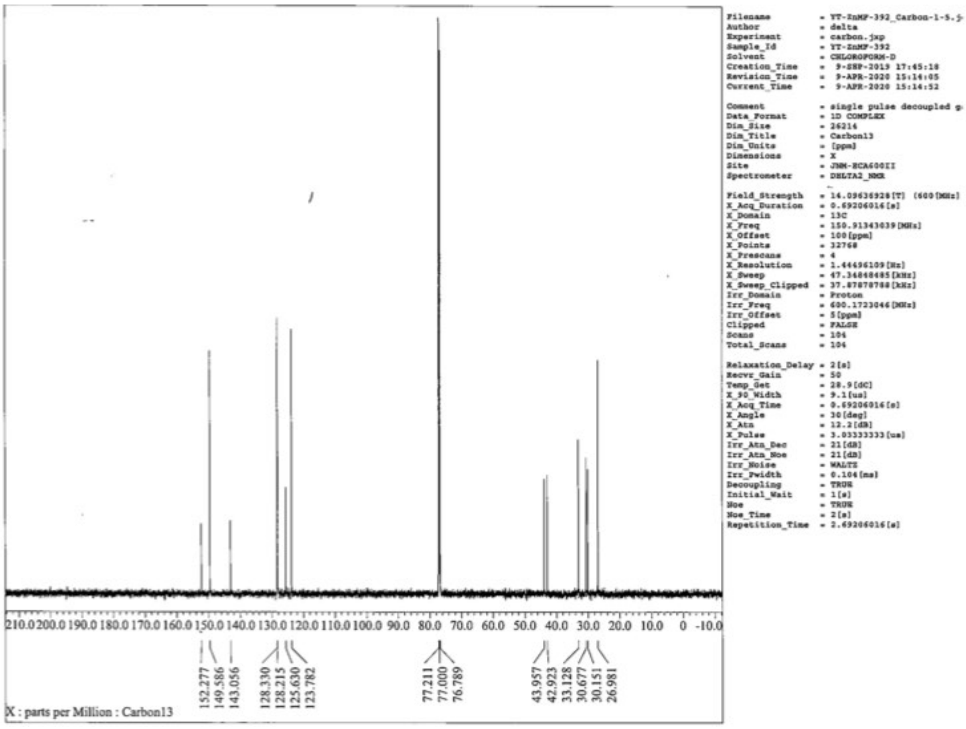

^{1}H-NMR (600 MHz, $CDCl_3$) of 3am

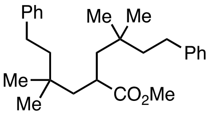

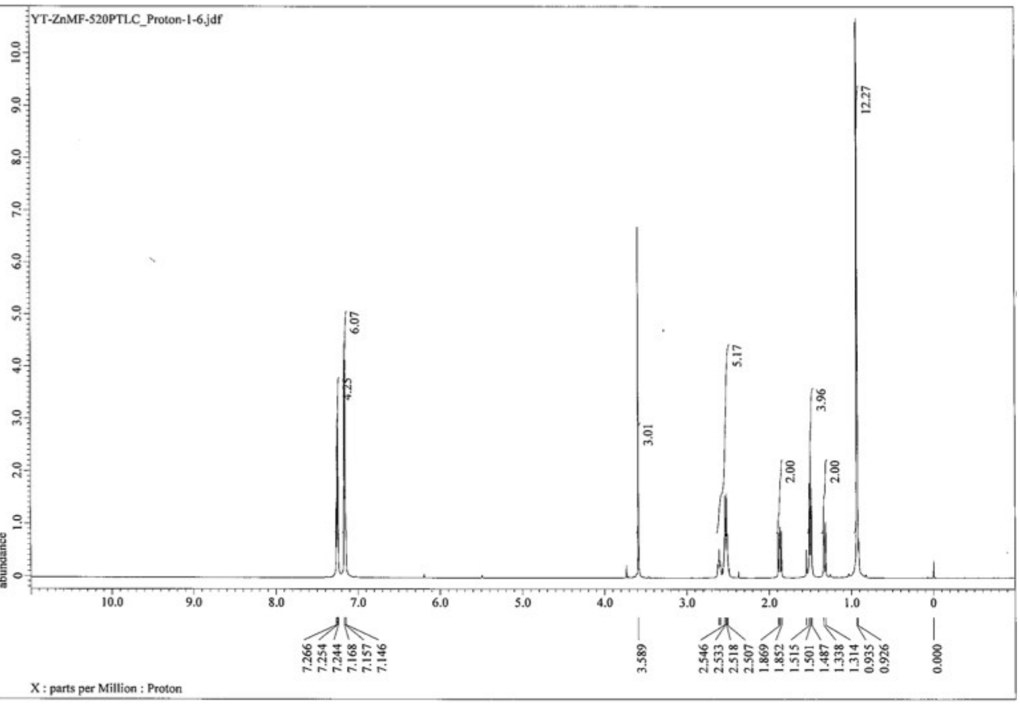

^{13}C-NMR (150 MHz, $CDCl_3$) of 3am

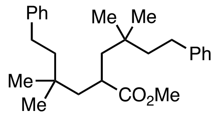

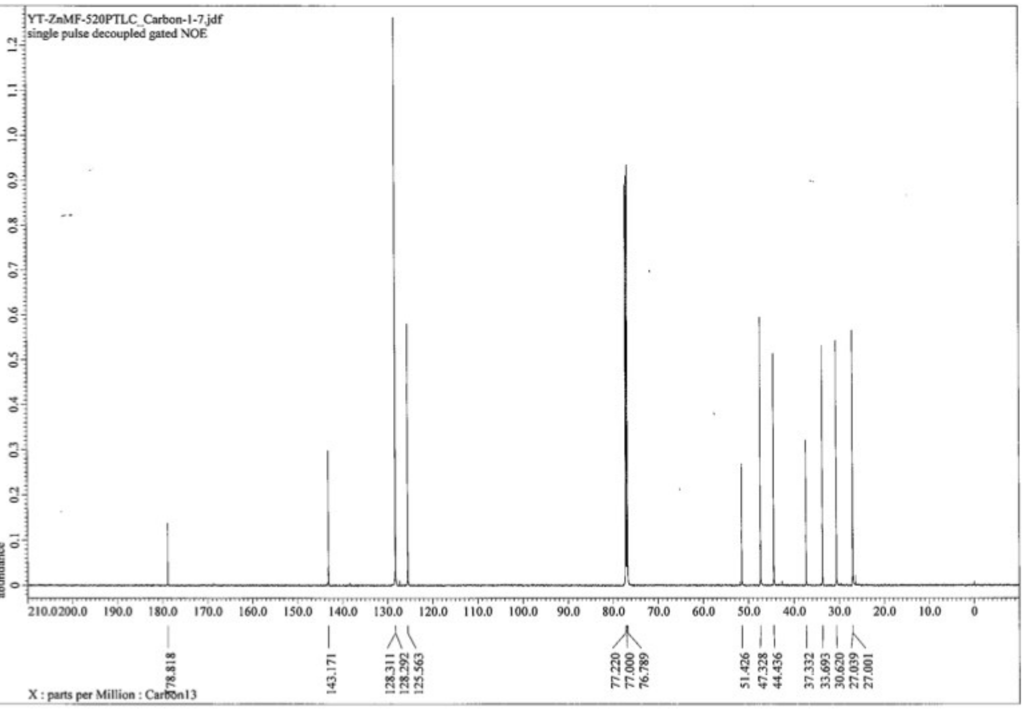

^{1}H-NMR (600 MHz, $CDCl_3$) of 3ba

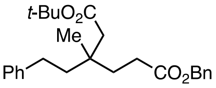

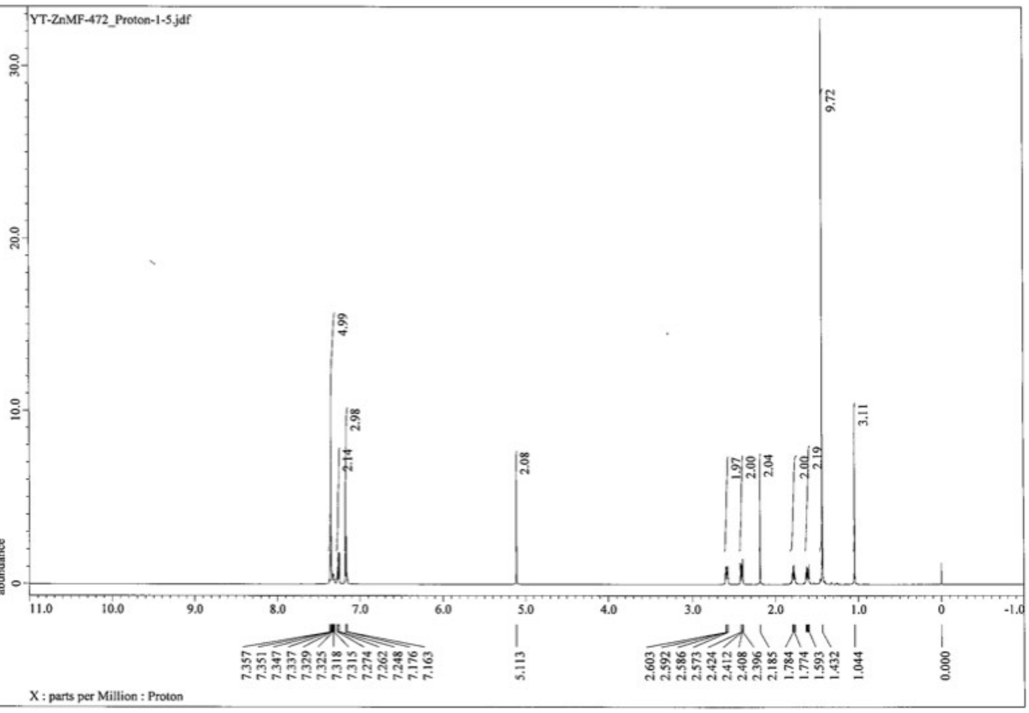

^{13}C-NMR (150 MHz, $CDCl_3$) of 3ba

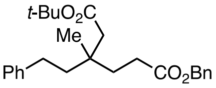

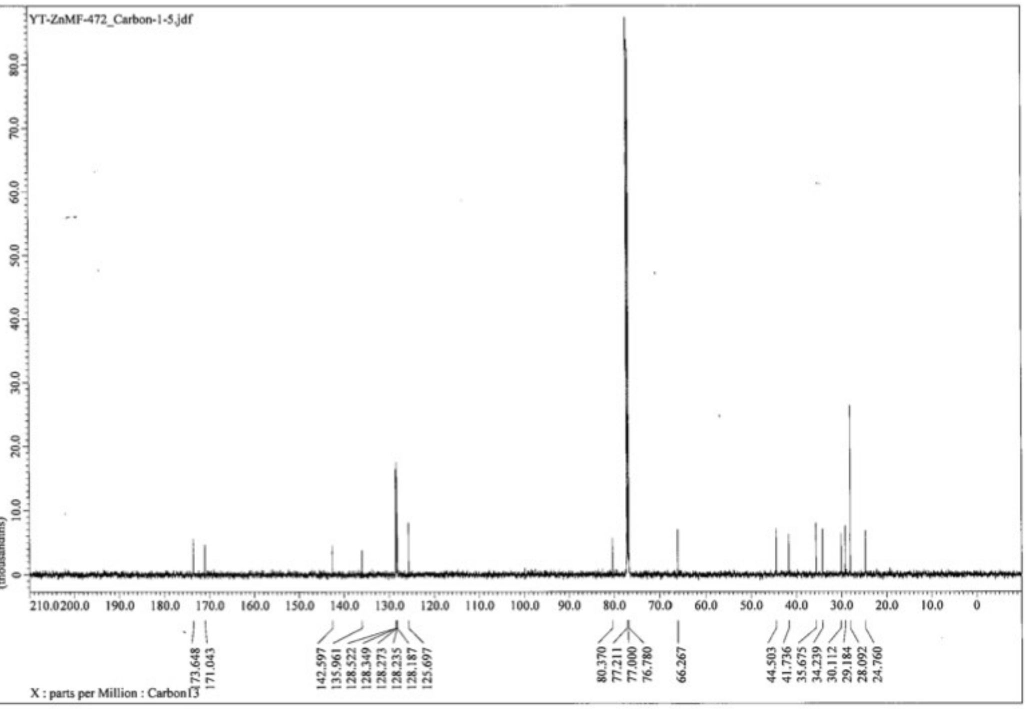

^{1}H-NMR (600 MHz, $CDCl_3$) of 3ca

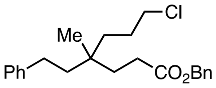

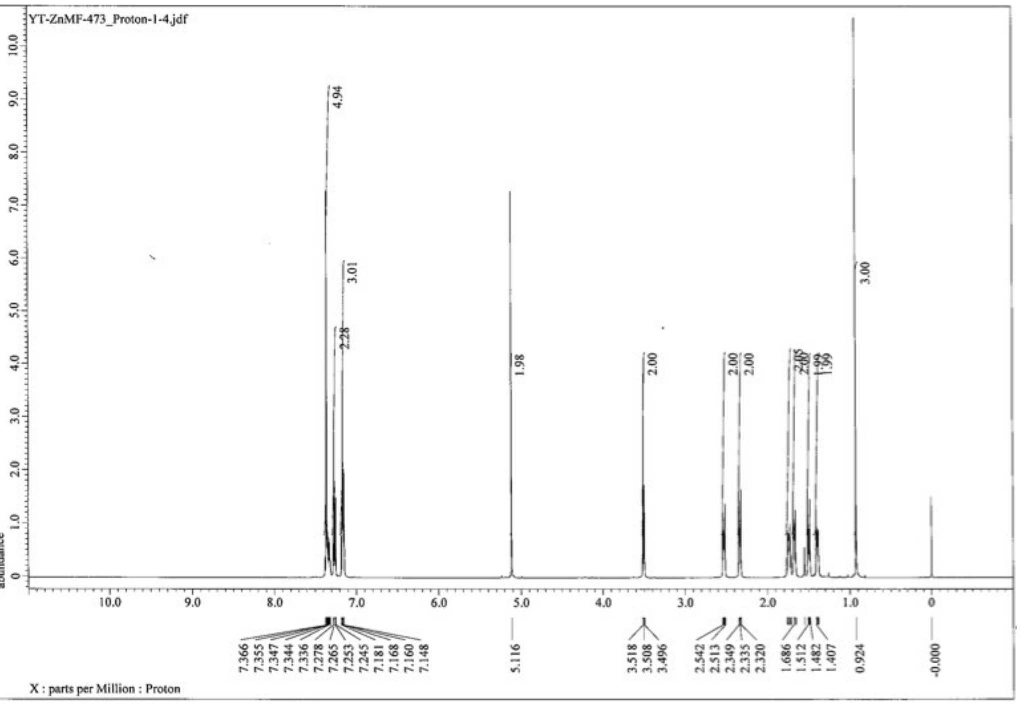

^{13}C-NMR (150 MHz, $CDCl_3$) of 3ca

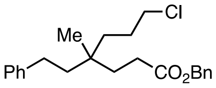

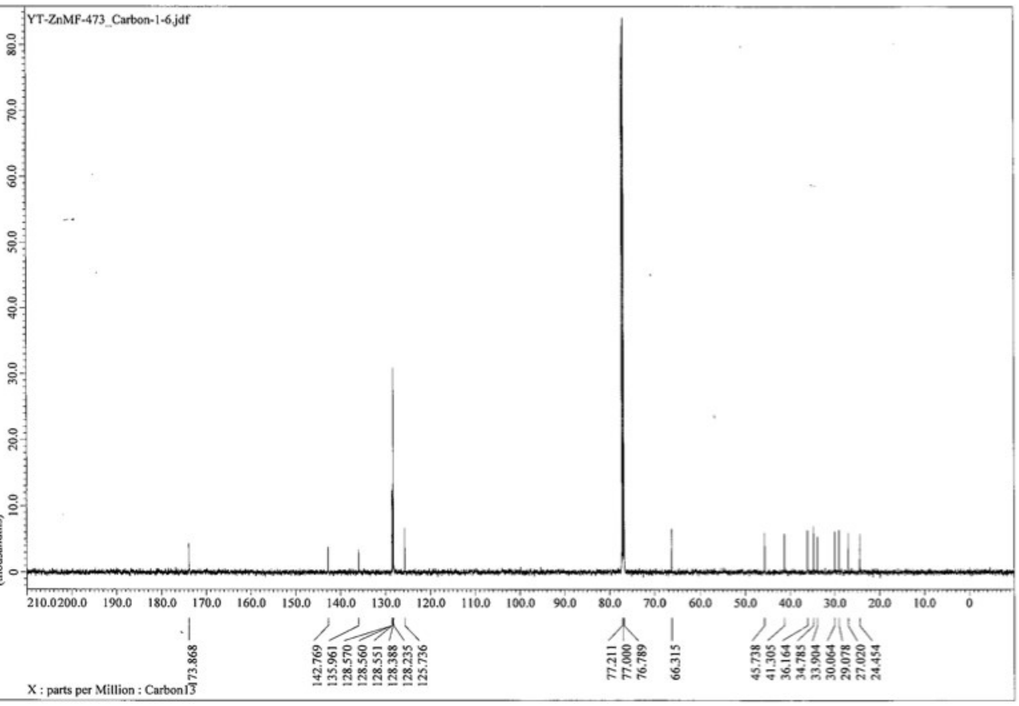

YT-ZnMF-473_Carbon-1-6.jdf
X : parts per Million : Carbon13
173.868
142.769
135.961
128.570
128.560
128.551
128.388
128.235
125.736
77.211
77.000
76.789
66.315
45.738
41.305
36.164
34.785
33.904
30.064
29.078
27.020
24.454

^{1}H-NMR (600 MHz, $CDCl_3$) of 3da

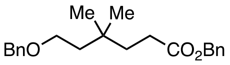

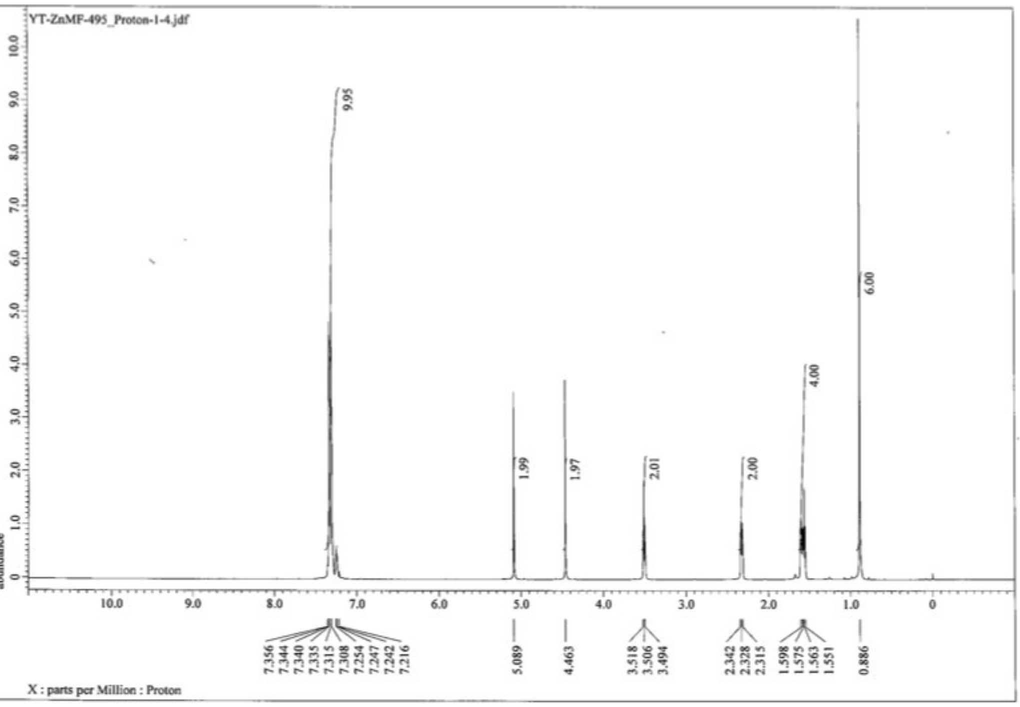

^{13}C-NMR (150 MHz, $CDCl_3$) of 3da

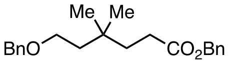

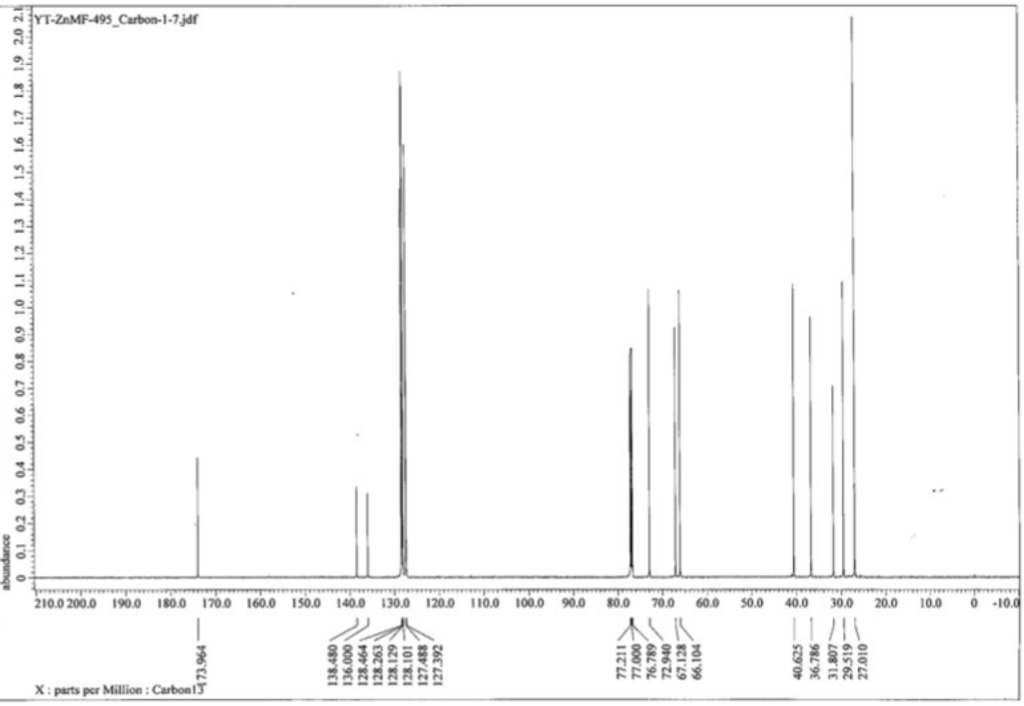
YT-ZnMF-495_Carbon-1-7.jdf
abundance
X : parts per Million : Carbon13
173.964
138.480
136.000
128.464
128.263
128.129
128.101
127.488
127.392
77.211
77.000
76.789
72.940
67.128
66.104
40.625
36.786
31.807
29.519
27.010

^{1}H-NMR (600 MHz, $CDCl_3$) of 3ea

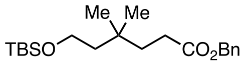

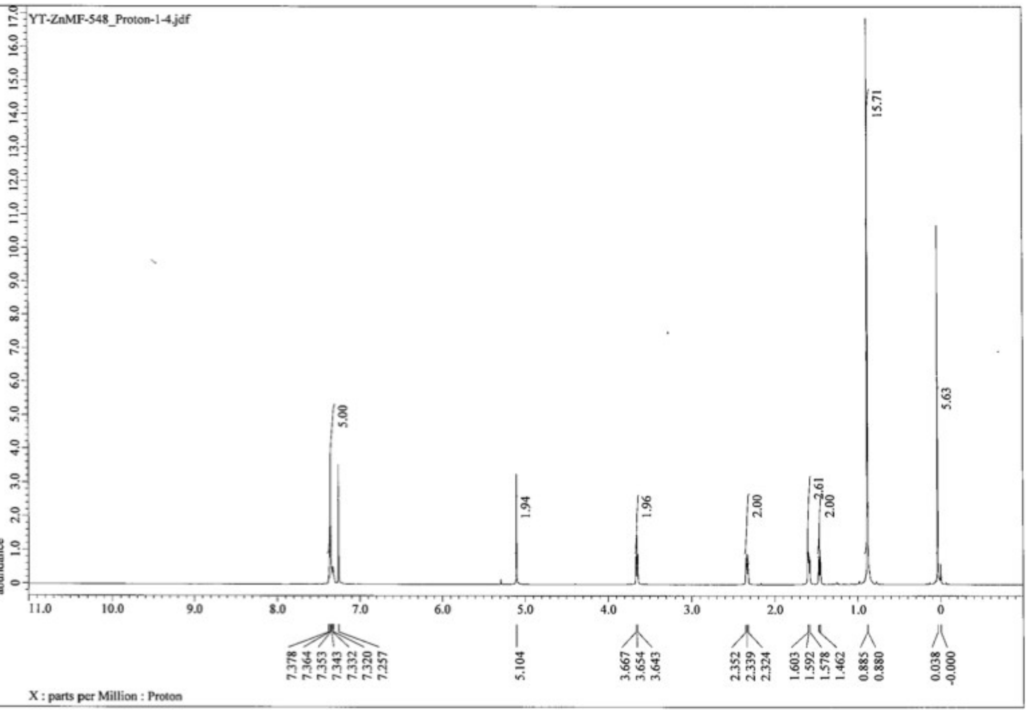

^{13}C-NMR (150 MHz, $CDCl_3$) of 3ea

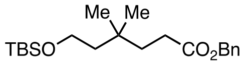

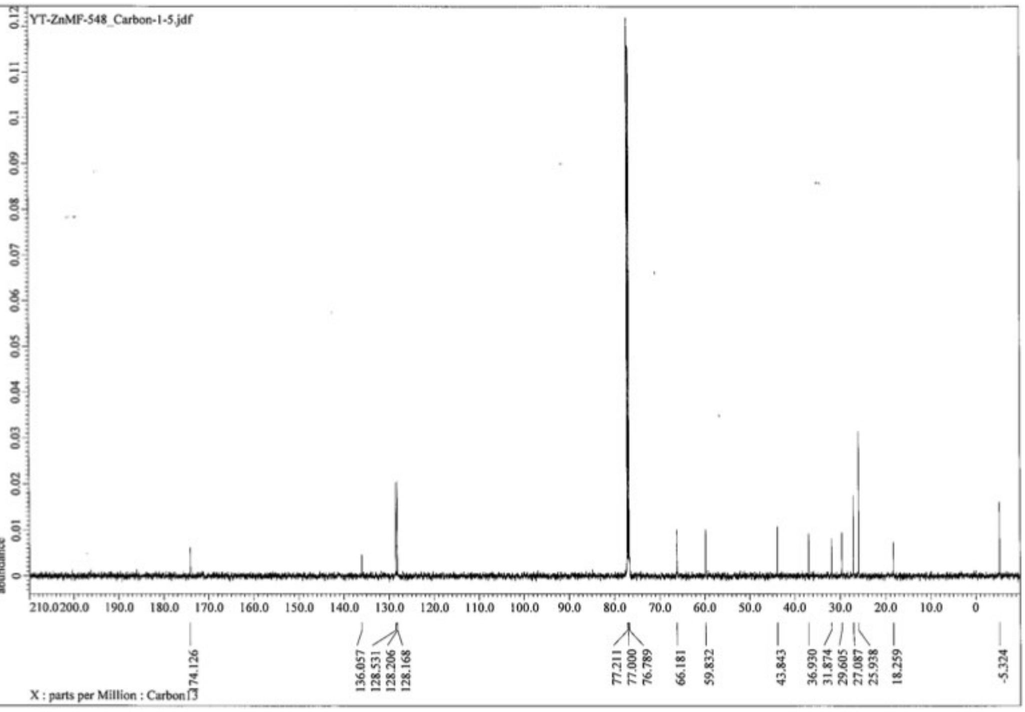

^{1}H-NMR (600 MHz, $CDCl_3$) of 3fa

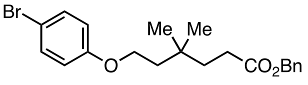

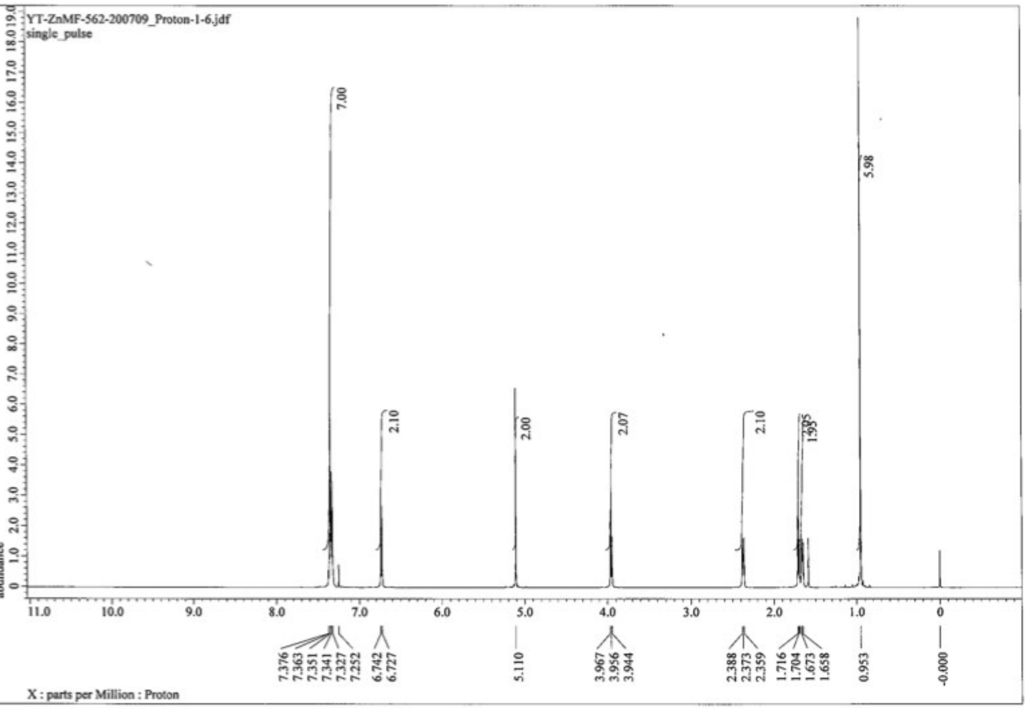

^{13}C-NMR (150 MHz, $CDCl_3$) of 3fa

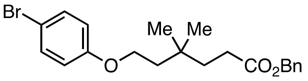

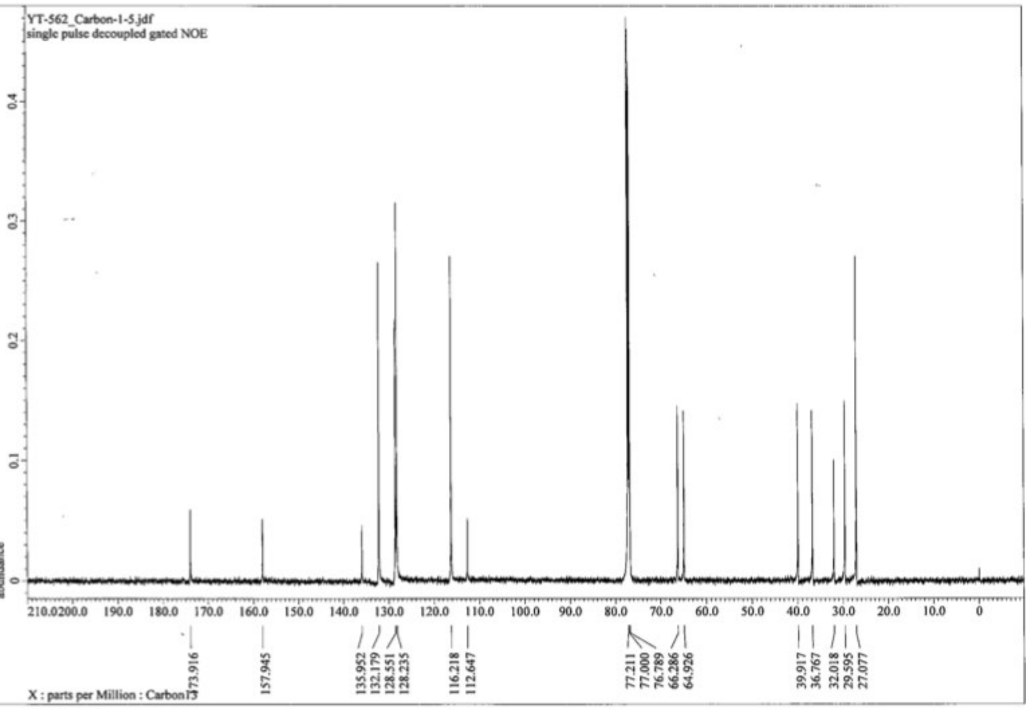

^{1}H-NMR (400 MHz, $CDCl_3$) of 3ga

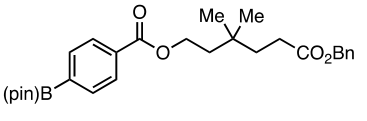

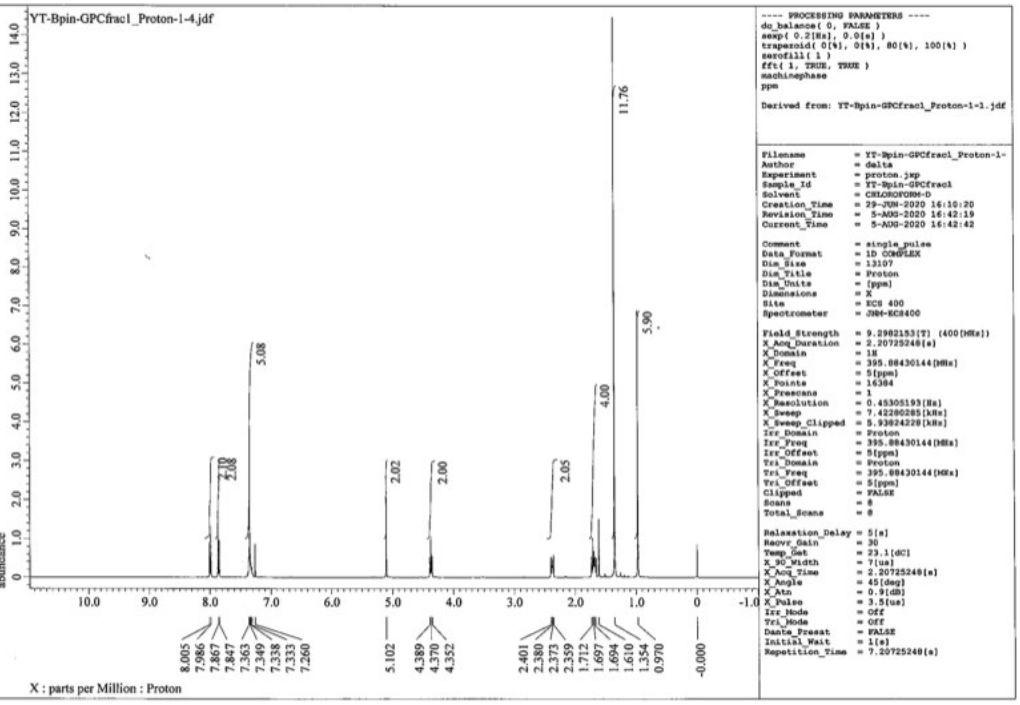

^{13}C-NMR (150 MHz, $CDCl_3$) of 3ga

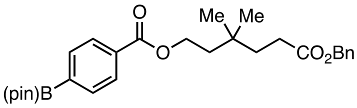

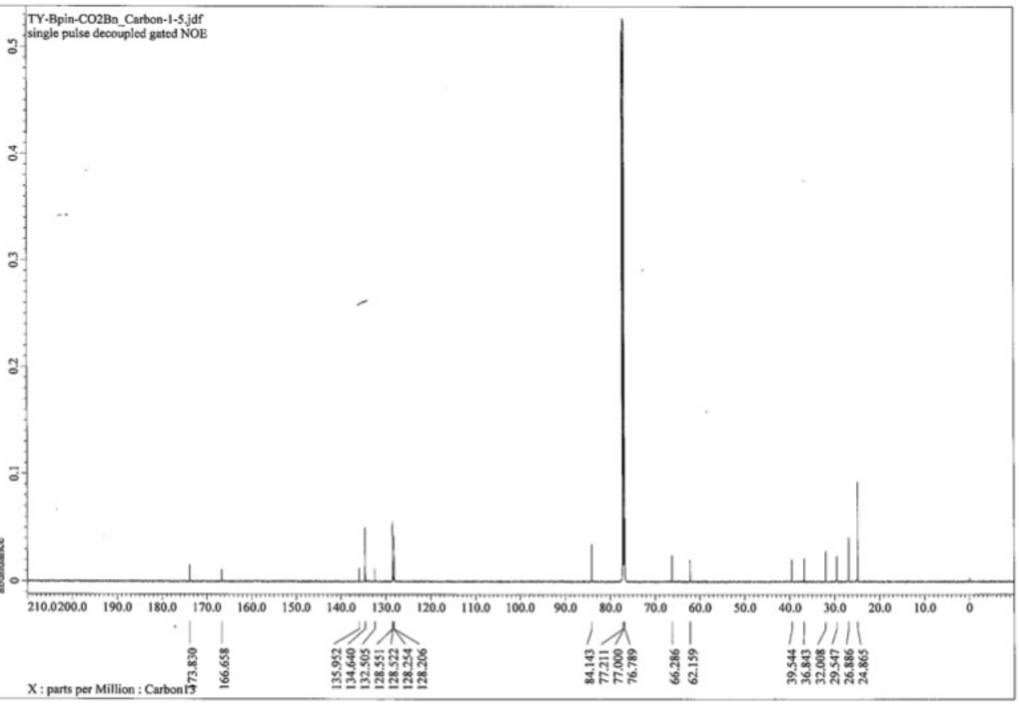

^{1}H-NMR (600 MHz, $CDCl_3$) of 3ha

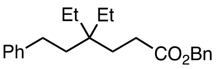

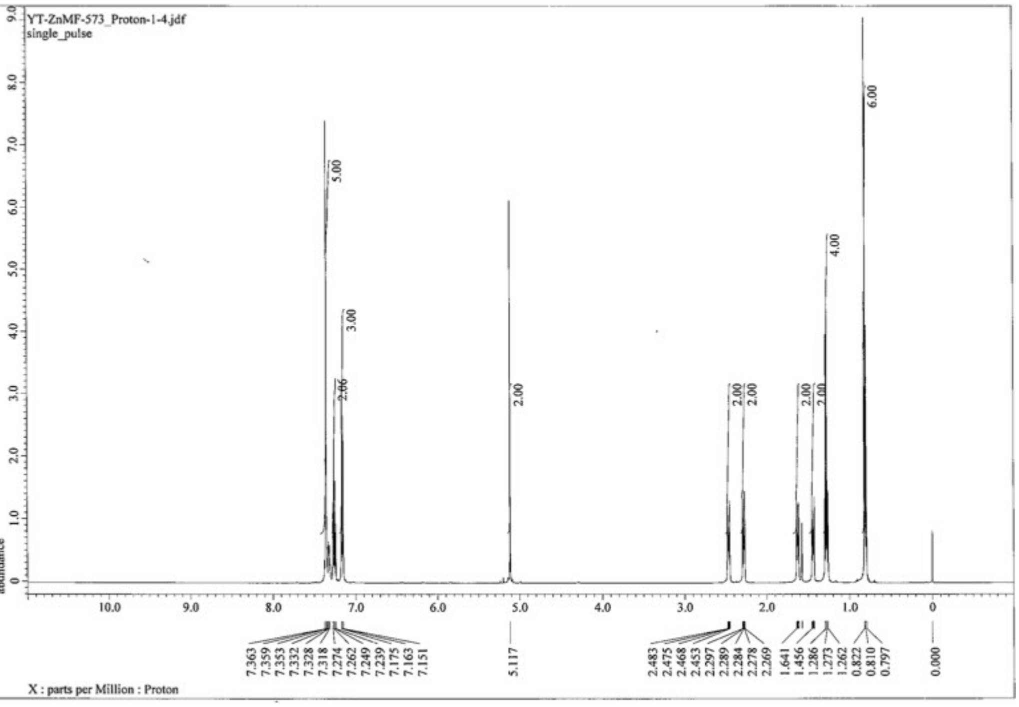

^{13}C-NMR (150 MHz, $CDCl_3$) of 3ha

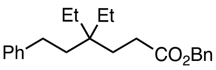

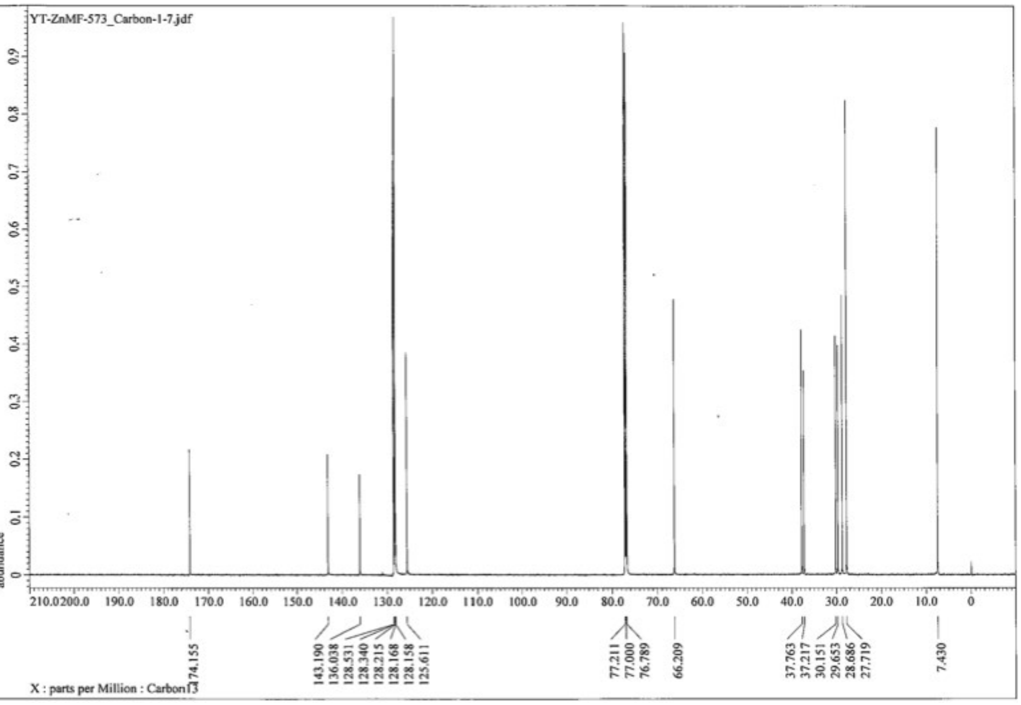
YT-ZnMF-573_Carbon-1-7.jdf
X : parts per Million : Carbon13
174.155
143.190
136.038
128.531
128.340
128.215
128.168
128.158
125.611
77.211
77.000
76.789
66.209
37.763
37.217
30.151
29.653
28.686
27.719
7.430

^{1}H-NMR (600 MHz, $CDCl_3$) of 3ia

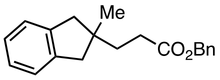

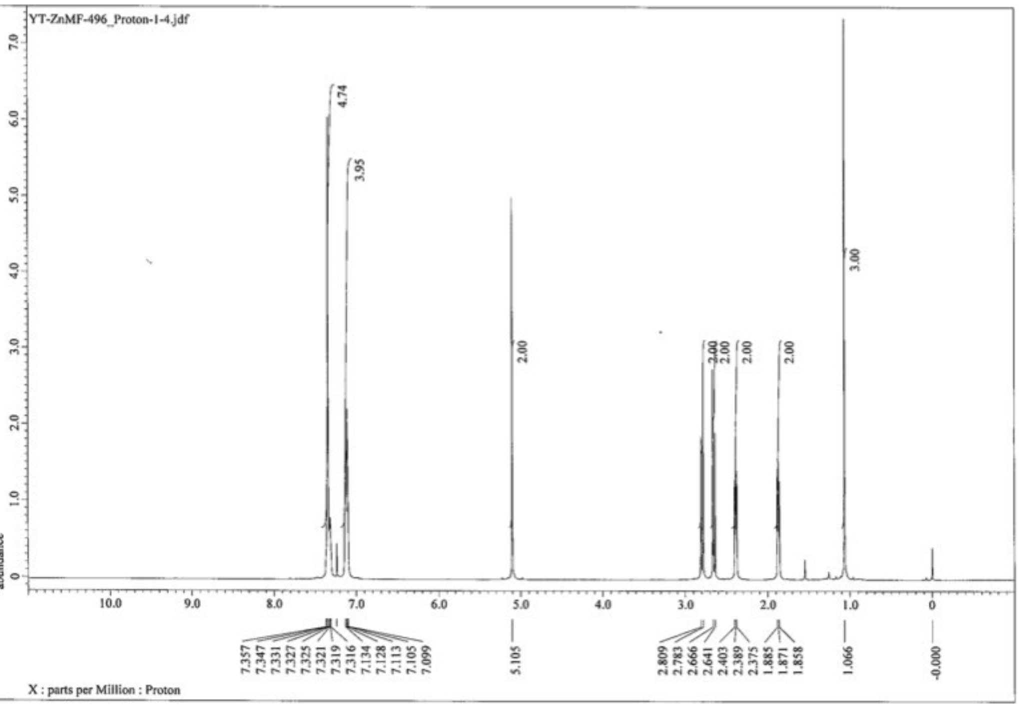

^{13}C-NMR (150 MHz, $CDCl_3$) of 3ia

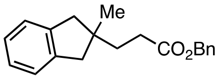

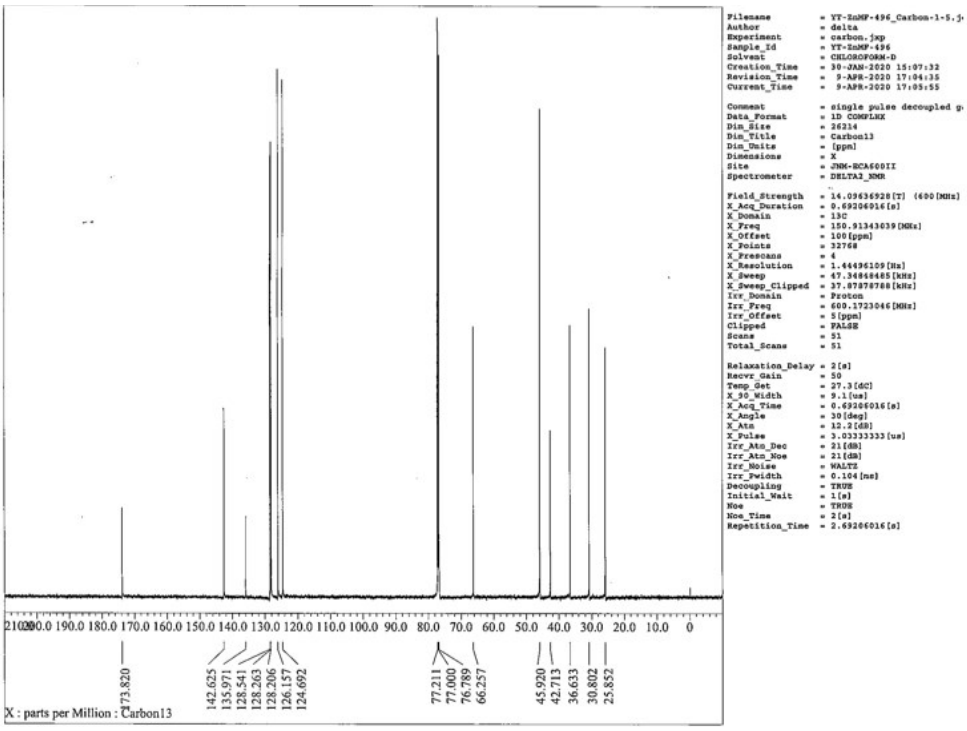

^{1}H-NMR (600 MHz, $CDCl_3$) of 3ja

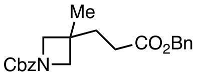

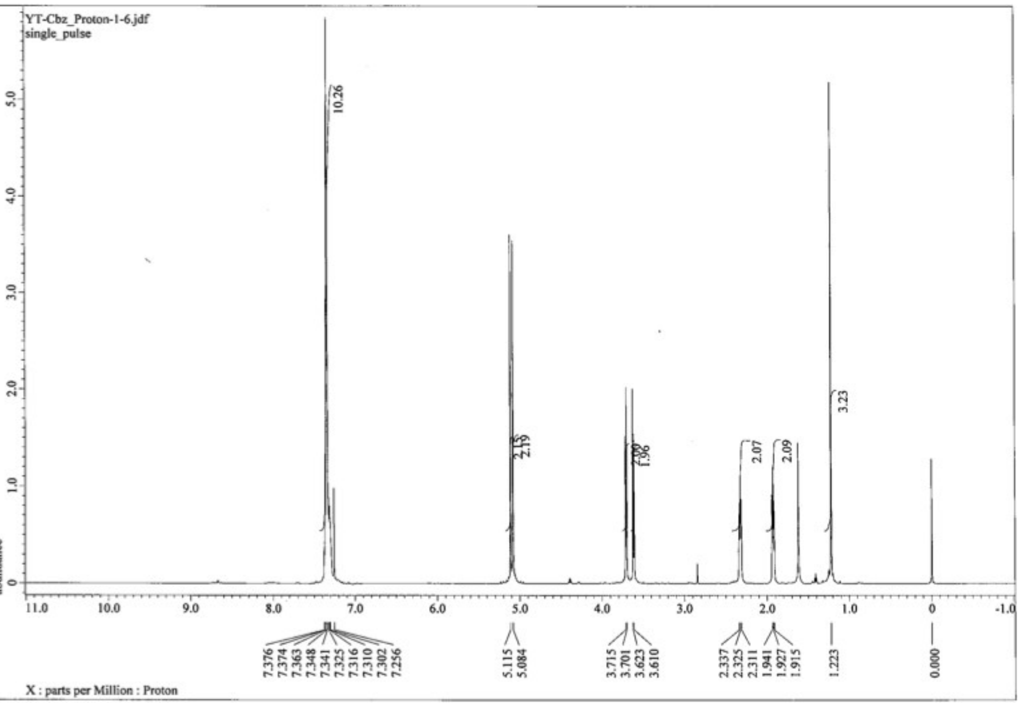

^{13}C-NMR (150 MHz, $CDCl_3$) of 3ja

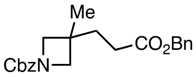

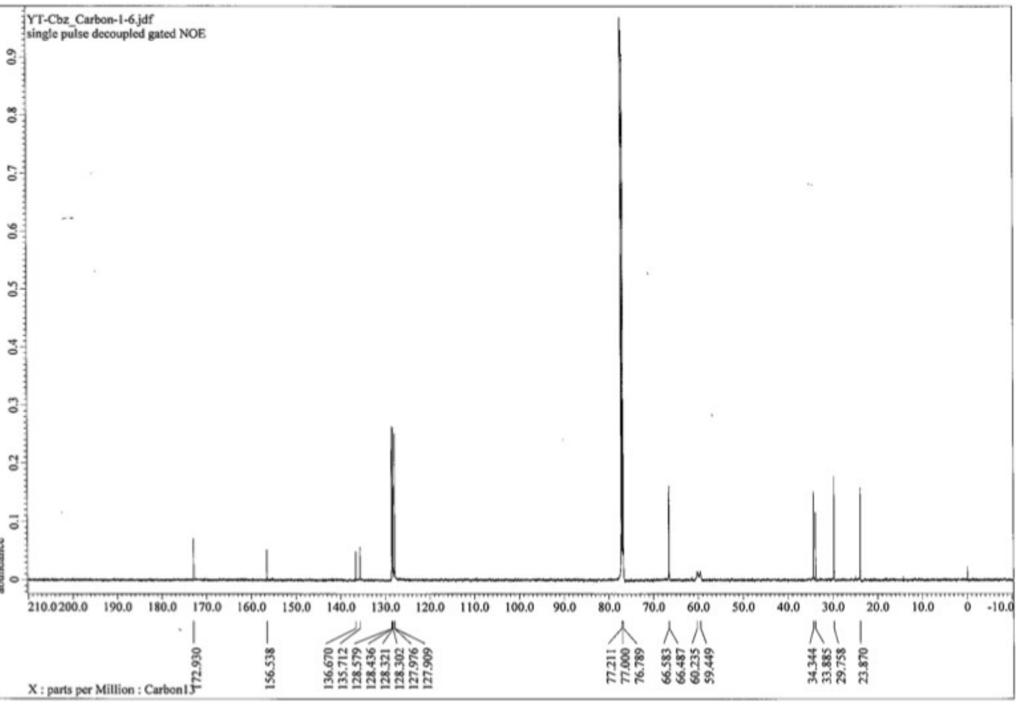
YT-Cbz_Carbon-1-6.jdf
single pulse decoupled gated NOE
X : parts per Million : Carbon13
172.930
156.538
136.670
135.712
128.579
128.436
128.321
128.302
127.976
127.909
77.211
77.000
76.789
66.583
66.487
60.235
59.449
34.344
33.885
29.758
23.870

^{1}H-NMR (600 MHz, CDCl$_3$) of 3ka

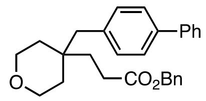

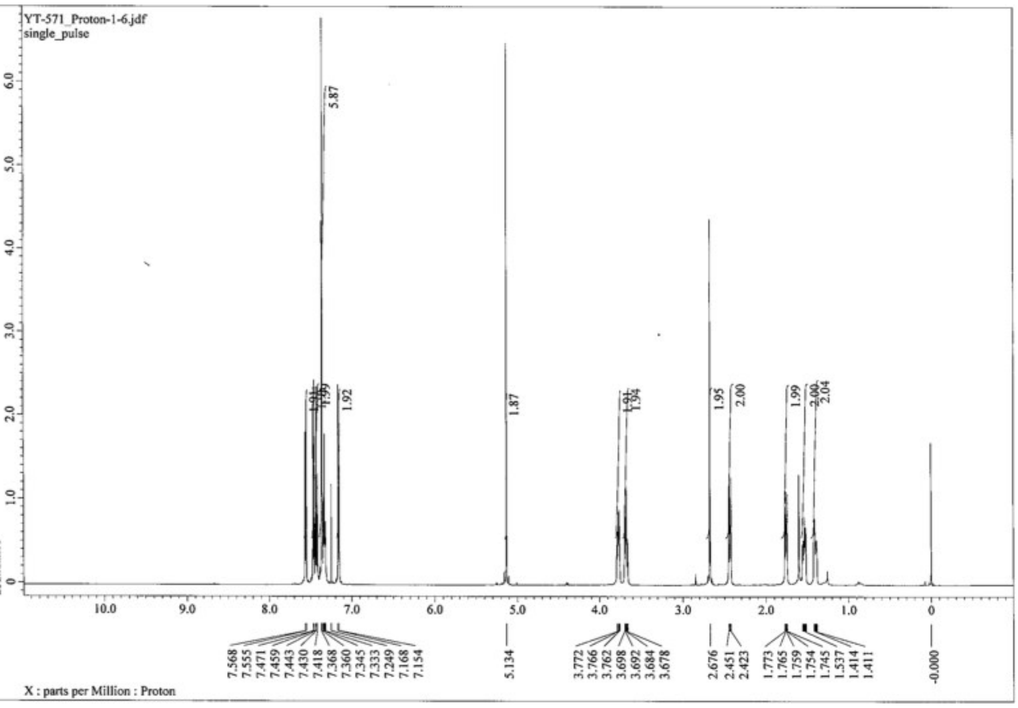

^{13}C-NMR (150 MHz, CDCl$_3$) of 3ka

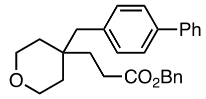

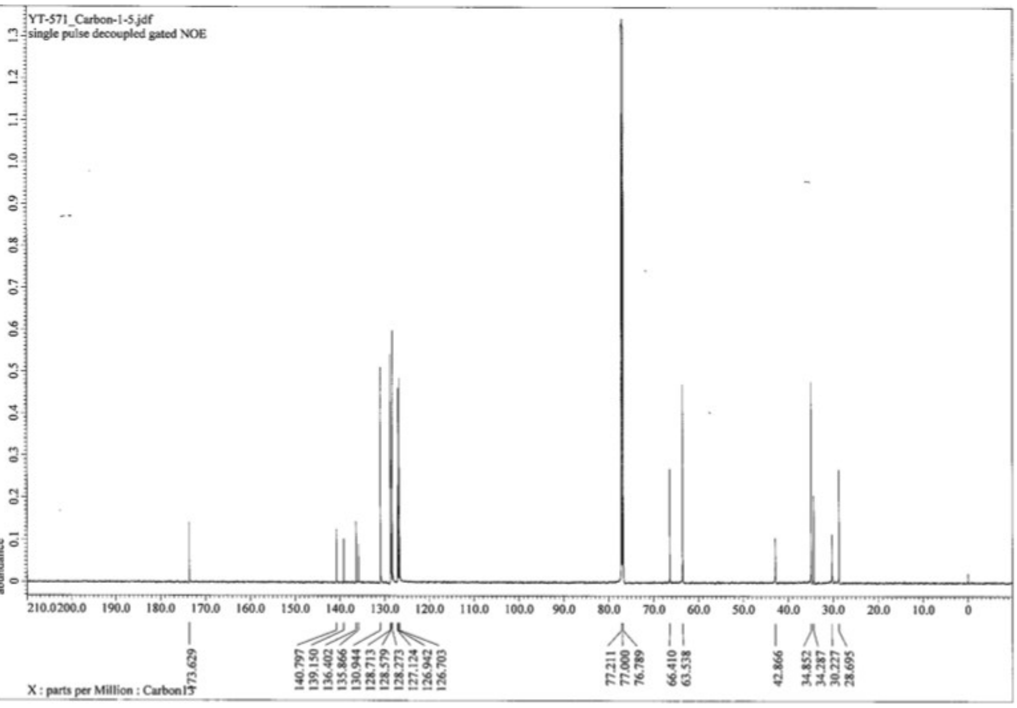

^{1}H-NMR (600 MHz, $CDCl_3$) of 3la

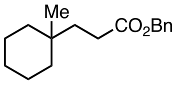

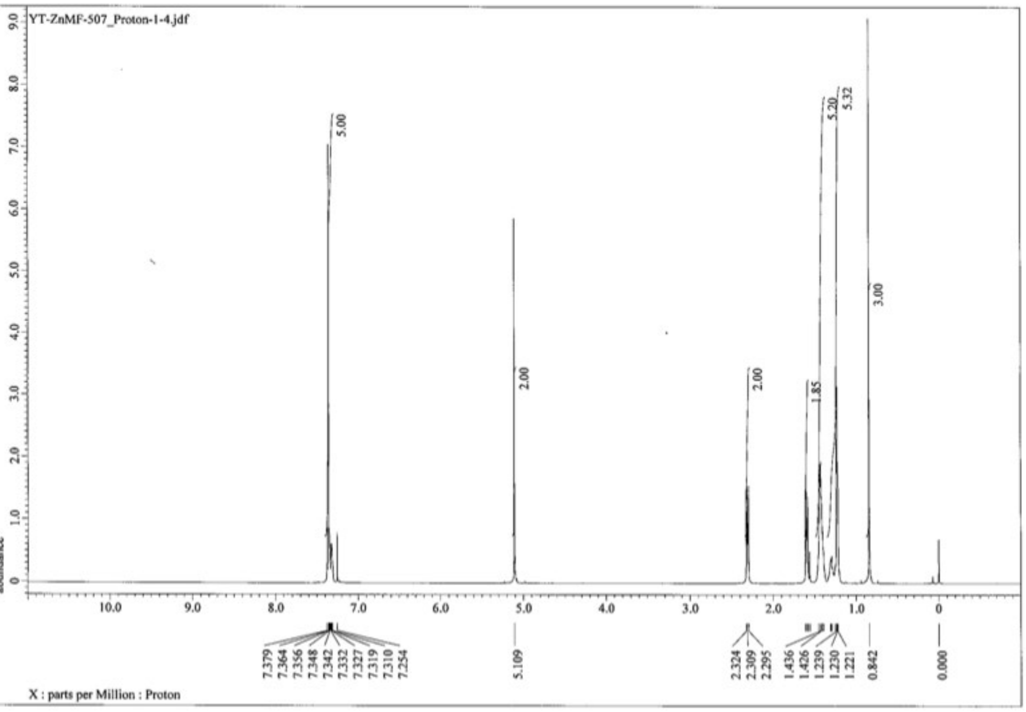

^{13}C-NMR (150 MHz, $CDCl_3$) of 3la

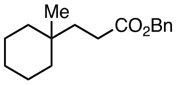

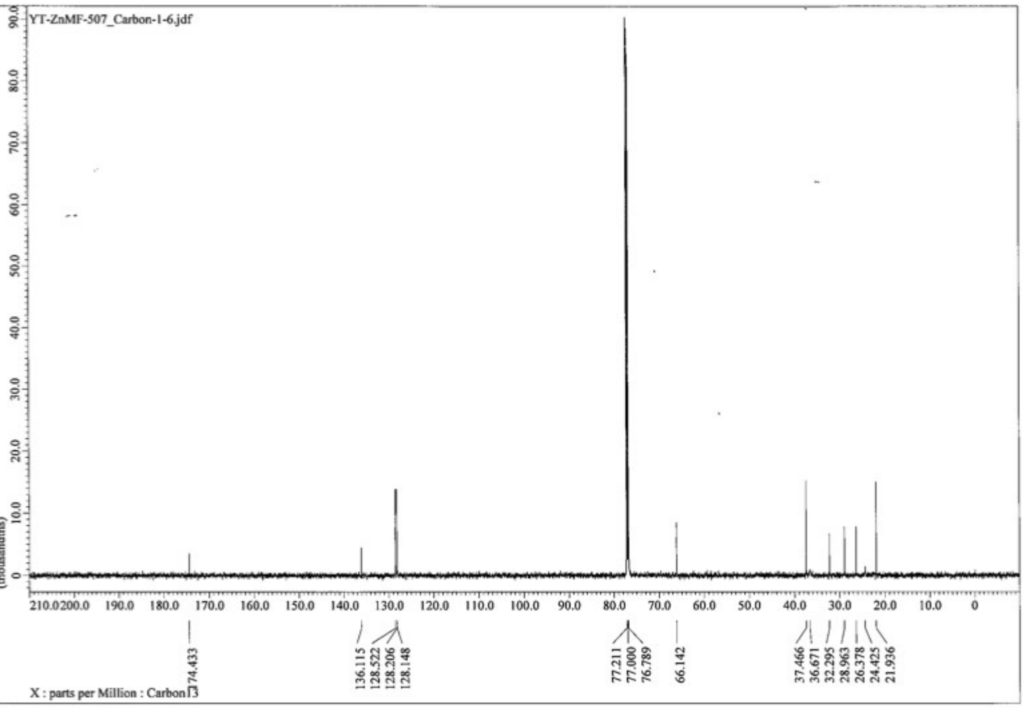
YT-ZnMF-507_Carbon-1-6.jdf
X : parts per Million : Carbon13
174.433
136.115
128.522
128.206
128.148
77.211
77.000
76.789
66.142
37.466
36.671
32.295
28.963
26.378
24.425
21.936

^{1}H-NMR (600 MHz, $CDCl_3$) of 3ma

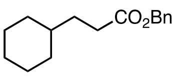

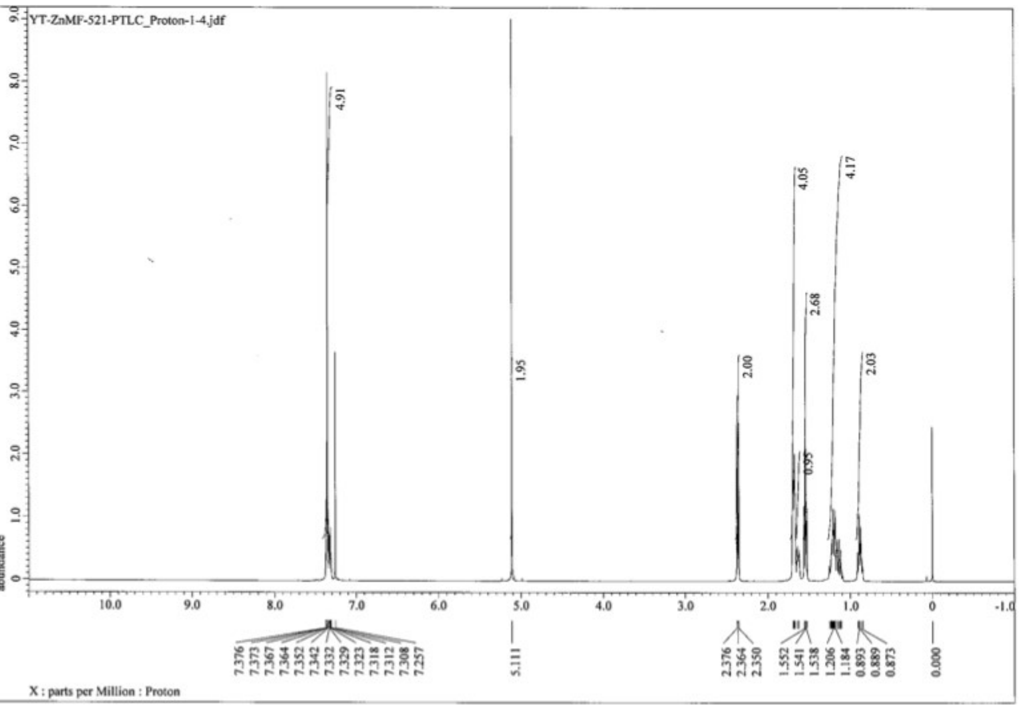

^{13}C-NMR (150 MHz, $CDCl_3$) of 3ma

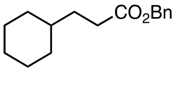

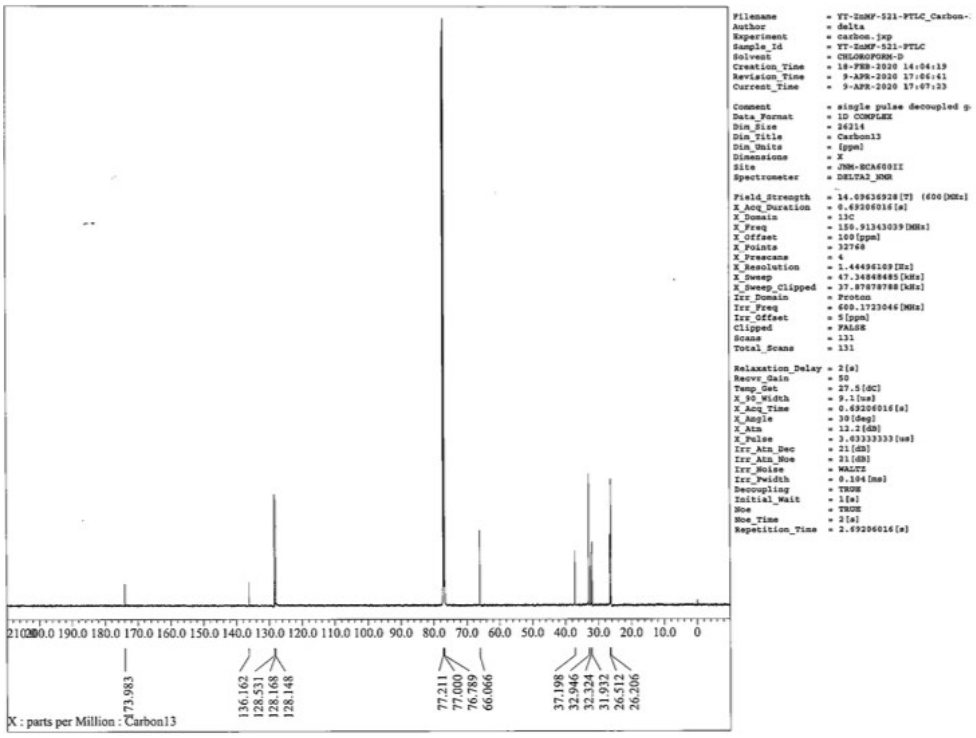

^{1}H-NMR (500 MHz, $CDCl_3$) of 11a

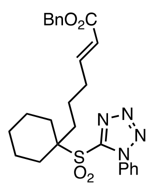

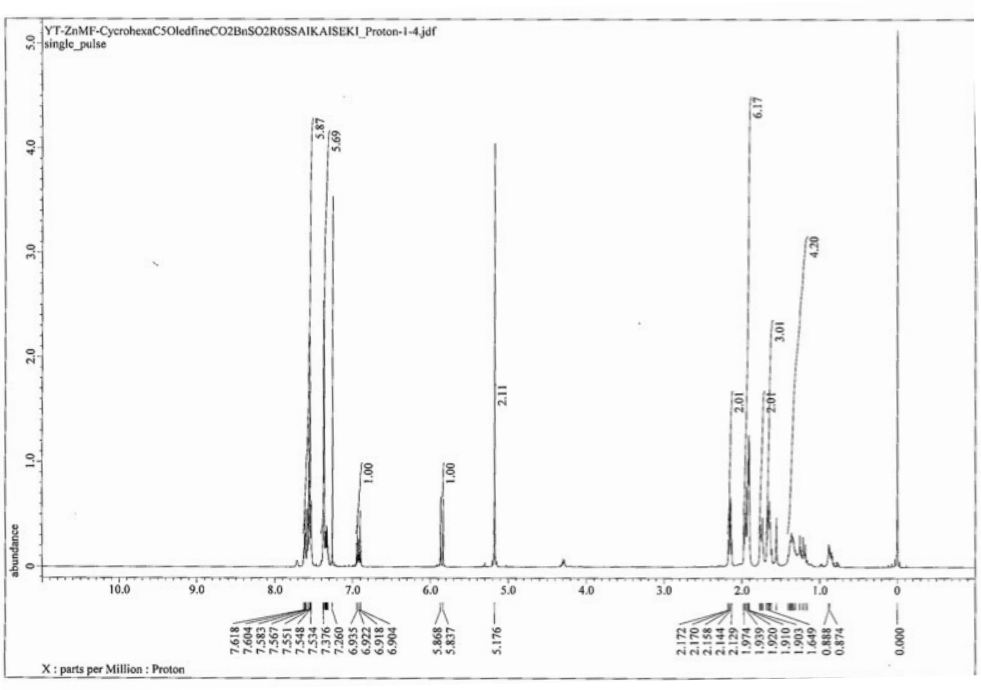

^{13}C-NMR (125 MHz, $CDCl_3$) of 11a

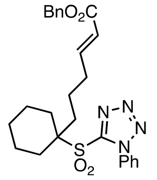

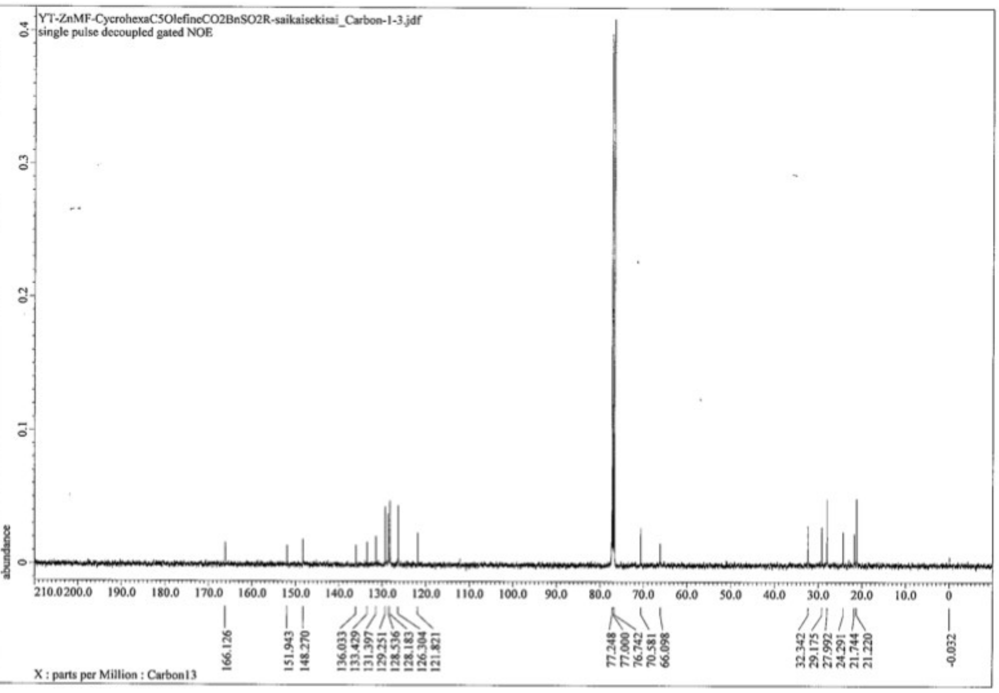

^{1}H-NMR (600 MHz, $CDCl_3$) of 11b

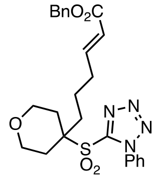

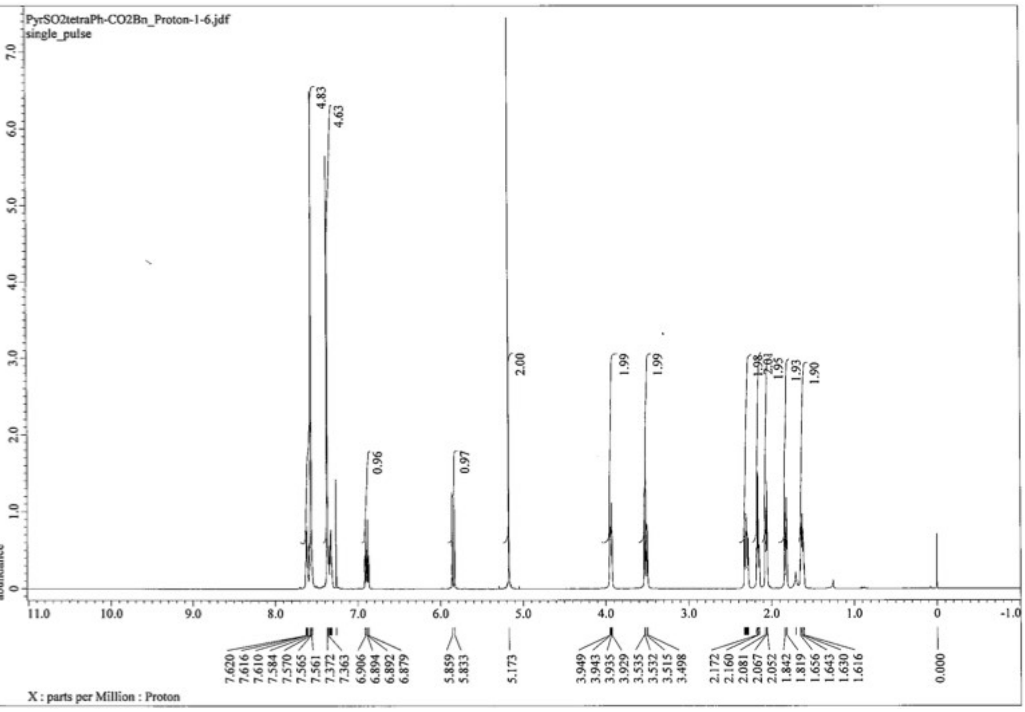

^{13}C-NMR (150 MHz, $CDCl_3$) of 11b

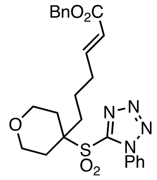

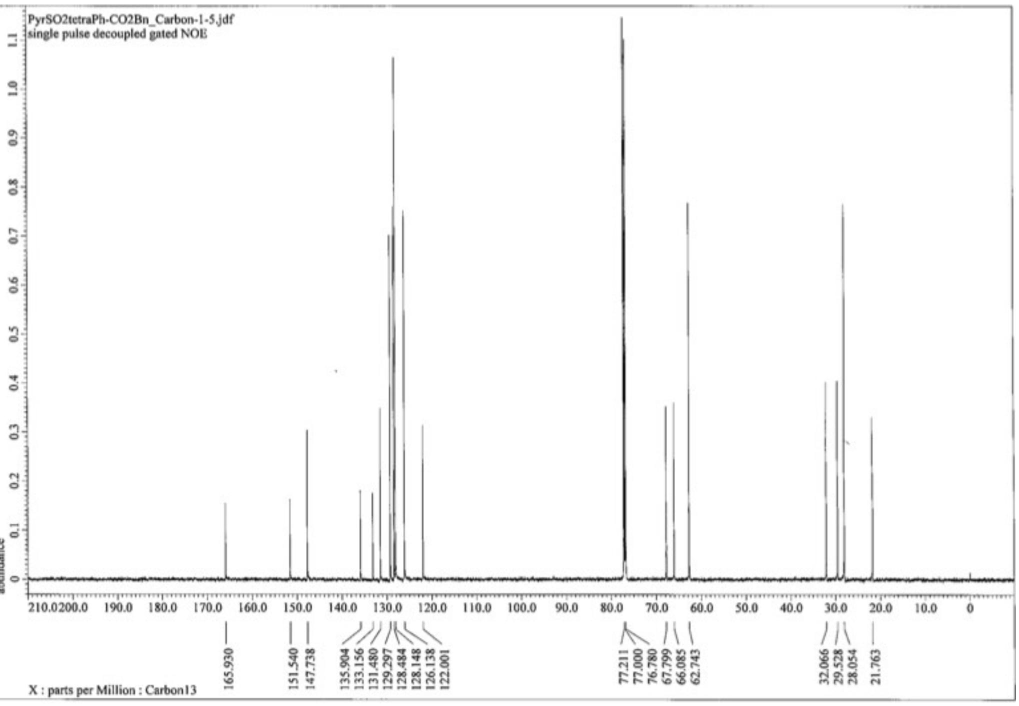

¹H-NMR (500 MHz, $CDCl_3$) of 11c

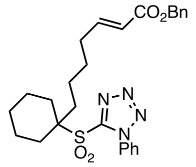

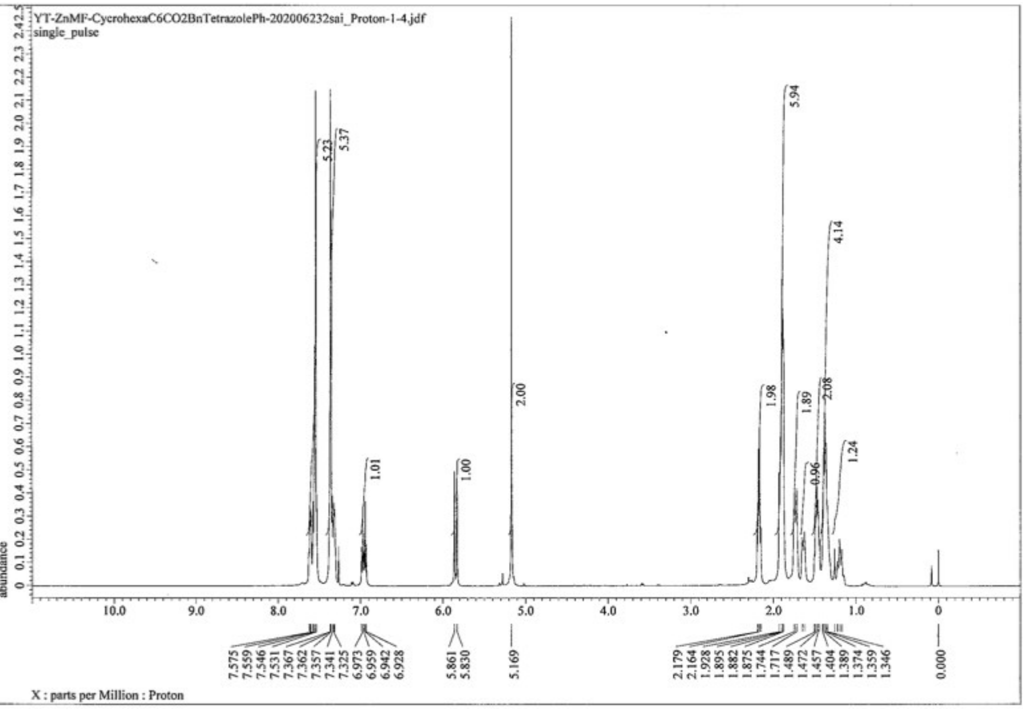

^{13}C-NMR (125 MHz, $CDCl_3$) of 11c

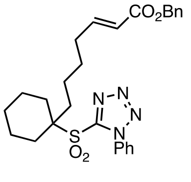

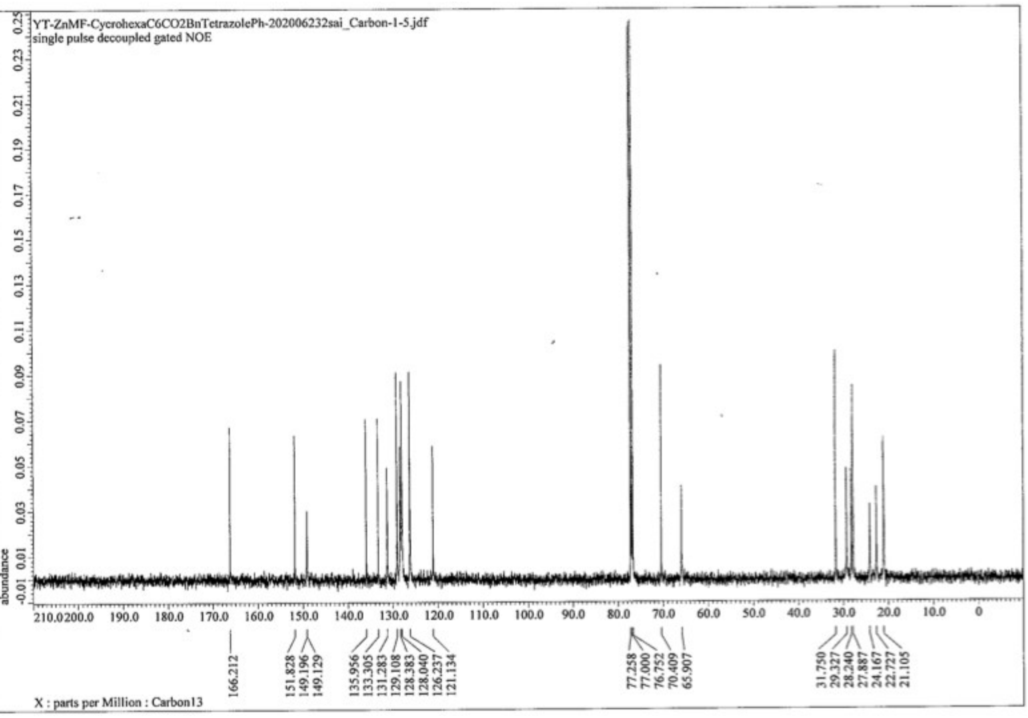

^{1}H-NMR (600 MHz, $CDCl_3$) of 11d

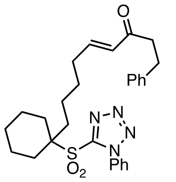

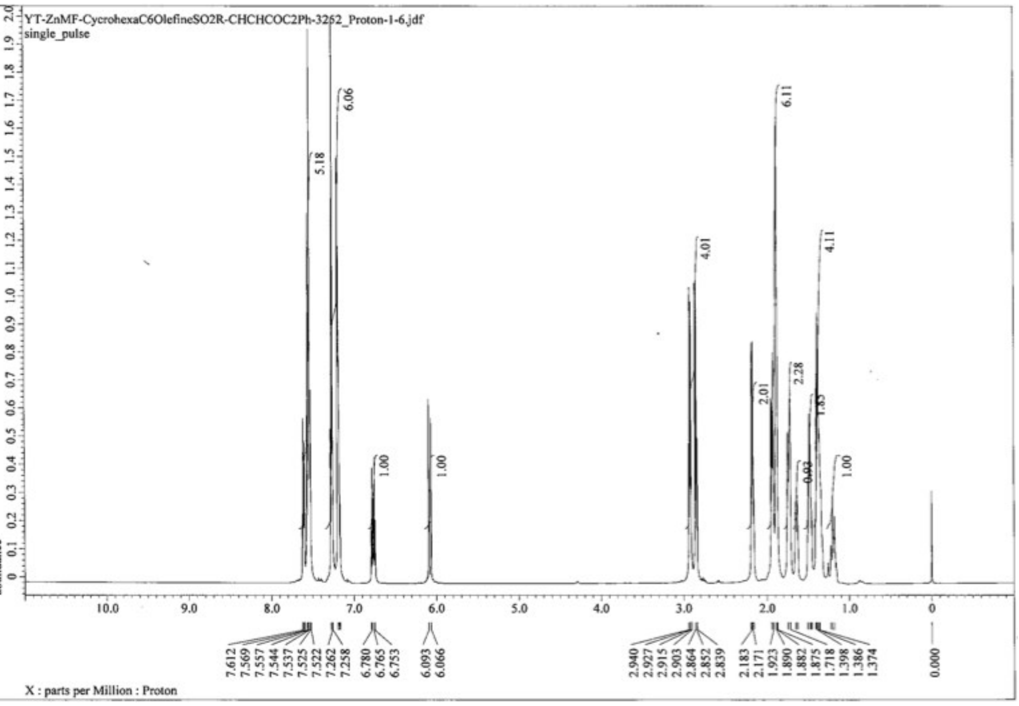

^{13}C-NMR (150 MHz, $CDCl_3$) of 11d

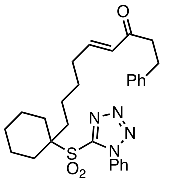

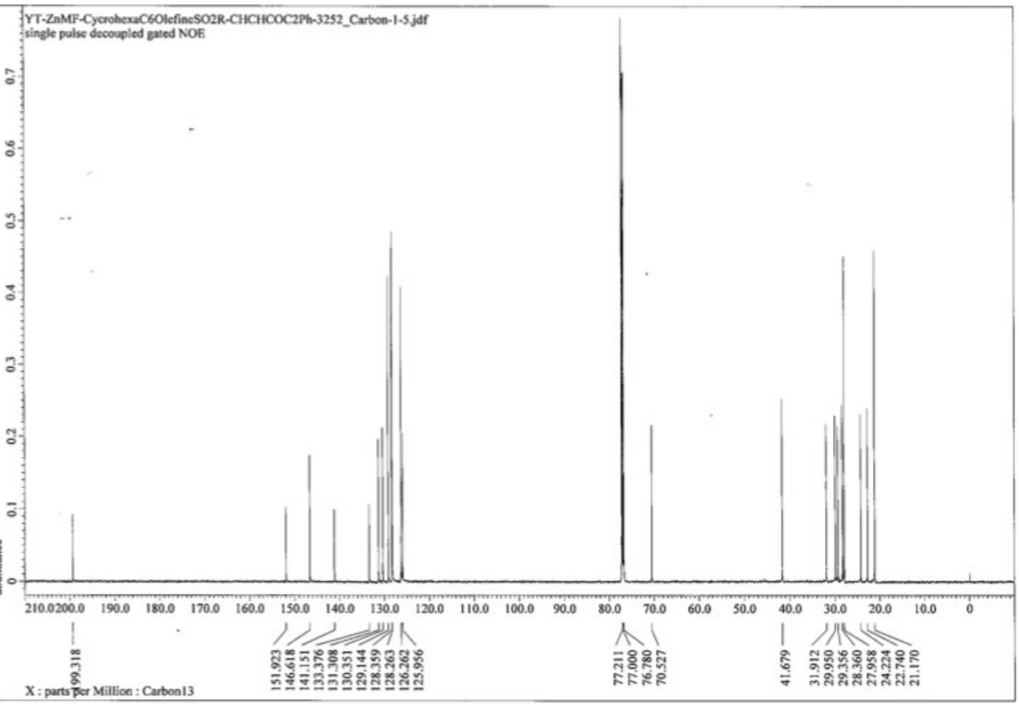

^{1}H-NMR (600 MHz, $CDCl_3$) of 12a

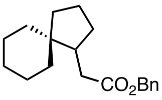

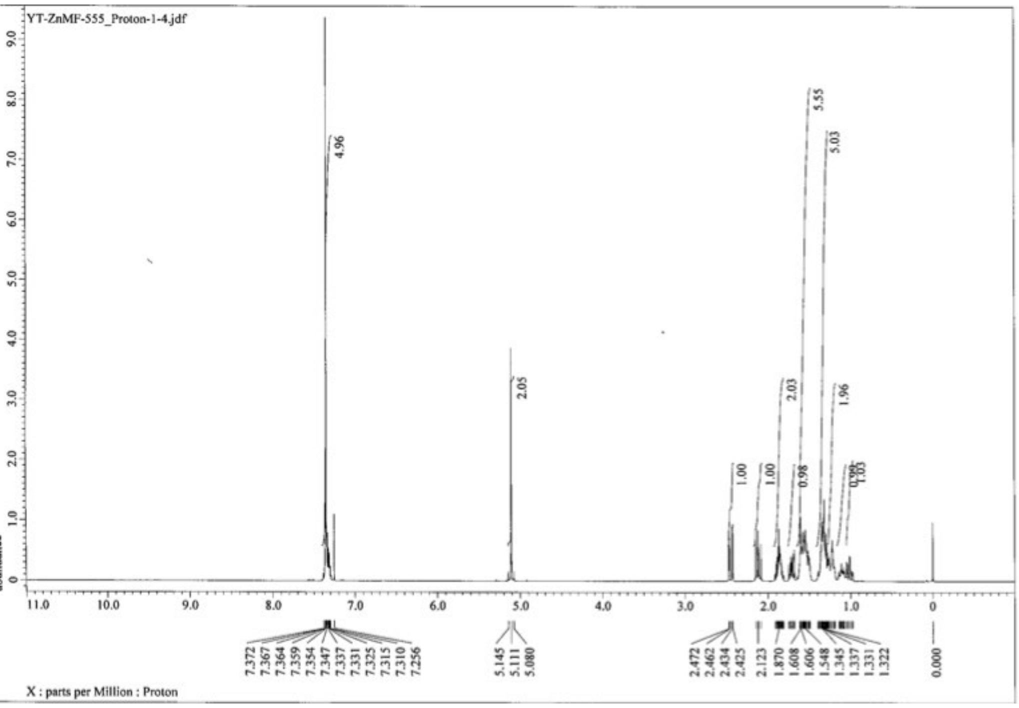

^{13}C-NMR (150 MHz, $CDCl_3$) of 12a

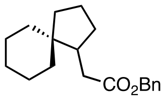

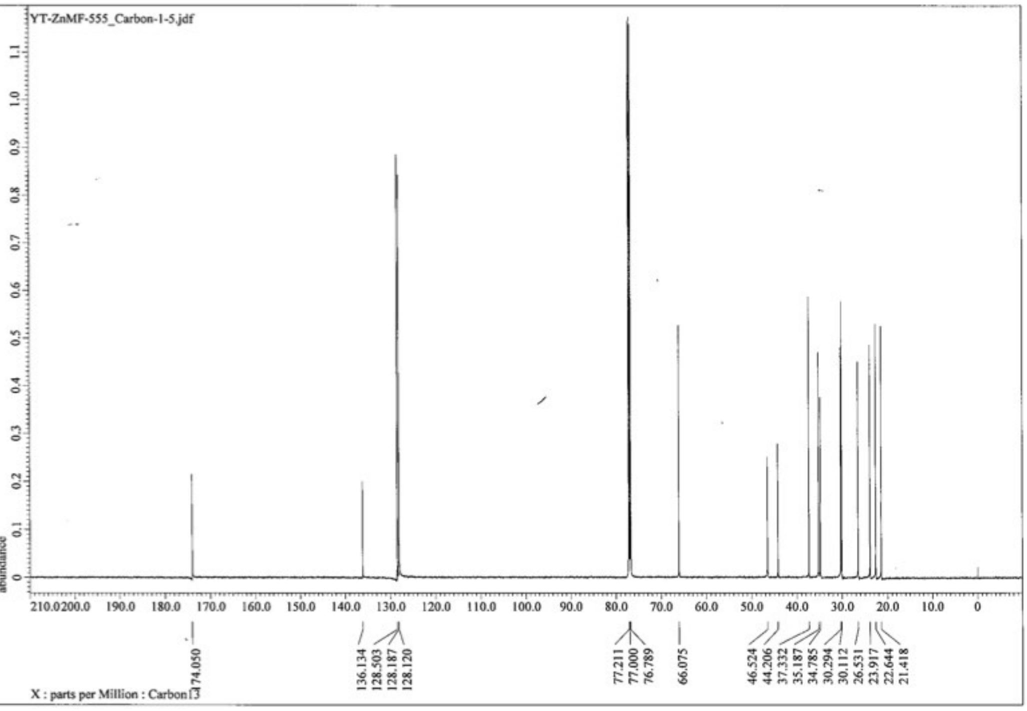
YT-ZnMF-555_Carbon-1-5.jdf
abundance
174.050
136.134
128.503
128.187
128.120
77.211
77.000
76.789
66.075
46.524
44.206
37.332
35.187
34.785
30.294
30.112
26.531
23.917
22.644
21.418
X : parts per Million : Carbon13

^{1}H-NMR (600 MHz, $CDCl_3$) of 12b

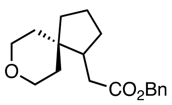

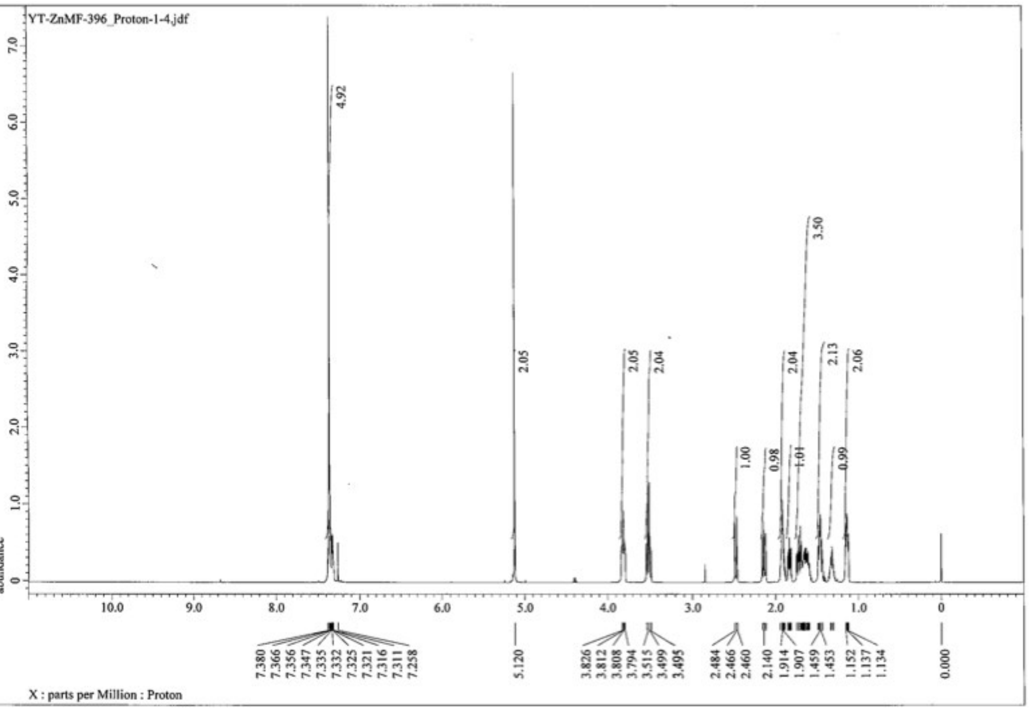

^{13}C-NMR (150 MHz, $CDCl_3$) of 12b

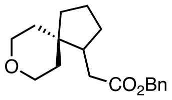

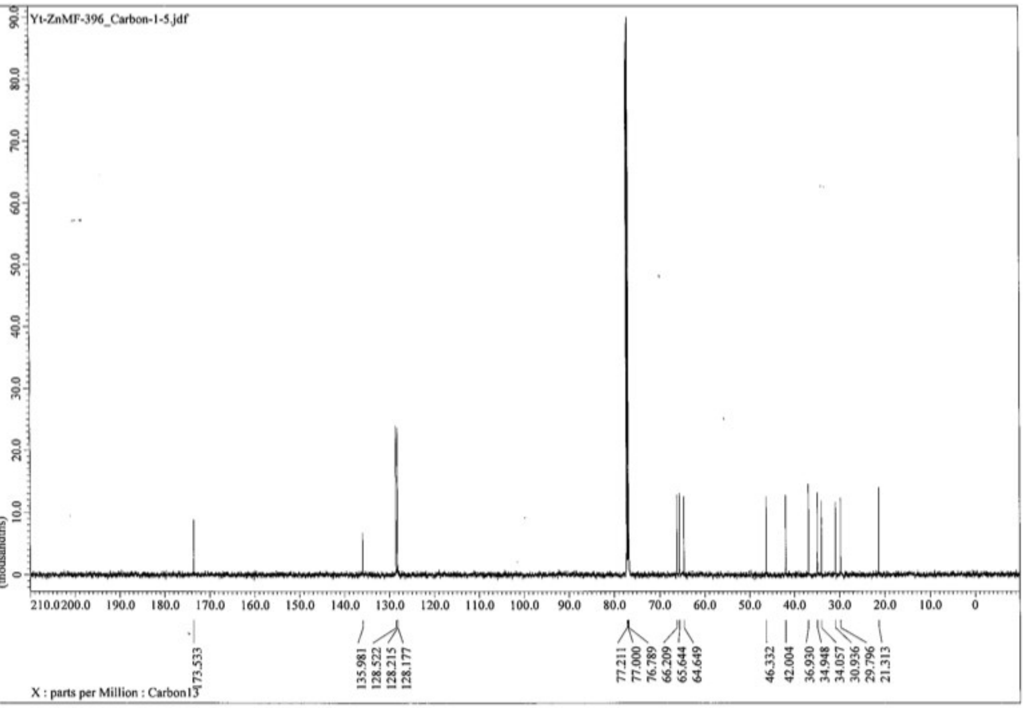
Yt-ZnMF-396_Carbon-1-5.jdf
173.533
135.981
128.522
128.215
128.177
77.211
77.000
76.789
66.209
65.644
64.649
46.332
42.004
36.930
34.948
34.057
30.936
29.796
21.313
X : parts per Million : Carbon13

^{1}H-NMR (600 MHz, $CDCl_3$) of 12c

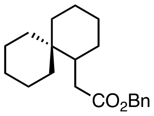

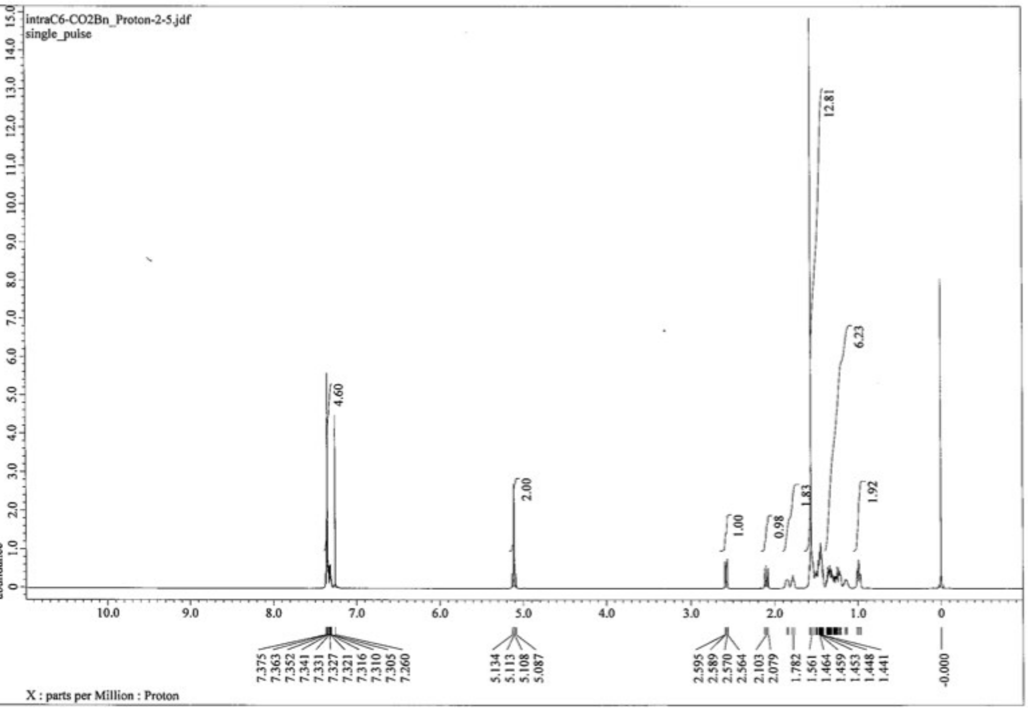

^{13}C-NMR (150 MHz, $CDCl_3$) of 12c

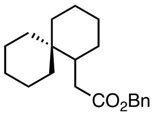

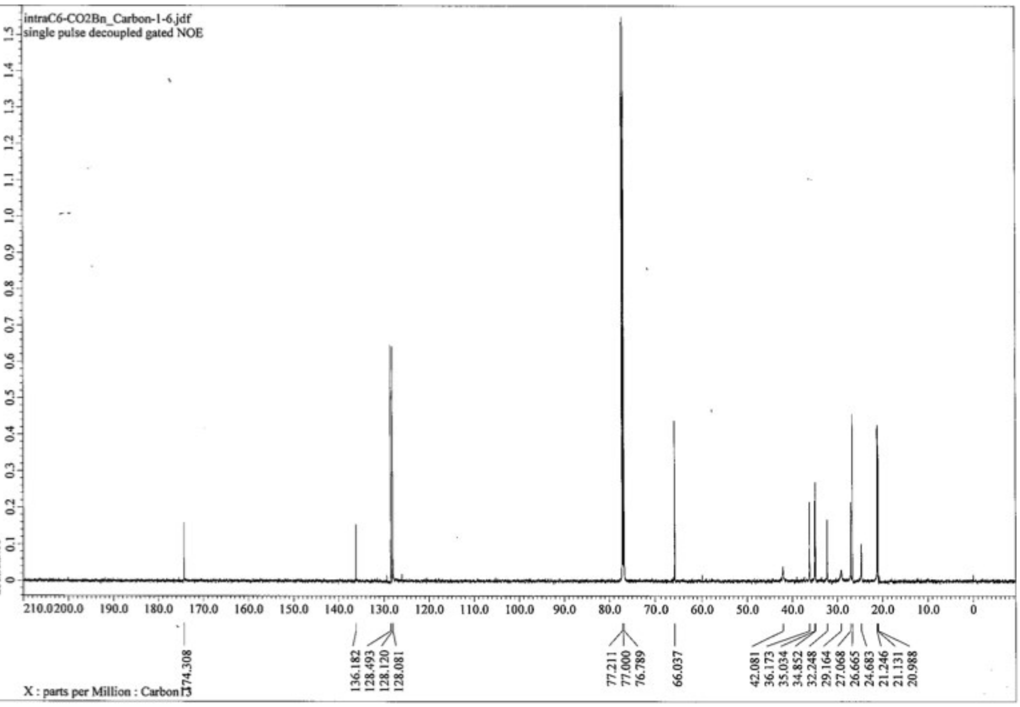
intraC6-CO2Bn_Carbon-1-6.jdf
single pulse decoupled gated NOE
174.308
136.182
128.493
128.120
128.081
77.211
77.000
76.789
66.037
42.081
36.173
35.034
34.852
32.248
29.164
27.068
26.665
24.683
21.246
21.131
20.988
X : parts per Million : Carbon13

^{1}H-NMR (600 MHz, $CDCl_3$) of 12d

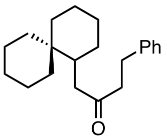

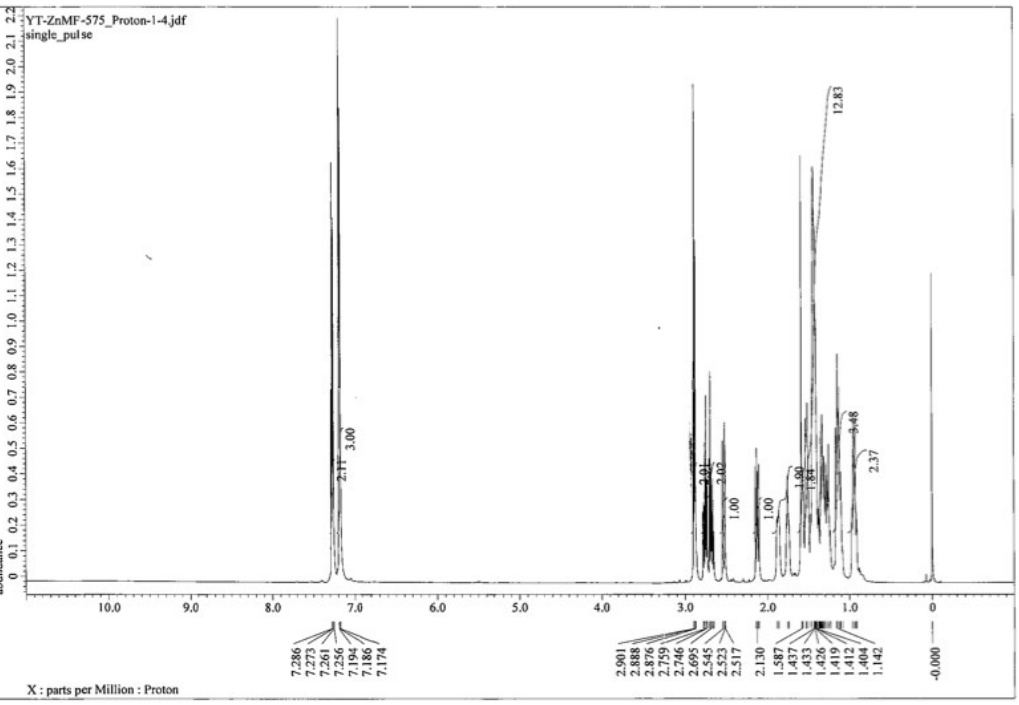

^{13}C-NMR (150 MHz, $CDCl_3$) of 12d

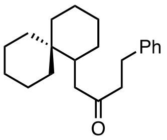

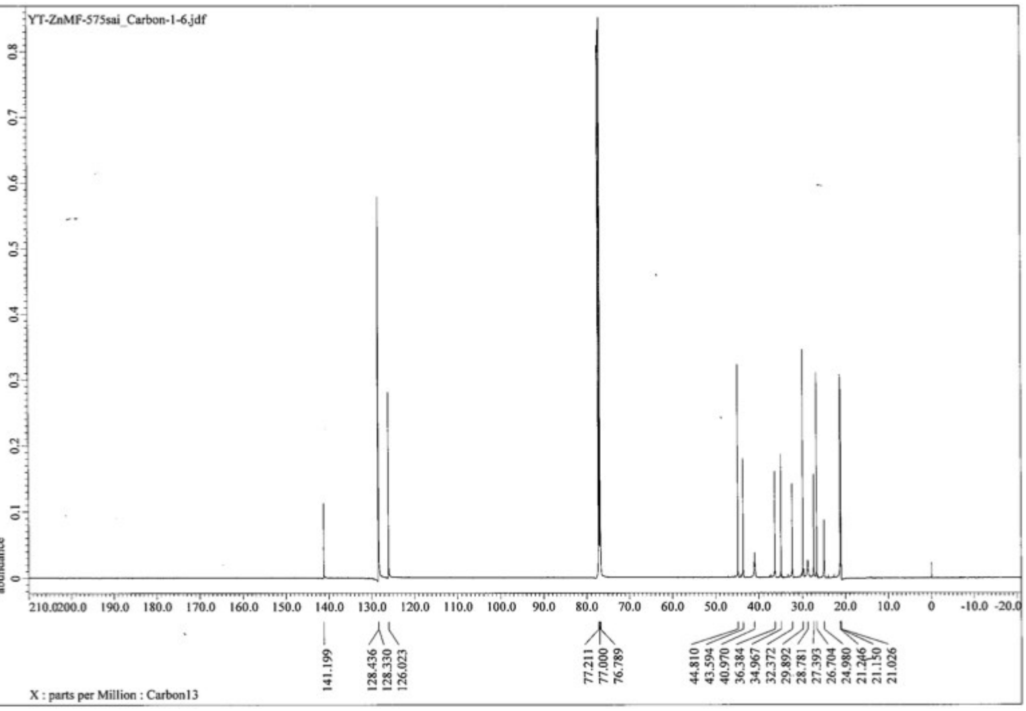

^{1}H-NMR (600 MHz, $CDCl_3$) of *t*-butyl ester

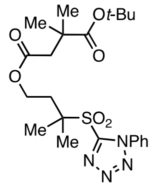

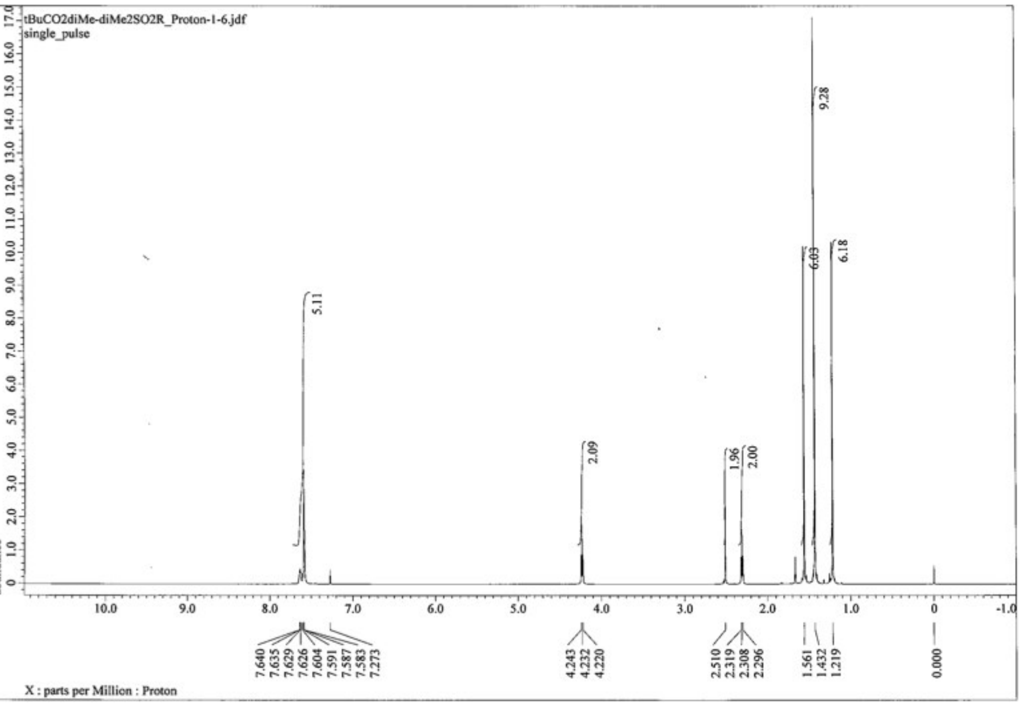

^{13}C-NMR (150 MHz, $CDCl_3$) of *t*-butyl ester

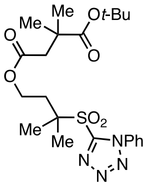

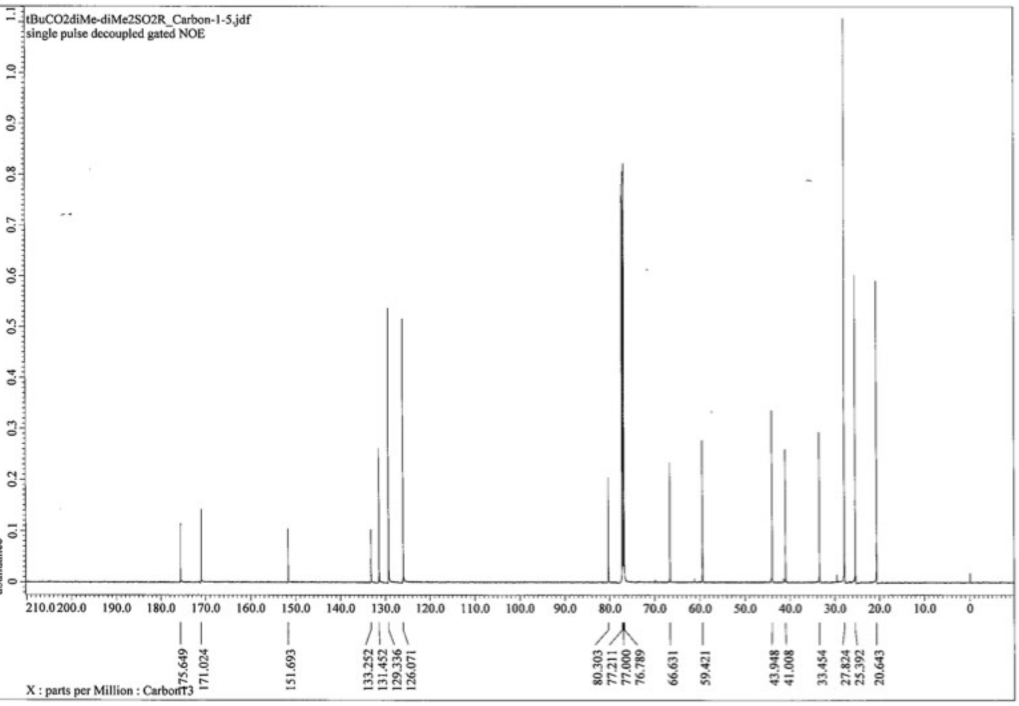

^{1}H-NMR (600 MHz, $CDCl_3$) of 13

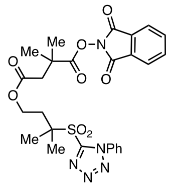

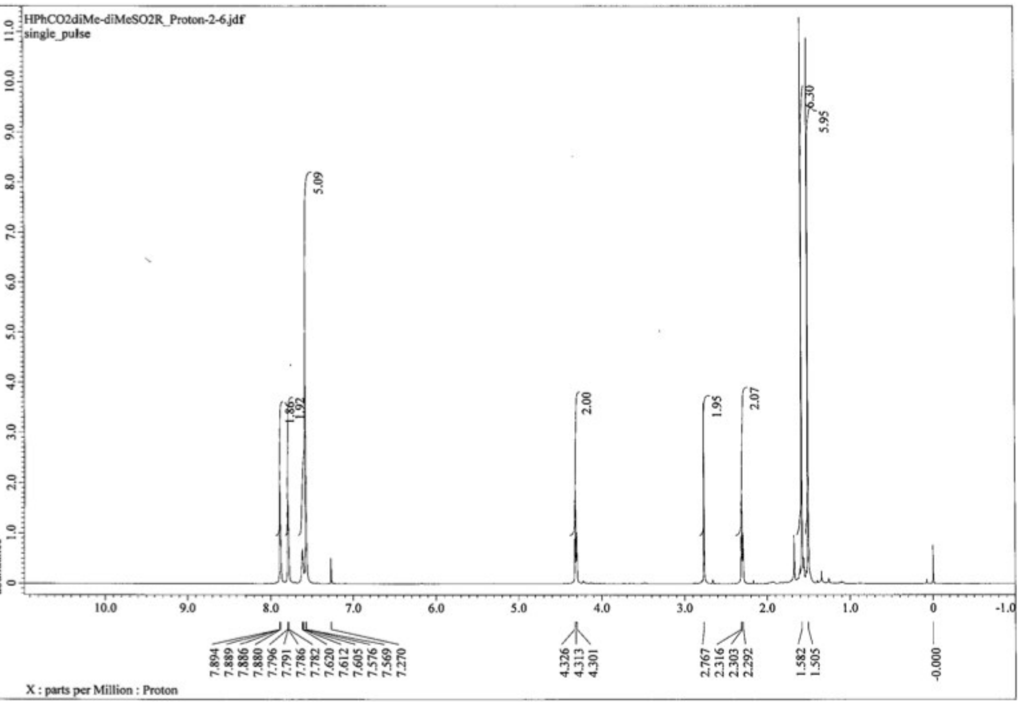

^{13}C-NMR (150 MHz, $CDCl_3$) of 13

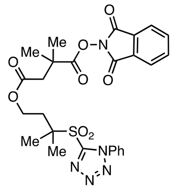

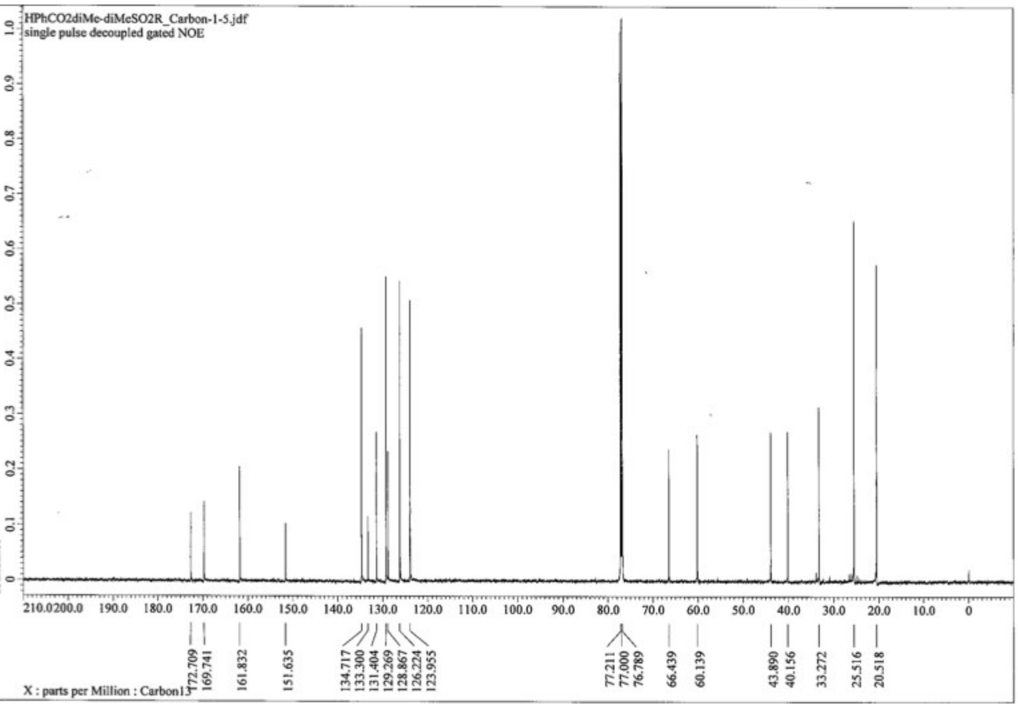

¹H-NMR (600 MHz, $CDCl_3$) of 14

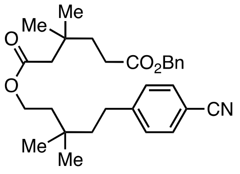

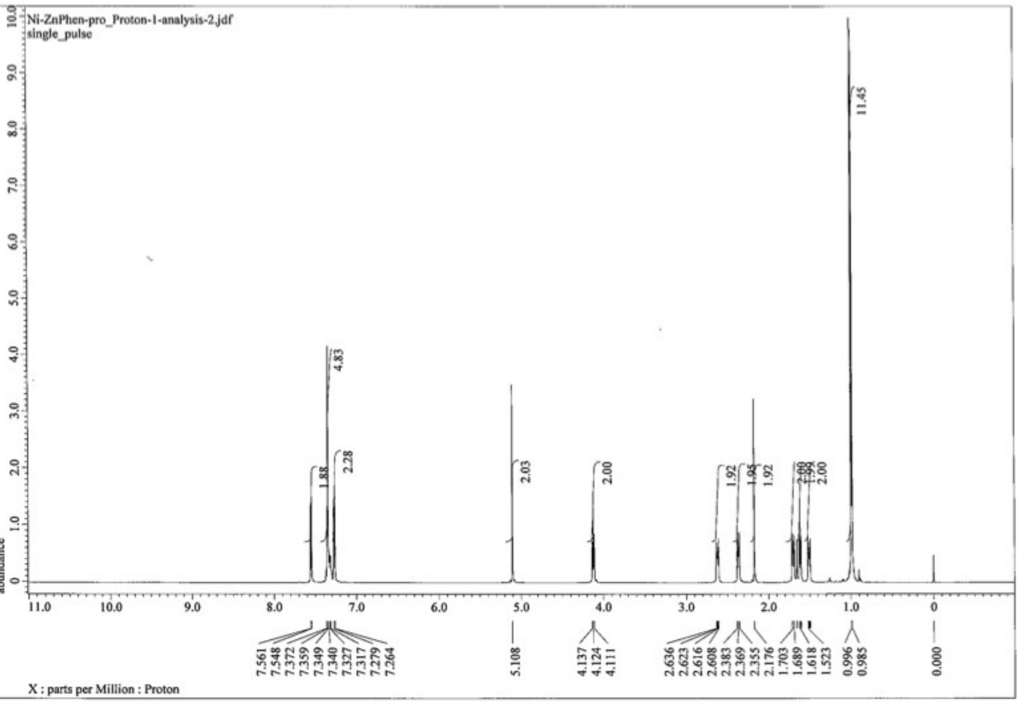

^{13}C-NMR (150 MHz, $CDCl_3$) of 14

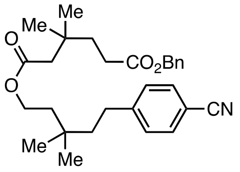

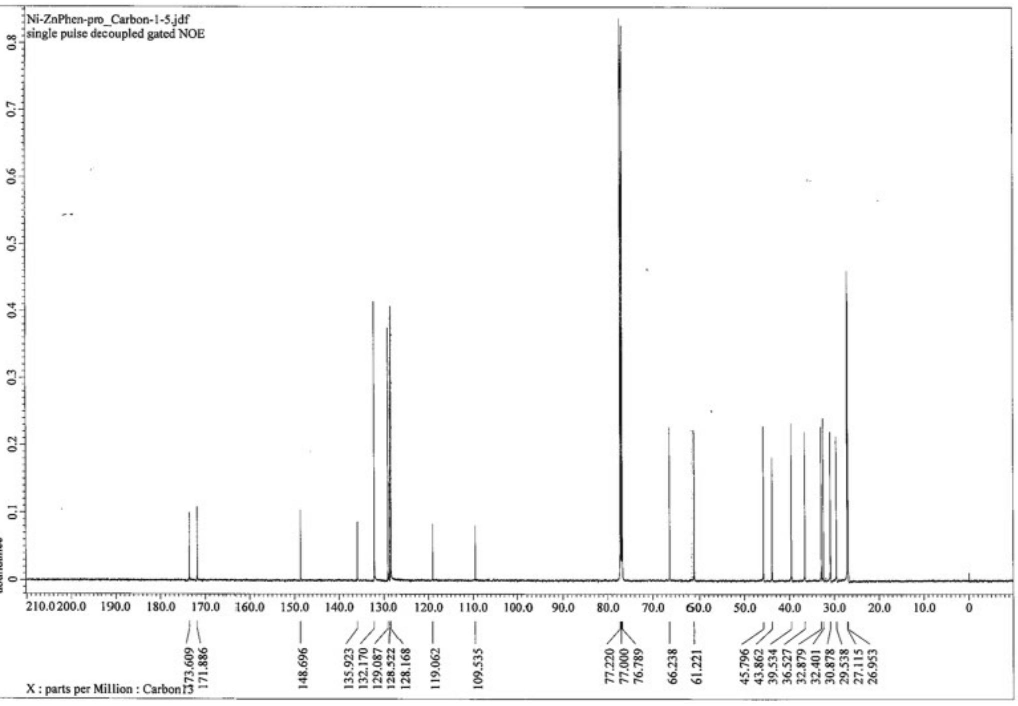

12. Coordinates of calculated structures

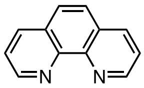

C	-3.47711400	-0.36819000	-0.00000500
C	-2.82021000	0.83858700	-0.00000300
C	-1.41128700	0.86165200	-0.00000100
C	-0.72931800	-0.37719200	-0.00000100
C	-2.70648400	-1.54386900	-0.00000500
C	-0.67669200	2.09543500	0.00000000
C	0.72931800	-0.37719200	0.00000100
C	1.41128700	0.86165200	0.00000200
C	0.67669200	2.09543500	0.00000200
C	2.82021000	0.83858700	0.00000400
H	3.36987300	1.77570400	0.00000500
C	3.47711400	-0.36819000	0.00000500
C	2.70648400	-1.54386900	0.00000400
H	-1.23246800	3.02866200	0.00000000
H	-4.56017500	-0.42613700	-0.00000700
H	-3.36987300	1.77570400	-0.00000300
H	-3.19946800	-2.51358900	-0.00000700
H	1.23246800	3.02866200	0.00000300
H	4.56017500	-0.42613700	0.00000600
H	3.19946800	-2.51358900	0.00000500
N	-1.38668300	-1.55759700	-0.00000300

N	1.38668300	-1.55759700	0.00000200

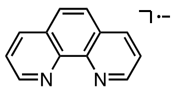

C	-3.49654400	-0.38277400	-0.00000500
C	-2.85346000	0.83474400	-0.00000500
C	-1.42427800	0.89035400	-0.00000200
C	-0.73941500	-0.37904200	-0.00000100
C	-2.73307300	-1.55786400	-0.00000300
C	-0.70222500	2.09266800	-0.00000100
C	0.73941500	-0.37904200	0.00000100
C	1.42427800	0.89035400	0.00000300
C	0.70222500	2.09266800	0.00000200
C	2.85346000	0.83474400	0.00000500
H	3.41781400	1.76440100	0.00000700
C	3.49654400	-0.38277400	0.00000500
C	2.73307300	-1.55786400	0.00000200
H	-1.24903200	3.03307100	-0.00000200
H	-4.58189500	-0.44094600	-0.00000700
H	-3.41781400	1.76440100	-0.00000600
H	-3.21058900	-2.53477700	-0.00000300
H	1.24903200	3.03307100	0.00000300
H	4.58189500	-0.44094600	0.00000700
H	3.21058900	-2.53477700	0.00000100
N	-1.38992400	-1.54432500	-0.00000100
N	1.38992400	-1.54432500	0.00000000

$[Zn(phen)_3]^{2+}$

C	-2.88177600	-1.84674600	2.90052500
C	-4.09747200	-1.48921800	2.36597600
C	-4.13938800	-0.72093700	1.18513800
C	-2.91115300	-0.35506600	0.60666900
C	-1.70918300	-1.42842800	2.25487100
C	-5.36683300	-0.30510800	0.56669400
C	-2.90152600	0.42415300	-0.60875100
C	-4.12032900	0.82081000	-1.18684500
C	-5.35765200	0.43685600	-0.56745600
C	-4.05950300	1.58637300	-2.36863500
H	-4.97963900	1.91175500	-2.84437600
C	-2.83540100	1.91126000	-2.90484000
C	-1.67348600	1.46401600	-2.25924700
H	-6.30369400	-0.59723100	1.03049000
H	-2.81280300	-2.43793000	3.80620300
H	-5.02532200	-1.79125600	2.84210800
H	-0.73487400	-1.68865600	2.65533400
H	-6.28698300	0.75270400	-1.03083800
H	-2.75189700	2.49890700	-3.81159200
H	-0.69308100	1.69845800	-2.66075300
N	-1.72047100	-0.70420000	1.14766300
N	-1.70260800	0.74242400	-1.15065400
Zn	-0.00309900	-0.00007400	-0.00069400

N	1.47088900	-1.12733000	1.14758100
C	1.76578000	-2.33427600	0.61035600
C	2.09584100	-0.74890800	2.25069500
C	2.70368000	-3.20854200	1.18756700
C	1.08015700	-2.72436300	-0.59884600
C	3.05091700	-1.54865700	2.89493000
H	1.83215000	0.22544100	2.64880200
C	3.35236300	-2.78073700	2.36338500
C	2.95955700	-4.48082600	0.57305500
C	1.34709100	-3.98026400	-1.17204600
N	0.19736600	-1.85191700	-1.13911200
H	3.53099400	-1.18776500	3.79703800
H	4.08340100	-3.42835800	2.83786500
C	2.30675500	-4.85215400	-0.55509300
H	3.68720100	-5.14046300	1.03504800
C	0.64544200	-4.32062000	-2.34602900
C	-0.45041900	-2.19747200	-2.23986900
H	2.49930000	-5.81607900	-1.01535100
H	0.82417500	-5.28229900	-2.81735600
C	-0.25698100	-3.43042900	-2.87956100
H	-1.15096700	-1.47182000	-2.63975800
H	-0.81496300	-3.66002200	-3.77980500
N	1.50158800	1.09235700	-1.14637400
C	1.82334200	2.29224000	-0.60868000
C	2.12125900	0.69886100	-2.24713900
C	2.78324000	3.14400100	-1.18339200
C	1.14400900	2.69852000	0.59868200
C	3.09697300	1.47550000	-2.88872700
H	1.83635100	-0.26938500	-2.64556500
C	3.42542800	2.70054500	-2.35698800
C	3.06713800	4.41017300	-0.56857100
C	1.43906100	3.94782600	1.17253900
N	0.23978200	1.84700800	1.13697700
H	3.57143800	1.10271400	-3.78896300
H	4.17298800	3.33058000	-2.82940900
C	2.42028300	4.79697200	0.55778900
H	3.81110100	5.05255300	-1.02887400
C	0.74327100	4.30448100	2.34516100
C	-0.40168600	2.20754500	2.23665000
H	2.63427100	5.75618000	1.01838100
H	0.94347700	5.26173800	2.81689700
C	-0.18064000	3.43555500	2.87691400
H	-1.11973500	1.49848700	2.63522300
H	-0.73470000	3.67796300	3.77622300

$[Zn(phen)_3]^{\bullet +}$

C	-3.09215100	-1.40845800	-2.90868600
C	-3.43330000	-2.63921600	-2.39873500
C	-2.79423400	-3.10887200	-1.23386100
C	-1.82550400	-2.27540000	-0.64514100
C	-2.10786500	-0.65349600	-2.25226700
C	-3.08879300	-4.38412800	-0.64210000
C	-1.14747400	-2.71340400	0.55314600
C	-1.45256700	-3.97126600	1.10390500
C	-2.44368300	-4.79901300	0.47540200

C	-0.75451800	-4.35692600	2.26610000
H	-0.96259000	-5.32084700	2.72081400
C	0.18251200	-3.50824200	2.80825000
C	0.41308100	-2.27025100	2.18880600
H	-3.83933300	-5.01098900	-1.11360700
H	-3.56257200	-1.01527600	-3.80260700
H	-4.18718700	-3.25376200	-2.88159200
H	-1.81308700	0.31865800	-2.63445100
H	-2.66535300	-5.76556900	0.91711800
H	0.73970500	-3.77494900	3.69895100
H	1.14206800	-1.57177600	2.58783600
N	-1.49240100	-1.06964100	-1.15906300
N	-0.23357600	-1.88323400	1.10264400
Zn	0.10600700	-0.00056200	-0.00032400
N	-0.21793100	1.88478600	-1.10288100
C	-1.12490900	2.72250100	-0.55333100
C	0.43259900	2.26688800	-2.18848100
C	-1.41882000	3.98329000	-1.10348200
C	-1.80732300	2.28968300	0.64434400
C	0.21308800	3.50721800	-2.80724600
H	1.15570700	1.56238900	-2.58761700
C	-0.71682000	4.36365700	-2.26503400
C	-2.40301700	4.81923300	-0.47498400
C	-2.76920000	3.13112700	1.23298200
N	-1.48492700	1.08078500	1.15774200
H	0.77304900	3.76966800	-3.69747600
H	-0.91624100	5.32966000	-2.71920100
C	-3.05232900	4.40928200	0.64190500
H	-2.61597800	5.78797700	-0.91619300
C	-3.41311300	2.66622800	2.39709400
C	-2.10470400	0.66927800	2.25026000
H	-3.79752000	5.04245400	1.11343700
H	-4.16211800	3.28687400	2.87976500
C	-3.08293500	1.43224700	2.90647600
H	-1.81858300	-0.30563400	2.63200300
H	-3.55739200	1.04253500	3.79978500
N	1.71236600	0.65242000	1.18724300
C	2.92930100	0.33154600	0.61297500
C	1.71656000	1.31973500	2.34930500
C	4.16165000	0.68729300	1.24745100
C	2.92693900	-0.35485100	-0.61206500
C	2.86693600	1.69883300	3.01762800
H	0.73884800	1.55667200	2.76086600
C	4.11660900	1.37043300	2.45123400
C	5.39163700	0.31198300	0.58992100
C	4.15676600	-0.72037400	-1.24588700
N	1.70784100	-0.66611100	-1.18697600
H	2.79591100	2.23600900	3.95665300
H	5.04092600	1.64974000	2.94959000
C	5.38933100	-0.35478200	-0.58771600
H	6.33008900	0.57967400	1.06859400
C	4.10695300	-1.40318200	-2.44966900
C	1.70734400	-1.33343900	-2.34903200
H	6.32589700	-0.62984800	-1.06589700
H	5.02929000	-1.68987000	-2.94750300
C	2.85502200	-1.72167600	-3.01674100

H	0.72798000	-1.56257900	-2.76106100
H	2.78025500	-2.25827500	-3.95580500

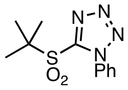

C	-2.88420900	-0.46741400	0.29206500
S	-1.11163800	-0.67462200	-0.18421600
O	-0.40645100	-1.40612800	0.87280000
O	-1.02276400	-1.16925800	-1.56226900
C	-0.36651300	0.97029100	-0.19857400
N	0.95702300	1.20761800	-0.12574100
N	1.12241900	2.53853900	-0.18015400
N	-0.04671500	3.06382200	-0.27963600
N	-1.00111200	2.11353900	-0.30094600
C	2.08843700	0.32758200	-0.02812200
C	2.36023300	-0.54605000	-1.07321900
C	2.89335700	0.40245600	1.10183800
C	3.46770800	-1.38185200	-0.97138800
H	1.71825000	-0.57280500	-1.94706900
C	4.00226600	-0.43245200	1.18598300
H	2.65079100	1.09685900	1.89896400
C	4.28673400	-1.32459500	0.15380700
H	3.69268800	-2.07249700	-1.77730700
H	4.64111300	-0.38813600	2.06182400
H	5.15156900	-1.97636700	0.22615700
C	-3.62786300	0.26062100	-0.82673300
H	-4.68698500	0.29278300	-0.55356400
H	-3.27609000	1.28546000	-0.95286600
H	-3.53833500	-0.27100900	-1.77681700
C	-2.96035300	0.26817500	1.62909800
H	-2.64342400	1.30926100	1.54446300
H	-4.00520100	0.25918100	1.95374800
H	-2.36493600	-0.23023500	2.39812000
C	-3.38391500	-1.91248000	0.43181600
H	-2.86220100	-2.44966500	1.22687600
H	-4.44567100	-1.86582200	0.68994500
H	-3.28823600	-2.47027500	-0.50334000

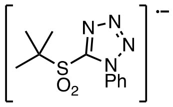

C	-2.44671900	-0.47854200	0.85042800
S	-1.39823100	-0.61066900	-0.69190700
O	-0.52887500	-1.79496500	-0.50471100
O	-2.33038300	-0.63804000	-1.84241300
C	-0.31586000	0.80639400	-0.94963100
N	0.80200700	1.02689400	-0.13883200
N	0.80463600	2.36707800	0.22415500
N	-0.24575800	2.88898600	-0.32264400
N	-0.95647100	2.02569300	-1.02159800
C	1.99044400	0.28825900	-0.01388500
C	2.30620500	-0.72242000	-0.92668200
C	2.87848200	0.58798000	1.02596700
C	3.49658300	-1.42744800	-0.79123400

H	1.61591700	-0.95258600	-1.72852300
C	4.06756800	-0.12272700	1.14643000
H	2.63130400	1.37329300	1.73050000
C	4.38494000	-1.13551000	0.24296100
H	3.73332300	-2.20902400	-1.50752700
H	4.74867500	0.11689200	1.95791700
H	5.31308400	-1.68982600	0.34269400
C	-3.50662600	0.59922300	0.63472100
H	-4.10544200	0.68395100	1.54836200
H	-3.05779600	1.57262400	0.42459100
H	-4.17362200	0.33866700	-0.19043200
C	-1.52386000	-0.12718700	2.01700800
H	-1.07896600	0.86517100	1.90121700
H	-2.11358500	-0.12328400	2.93995500
H	-0.72057400	-0.86019300	2.13253300
C	-3.09410400	-1.84889000	1.05531600
H	-2.34566300	-2.62515900	1.22929600
H	-3.74626900	-1.79508900	1.93423800
H	-3.70784500	-2.13603400	0.19673400

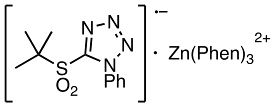

C	-5.39242700	-3.28351300	0.28170800
C	-6.20474000	-2.95926800	-0.77953000
C	-5.91266100	-1.82049000	-1.55658700
C	-4.78292400	-1.06371100	-1.19821600
C	-4.29149100	-2.46267800	0.56654500
C	-6.71350100	-1.41364400	-2.67677100
C	-4.43602700	0.10548900	-1.97133400
C	-5.24328200	0.48023200	-3.05994400
C	-6.39512800	-0.30913000	-3.39466900
C	-4.86803800	1.63199800	-3.78005100
H	-5.46665500	1.95348700	-4.62686900
C	-3.74765000	2.33425000	-3.40222800
C	-3.00137900	1.87870700	-2.30555800
H	-7.58268000	-2.01039500	-2.93446700
H	-5.58684500	-4.15273500	0.89912800
H	-7.06820900	-3.56969200	-1.02630500
H	-3.63531100	-2.68801200	1.40077300
H	-7.00440900	-0.00243100	-4.23905400
H	-3.43083000	3.22383900	-3.93391400
H	-2.10950600	2.40736500	-1.98566200
N	-3.99644000	-1.38703100	-0.14507900
N	-3.33059100	0.80002500	-1.61370800
Zn	-2.30935500	-0.02354800	0.13347000
N	-1.34713600	-1.24798600	1.66300000
C	-0.44503400	-2.12458900	1.16268100
C	-1.54097000	-1.20663200	2.97119400
C	0.28331400	-3.00716400	1.97990000
C	-0.23821400	-2.14393300	-0.26610200
C	-0.85668600	-2.04113500	3.86662200
H	-2.27021700	-0.48847100	3.33156700
C	0.05321300	-2.94462600	3.36890500
C	1.21292700	-3.92387700	1.38222500

C	0.67565100	-3.05677800	-0.82129000
N	-0.94112600	-1.26865800	-1.02226500
H	-1.05637100	-1.96505900	4.92911300
H	0.59709000	-3.60992500	4.03270900
C	1.39787800	-3.95031400	0.04001800
H	1.76550100	-4.59585800	2.03125500
C	0.83717300	-3.04374300	-2.22127200
C	-0.77206900	-1.27884500	-2.33452900
H	2.10123900	-4.64423700	-0.40910000
H	1.53505300	-3.73286000	-2.68660800
C	0.11055400	-2.15630300	-2.97995700
H	-1.35451400	-0.56242600	-2.90434000
H	0.21106400	-2.11797700	-4.05832400
N	-0.93449200	1.64423900	0.35507000
C	-1.37889600	2.60635200	1.19740700
C	0.25256700	1.79084000	-0.21159700
C	-0.63334800	3.76415400	1.47983400
C	-2.66871000	2.42105600	1.81870700
C	1.07406800	2.90579100	0.01283100
H	0.57502000	0.99543200	-0.87550300
C	0.62504600	3.89410500	0.85778100
C	-1.17036500	4.75262500	2.37247900
C	-3.16895100	3.40977000	2.68463100
N	-3.35110500	1.28532700	1.54111200
H	2.03950900	2.96168700	-0.48064600
H	1.22963500	4.77469300	1.05388700
C	-2.38649800	4.58482200	2.94695100
H	-0.57931400	5.64008800	2.57586400
C	-4.43497200	3.18675600	3.26176000
C	-4.53901800	1.10206500	2.09440700
H	-2.79064000	5.33533400	3.61905300
H	-4.85480200	3.92773300	3.93519300
C	-5.12295700	2.03320500	2.96540900
H	-5.05465100	0.18050300	1.84475700
H	-6.09873900	1.83037600	3.39117100
C	6.58895800	1.77897500	-1.72483100
S	4.92253900	1.59991000	-0.89411400
O	5.15139200	1.80761600	0.55406400
O	4.00432000	2.54861900	-1.56800300
C	4.18622000	-0.03555700	-1.08348000
N	4.69062100	-1.14029600	-0.39098800
N	4.80937200	-2.18867600	-1.29203700
N	4.41540900	-1.72667800	-2.43418600
N	4.04092900	-0.46239800	-2.38696600
C	4.69779200	-1.42030000	0.98561900
C	3.93653700	-0.65737700	1.87608500
C	5.46248300	-2.49023100	1.46490600
C	3.94938600	-0.96234200	3.23205400
H	3.34847100	0.17153900	1.50247100
C	5.46165600	-2.78686400	2.82350300
H	6.05114400	-3.07983200	0.77190200
C	4.70936300	-2.02609900	3.71678500
H	3.35293300	-0.36386600	3.91481700
H	6.06033600	-3.61789500	3.18509700
H	4.71549700	-2.25863000	4.77725000
C	6.38694700	1.78339200	-3.23819300

H	7.36775500	1.87133400	-3.71852900
H	5.91391700	0.86243100	-3.58614600
H	5.77305100	2.63052200	-3.55321900
C	7.45939600	0.60395700	-1.28133800
H	7.06713700	-0.35205000	-1.63960000
H	8.46186700	0.73293100	-1.70303700
H	7.55396900	0.55919800	-0.19285900
C	7.17112900	3.10940600	-1.24574500
H	7.33006400	3.11329100	-0.16509600
H	8.13881300	3.26091600	-1.73709400
H	6.52298100	3.94977700	-1.51044400

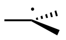

C	-0.00013100	0.00004000	-0.17324100
C	0.89496700	1.18165800	0.01722900
H	1.05981600	1.39534300	1.08941200
H	1.88450800	1.01801000	-0.42504500
H	0.46776900	2.09092100	-0.42127100
C	0.57601600	-1.36577200	0.01722200
H	1.57572300	-1.45095900	-0.42428700
H	0.68219100	-1.61384200	1.08941700
H	-0.06179500	-2.14153600	-0.42201400
C	-1.47097400	0.18412000	0.01724000
H	-2.04464300	-0.63959700	-0.42317100
H	-1.73883300	0.21760400	1.08942700
H	-1.82400600	1.12377800	-0.42316400

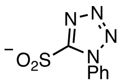

S	-2.06057300	-1.26248800	0.31789900
O	-1.13478000	-1.67739000	1.44532700
O	-1.70406500	-1.95095400	-0.98882900
C	-1.48699400	0.48067000	-0.00803400
N	-0.25325700	1.01563800	-0.12261700
N	-0.39143200	2.34448100	-0.30800200
N	-1.65100900	2.59246000	-0.30078900
N	-2.36154700	1.46027500	-0.11617100
C	1.05025300	0.42849300	-0.08698800
C	1.28489300	-0.76687000	-0.75630700
C	2.06283300	1.09212800	0.59887900
C	2.56602000	-1.30956700	-0.72710100
H	0.47117600	-1.26916600	-1.26983100
C	3.34065500	0.54294900	0.61028700
H	1.85022500	2.02086800	1.11626500
C	3.59405600	-0.65748600	-0.05019800
H	2.76007400	-2.24504500	-1.24237200
H	4.13694400	1.05276000	1.14319300
H	4.59191900	-1.08473900	-0.03579100

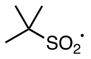

C	0.87911000	-0.00002000	0.02642700
S	-1.00118000	0.00000400	-0.36527600

O	-1.56966900	-1.27192700	0.14977400
O	-1.56945500	1.27196300	0.14987300
C	1.43771900	1.26609000	-0.60437500
H	2.51329300	1.29721700	-0.40132700
H	1.30087500	1.27616400	-1.68958400
H	0.98548900	2.16467400	-0.17907500
C	1.43741100	-1.26723100	-0.60237700
H	1.29929800	-1.27971200	-1.68739800
H	2.51325900	-1.29746400	-0.40061900
H	0.98602600	-2.16508300	-0.17465100
C	0.98410500	0.00112300	1.54737700
H	0.52712300	-0.88959400	1.98531300
H	2.04816000	0.00056600	1.80837400
H	0.52828300	0.89311100	1.98390000

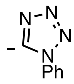

C	-1.88038900	1.13862400	-0.35872700
N	-1.06506600	0.09042600	-0.02008800
N	-1.77781400	-1.01378900	0.32939000
N	-3.00699000	-0.67563700	0.21811300
N	-3.11384800	0.61839700	-0.19823000
C	0.35345700	0.04263500	-0.01156000
C	1.08280500	1.22066700	0.14724300
C	1.01563600	-1.17565200	-0.16270400
C	2.47341300	1.17587200	0.14856300
H	0.55539800	2.15915200	0.26842700
C	2.40705900	-1.21035900	-0.14911400
H	0.44233500	-2.08575700	-0.29274900
C	3.14279300	-0.03762000	0.00369500
H	3.03530300	2.09691400	0.27232300
H	2.91630400	-2.16229300	-0.26672600
H	4.22802700	-0.06880200	0.01006300
